# Supplementary material for: NHANES 2011–2014 Reveals Decreased Cognitive Performance in U.S. Older Adults with Metabolic Syndrome Combinations
Source: Int J Environ Res Public Health. 2023 Mar 24;20(7):5257. doi: 10.3390/ijerph20075257 (PMC10093810; doi:10.3390/ijerph20075257)
Supplement: Supplementary file 1 [file ijerph-20-05257-s001.zip › ijerph-2135158-supplementary.pdf]

Supplementary material

This section presents the complete multiple linear regression analysis shown partially in Table 5. Remember that the analysis involved 92 models from considering the 23 predictors from the MetS combinations and the four cognitive tests used as dependent variables. The estimated marginal means for each category of covariates and predictors are also shown. Please see the scheme below for the easy location of the results.

- CERAD–IR versus classical MetS diagnosis and other covariates: Table S1 (p. 2).
- CERAD–IR versus the ten combinations of three criteria and other covariates: Tables S2–S11 (pp. 3–12).
- CERAD–IR versus the one combination of all combinations of three criteria and other covariates: Table S12 (p. 13).
- CERAD–IR versus the five combinations of four criteria and other covariates: Table S13–S17 (pp. 14–18).
- CERAD–IR versus the one combination of all combinations of four criteria and other covariates: Table S18 (p. 19).
- CERAD–IR versus the one combination of five criteria and other covariates: Table S19 (p. 20).
- CERAD–IR versus the one combination of all combinations with abdominal obesity and other covariates: Table S20 (p. 21).
- CERAD–IR versus the one combination of all combinations without abdominal obesity and other covariates: Table S21 (p. 22).
- CERAD–IR versus the one combination of all combinations with hyperglycemia and other covariates: Table S22 (p. 23).
- CERAD–IR versus the one combination of all combinations without hyperglycemia and other covariates: Table S23 (p. 24).

- CERAD–DR versus classical MetS diagnosis and other covariates: Table S24 (p. 25).
- CERAD–DR versus the ten combinations of three criteria and other covariates: Tables S25–S34 (pp. 26–35).
- CERAD–DR versus the one combination of all combinations of three criteria and other covariates: Table S35 (p. 36).
- CERAD–DR versus the five combinations of four criteria and other covariates: Table S36–S40 (pp. 37–41).
- CERAD–DR versus the one combination of all combinations of four criteria and other covariates: Table S41 (p. 42).
- CERAD–DR versus the one combination of five criteria and other covariates: Table S42 (p. 43).
- CERAD–DR versus the one combination of all combinations with abdominal obesity and other covariates: Table S43 (p. 44).
- CERAD–DR versus the one combination of all combinations without abdominal obesity and other covariates: Table S44 (p. 45).
- CERAD–DR versus the one combination of all combinations with hyperglycemia and other covariates: Table S45 (p. 46).
- CERAD–DR versus the one combination of all combinations without hyperglycemia and other covariates: Table S46 (p. 47).

- AFT versus classical MetS diagnosis and other covariates: Table S47 (p. 48).
- AFT versus the ten combinations of three criteria and other covariates: Tables S48–S57 (pp. 49–58).
- AFT versus the one combination of all combinations of three criteria and other covariates: Table S58 (p. 59).
- AFT versus the five combinations of four criteria and other covariates: Table S59–S63 (pp. 60–64).
- AFT versus the one combination of all combinations of four criteria and other covariates: Table S64 (p. 65).
- AFT versus the one combination of five criteria and other covariates: Table S65 (p. 66).
- AFT versus the one combination of all combinations with abdominal obesity and other covariates: Table S66 (p. 67).
- AFT versus the one combination of all combinations without abdominal obesity and other covariates: Table S67 (p. 68).
- AFT versus the one combination of all combinations with hyperglycemia and other covariates: Table S68 (p. 69).
- AFT versus the one combination of all combinations without hyperglycemia and other covariates: Table S69 (p. 70).

- DSST versus classical MetS diagnosis and other covariates: Table S70 (p. 71).
- DSST versus the ten combinations of three criteria and other covariates: Tables S71–S80 (pp. 72–81).
- DSST versus the one combination of all combinations of three criteria and other covariates: Table S81 (p. 82).
- DSST versus the five combinations of four criteria and other covariates: Table S82–S86 (pp. 83–87).
- DSST versus the one combination of all combinations of four criteria and other covariates: Table S87 (p. 88).
- DSST versus the one combination of five criteria and other covariates: Table S88 (p. 89).
- DSST versus the one combination of all combinations with abdominal obesity and other covariates: Table S89 (p. 90).
- DSST versus the one combination of all combinations without abdominal obesity and other covariates: Table S90 (p. 91).
- DSST versus the one combination of all combinations with hyperglycemia and other covariates: Table S91 (p. 92).
- DSST versus the one combination of all combinations without hyperglycemia and other covariates: Table S92 (p. 93).

Table S1. Multiple linear regression model explaining the relationship between CERAD-IR and classic MetS diagnosis after adjusting by sociodemographic characteristics and medical history, and estimated marginal means for each category of predictors and covariates

**Model coefficient**	**Categories (for categorical predictor and covariates)**	*Beta* (*SE*)	95% CI	*t* (*p*)	Estimated marginal means[a]
Constant	Not applied (model intercept)	34.71 (2.49)	[29.64, 39.77]	13.95 (< 0.001)	Not applied (model intercept)
Education level	Up to 12th grade vs. *college graduate or above*	–1.50 (0.78)	[–3.09, 0.09]	–1.92 (0.064)	18.82 (0.87) vs. *20.32 (0.84)*
	High school graduate vs. *college graduate or above*	–1.16 (0.59)	[–2.37, 0.05]	–1.95 (0.060)	19.16 (0.54) vs. *20.32 (0.84)*
	Some college or AA degree vs. *college graduate or above*	0.02 (0.61)	[–1.22, 1.25]	0.03 (0.978)	20.34 (0.80) vs. *20.32 (0.84)*
Gender	Males vs. *females*	–1.22 (0.37)	[–1.98, –0.47]	–3.31 (0.002)	19.05 (0.67) vs. *20.27 (0.73)*
Race	Mexican American vs. *Non-Hispanic White*	–1.87 (0.66)	[–3.21, –0.53]	–2.84 (0.008)	19.01 (0.88) vs. *20.88 (0.71)*
	Other Hispanic vs. *Non-Hispanic White*	–2.39 (0.71)	[–3.82, –0.95]	–3.38 (0.002)	18.49 (0.77) vs. *20.88 (0.71)*
	Non–Hispanic Black vs. *Non-Hispanic White*	–0.98 (0.49)	[–1.98, 0.03]	–1.98 (0.057)	19.90 (0.74) vs. *20.88 (0.71)*
	Non–Hispanic Asian vs. *Non-Hispanic White*	0.67 (0.91)	[–1.18, 2.52]	0.74 (0.467)	21.55 (0.90) vs. *20.88 (0.71)*
	Other Race and Multi-Racial vs. *Non-Hispanic White*	–2.74 (1.13)	[–5.05, –0.43]	–2.42 (0.022)	18.14 (1.21) vs. *20.88 (0.71)*
Marital status	Single or never married vs. *married or living with a partner*	1.65 (0.97)	[–0.33, 3.63]	1.70 (0.100)	21.08 (1.03) vs. *19.43 (0.65)*
	Divorced or separated vs. *married or living with a partner*	–0.24 (0.57)	[–1.41, 0.92]	–0.42 (0.677)	19.19 (0.99) vs. *19.43 (0.65)*
	Widowed vs. *married or living with a partner*	–0.50 (0.57)	[–1.67, 0.67]	–0.88 (0.388)	18.93 (0.67) vs. *19.43 (0.65)*
Self-report general health condition	Poor vs. *excellent or very good*	3.68 (0.92)	[1.80, 5.56]	3.99 (< 0.001)	21.86 (1.04) vs. *18.18 (0.71)*
	Good or fair vs. *excellent or very good*	0.75 (0.44)	[–0.15, 1.66]	1.70 (0.098)	18.94 (0.60) vs. *18.18 (0.71)*
Difficulties in thinking or remembering	Yes vs. *no*	–1.75 (0.48)	[–2.71, –0.78]	–3.68 (0.001)	18.79 (0.80) vs. *20.53 (0.61)*
Ever told you have a heart disease	Yes vs. *no*	–0.73 (0.67)	[–2.10, 0.64]	–1.09 (0.285)	19.30 (0.87) vs. *20.03 (0.61)*
Ever told you had a stroke	Yes vs. *no*	0.58 (0.83)	[–1.12, 2.28]	0.69 (0.494)	19.95 (0.95) vs. *19.37 (0.60)*
Ever told you have diabetes	Yes vs. *no*	–0.66 (0.70)	[–2.10, 0.77]	–0.94 (0.354)	19.33 (0.77) vs. *19.99 (0.75)*
Have smoked at least 100 cigarettes in life	Yes vs. *no*	–0.33 (0.39)	[–1.12, 0.47]	–0.84 (0.406)	19.50 (0.59) vs. *19.82 (0.80)*
Classic MetS diagnosis (all three or more criteria)	MetS vs. *metabolically–healthy people*	–1.27 (0.51)	[–2.31, –0.23]	–2.48 (0.019)	19.03 (0.64) vs. *20.29 (0.79)*
Age at screening	Not applied (continuous covariate)	–0.17 (0.04)	[–0.24, –0.10]	–4.91 (< 0.001)	Not applied (continuous cov.)
Ratio of family income to the poverty level	Not applied (continuous covariate)	–0.06 (0.11)	[–0.30, 0.17]	–0.57 (0.573)	Not applied (continuous cov.)
Depression raw scores	Not applied (continuous covariate)	–0.04 (0.05)	[–0.14, 0.06]	–0.84 (0.408)	Not applied (continuous cov.)

[a]The column labeled "estimated marginal means" shows each category's marginal means against the reference category's marginal mean. The reference categories and the marginal means of the reference categories are shown in italics. The significant results have been colored.

Table S2. Multiple linear regression model explaining the relationship between CERAD-IR and specific combination AO + TRI + HDL after adjusting by sociodemographic characteristics and medical history, and estimated marginal means for each category of predictors and covariates[a]

**Model coefficient**	**Categories (for categorical predictor and covariates)**	*Beta* (*SE*)	95% CI	*t* (*p*)	Estimated marginal means[b]
Constant	Not applied (model intercept)	47.23 (4.84)	[36.77, 57.68]	9.76 (< 0.001)	Not applied (model intercept)
Education level	Up to 12th grade vs. *college graduate or above*	–0.35 (0.97)	[–2.45, 1.75]	–0.36 (0.725)	20.47 (1.17) vs. *20.82 (0.79)*
	High school graduate vs. *college graduate or above*	–2.87 (1.19)	[–5.43, –0.30]	–2.41 (0.031)	17.95 (1.36) vs. *20.82 (0.79)*
	Some college or AA degree vs. *college graduate or above*	–0.30 (1.05)	[–2.57, 1.97]	–0.29 (0.777)	20.52 (0.95) vs. *20.82 (0.79)*
Gender	Males vs. *females*	–0.13 (0.49)	[–1.18, 0.93]	–0.26 (0.802)	19.88 (0.94) vs. *20.00 (0.89)*
Race	Mexican American vs. *Non-Hispanic White*	–3.42 (1.25)	[–6.12, –0.71]	–2.73 (0.017)	18.92 (2.09) vs. *22.34 (1.39)*
	Other Hispanic vs. *Non-Hispanic White*	–2.98 (1.56)	[–6.35, 0.39]	–1.91 (0.078)	19.36 (0.94) vs. *22.34 (1.39)*
	Non–Hispanic Black vs. *Non-Hispanic White*	–3.73 (1.95)	[–7.94, 0.47]	–1.92 (0.078)	18.61 (1.37) vs. *22.34 (1.39)*
	Non–Hispanic Asian vs. *Non-Hispanic White*	–1.87 (1.77)	[–5.70, 1.95]	–1.06 (0.309)	20.47 (1.35) vs. *22.34 (1.39)*
	Other Race and Multi-Racial vs. *Non-Hispanic White*	No cases	No cases	No cases	No cases
Marital status	Single or never married vs. *married or living with a partner*	0.77 (1.97)	[–3.49, 5.04]	0.39 (0.701)	20.18 (2.23) vs. *19.41 (0.74)*
	Divorced or separated vs. *married or living with a partner*	0.61 (1.34)	[–2.29, 3.51]	0.45 (0.658)	20.02 (1.64) vs. *19.41 (0.74)*
	Widowed vs. *married or living with a partner*	0.74 (1.22)	[–1.90, 3.38]	0.61 (0.553)	20.15 (1.08) vs. *19.41 (0.74)*
Self-report general health condition	Poor vs. *excellent or very good*	2.46 (1.93)	[–1.71, 6.63]	1.27 (0.226)	21.62 (1.63) vs. *19.16 (0.95)*
	Good or fair vs. *excellent or very good*	–0.13 (0.63)	[–1.49, 1.24]	–0.20 (0.846)	19.04 (1.03) vs. *19.16 (0.95)*
Difficulties in thinking or remembering	Yes vs. *no*	–0.47 (0.93)	[–2.48, 1.55]	–0.50 (0.624)	19.71 (0.95) vs. *20.17 (1.04)*
Ever told you have a heart disease	Yes vs. *no*	–1.70 (1.12)	[–4.13, 0.73]	–1.51 (0.155)	19.09 (1.07) vs. *20.79 (1.02)*
Ever told you had a stroke	Yes vs. *no*	1.09 (1.70)	[–2.59, 4.76]	0.64 (0.534)	20.48 (1.40) vs. *19.40 (1.02)*
Ever told you have diabetes	Yes vs. *no*	2.96 (1.20)	[0.37, 5.54]	2.47 (0.028)	21.42 (1.15) vs. *18.46 (0.98)*
Have smoked at least 100 cigarettes in life	Yes vs. *no*	0.17 (0.91)	[–1.79, 2.13]	0.19 (0.855)	20.03 (0.66) vs. *19.86 (1.24)*
Three criteria combination: AO + TRI + HDL	AO + TRI + HDL vs. *metabolically–healthy people*	–4.39 (1.32)	[–7.23, –1.55]	–3.33 (0.005)	17.75 (1.36) vs. *22.14 (0.76)*
Age at screening	Not applied (continuous covariate)	–0.35 (0.07)	[–0.51, –0.20]	–4.87 (< 0.001)	Not applied (continuous cov.)
Ratio of family income to the poverty level	Not applied (continuous covariate)	–0.39 (0.19)	[–0.79, 0.01]	–2.08 (0.058)	Not applied (continuous cov.)
Depression raw scores	Not applied (continuous covariate)	0.10 (0.08)	[–0.07, 0.26]	1.29 (0.221)	Not applied (continuous cov.)

[a]The design-based covariance matrix is singular. The validity of results is uncertain. [b]The column labeled "estimated marginal means" shows each category's marginal means against the reference category's marginal mean. The reference categories and the marginal means of the reference categories are shown in italics. The significant results have been colored.

Table S3. Multiple linear regression model explaining the relationship between CERAD-IR and specific combination AO + TRI + HBP after adjusting by sociodemographic characteristics and medical history, and estimated marginal means for each category of predictors and covariates[a]

**Model coefficient**	**Categories (for categorical predictor and covariates)**	*Beta* (*SE*)	95% CI	*t* (*p*)	Estimated marginal means[b]
Constant	Not applied (model intercept)	46.59 (2.92)	[40.34, 52.85]	15.98 (< 0.001)	Not applied (model intercept)
Education level	Up to 12th grade vs. *college graduate or above*	–0.27 (1.05)	[–2.53, 1.99]	–0.26 (0.802)	19.64 (1.39) vs. *19.91 (0.82)*
	High school graduate vs. *college graduate or above*	–3.09 (0.92)	[–5.06, –1.13]	–3.37 (0.005)	16.81 (1.08) vs. *19.91 (0.82)*
	Some college or AA degree vs. *college graduate or above*	–1.23 (0.87)	[–3.10, 0.63]	–1.42 (0.179)	18.67 (1.24) vs. *19.91 (0.82)*
Gender	Males vs. *females*	–1.40 (0.46)	[–2.39, –0.41]	–3.03 (0.009)	18.06 (1.09) vs. *19.46 (0.92)*
Race	Mexican American vs. *Non-Hispanic White*	–1.53 (1.47)	[–4.67, 1.62]	–1.04 (0.316)	18.33 (1.97) vs. *19.86 (1.29)*
	Other Hispanic vs. *Non-Hispanic White*	–1.23 (1.80)	[–5.08, 2.63]	–0.68 (0.506)	18.63 (1.20) vs. *19.86 (1.29)*
	Non–Hispanic Black vs. *Non-Hispanic White*	–2.24 (1.59)	[–5.64, 1.17]	–1.41 (0.180)	17.62 (1.20) vs. *19.86 (1.29)*
	Non–Hispanic Asian vs. *Non-Hispanic White*	–0.53 (1.57)	[–3.89, 2.84]	–0.33 (0.743)	19.34 (1.99) vs. *19.86 (1.29)*
	Other Race and Multi-Racial vs. *Non-Hispanic White*	No cases	No cases	No cases	No cases
Marital status	Single or never married vs. *married or living with a partner*	–1.45 (2.53)	[–6.87, 3.97]	–0.57 (0.576)	17.66 (2.60) vs. *19.11 (0.99)*
	Divorced or separated vs. *married or living with a partner*	0.52 (1.48)	[–2.65, 3.68]	0.35 (0.732)	19.62 (1.50) vs. *19.11 (0.99)*
	Widowed vs. *married or living with a partner*	–0.46 (1.05)	[–2.72, 1.80]	–0.44 (0.668)	18.64 (1.17) vs. *19.11 (0.99)*
Self-report general health condition	Poor vs. *excellent or very good*	No cases	No cases	No cases	No cases
	Good or fair vs. *excellent or very good*	0.55 (0.67)	[–0.89, 1.99]	0.82 (0.425)	19.03 (1.05) vs. *18.48 (1.03)*
Difficulties in thinking or remembering	Yes vs. *no*	–0.55 (1.15)	[–3.02, 1.91]	–0.48 (0.637)	18.48 (1.32) vs. *19.03 (0.93)*
Ever told you have a heart disease	Yes vs. *no*	–1.19 (0.76)	[–2.82, 0.43]	–1.58 (0.137)	18.16 (1.03) vs. *19.35 (1.08)*
Ever told you had a stroke	Yes vs. *no*	3.46 (2.24)	[–1.35, 8.27]	1.54 (0.145)	20.49 (1.93) vs. *17.03 (0.84)*
Ever told you have diabetes	Yes vs. *no*	–1.44 (1.19)	[–3.99, 1.11]	–1.21 (0.246)	18.04 (1.01) vs. *19.48 (1.28)*
Have smoked at least 100 cigarettes in life	Yes vs. *no*	0.70 (0.85)	[–1.12, 2.52]	0.83 (0.422)	19.11 (0.93) vs. *18.41 (1.19)*
Three criteria combination: AO + TRI + HBP	AO + TRI + HBP vs. *metabolically–healthy people*	–3.95 (0.69)	[–5.42, –2.48]	–5.76 (< 0.001)	16.78 (1.16) vs. *20.73 (0.90)*
Age at screening	Not applied (continuous covariate)	–0.35 (0.04)	[–0.44, –0.25]	–7.88 (< 0.001)	Not applied (continuous cov.)
Ratio of family income to the poverty level	Not applied (continuous covariate)	–0.05 (0.25)	[–0.57, 0.48]	–0.19 (0.854)	Not applied (continuous cov.)
Depression raw scores	Not applied (continuous covariate)	0.07 (0.08)	[–0.10, 0.24]	0.88 (0.392)	Not applied (continuous cov.)

[a]The design-based covariance matrix is singular. The validity of results is uncertain. [b]The column labeled "estimated marginal means" shows each category's marginal means against the reference category's marginal mean. The reference categories and the marginal means of the reference categories are shown in italics. The significant results have been colored.

Table S4. Multiple linear regression model explaining the relationship between CERAD-IR and specific combination AO + TRI + GLY after adjusting by sociodemographic characteristics and medical history, and estimated marginal means for each category of predictors and covariates[a]

**Model coefficient**	**Categories (for categorical predictor and covariates)**	*Beta* (*SE*)	95% CI	*t* (*p*)	Estimated marginal means[b]
Constant	Not applied (model intercept)	39.43 (4.45)	[30.17 48.69]	8.85 (< 0.001)	Not applied (model intercept)
Education level	Up to 12th grade vs. *college graduate or above*	–0.73 (1.59)	[–4.03 2.57]	–0.46 (0.650)	18.54 (1.71) vs. *19.27 (2.47)*
	High school graduate vs. *college graduate or above*	–2.71 (1.34)	[–5.50 0.09]	–2.02 (0.057)	16.56 (1.75) vs. *19.27 (2.47)*
	Some college or AA degree vs. *college graduate or above*	–1.09 (1.24)	[–3.68 1.50]	–0.88 (0.391)	18.18 (2.22) vs. *19.27 (2.47)*
Gender	Males vs. *females*	–1.15 (0.68)	[–2.57 0.27]	–1.68 (0.108)	17.56 (1.74) vs. *18.71 (2.09)*
Race	Mexican American vs. *Non-Hispanic White*	–3.72 (1.12)	[–6.05 –1.40]	–3.33 (0.003)	16.26 (2.34) vs. *19.99 (1.55)*
	Other Hispanic vs. *Non-Hispanic White*	–1.60 (1.75)	[–5.25 2.04]	–0.92 (0.370)	18.38 (2.36) vs. *19.99 (1.55)*
	Non–Hispanic Black vs. *Non-Hispanic White*	–2.20 (1.79)	[–5.91 1.52]	–1.23 (0.232)	17.79 (2.90) vs. *19.99 (1.55)*
	Non–Hispanic Asian vs. *Non-Hispanic White*	–1.72 (1.39)	[–4.61 1.17]	–1.24 (0.229)	18.26 (1.69) vs. *19.99 (1.55)*
	Other Race and Multi-Racial vs. *Non-Hispanic White*	No cases	No cases	No cases	No cases
Marital status	Single or never married vs. *married or living with a partner*	–0.62 (1.34)	[–3.41 2.17]	–0.46 (0.650)	17.50 (2.23) vs. *18.12 (1.87)*
	Divorced or separated vs. *married or living with a partner*	0.60 (0.95)	[–1.38 2.57]	0.63 (0.537)	18.72 (2.01) vs. *18.12 (1.87)*
	Widowed vs. *married or living with a partner*	0.08 (1.07)	[–2.16 2.31]	0.07 (0.943)	18.20 (2.22) vs. *18.12 (1.87)*
Self-report general health condition	Poor vs. *excellent or very good*	2.75 (2.59)	[–2.64 8.14]	1.06 (0.301)	19.76 (3.25) vs. *17.01 (1.52)*
	Good or fair vs. *excellent or very good*	0.64 (1.01)	[–1.46 2.73]	0.63 (0.533)	17.65 (1.42) vs. *17.01 (1.52)*
Difficulties in thinking or remembering	Yes vs. *no*	–0.80 (1.30)	[–3.51 1.91]	–0.61 (0.547)	17.74 (2.14) vs. *18.54 (1.85)*
Ever told you have a heart disease	Yes vs. *no*	–1.51 (1.24)	[–4.09 1.07]	–1.22 (0.237)	17.38 (2.36) vs. *18.89 (1.53)*
Ever told you had a stroke	Yes vs. *no*	–2.01 (2.30)	[–6.80 2.77]	–0.87 (0.392)	17.13 (2.84) vs. *19.14 (1.31)*
Ever told you have diabetes	Yes vs. *no*	–1.20 (2.92)	[–7.28 4.88]	–0.41 (0.685)	17.54 (2.21) vs. *18.74 (2.56)*
Have smoked at least 100 cigarettes in life	Yes vs. *no*	0.14 (1.28)	[–2.52 2.79]	0.11 (0.915)	18.20 (2.18) vs. *18.07 (1.80)*
Three criteria combination: AO + TRI + GLY	AO + TRI + HBP vs. *metabolically–healthy people*	–0.16 (0.96)	[–2.16 1.83]	–0.17 (0.868)	18.06 (1.87) vs. *18.22 (2.03)*
Age at screening	Not applied (continuous covariate)	–0.23 (0.06)	[–0.36 –0.11]	–3.78 (0.001)	Not applied (continuous cov.)
Ratio of family income to the poverty level	Not applied (continuous covariate)	0.01 (0.24)	[–0.50 0.51]	0.03 (0.974)	Not applied (continuous cov.)
Depression raw scores	Not applied (continuous covariate)	–0.13 (0.13)	[–0.40 0.14]	–0.98 (0.340)	Not applied (continuous cov.)

[a]The design-based covariance matrix is singular. The validity of results is uncertain. [b]The column labeled "estimated marginal means" shows each category's marginal means against the reference category's marginal mean. The reference categories and the marginal means of the reference categories are shown in italics. The significant results have been colored.

Table S5. Multiple linear regression model explaining the relationship between CERAD-IR and specific combination AO + HDL + HBP after adjusting by sociodemographic characteristics and medical history, and estimated marginal means for each category of predictors and covariates[a]

**Model coefficient**	**Categories (for categorical predictor and covariates)**	*Beta* (*SE*)	95% CI	*t* (*p*)	Estimated marginal means[b]
Constant	Not applied (model intercept)	51.01 (5.35)	[39.68, 62.34]	9.54 (< 0.001)	Not applied (model intercept)
Education level	Up to 12th grade vs. *college graduate or above*	–0.33 (1.39)	[–3.27, 2.61]	–0.24 (0.814)	17.40 (1.54) vs. *17.73 (1.54)*
	High school graduate vs. *college graduate or above*	–4.36 (1.44)	[–7.42, –1.30]	–3.02 (0.008)	13.38 (1.31) vs. *17.73 (1.54)*
	Some college or AA degree vs. *college graduate or above*	0.01 (1.31)	[–2.76, 2.78]	0.01 (0.992)	17.74 (1.51) vs. *17.73 (1.54)*
Gender	Males vs. *females*	0.38 (0.71)	[–1.12, 1.89]	0.54 (0.596)	16.75 (1.24) vs. *16.37 (1.36)*
Race	Mexican American vs. *Non-Hispanic White*	–3.93 (1.22)	[–6.51, –1.35]	–3.23 (0.005)	15.49 (1.97) vs. *19.42 (1.32)*
	Other Hispanic vs. *Non-Hispanic White*	–2.88 (1.75)	[–6.60, 0.84]	–1.64 (0.120)	16.54 (1.84) vs. *19.42 (1.32)*
	Non–Hispanic Black vs. *Non-Hispanic White*	–2.10 (0.86)	[–3.92, –0.28]	–2.45 (0.026)	17.32 (1.14) vs. *19.42 (1.32)*
	Non–Hispanic Asian vs. *Non-Hispanic White*	–0.12 (1.71)	[–3.74, 3.50]	–0.07 (0.945)	19.30 (1.57) vs. *19.42 (1.32)*
	Other Race and Multi-Racial vs. *Non-Hispanic White*	–8.13 (1.72)	[–11.77, –4.49]	–4.73 (< 0.001)	11.30 (2.41) vs. *19.42 (1.32)*
Marital status	Single or never married vs. *married or living with a partner*	1.33 (2.00)	[–2.92, 5.58]	0.66 (0.516)	16.73 (2.31) vs. *15.40 (1.25)*
	Divorced or separated vs. *married or living with a partner*	1.33 (0.81)	[–0.39, 3.04]	1.64 (0.120)	16.73 (1.07) vs. *15.40 (1.25)*
	Widowed vs. *married or living with a partner*	1.99 (0.93)	[0.02, 3.97]	2.14 (0.048)	17.39 (1.72) vs. *15.40 (1.25)*
Self-report general health condition	Poor vs. *excellent or very good*	No cases	No cases	No cases	No cases
	Good or fair vs. *excellent or very good*	–0.82 (0.74)	[–2.40, 0.76]	–1.10 (0.289)	16.15 (1.34) vs. *17.01 (1.52)*
Difficulties in thinking or remembering	Yes vs. *no*	–0.88 (1.02)	[–3.05, 1.29]	–0.86 (0.401)	17.74 (2.14) vs. *18.54 (1.85)*
Ever told you have a heart disease	Yes vs. *no*	–1.16 (1.17)	[–3.64, 1.31]	–0.99 (0.335)	17.38 (2.36) vs. *18.89 (1.53)*
Ever told you had a stroke	Yes vs. *no*	–3.07 (2.00)	[–7.30, 1.15]	–1.54 (0.143)	17.13 (2.84) vs. *19.14 (1.31)*
Ever told you have diabetes	Yes vs. *no*	–0.50 (0.97)	[–2.54, 1.55]	–0.51 (0.615)	17.54 (2.21) vs. *18.74 (2.56)*
Have smoked at least 100 cigarettes in life	Yes vs. *no*	0.93 (1.04)	[–1.28, 3.14]	0.89 (0.386)	18.20 (2.18) vs. *18.07 (1.80)*
Three criteria combination: AO + HDL + HBP	AO + HDL + HBP vs. *metabolically–healthy people*	–0.98 (1.32)	[–3.78, 1.83]	–0.74 (0.471)	18.06 (1.87) vs. *18.22 (2.03)*
Age at screening	Not applied (continuous covariate)	–0.41 (0.08)	[–0.59, –0.23]	–4.94 (< 0.001)	Not applied (continuous cov.)
Ratio of family income to the poverty level	Not applied (continuous covariate)	–0.47 (0.21)	[–0.91, –0.02]	–2.23 (0.041)	Not applied (continuous cov.)
Depression raw scores	Not applied (continuous covariate)	0.13 (0.12)	[–0.12, 0.39]	1.12 (0.279)	Not applied (continuous cov.)

[a]The design-based covariance matrix is singular. The validity of results is uncertain. [b]The column labeled "estimated marginal means" shows each category's marginal means against the reference category's marginal mean. The reference categories and the marginal means of the reference categories are shown in italics. The significant results have been colored.

Table S6. Multiple linear regression model explaining the relationship between CERAD-IR and specific combination AO + HDL + GLY after adjusting by sociodemographic characteristics and medical history, and estimated marginal means for each category of predictors and covariates[a]

**Model coefficient**	**Categories (for categorical predictor and covariates)**	*Beta* (*SE*)	95% CI	*t* (*p*)	Estimated marginal means[b]
Constant	Not applied (model intercept)	36.60 (3.73)	[28.88, 44.32]	9.81 (< 0.001)	Not applied (model intercept)
Education level	Up to 12th grade vs. *college graduate or above*	–1.15 (1.13)	[–3.50, 1.19]	–1.02 (0.319)	21.52 (1.71) vs. *22.67 (1.07)*
	High school graduate vs. *college graduate or above*	–2.40 (1.00)	[–4.48, –0.33]	–2.39 (0.025)	20.27 (1.38) vs. *22.67 (1.07)*
	Some college or AA degree vs. *college graduate or above*	0.01 (0.83)	[–1.71, 1.73]	0.01 (0.991)	22.68 (1.32) vs. *22.67 (1.07)*
Gender	Males vs. *females*	–0.69 (0.56)	[–1.86, 0.47]	–1.23 (0.231)	21.44 (1.39) vs. *22.13 (1.09)*
Race	Mexican American vs. *Non-Hispanic White*	–2.79 (1.36)	[–5.60, 0.02]	–2.06 (0.051)	20.58 (1.55) vs. *23.37 (1.22)*
	Other Hispanic vs. *Non-Hispanic White*	–1.39 (1.31)	[–4.09, 1.32]	–1.06 (0.299)	21.98 (1.55) vs. *23.37 (1.22)*
	Non–Hispanic Black vs. *Non-Hispanic White*	–1.12 (0.77)	[–2.71, 0.47]	–1.45 (0.159)	22.25 (1.24) vs. *23.37 (1.22)*
	Non–Hispanic Asian vs. *Non-Hispanic White*	–2.04 (1.18)	[–4.49, 0.40]	–1.73 (0.098)	21.33 (1.38) vs. *23.37 (1.22)*
	Other Race and Multi-Racial vs. *Non-Hispanic White*	–2.20 (1.35)	[–5.00, 0.60]	–1.62 (0.118)	21.18 (2.08) vs. *23.37 (1.22)*
Marital status	Single or never married vs. *married or living with a partner*	1.35 (1.75)	[–2.26, 4.97]	0.77 (0.447)	22.80 (2.19) vs. *21.45 (0.97)*
	Divorced or separated vs. *married or living with a partner*	0.62 (1.08)	[–1.62, 2.86]	0.58 (0.571)	22.08 (1.33) vs. *21.45 (0.97)*
	Widowed vs. *married or living with a partner*	–0.66 (1.40)	[–3.55, 2.24]	–0.47 (0.643)	20.80 (1.54) vs. *21.45 (0.97)*
Self-report general health condition	Poor vs. *excellent or very good*	4.04 (1.53)	[0.87, 7.21]	2.64 (0.015)	24.53 (1.88) vs. *20.48 (1.22)*
	Good or fair vs. *excellent or very good*	–0.14 (0.72)	[–1.64, 1.35]	–0.20 (0.843)	20.34 (0.96) vs. *20.48 (1.22)*
Difficulties in thinking or remembering	Yes vs. *no*	0.55 (0.73)	[–0.97, 2.07]	0.75 (0.462)	22.06 (1.48) vs. *21.51 (1.01)*
Ever told you have a heart disease	Yes vs. *no*	–2.17 (1.07)	[–4.38, 0.04]	–2.03 (0.054)	20.70 (1.27) vs. *22.87 (1.38)*
Ever told you had a stroke	Yes vs. *no*	1.50 (1.64)	[–1.90, 4.90]	0.91 (0.372)	22.53 (1.67) vs. *21.03 (1.23)*
Ever told you have diabetes	Yes vs. *no*	2.91 (0.83)	[1.19, 4.63]	3.49 (0.002)	23.24 (1.44) vs. *20.33 (1.11)*
Have smoked at least 100 cigarettes in life	Yes vs. *no*	0.79 (0.74)	[–0.74, 2.32]	1.07 (0.294)	22.18 (1.35) vs. *21.39 (1.18)*
Three criteria combination: AO + HDL + GLY	AO + HDL + GLY vs. *metabolically–healthy people*	–2.30 (1.18)	[–4.73, 0.13]	–1.96 (0.062)	20.63 (1.07) vs. *22.93 (1.58)*
Age at screening	Not applied (continuous covariate)	–0.19 (0.06)	[–0.31, –0.08]	–3.40 (0.002)	Not applied (continuous cov.)
Ratio of family income to the poverty level	Not applied (continuous covariate)	–0.21 (0.19)	[–0.61, 0.19]	–1.11 (0.280)	Not applied (continuous cov.)
Depression raw scores	Not applied (continuous covariate)	–0.19 (0.11)	[–0.42, 0.03]	–1.75 (0.093)	Not applied (continuous cov.)

[a]The design-based covariance matrix is singular. The validity of results is uncertain. [b]The column labeled "estimated marginal means" shows each category's marginal means against the reference category's marginal mean. The reference categories and the marginal means of the reference categories are shown in italics. The significant results have been colored.

Table S7. Multiple linear regression model explaining the relationship between CERAD-IR and specific combination AO + HBP + GLY after adjusting by sociodemographic characteristics and medical history, and estimated marginal means for each category of predictors and covariates

**Model coefficient**	**Categories (for categorical predictor and covariates)**	*Beta* (*SE*)	95% CI	*t* (*p*)	Estimated marginal means[a]
Constant	Not applied (model intercept)	38.48 (3.51)	[31.30, 45.66]	10.96 (< 0.001)	Not applied (model intercept)
Education level	Up to 12th grade vs. *college graduate or above*	–0.09 (1.04)	[–2.22, 2.04]	–0.08 (0.933)	18.26 (1.86) vs. *18.34 (1.93)*
	High school graduate vs. *college graduate or above*	–0.44 (0.54)	[–1.55, 0.66]	–0.82 (0.420)	17.90 (1.81) vs. *18.34 (1.93)*
	Some college or AA degree vs. *college graduate or above*	0.81 (0.88)	[–1.00, 2.62]	0.92 (0.365)	19.16 (1.60) vs. *18.34 (1.93)*
Gender	Males vs. *females*	–0.63 (0.73)	[–2.12, 0.86]	–0.86 (0.394)	18.10 (1.64) vs. *18.73 (1.90)*
Race	Mexican American vs. *Non-Hispanic White*	–1.38 (0.80)	[–3.01, 0.25]	–1.73 (0.095)	17.88 (1.82) vs. *19.26 (1.55)*
	Other Hispanic vs. *Non-Hispanic White*	–1.43 (0.72)	[–2.90, 0.05]	–1.98 (0.057)	17.83 (1.59) vs. *19.26 (1.55)*
	Non–Hispanic Black vs. *Non-Hispanic White*	–2.13 (0.69)	[–3.55, –0.71]	–3.07 (0.005)	17.13 (1.57) vs. *19.26 (1.55)*
	Non–Hispanic Asian vs. *Non-Hispanic White*	0.00 (1.00)	[–2.04, 2.04]	0.00 (1.000)	19.26 (1.79) vs. *19.26 (1.55)*
	Other Race and Multi-Racial vs. *Non-Hispanic White*	–0.13 (3.60)	[–7.49, 7.23]	–0.04 (0.972)	19.13 (4.04) vs. *19.26 (1.55)*
Marital status	Single or never married vs. *married or living with a partner*	2.92 (1.68)	[–0.50, 6.35]	1.74 (0.092)	20.83 (2.21) vs. *17.91 (1.65)*
	Divorced or separated vs. *married or living with a partner*	0.11 (1.27)	[–2.48, 2.70]	0.09 (0.933)	18.02 (2.06) vs. *17.91 (1.65)*
	Widowed vs. *married or living with a partner*	–1.02 (1.00)	[–3.06, 1.02]	–1.03 (0.313)	16.89 (1.96) vs. *17.91 (1.65)*
Self-report general health condition	Poor vs. *excellent or very good*	–0.11 (2.16)	[–4.54, 4.31]	–0.05 (0.959)	18.16 (2.81) vs. *18.27 (1.40)*
	Good or fair vs. *excellent or very good*	0.55 (0.71)	[–0.91, 2.00]	0.77 (0.448)	18.82 (1.51) vs. *18.27 (1.40)*
Difficulties in thinking or remembering	Yes vs. *no*	–1.35 (0.96)	[–3.31, 0.62]	–1.40 (0.172)	17.74 (1.95) vs. *19.09 (1.63)*
Ever told you have a heart disease	Yes vs. *no*	–1.52 (0.88)	[–3.32, 0.28]	–1.73 (0.094)	17.65 (1.95) vs. *19.18 (1.61)*
Ever told you had a stroke	Yes vs. *no*	–0.86 (2.08)	[–5.11, 3.39]	–0.41 (0.682)	17.98 (2.50) vs. *18.84 (1.39)*
Ever told you have diabetes	Yes vs. *no*	–0.85 (0.88)	[–2.64, 0.94]	–0.97 (0.339)	17.99 (1.84) vs. *18.84 (1.73)*
Have smoked at least 100 cigarettes in life	Yes vs. *no*	–0.65 (0.41)	[–1.49, 0.20]	–1.57 (0.127)	18.09 (1.72) vs. *18.74 (1.78)*
Three criteria combination: AO + HBP + GLY	AO + HBP + GLY vs. *metabolically–healthy people*	–1.13 (0.59)	[–2.34, 0.07]	–1.93 (0.063)	17.85 (1.69) vs. *18.98 (1.82)*
Age at screening	Not applied (continuous covariate)	–0.23 (0.05)	[–0.33, –0.12]	–4.48 (< 0.001)	Not applied (continuous cov.)
Ratio of family income to the poverty level	Not applied (continuous covariate)	–0.39 (0.15)	[–0.69, –0.08]	–2.60 (0.015)	Not applied (continuous cov.)
Depression raw scores	Not applied (continuous covariate)	0.05 (0.07)	[–0.09, 0.19]	0.75 (0.459)	Not applied (continuous cov.)

[a]The column labeled "estimated marginal means" shows each category's marginal means against the reference category's marginal mean. The reference categories and the marginal means of the reference categories are shown in italics. The significant results have been colored.

Table S8. Multiple linear regression model explaining the relationship between CERAD-IR and specific combination TRI + HDL + HBP after adjusting by sociodemographic characteristics and medical history, and estimated marginal means for each category of predictors and covariates[a]

**Model coefficient**	**Categories (for categorical predictor and covariates)**	*Beta* (*SE*)	95% CI	*t* (*p*)	Estimated marginal means[b]
Constant	Not applied (model intercept)	52.07 (3.95)	[43.47, 60.68]	13.18 (< 0.001)	Not applied (model intercept)
Education level	Up to 12th grade vs. *college graduate or above*	0.25 (1.13)	[–2.21, 2.72]	0.22 (0.826)	18.86 (1.19) vs. *18.61 (1.23)*
	High school graduate vs. *college graduate or above*	–3.62 (1.05)	[–5.90, –1.35]	–3.47 (0.005)	14.99 (1.25) vs. *18.61 (1.23)*
	Some college or AA degree vs. *college graduate or above*	0.60 (1.09)	[–1.78, 2.99]	0.55 (0.591)	19.21 (1.35) vs. *18.61 (1.23)*
Gender	Males vs. *females*	–0.57 (0.49)	[–1.63, 0.50]	–1.16 (0.270)	17.64 (1.00) vs. *18.20 (1.16)*
Race	Mexican American vs. *Non-Hispanic White*	–3.96 (1.18)	[–6.53, –1.38]	–3.35 (0.006)	15.92 (1.74) vs. *19.88 (1.14)*
	Other Hispanic vs. *Non-Hispanic White*	–0.94 (2.57)	[–6.55, 4.66]	–0.37 (0.720)	18.93 (2.18) vs. *19.88 (1.14)*
	Non–Hispanic Black vs. *Non-Hispanic White*	–4.39 (1.31)	[–7.25, –1.53]	–3.34 (0.006)	15.49 (1.41) vs. *19.88 (1.14)*
	Non–Hispanic Asian vs. *Non-Hispanic White*	–0.49 (1.69)	[–4.18, 3.20]	–0.29 (0.777)	19.39 (1.65) vs. *19.88 (1.14)*
	Other Race and Multi-Racial vs. *Non-Hispanic White*	No cases	No cases	No cases	No cases
Marital status	Single or never married vs. *married or living with a partner*	1.20 (1.84)	[–2.81, 5.20]	0.65 (0.528)	17.68 (1.97) vs. *16.48 (1.14)*
	Divorced or separated vs. *married or living with a partner*	2.44 (1.01)	[0.23, 4.65]	2.40 (0.033)	18.92 (1.35) vs. *16.48 (1.14)*
	Widowed vs. *married or living with a partner*	2.11 (0.84)	[0.28, 3.93]	2.51 (0.027)	18.59 (1.39) vs. *16.48 (1.14)*
Self-report general health condition	Poor vs. *excellent or very good*	No cases	No cases	No cases	No cases
	Good or fair vs. *excellent or very good*	–0.90 (0.61)	[–2.23, 0.43]	–1.47 (0.167)	17.47 (1.00) vs. *18.37 (1.19)*
Difficulties in thinking or remembering	Yes vs. *no*	–0.27 (1.05)	[–2.56, 2.02]	–0.26 (0.801)	17.78 (1.28) vs. *18.05 (1.07)*
Ever told you have a heart disease	Yes vs. *no*	–1.86 (0.81)	[–3.64, –0.09]	–2.29 (0.041)	16.99 (1.37) vs. *18.85 (0.83)*
Ever told you had a stroke	Yes vs. *no*	2.09 (1.15)	[–0.41, 4.59]	1.82 (0.094)	18.96 (1.15) vs. *16.88 (1.26)*
Ever told you have diabetes	Yes vs. *no*	–1.20 (1.43)	[–4.33, 1.92]	–0.84 (0.418)	17.32 (1.21) vs. *18.52 (1.34)*
Have smoked at least 100 cigarettes in life	Yes vs. *no*	1.46 (0.91)	[–0.52, 3.44]	1.61 (0.134)	18.65 (1.19) vs. *17.19 (1.11)*
Three criteria combination: TRI + HDL + HBP	TRI + HDL + HBP vs. *metabolically–healthy people*	–5.20 (1.73)	[–8.96, –1.43]	–3.01 (0.011)	15.32 (1.82) vs. *20.52 (0.64)*
Age at screening	Not applied (continuous covariate)	–0.43 (0.06)	[–0.55, –0.30]	–7.38 (< 0.001)	Not applied (continuous cov.)
Ratio of family income to the poverty level	Not applied (continuous covariate)	–0.61 (0.21)	[–1.07, –0.15]	–2.90 (0.013)	Not applied (continuous cov.)
Depression raw scores	Not applied (continuous covariate)	0.25 (0.11)	[0.00, 0.49]	2.19 (0.049)	Not applied (continuous cov.)

[a]The design-based covariance matrix is singular. The validity of results is uncertain. [b]The column labeled "estimated marginal means" shows each category's marginal means against the reference category's marginal mean. The reference categories and the marginal means of the reference categories are shown in italics. The significant results have been colored.

Table S9. Multiple linear regression model explaining the relationship between CERAD-IR and specific combination TRI + HDL + GLY after adjusting by sociodemographic characteristics and medical history, and estimated marginal means for each category of predictors and covariates[a]

**Model coefficient**	**Categories (for categorical predictor and covariates)**	*Beta* (*SE*)	95% CI	*t* (*p*)	Estimated marginal means[b]
Constant	Not applied (model intercept)	51.25 (3.80)	[43.05, 59.45]	13.50 (< 0.001)	Not applied (model intercept)
Education level	Up to 12th grade vs. *college graduate or above*	0.97 (0.86)	[–0.88, 2.82]	1.13 (0.279)	20.83 (1.78) vs. *19.86 (1.24)*
	High school graduate vs. *college graduate or above*	–3.58 (0.92)	[–5.57, –1.60]	–3.90 (0.002)	16.27 (0.98) vs. *19.86 (1.24)*
	Some college or AA degree vs. *college graduate or above*	0.68 (0.84)	[–1.12, 2.49]	0.82 (0.428)	20.54 (1.27) vs. *19.86 (1.24)*
Gender	Males vs. *females*	–0.49 (0.48)	[–1.53, 0.56]	–1.01 (0.333)	19.13 (1.32) vs. *19.62 (1.14)*
Race	Mexican American vs. *Non-Hispanic White*	–4.33 (1.27)	[–7.08, –1.58]	–3.40 (0.005)	17.21 (2.27) vs. *21.54 (1.54)*
	Other Hispanic vs. *Non-Hispanic White*	–2.34 (1.84)	[–6.32, 1.64]	–1.27 (0.227)	19.21 (1.63) vs. *21.54 (1.54)*
	Non–Hispanic Black vs. *Non-Hispanic White*	–3.58 (1.29)	[–6.37, –0.80]	–2.78 (0.016)	17.96 (1.54) vs. *21.54 (1.54)*
	Non–Hispanic Asian vs. *Non-Hispanic White*	–0.59 (1.53)	[–3.89, 2.71]	–0.39 (0.705)	20.95 (1.34) vs. *21.54 (1.54)*
	Other Race and Multi-Racial vs. *Non-Hispanic White*	No cases	No cases	No cases	No cases
Marital status	Single or never married vs. *married or living with a partner*	2.29 (1.56)	[–1.08, 5.66]	1.47 (0.165)	19.99 (2.27) vs. *17.70 (1.13)*
	Divorced or separated vs. *married or living with a partner*	3.10 (0.71)	[1.56, 4.63]	4.35 (0.001)	20.80 (0.86) vs. *17.70 (1.13)*
	Widowed vs. *married or living with a partner*	1.31 (0.85)	[–0.52, 3.15]	1.54 (0.147)	19.01 (1.47) vs. *17.70 (1.13)*
Self-report general health condition	Poor vs. *excellent or very good*	No cases	No cases	No cases	No cases
	Good or fair vs. *excellent or very good*	–0.87 (0.63)	[–2.22, 0.49]	–1.38 (0.191)	18.94 (1.32) vs. *19.81 (1.18)*
Difficulties in thinking or remembering	Yes vs. *no*	0.07 (0.94)	[–1.97, 2.11]	0.07 (0.944)	19.41 (1.53) vs. *19.34 (1.02)*
Ever told you have a heart disease	Yes vs. *no*	–1.18 (0.83)	[–2.97, 0.62]	–1.41 (0.181)	18.79 (1.20) vs. *19.96 (1.36)*
Ever told you had a stroke	Yes vs. *no*	2.26 (1.18)	[–0.28, 4.80]	1.93 (0.076)	20.51 (1.60) vs. *18.24 (1.04)*
Ever told you have diabetes	Yes vs. *no*	–1.47 (1.92)	[–5.61, 2.67]	–0.77 (0.456)	18.64 (1.95) vs. *20.11 (0.98)*
Have smoked at least 100 cigarettes in life	Yes vs. *no*	1.30 (0.79)	[–0.40, 3.00]	1.65 (0.123)	20.02 (1.23) vs. *18.73 (1.32)*
Three criteria combination: TRI + HDL + GLY	TRI + HDL + GLY vs. *metabolically–healthy people*	–3.07 (1.11)	[–5.46, –0.68]	–2.77 (0.016)	17.84 (1.54) vs. *20.91 (1.08)*
Age at screening	Not applied (continuous covariate)	–0.41 (0.05)	[–0.53, –0.29]	–7.46 (< 0.001)	Not applied (continuous cov.)
Ratio of family income to the poverty level	Not applied (continuous covariate)	–0.65 (0.20)	[–1.07, –0.22]	–3.31 (0.006)	Not applied (continuous cov.)
Depression raw scores	Not applied (continuous covariate)	–0.01 (0.08)	[–0.18, 0.16]	–0.12 (0.904)	Not applied (continuous cov.)

[a]The design-based covariance matrix is singular. The validity of results is uncertain. [b]The column labeled "estimated marginal means" shows each category's marginal means against the reference category's marginal mean. The reference categories and the marginal means of the reference categories are shown in italics. The significant results have been colored.

Table S10. Multiple linear regression model explaining the relationship between CERAD-IR and specific combination TRI + HBP + GLY after adjusting by sociodemographic characteristics and medical history, and estimated marginal means for each category of predictors and covariates[a]

**Model coefficient**	**Categories (for categorical predictor and covariates)**	*Beta* (*SE*)	95% CI	*t* (*p*)	Estimated marginal means[b]
Constant	Not applied (model intercept)	47.54 (4.68)	[37.51, 57.58]	10.17 (< 0.001)	Not applied (model intercept)
Education level	Up to 12th grade vs. *college graduate or above*	–0.11 (1.31)	[–2.92, 2.71]	–0.08 (0.936)	22.97 (1.90) vs. *23.08 (1.38)*
	High school graduate vs. *college graduate or above*	–3.59 (1.20)	[–6.16, –1.02]	–2.99 (0.010)	19.49 (1.55) vs. *23.08 (1.38)*
	Some college or AA degree vs. *college graduate or above*	0.01 (1.10)	[–2.35, 2.38]	0.01 (0.991)	23.09 (1.72) vs. *23.08 (1.38)*
Gender	Males vs. *females*	–0.17 (0.54)	[–1.33, 0.99]	–0.31 (0.758)	22.07 (1.55) vs. *22.24 (1.46)*
Race	Mexican American vs. *Non-Hispanic White*	–3.73 (1.19)	[–6.27, –1.18]	–3.14 (0.007)	20.64 (2.36) vs. *24.36 (1.94)*
	Other Hispanic vs. *Non-Hispanic White*	–0.96 (2.73)	[–6.81, 4.89]	–0.35 (0.730)	23.41 (2.20) vs. *24.36 (1.94)*
	Non–Hispanic Black vs. *Non-Hispanic White*	–5.89 (1.93)	[–10.03, –1.75]	–3.05 (0.009)	18.47 (1.60) vs. *24.36 (1.94)*
	Non–Hispanic Asian vs. *Non-Hispanic White*	–0.45 (1.68)	[–4.05, 3.15]	–0.27 (0.791)	23.91 (1.95) vs. *24.36 (1.94)*
	Other Race and Multi-Racial vs. *Non-Hispanic White*	No cases	No cases	No cases	No cases
Marital status	Single or never married vs. *married or living with a partner*	0.97 (1.78)	[–2.85, 4.80]	0.54 (0.595)	21.97 (2.75) vs. *20.99 (1.34)*
	Divorced or separated vs. *married or living with a partner*	1.84 (1.17)	[–0.68, 4.36]	1.56 (0.140)	22.83 (1.59) vs. *20.99 (1.34)*
	Widowed vs. *married or living with a partner*	1.85 (1.14)	[–0.59, 4.28]	1.62 (0.127)	22.84 (1.48) vs. *20.99 (1.34)*
Self-report general health condition	Poor vs. *excellent or very good*	No cases	No cases	No cases	No cases
	Good or fair vs. *excellent or very good*	–0.59 (0.71)	[–2.12, 0.93]	–0.83 (0.418)	21.86 (1.51) vs. *22.46 (1.54)*
Difficulties in thinking or remembering	Yes vs. *no*	–0.20 (1.01)	[–2.37, 1.96]	–0.20 (0.843)	22.06 (1.70) vs. *22.26 (1.42)*
Ever told you have a heart disease	Yes vs. *no*	–1.97 (0.90)	[–3.91, –0.03]	–2.18 (0.047)	21.17 (1.65) vs. *23.14 (1.44)*
Ever told you had a stroke	Yes vs. *no*	3.63 (1.73)	[–0.09, 7.34]	2.09 (0.055)	23.97 (2.07) vs. *20.35 (1.27)*
Ever told you have diabetes	Yes vs. *no*	0.50 (1.13)	[–1.93, 2.92]	0.44 (0.667)	22.41 (1.44) vs. *21.91 (1.72)*
Have smoked at least 100 cigarettes in life	Yes vs. *no*	1.36 (1.04)	[–0.88, 3.60]	1.30 (0.215)	22.84 (1.40) vs. *21.48 (1.72)*
Three criteria combination: TRI + HBP + GLY	TRI + HBP + GLY vs. *metabolically–healthy people*	1.08 (2.23)	[–3.70, 5.86]	0.48 (0.636)	22.70 (2.53) vs. *21.62 (0.68)*
Age at screening	Not applied (continuous covariate)	–0.36 (0.07)	[–0.51, –0.22]	–5.33 (< 0.001)	Not applied (continuous cov.)
Ratio of family income to the poverty level	Not applied (continuous covariate)	–0.45 (0.24)	[–0.97, 0.07]	–1.85 (0.086)	Not applied (continuous cov.)
Depression raw scores	Not applied (continuous covariate)	0.13 (0.10)	[–0.09, 0.34]	1.26 (0.227)	Not applied (continuous cov.)

[a]The design-based covariance matrix is singular. The validity of results is uncertain. [b]The column labeled "estimated marginal means" shows each category's marginal means against the reference category's marginal mean. The reference categories and the marginal means of the reference categories are shown in italics. The significant results have been colored.

Table S11. Multiple linear regression model explaining the relationship between CERAD-IR and specific combination HDL + HBP + GLY after adjusting by sociodemographic characteristics and medical history, and estimated marginal means for each category of predictors and covariates[a]

**Model coefficient**	**Categories (for categorical predictor and covariates)**	*Beta* (*SE*)	95% CI	*t* (*p*)	Estimated marginal means[b]
Constant	Not applied (model intercept)	49.87 (3.82)	[41.78, 57.96]	13.06 (< 0.001)	Not applied (model intercept)
Education level	Up to 12th grade vs. *college graduate or above*	–0.10 (1.44)	[–3.17, 2.96]	–0.07 (0.944)	21.18 (1.22) vs. *21.28 (1.34)*
	High school graduate vs. *college graduate or above*	–3.75 (1.21)	[–6.31, –1.19]	–3.10 (0.007)	17.53 (1.42) vs. *21.28 (1.34)*
	Some college or AA degree vs. *college graduate or above*	–0.09 (1.09)	[–2.40, 2.22]	–0.08 (0.938)	21.20 (1.19) vs. *21.28 (1.34)*
Gender	Males vs. *females*	–0.35 (0.60)	[–1.63, 0.92]	–0.59 (0.563)	20.12 (0.97) vs. *20.47 (1.18)*
Race	Mexican American vs. *Non-Hispanic White*	–3.46 (1.20)	[–6.00, –0.92]	–2.89 (0.011)	18.62 (2.01) vs. *22.08 (1.14)*
	Other Hispanic vs. *Non-Hispanic White*	–2.26 (2.24)	[–7.00, 2.49]	–1.01 (0.328)	19.82 (1.77) vs. *22.08 (1.14)*
	Non–Hispanic Black vs. *Non-Hispanic White*	–2.55 (1.25)	[–5.20, 0.09]	–2.05 (0.058)	19.53 (1.15) vs. *22.08 (1.14)*
	Non–Hispanic Asian vs. *Non-Hispanic White*	–0.66 (1.50)	[–3.83, 2.51]	–0.44 (0.665)	21.42 (1.43) vs. *22.08 (1.14)*
	Other Race and Multi-Racial vs. *Non-Hispanic White*	No cases	No cases	No cases	No cases
Marital status	Single or never married vs. *married or living with a partner*	0.92 (2.10)	[–3.54, 5.37]	0.44 (0.669)	20.28 (2.34) vs. *19.36 (1.04)*
	Divorced or separated vs. *married or living with a partner*	1.74 (1.04)	[–0.46, 3.94]	1.68 (0.113)	21.10 (0.97) vs. *19.36 (1.04)*
	Widowed vs. *married or living with a partner*	1.08 (1.36)	[–1.81, 3.96]	0.79 (0.441)	20.44 (1.64) vs. *19.36 (1.04)*
Self-report general health condition	Poor vs. *excellent or very good*	–1.18 (2.44)	[–6.34, 3.99]	–0.48 (0.636)	19.78 (2.04) vs. *20.96 (1.24)*
	Good or fair vs. *excellent or very good*	–0.80 (0.79)	[–2.49, 0.88]	–1.01 (0.327)	20.15 (1.57) vs. *20.96 (1.24)*
Difficulties in thinking or remembering	Yes vs. *no*	–0.03 (1.09)	[–2.34, 2.29]	–0.03 (0.980)	20.28 (1.37) vs. *20.31 (0.94)*
Ever told you have a heart disease	Yes vs. *no*	–1.04 (0.82)	[–2.77, 0.69]	–1.27 (0.222)	19.78 (1.34) vs. *20.82 (0.83)*
Ever told you had a stroke	Yes vs. *no*	0.94 (1.19)	[–1.58, 3.46]	0.79 (0.440)	20.77 (1.24) vs. *19.83 (1.14)*
Ever told you have diabetes	Yes vs. *no*	–0.84 (1.84)	[–4.75, 3.06]	–0.46 (0.654)	19.88 (1.24) vs. *20.72 (1.52)*
Have smoked at least 100 cigarettes in life	Yes vs. *no*	1.12 (0.97)	[–0.95, 3.18]	1.15 (0.268)	20.86 (1.13) vs. *19.74 (1.16)*
Three criteria combination: HDL + HBP + GLY	HDL + HBP + GLY vs. *metabolically–healthy people*	0.96 (1.15)	[–1.48, 3.40]	0.83 (0.417)	20.78 (1.53) vs. *19.82 (0.69)*
Age at screening	Not applied (continuous covariate)	–0.39 (0.06)	[–0.51, –0.27]	–6.95 (< 0.001)	Not applied (continuous cov.)
Ratio of family income to the poverty level	Not applied (continuous covariate)	–0.42 (0.22)	[–0.88, 0.04]	–1.92 (0.073)	Not applied (continuous cov.)
Depression raw scores	Not applied (continuous covariate)	0.09 (0.14)	[–0.20, 0.38]	0.65 (0.524)	Not applied (continuous cov.)

[a]The design-based covariance matrix is singular. The validity of results is uncertain. [b]The column labeled "estimated marginal means" shows each category's marginal means against the reference category's marginal mean. The reference categories and the marginal means of the reference categories are shown in italics. The significant results have been colored.

Table S12. Multiple linear regression model explaining the relationship between CERAD-IR and all combinations of three criteria after adjusting by sociodemographic characteristics and medical history, and estimated marginal means for each category of predictors and covariates

**Model coefficient**	**Categories (for categorical predictor and covariates)**	*Beta* (*SE*)	95% CI	*t* (*p*)	Estimated marginal means[a]
Constant	Not applied (model intercept)	33.95 (2.72)	[28.41, 39.49]	12.48 (< 0.001)	Not applied (model intercept)
Education level	Up to 12th grade vs. *college graduate or above*	−0.95 (0.83)	[−2.65, 0.75]	−1.14 (0.262)	18.67 (1.19) vs. *19.62 (1.22)*
	High school graduate vs. *college graduate or above*	−1.07 (0.74)	[−2.58, 0.44]	−1.44 (0.160)	18.56 (0.97) vs. *19.62 (1.22)*
	Some college or AA degree vs. *college graduate or above*	0.19 (0.84)	[−1.52, 1.90]	0.23 (0.820)	19.82 (1.04) vs. *19.62 (1.22)*
Gender	Males vs. *females*	−0.82 (0.53)	[−1.91, 0.27]	−1.53 (0.136)	18.76 (0.99) vs. *19.58 (1.12)*
Race	Mexican American vs. *Non-Hispanic White*	−1.73 (0.76)	[−3.28, −0.19]	−2.28 (0.029)	18.68 (1.14) vs. *20.41 (0.91)*
	Other Hispanic vs. *Non-Hispanic White*	−1.80 (0.92)	[−3.67, 0.06]	−1.97 (0.058)	18.61 (1.02) vs. *20.41 (0.91)*
	Non–Hispanic Black vs. *Non-Hispanic White*	−1.55 (0.66)	[−2.91, −0.20]	−2.34 (0.025)	18.86 (0.88) vs. *20.41 (0.91)*
	Non–Hispanic Asian vs. *Non-Hispanic White*	−0.31 (0.84)	[−2.03, 1.40]	−0.37 (0.711)	20.10 (1.08) vs. *20.41 (0.91)*
	Other Race and Multi-Racial vs. *Non-Hispanic White*	−2.06 (2.15)	[−6.45, 2.33]	−0.96 (0.345)	18.35 (2.49) vs. *20.41 (0.91)*
Marital status	Single or never married vs. *married or living with a partner*	2.24 (1.20)	[−0.20, 4.68]	1.87 (0.071)	21.04 (1.54) vs. *18.81 (0.89)*
	Divorced or separated vs. *married or living with a partner*	−0.15 (0.81)	[−1.79, 1.49]	−0.19 (0.853)	18.65 (1.34) vs. *18.81 (0.89)*
	Widowed vs. *married or living with a partner*	−0.64 (0.99)	[−2.65, 1.37]	−0.65 (0.522)	18.17 (1.16) vs. *18.81 (0.89)*
Self-report general health condition	Poor vs. *excellent or very good*	2.89 (1.00)	[0.85, 4.93]	2.89 (0.007)	20.83 (1.34) vs. *17.94 (1.04)*
	Good or fair vs. *excellent or very good*	0.79 (0.51)	[−0.26, 1.83]	1.53 (0.135)	18.73 (0.95) vs. *17.94 (1.04)*
Difficulties in thinking or remembering	Yes vs. *no*	−1.48 (0.57)	[−2.64, −0.32]	−2.60 (0.014)	18.43 (1.12) vs. *19.91 (1.00)*
Ever told you have a heart disease	Yes vs. *no*	−2.06 (0.97)	[−4.03, −0.09]	−2.13 (0.041)	18.14 (1.32) vs. *20.20 (0.91)*
Ever told you had a stroke	Yes vs. *no*	0.26 (1.30)	[−2.40, 2.92]	0.20 (0.843)	19.30 (1.42) vs. *19.04 (0.96)*
Ever told you have diabetes	Yes vs. *no*	−0.45 (0.98)	[−2.44, 1.54]	−0.46 (0.649)	18.94 (1.21) vs. *19.39 (1.05)*
Have smoked at least 100 cigarettes in life	Yes vs. *no*	−0.37 (0.38)	[−1.14, 0.39]	−1.00 (0.326)	18.98 (0.99) vs. *19.36 (1.09)*
All combinations of three criteria	All combinations of three criteria vs. *metabolically–healthy people*	−1.47 (0.56)	[−2.61, −0.34]	−2.65 (0.013)	18.43 (1.00) vs. *19.90 (1.12)*
Age at screening	Not applied (continuous covariate)	−0.16 (0.04)	[−0.24, −0.08]	−3.91 (< 0.001)	Not applied (continuous cov.)
Ratio of family income to the poverty level	Not applied (continuous covariate)	−0.11 (0.12)	[−0.36, 0.13]	−0.95 (0.351)	Not applied (continuous cov.)
Depression raw scores	Not applied (continuous covariate)	−0.04 (0.05)	[−0.14, 0.06]	−0.91 (0.370)	Not applied (continuous cov.)

[a]The column labeled "estimated marginal means" shows each category's marginal means against the reference category's marginal mean. The reference categories and the marginal means of the reference categories are shown in italics. The significant results have been colored.

Table S13. Multiple linear regression model explaining the relationship between CERAD-IR and specific combination AO + TRI + HDL + HBP after adjusting by sociodemographic characteristics and medical history, and estimated marginal means for each category of predictors and covariates[a]

**Model coefficient**	**Categories (for categorical predictor and covariates)**	*Beta* (*SE*)	95% CI	*t* (*p*)	Estimated marginal means[b]
Constant	Not applied (model intercept)	51.80 (4.29)	[42.54, 61.07]	12.08 (< 0.001)	Not applied (model intercept)
Education level	Up to 12th grade vs. *college graduate or above*	0.24 (1.15)	[–2.23, 2.72]	0.21 (0.836)	17.26 (1.41) vs. *17.02 (1.79)*
	High school graduate vs. *college graduate or above*	–3.44 (1.17)	[–5.96, –0.93]	–2.95 (0.011)	13.57 (1.60) vs. *17.02 (1.79)*
	Some college or AA degree vs. *college graduate or above*	0.27 (1.11)	[–2.13, 2.67]	0.24 (0.812)	17.29 (1.73) vs. *17.02 (1.79)*
Gender	Males vs. *females*	–0.78 (0.59)	[–2.05, 0.49]	–1.33 (0.205)	15.89 (1.48) vs. *16.68 (1.56)*
Race	Mexican American vs. *Non-Hispanic White*	–4.20 (1.24)	[–6.87, –1.53]	–3.39 (0.005)	14.24 (2.43) vs. *18.43 (1.53)*
	Other Hispanic vs. *Non-Hispanic White*	–0.98 (2.56)	[–6.50, 4.54]	–0.38 (0.708)	17.45 (1.94) vs. *18.43 (1.53)*
	Non–Hispanic Black vs. *Non-Hispanic White*	–3.17 (1.23)	[–5.83, –0.52]	–2.58 (0.023)	15.26 (1.75) vs. *18.43 (1.53)*
	Non–Hispanic Asian vs. *Non-Hispanic White*	–2.39 (2.10)	[–6.93, 2.14]	–1.14 (0.275)	16.04 (2.40) vs. *18.43 (1.53)*
	Other Race and Multi-Racial vs. *Non-Hispanic White*	No cases	No cases	No cases	No cases
Marital status	Single or never married vs. *married or living with a partner*	1.19 (2.28)	[–3.74, 6.11]	0.52 (0.611)	16.20 (2.83) vs. *15.01 (1.46)*
	Divorced or separated vs. *married or living with a partner*	2.49 (0.99)	[0.35, 4.62]	2.52 (0.026)	17.50 (1.32) vs. *15.01 (1.46)*
	Widowed vs. *married or living with a partner*	1.41 (1.07)	[–0.90, 3.73]	1.32 (0.210)	16.42 (1.72) vs. *15.01 (1.46)*
Self-report general health condition	Poor vs. *excellent or very good*	–7.59 (3.21)	[–14.52, –0.66]	–2.37 (0.034)	11.25 (2.70) vs. *18.84 (1.85)*
	Good or fair vs. *excellent or very good*	–0.07 (0.82)	[–1.84, 1.70]	–0.08 (0.934)	18.77 (1.39) vs. *18.84 (1.85)*
Difficulties in thinking or remembering	Yes vs. *no*	–1.24 (1.13)	[–3.69, 1.21]	–1.09 (0.295)	15.66 (1.81) vs. *16.90 (1.35)*
Ever told you have a heart disease	Yes vs. *no*	–1.13 (1.01)	[–3.32, 1.06]	–1.11 (0.286)	15.72 (1.88) vs. *16.85 (1.18)*
Ever told you had a stroke	Yes vs. *no*	–2.26 (2.93)	[–8.60, 4.07]	–0.77 (0.454)	15.15 (2.65) vs. *17.42 (1.31)*
Ever told you have diabetes	Yes vs. *no*	0.87 (2.31)	[–4.14, 5.87]	0.37 (0.715)	16.72 (1.43) vs. *15.85 (2.25)*
Have smoked at least 100 cigarettes in life	Yes vs. *no*	0.77 (0.95)	[–1.27, 2.82]	0.82 (0.428)	16.67 (1.57) vs. *15.90 (1.56)*
Four criteria combination: AO + TRI + HDL + HBP	AO + TRI + HDL + HBP vs. *metabolically–healthy people*	–0.29 (1.41)	[–3.34, 2.76]	–0.20 (0.841)	16.14 (1.75) vs. *16.43 (1.54)*
Age at screening	Not applied (continuous covariate)	–0.42 (0.06)	[–0.55, –0.28]	–6.79 (< 0.001)	Not applied (continuous cov.)
Ratio of family income to the poverty level	Not applied (continuous covariate)	–0.50 (0.23)	[–1.00, 0.01]	–2.13 (0.053)	Not applied (continuous cov.)
Depression raw scores	Not applied (continuous covariate)	0.03 (0.09)	[–0.17, 0.23]	0.31 (0.761)	Not applied (continuous cov.)

[a]The design-based covariance matrix is singular. The validity of results is uncertain. [b]The column labeled "estimated marginal means" shows each category's marginal means against the reference category's marginal mean. The reference categories and the marginal means of the reference categories are shown in italics. The significant results have been colored.

Table S14. Multiple linear regression model explaining the relationship between CERAD-IR and specific combination AO + TRI + HDL + GLY after adjusting by sociodemographic characteristics and medical history, and estimated marginal means for each category of predictors and covariates[a]

**Model coefficient**	**Categories (for categorical predictor and covariates)**	*Beta* (*SE*)	95% CI	*t* (*p*)	Estimated marginal means[b]
Constant	Not applied (model intercept)	42.90 (3.60)	[35.37, 50.43]	11.93 (< 0.001)	Not applied (model intercept)
Education level	Up to 12th grade vs. *college graduate or above*	–1.69 (1.86)	[–5.58, 2.20]	–0.91 (0.375)	17.65 (2.02) vs. *19.34 (1.20)*
	High school graduate vs. *college graduate or above*	–2.23 (1.07)	[–4.48, 0.02]	–2.08 (0.051)	17.10 (0.92) vs. *19.34 (1.20)*
	Some college or AA degree vs. *college graduate or above*	–1.23 (1.31)	[–3.98, 1.52]	–0.93 (0.362)	18.11 (0.92) vs. *19.34 (1.20)*
Gender	Males vs. *females*	–1.21 (0.77)	[–2.82, 0.40]	–1.57 (0.133)	17.44 (1.00) vs. *18.65 (1.10)*
Race	Mexican American vs. *Non-Hispanic White*	–3.38 (0.59)	[–4.61, –2.14]	–5.73 (< 0.001)	16.50 (1.42) vs. *19.88 (1.24)*
	Other Hispanic vs. *Non-Hispanic White*	–2.16 (1.73)	[–5.78, 1.46]	–1.25 (0.227)	17.72 (1.33) vs. *19.88 (1.24)*
	Non–Hispanic Black vs. *Non-Hispanic White*	–3.67 (1.61)	[–7.05, –0.30]	–2.28 (0.035)	16.20 (1.97) vs. *19.88 (1.24)*
	Non–Hispanic Asian vs. *Non-Hispanic White*	0.68 (1.33)	[–2.09, 3.46]	0.52 (0.612)	20.56 (1.39) vs. *19.88 (1.24)*
	Other Race and Multi-Racial vs. *Non-Hispanic White*	–2.45 (1.57)	[–5.74, 0.83]	–1.56 (0.134)	17.42 (1.63) vs. *19.88 (1.24)*
Marital status	Single or never married vs. *married or living with a partner*	0.35 (2.12)	[–4.09, 4.79]	0.16 (0.872)	18.91 (2.28) vs. *18.57 (1.03)*
	Divorced or separated vs. *married or living with a partner*	–1.86 (1.38)	[–4.75, 1.03]	–1.35 (0.194)	16.71 (1.27) vs. *18.57 (1.03)*
	Widowed vs. *married or living with a partner*	–0.56 (1.55)	[–3.81, 2.68]	–0.36 (0.720)	18.00 (1.49) vs. *18.57 (1.03)*
Self-report general health condition	Poor vs. *excellent or very good*	–0.77 (1.63)	[–4.18, 2.64]	–0.47 (0.643)	17.43 (1.58) vs. *18.20 (1.16)*
	Good or fair vs. *excellent or very good*	0.32 (0.96)	[–1.68, 2.33]	0.34 (0.741)	18.52 (0.83) vs. *18.20 (1.16)*
Difficulties in thinking or remembering	Yes vs. *no*	–0.95 (0.84)	[–2.72, 0.82]	–1.12 (0.275)	17.57 (1.22) vs. *18.52 (0.89)*
Ever told you have a heart disease	Yes vs. *no*	–1.31 (1.17)	[–3.76, 1.14]	–1.12 (0.277)	17.39 (1.25) vs. *18.70 (1.02)*
Ever told you had a stroke	Yes vs. *no*	1.18 (1.19)	[–1.31, 3.67]	0.99 (0.334)	18.64 (1.21) vs. *17.46 (1.07)*
Ever told you have diabetes	Yes vs. *no*	–0.09 (1.23)	[–2.66, 2.47]	–0.08 (0.940)	18.00 (1.24) vs. *18.09 (1.06)*
Have smoked at least 100 cigarettes in life	Yes vs. *no*	–0.01 (0.84)	[–1.78, 1.76]	–0.01 (0.989)	18.04 (1.02) vs. *18.05 (1.11)*
Four criteria combination: AO + TRI + HDL + GLY	AO + TRI + HDL + GLY vs. *metabolically–healthy people*	–1.85 (0.79)	[–3.51, –0.20]	–2.34 (0.030)	17.12 (1.07) vs. *18.97 (1.04)*
Age at screening	Not applied (continuous covariate)	–0.30 (0.05)	[–0.39, –0.20]	–6.35 (< 0.001)	Not applied (continuous cov.)
Ratio of family income to the poverty level	Not applied (continuous covariate)	0.24 (0.22)	[–0.21, 0.70]	1.12 (0.278)	Not applied (continuous cov.)
Depression raw scores	Not applied (continuous covariate)	0.17 (0.13)	[–0.10, 0.43]	1.31 (0.205)	Not applied (continuous cov.)

[a]The design-based covariance matrix is singular. The validity of results is uncertain. [b]The column labeled "estimated marginal means" shows each category's marginal means against the reference category's marginal mean. The reference categories and the marginal means of the reference categories are shown in italics. The significant results have been colored.

Table S15. Multiple linear regression model explaining the relationship between CERAD-IR and specific combination AO + TRI + HBP + GLY after adjusting by sociodemographic characteristics and medical history, and estimated marginal means for each category of predictors and covariates[a]

**Model coefficient**	**Categories (for categorical predictor and covariates)**	*Beta* (*SE*)	95% CI	*t* (*p*)	Estimated marginal means[b]
Constant	Not applied (model intercept)	46.15 (4.09)	[37.72, 54.59]	11.29 (< 0.001)	Not applied (model intercept)
Education level	Up to 12th grade vs. *college graduate or above*	–1.12 (1.02)	[–3.23, 0.98]	–1.10 (0.282)	21.25 (1.01) vs. *22.37 (0.80)*
	High school graduate vs. *college graduate or above*	–2.16 (0.84)	[–3.91, –0.42]	–2.56 (0.017)	20.21 (0.91) vs. *22.37 (0.80)*
	Some college or AA degree vs. *college graduate or above*	–0.23 (0.80)	[–1.87, 1.42]	–0.28 (0.780)	22.15 (0.98) vs. *22.37 (0.80)*
Gender	Males vs. *females*	–1.94 (0.57)	[–3.11, –0.76]	–3.40 (0.002)	20.53 (0.88) vs. *22.46 (0.70)*
Race	Mexican American vs. *Non-Hispanic White*	–2.98 (0.93)	[–4.89, –1.06]	–3.20 (0.004)	20.00 (1.26) vs. *22.98 (0.77)*
	Other Hispanic vs. *Non-Hispanic White*	–4.18 (0.94)	[–6.12, –2.24]	–4.44 (< 0.001)	18.80 (0.90) vs. *22.98 (0.77)*
	Non–Hispanic Black vs. *Non-Hispanic White*	–0.31 (0.80)	[–1.96, 1.33]	–0.39 (0.697)	22.67 (1.15) vs. *22.98 (0.77)*
	Non–Hispanic Asian vs. *Non-Hispanic White*	0.04 (1.07)	[–2.17, 2.26]	0.04 (0.967)	23.03 (1.10) vs. *22.98 (0.77)*
	Other Race and Multi-Racial vs. *Non-Hispanic White*	No cases	No cases	No cases	No cases
Marital status	Single or never married vs. *married or living with a partner*	1.16 (1.74)	[–2.42, 4.75]	0.67 (0.510)	21.56 (1.75) vs. *20.40 (0.95)*
	Divorced or separated vs. *married or living with a partner*	2.48 (1.14)	[0.12, 4.84]	2.16 (0.041)	22.88 (1.35) vs. *20.40 (0.95)*
	Widowed vs. *married or living with a partner*	0.75 (1.11)	[–1.55, 3.05]	0.67 (0.508)	21.15 (0.79) vs. *20.40 (0.95)*
Self-report general health condition	Poor vs. *excellent or very good*	0.37 (1.95)	[–3.64, 4.39]	0.19 (0.850)	21.86 (1.59) vs. *21.48 (1.04)*
	Good or fair vs. *excellent or very good*	–0.34 (0.60)	[–1.58, 0.91]	–0.56 (0.584)	21.15 (0.87) vs. *21.48 (1.04)*
Difficulties in thinking or remembering	Yes vs. *no*	0.25 (1.45)	[–2.76, 3.25]	0.17 (0.868)	21.62 (1.10) vs. *21.37 (0.97)*
Ever told you have a heart disease	Yes vs. *no*	1.39 (0.88)	[–0.43, 3.21]	1.58 (0.128)	22.19 (0.92) vs. *20.80 (0.80)*
Ever told you had a stroke	Yes vs. *no*	2.05 (1.30)	[–0.64, 4.74]	1.57 (0.130)	22.52 (1.14) vs. *20.47 (0.81)*
Ever told you have diabetes	Yes vs. *no*	–1.32 (0.88)	[–3.13, 0.50]	–1.50 (0.147)	20.84 (1.00) vs. *22.15 (0.70)*
Have smoked at least 100 cigarettes in life	Yes vs. *no*	–0.26 (0.86)	[–2.04, 1.53]	–0.30 (0.769)	21.37 (0.83) vs. *21.62 (0.89)*
Four criteria combination: AO + TRI + HBP + GLY	AO + TRI + HBP + GLY vs. *metabolically–healthy people*	0.34 (0.77)	[–1.25, 1.93]	0.44 (0.666)	21.66 (0.78) vs. *21.33 (0.90)*
Age at screening	Not applied (continuous covariate)	–0.33 (0.06)	[–0.46, –0.20]	–5.21 (< 0.001)	Not applied (continuous cov.)
Ratio of family income to the poverty level	Not applied (continuous covariate)	–0.31 (0.19)	[–0.71, 0.08]	–1.62 (0.117)	Not applied (continuous cov.)
Depression raw scores	Not applied (continuous covariate)	–0.10 (0.03)	[–0.17, –0.04]	–3.31 (0.003)	Not applied (continuous cov.)

[a]The design-based covariance matrix is singular. The validity of results is uncertain. [b]The column labeled "estimated marginal means" shows each category's marginal means against the reference category's marginal mean. The reference categories and the marginal means of the reference categories are shown in italics. The significant results have been colored.

Table S16. Multiple linear regression model explaining the relationship between CERAD-IR and specific combination AO + HDL + HBP + GLY after adjusting by sociodemographic characteristics and medical history, and estimated marginal means for each category of predictors and covariates[a]

**Model coefficient**	**Categories (for categorical predictor and covariates)**	*Beta* (*SE*)	95% CI	*t* (*p*)	Estimated marginal means[b]
Constant	Not applied (model intercept)	47.87 (3.43)	[40.66, 55.08]	13.95 (< 0.001)	Not applied (model intercept)
Education level	Up to 12th grade vs. *college graduate or above*	–0.97 (1.14)	[–3.36, 1.43]	–0.85 (0.408)	21.10 (2.68) vs. *22.07 (2.19)*
	High school graduate vs. *college graduate or above*	–2.77 (0.92)	[–4.71, –0.83]	–3.00 (0.008)	19.30 (2.18) vs. *22.07 (2.19)*
	Some college or AA degree vs. *college graduate or above*	0.31 (0.85)	[–1.48, 2.10]	0.36 (0.720)	22.38 (2.25) vs. *22.07 (2.19)*
Gender	Males vs. *females*	–1.10 (0.47)	[–2.08, –0.12]	–2.35 (0.031)	20.66 (2.31) vs. *21.76 (2.18)*
Race	Mexican American vs. *Non-Hispanic White*	–4.97 (1.47)	[–8.04, –1.89]	–3.39 (0.003)	18.14 (3.05) vs. *23.11 (2.25)*
	Other Hispanic vs. *Non-Hispanic White*	–2.07 (1.20)	[–4.59, 0.45]	–1.73 (0.102)	21.04 (2.15) vs. *23.11 (2.25)*
	Non–Hispanic Black vs. *Non-Hispanic White*	–1.81 (1.05)	[–4.01, 0.38]	–1.73 (0.100)	21.30 (2.06) vs. *23.11 (2.25)*
	Non–Hispanic Asian vs. *Non-Hispanic White*	0.00 (1.82)	[–3.83, 3.83]	0.00 (0.999)	23.11 (3.10) vs. *23.11 (2.25)*
	Other Race and Multi-Racial vs. *Non-Hispanic White*	–2.54 (1.63)	[–5.97, 0.88]	–1.56 (0.136)	20.57 (2.22) vs. *23.11 (2.25)*
Marital status	Single or never married vs. *married or living with a partner*	–0.02 (1.21)	[–2.55, 2.52]	–0.01 (0.990)	20.00 (2.94) vs. *20.02 (1.98)*
	Divorced or separated vs. *married or living with a partner*	3.36 (0.63)	[2.05, 4.67]	5.37 (< 0.001)	23.38 (2.10) vs. *20.02 (1.98)*
	Widowed vs. *married or living with a partner*	1.43 (1.17)	[–1.03, 3.88]	1.22 (0.238)	21.44 (2.29) vs. *20.02 (1.98)*
Self-report general health condition	Poor vs. *excellent or very good*	–0.33 (2.68)	[–5.97, 5.31]	–0.12 (0.903)	21.21 (3.11) vs. *21.54 (2.33)*
	Good or fair vs. *excellent or very good*	–0.66 (0.67)	[–2.05, 0.74]	–0.99 (0.336)	20.88 (2.05) vs. *21.54 (2.33)*
Difficulties in thinking or remembering	Yes vs. *no*	–0.57 (1.04)	[–2.76, 1.63]	–0.55 (0.592)	20.93 (2.49) vs. *21.50 (2.09)*
Ever told you have a heart disease	Yes vs. *no*	–0.98 (0.74)	[–2.53, 0.57]	–1.33 (0.201)	20.72 (2.36) vs. *21.70 (2.16)*
Ever told you had a stroke	Yes vs. *no*	4.94 (3.29)	[–1.98, 11.85]	1.50 (0.151)	23.68 (3.78) vs. *18.74 (1.05)*
Ever told you have diabetes	Yes vs. *no*	–1.69 (1.18)	[–4.16, 0.78]	–1.44 (0.168)	20.37 (2.32) vs. *22.05 (2.30)*
Have smoked at least 100 cigarettes in life	Yes vs. *no*	0.46 (0.66)	[–0.93, 1.85]	0.69 (0.496)	21.44 (2.25) vs. *20.98 (2.27)*
Four criteria combination: AO + HDL + HBP + GLY	AO + HDL + HBP + GLY vs. *metabolically–healthy people*	0.13 (0.92)	[–1.81, 2.06]	0.14 (0.892)	21.27 (2.21) vs. *21.15 (2.35)*
Age at screening	Not applied (continuous covariate)	–0.36 (0.05)	[–0.46, –0.26]	–7.33 (< 0.001)	Not applied (continuous cov.)
Ratio of family income to the poverty level	Not applied (continuous covariate)	–0.42 (0.20)	[–0.83, –0.01]	–2.13 (0.047)	Not applied (continuous cov.)
Depression raw scores	Not applied (continuous covariate)	0.04 (0.06)	[–0.09, 0.18]	0.66 (0.515)	Not applied (continuous cov.)

[a]The design-based covariance matrix is singular. The validity of results is uncertain. [b]The column labeled "estimated marginal means" shows each category's marginal means against the reference category's marginal mean. The reference categories and the marginal means of the reference categories are shown in italics. The significant results have been colored.

Table S17. Multiple linear regression model explaining the relationship between CERAD-IR and specific combination TRI + HDL + HBP + GLY after adjusting by sociodemographic characteristics and medical history, and estimated marginal means for each category of predictors and covariates[a]

**Model coefficient**	**Categories (for categorical predictor and covariates)**	*Beta* (*SE*)	95% CI	*t* (*p*)	Estimated marginal means[b]
Constant	Not applied (model intercept)	51.97 (4.03)	[43.26, 60.68]	12.89 (< 0.001)	Not applied (model intercept)
Education level	Up to 12th grade vs. *college graduate or above*	0.32 (1.22)	[−2.31, 2.96]	0.27 (0.794)	21.03 (1.24) vs. *20.71 (0.85)*
	High school graduate vs. *college graduate or above*	−3.77 (1.11)	[−6.17, −1.36]	−3.38 (0.005)	16.94 (0.98) vs. *20.71 (0.85)*
	Some college or AA degree vs. *college graduate or above*	0.23 (1.07)	[−2.09, 2.55]	0.21 (0.836)	20.93 (1.25) vs. *20.71 (0.85)*
Gender	Males vs. *females*	−0.42 (0.48)	[−1.46, 0.62]	−0.87 (0.399)	19.69 (0.97) vs. *20.11 (0.80)*
Race	Mexican American vs. *Non-Hispanic White*	−3.87 (1.21)	[−6.49, −1.25]	−3.19 (0.007)	18.19 (1.68) vs. *22.06 (1.39)*
	Other Hispanic vs. *Non-Hispanic White*	−0.84 (2.86)	[−7.01, 5.33]	−0.29 (0.773)	21.22 (2.43) vs. *22.06 (1.39)*
	Non–Hispanic Black vs. *Non-Hispanic White*	−3.76 (1.36)	[−6.68, −0.83]	−2.77 (0.016)	18.31 (0.99) vs. *22.06 (1.39)*
	Non–Hispanic Asian vs. *Non-Hispanic White*	−0.90 (1.91)	[−5.03, 3.24]	−0.47 (0.647)	21.17 (2.18) vs. *22.06 (1.39)*
	Other Race and Multi-Racial vs. *Non-Hispanic White*	−3.61 (2.86)	[−9.78, 2.56]	−1.26 (0.229)	18.46 (2.27) vs. *22.06 (1.39)*
Marital status	Single or never married vs. *married or living with a partner*	1.31 (1.97)	[−2.94, 5.56]	0.66 (0.518)	19.87 (2.18) vs. *18.56 (0.77)*
	Divorced or separated vs. *married or living with a partner*	2.31 (1.02)	[0.11, 4.51]	2.27 (0.041)	20.87 (1.04) vs. *18.56 (0.77)*
	Widowed vs. *married or living with a partner*	1.73 (0.96)	[−0.35, 3.82]	1.80 (0.095)	20.30 (1.27) vs. *18.56 (0.77)*
Self-report general health condition	Poor vs. *excellent or very good*	−1.76 (2.54)	[−7.24, 3.73]	−0.69 (0.502)	19.04 (2.13) vs. *20.79 (1.07)*
	Good or fair vs. *excellent or very good*	−0.92 (0.66)	[−2.34, 0.50]	−1.40 (0.186)	19.88 (1.29) vs. *20.79 (1.07)*
Difficulties in thinking or remembering	Yes vs. *no*	0.05 (1.03)	[−2.18, 2.27]	0.05 (0.965)	19.93 (1.01) vs. *19.88 (0.99)*
Ever told you have a heart disease	Yes vs. *no*	−1.32 (0.81)	[−3.08, 0.44]	−1.62 (0.129)	19.24 (1.02) vs. *20.56 (0.87)*
Ever told you had a stroke	Yes vs. *no*	1.93 (1.23)	[−0.73, 4.59]	1.57 (0.141)	20.87 (1.33) vs. *18.94 (0.67)*
Ever told you have diabetes	Yes vs. *no*	0.48 (1.62)	[−3.02, 3.98]	0.30 (0.772)	20.14 (0.80) vs. *19.66 (1.46)*
Have smoked at least 100 cigarettes in life	Yes vs. *no*	1.46 (0.98)	[−0.65, 3.56]	1.49 (0.160)	20.63 (0.81) vs. *19.17 (1.13)*
Four criteria combination: TRI + HDL + HBP + GLY	TRI + HDL + HBP + GLY vs. *metabolically–healthy people*	−1.73 (2.80)	[−7.79, 4.32]	−0.62 (0.547)	19.04 (2.03) vs. *20.77 (1.13)*
Age at screening	Not applied (continuous covariate)	−0.42 (0.06)	[−0.55, −0.30]	−7.20 (< 0.001)	Not applied (continuous cov.)
Ratio of family income to the poverty level	Not applied (continuous covariate)	−0.55 (0.24)	[−1.07, −0.04]	−2.32 (0.037)	Not applied (continuous cov.)
Depression raw scores	Not applied (continuous covariate)	0.12 (0.12)	[−0.13, 0.38]	1.05 (0.311)	Not applied (continuous cov.)

[a]The design-based covariance matrix is singular. The validity of results is uncertain. [b]The column labeled "estimated marginal means" shows each category's marginal means against the reference category's marginal mean. The reference categories and the marginal means of the reference categories are shown in italics. The significant results have been colored.

Table S18. Multiple linear regression model explaining the relationship between CERAD-IR and all combinations of four criteria after adjusting by sociodemographic characteristics and medical history, and estimated marginal means for each category of predictors and covariates

**Model coefficient**	**Categories (for categorical predictor and covariates)**	*Beta* (*SE*)	95% CI	*t* (*p*)	Estimated marginal means[a]
Constant	Not applied (model intercept)	42.11 (3.39)	[35.15, 49.07]	12.42 (< 0.001)	Not applied (model intercept)
Education level	Up to 12th grade vs. *college graduate or above*	–1.81 (1.10)	[–4.07, 0.46]	–1.64 (0.113)	18.12 (1.40) vs. *19.93 (0.87)*
	High school graduate vs. *college graduate or above*	–1.54 (0.56)	[–2.69, –0.38]	–2.74 (0.011)	18.39 (0.95) vs. *19.93 (0.87)*
	Some college or AA degree vs. *college graduate or above*	–0.32 (0.90)	[–2.15, 1.52]	–0.35 (0.727)	19.61 (1.19) vs. *19.93 (0.87)*
Gender	Males vs. *females*	–1.67 (0.67)	[–3.05, –0.29]	–2.48 (0.020)	18.18 (1.09) vs. *19.85 (0.95)*
Race	Mexican American vs. *Non-Hispanic White*	–3.46 (0.63)	[–4.76, –2.16]	–5.45 (< 0.001)	17.25 (1.08) vs. *20.71 (1.05)*
	Other Hispanic vs. *Non-Hispanic White*	–3.73 (1.06)	[–5.90, –1.56]	–3.53 (0.002)	16.97 (1.26) vs. *20.71 (1.05)*
	Non–Hispanic Black vs. *Non-Hispanic White*	–0.38 (0.55)	[–1.50, 0.75]	–0.69 (0.498)	20.33 (0.94) vs. *20.71 (1.05)*
	Non–Hispanic Asian vs. *Non-Hispanic White*	0.78 (1.31)	[–1.90, 3.46]	0.60 (0.555)	21.49 (1.63) vs. *20.71 (1.05)*
	Other Race and Multi-Racial vs. *Non-Hispanic White*	–3.37 (1.06)	[–5.54, –1.20]	–3.19 (0.004)	17.33 (1.31) vs. *20.71 (1.05)*
Marital status	Single or never married vs. *married or living with a partner*	–0.39 (1.28)	[–3.02, 2.24]	–0.31 (0.763)	18.53 (1.60) vs. *18.92 (0.74)*
	Divorced or separated vs. *married or living with a partner*	0.21 (0.82)	[–1.47, 1.89]	0.26 (0.799)	19.13 (1.24) vs. *18.92 (0.74)*
	Widowed vs. *married or living with a partner*	0.56 (0.78)	[–1.04, 2.16]	0.72 (0.480)	19.48 (0.91) vs. *18.92 (0.74)*
Self-report general health condition	Poor vs. *excellent or very good*	2.17 (1.13)	[–0.16, 4.50]	1.91 (0.067)	20.30 (1.27) vs. *18.13 (1.20)*
	Good or fair vs. *excellent or very good*	0.47 (0.82)	[–1.20, 2.14]	0.57 (0.571)	18.60 (0.80) vs. *18.13 (1.20)*
Difficulties in thinking or remembering	Yes vs. *no*	–2.15 (0.77)	[–3.73, –0.56]	–2.78 (0.010)	17.94 (1.20) vs. *20.09 (0.84)*
Ever told you have a heart disease	Yes vs. *no*	–0.22 (0.99)	[–2.25, 1.82]	–0.22 (0.829)	18.90 (1.20) vs. *19.12 (0.96)*
Ever told you had a stroke	Yes vs. *no*	1.64 (1.16)	[–0.75, 4.03]	1.41 (0.169)	19.83 (1.37) vs. *18.19 (0.82)*
Ever told you have diabetes	Yes vs. *no*	–0.71 (0.65)	[–2.04, 0.62]	–1.10 (0.282)	18.66 (0.96) vs. *19.37 (1.07)*
Have smoked at least 100 cigarettes in life	Yes vs. *no*	–0.44 (0.68)	[–1.83, 0.96]	–0.64 (0.525)	18.79 (1.04) vs. *19.23 (1.01)*
All combinations of four criteria	All combinations of four criteria vs. *metabolically–healthy people*	–1.13 (0.66)	[–2.47, 0.21]	–1.73 (0.096)	18.45 (0.96) vs. *19.58 (1.07)*
Age at screening	Not applied (continuous covariate)	–0.28 (0.05)	[–0.37, –0.18]	–5.97 (< 0.001)	Not applied (continuous cov.)
Ratio of family income to the poverty level	Not applied (continuous covariate)	0.04 (0.19)	[–0.35, 0.44]	0.23 (0.819)	Not applied (continuous cov.)
Depression raw scores	Not applied (continuous covariate)	–0.02 (0.07)	[–0.18, 0.13]	–0.31 (0.760)	Not applied (continuous cov.)

[a]The column labeled "estimated marginal means" shows each category's marginal means against the reference category's marginal mean. The reference categories and the marginal means of the reference categories are shown in italics. The significant results have been colored.

Table S19. Multiple linear regression model explaining the relationship between CERAD-IR and combination of five criteria after adjusting by sociodemographic characteristics and medical history, and estimated marginal means for each category of predictors and covariates[a]

**Model coefficient**	**Categories (for categorical predictor and covariates)**	*Beta* (*SE*)	95% CI	*t* (*p*)	Estimated marginal means[b]
Constant	Not applied (model intercept)	40.70 (5.01)	[30.26, 51.15]	8.13 (< 0.001)	Not applied (model intercept)
Education level	Up to 12th grade vs. *college graduate or above*	–0.70 (1.70)	[–4.26, 2.85]	–0.41 (0.685)	18.82 (1.54) vs. *19.52 (1.68)*
	High school graduate vs. *college graduate or above*	–3.63 (1.00)	[–5.71, –1.55]	–3.65 (0.002)	15.89 (1.55) vs. *19.52 (1.68)*
	Some college or AA degree vs. *college graduate or above*	–1.15 (0.81)	[–2.84, 0.55]	–1.41 (0.173)	18.38 (1.53) vs. *19.52 (1.68)*
Gender	Males vs. *females*	–1.13 (0.59)	[–2.36, 0.11]	–1.90 (0.072)	17.59 (1.28) vs. *18.72 (1.44)*
Race	Mexican American vs. *Non-Hispanic White*	–0.84 (0.94)	[–2.80, 1.11]	–0.90 (0.378)	18.63 (1.36) vs. *19.47 (0.88)*
	Other Hispanic vs. *Non-Hispanic White*	–3.62 (1.59)	[–6.93, –0.31]	–2.28 (0.033)	15.85 (1.89) vs. *19.47 (0.88)*
	Non–Hispanic Black vs. *Non-Hispanic White*	–1.51 (1.91)	[–5.49, 2.46]	–0.79 (0.436)	17.96 (2.25) vs. *19.47 (0.88)*
	Non–Hispanic Asian vs. *Non-Hispanic White*	–0.62 (1.08)	[–2.87, 1.62]	–0.58 (0.568)	18.85 (1.63) vs. *19.47 (0.88)*
	Other Race and Multi-Racial vs. *Non-Hispanic White*	No cases	No cases	No cases	No cases
Marital status	Single or never married vs. *married or living with a partner*	0.52 (2.01)	[–3.68, 4.72]	0.26 (0.800)	18.93 (1.71) vs. *18.42 (1.62)*
	Divorced or separated vs. *married or living with a partner*	1.23 (0.96)	[–0.78, 3.23]	1.28 (0.217)	19.64 (1.73) vs. *18.42 (1.62)*
	Widowed vs. *married or living with a partner*	–2.80 (0.84)	[–4.56, –1.05]	–3.34 (0.003)	15.61 (1.64) vs. *18.42 (1.62)*
Self-report general health condition	Poor vs. *excellent or very good*	2.43 (1.98)	[–1.70, 6.56]	1.23 (0.233)	19.86 (2.51) vs. *17.42 (1.08)*
	Good or fair vs. *excellent or very good*	–0.24 (0.77)	[–1.86, 1.37]	–0.32 (0.756)	17.18 (0.88) vs. *17.42 (1.08)*
Difficulties in thinking or remembering	Yes vs. *no*	1.07 (1.31)	[–1.65, 3.79]	0.82 (0.422)	18.69 (1.28) vs. *17.62 (1.66)*
Ever told you have a heart disease	Yes vs. *no*	0.04 (0.62)	[–1.26, 1.34]	0.07 (0.946)	18.17 (1.55) vs. *18.13 (1.16)*
Ever told you had a stroke	Yes vs. *no*	–4.79 (1.35)	[–7.60, –1.99]	–3.56 (0.002)	15.75 (1.88) vs. *20.55 (0.96)*
Ever told you have diabetes	Yes vs. *no*	–1.32 (1.46)	[–4.37, 1.74]	–0.90 (0.379)	17.49 (1.82) vs. *18.81 (1.13)*
Have smoked at least 100 cigarettes in life	Yes vs. *no*	0.39 (0.89)	[–1.47, 2.25]	0.44 (0.666)	18.35 (1.57) vs. *17.96 (1.21)*
Combination of five criteria	AO + TRI + HDL + HBP + GLY vs. *metabolically–healthy people*	–0.19 (0.74)	[–1.74, 1.36]	–0.26 (0.800)	18.06 (1.14) vs. *18.25 (1.59)*
Age at screening	Not applied (continuous covariate)	–0.24 (0.08)	[–0.40, –0.08]	–3.17 (0.005)	Not applied (continuous cov.)
Ratio of family income to the poverty level	Not applied (continuous covariate)	–0.36 (0.20)	[–0.77, 0.05]	–1.81 (0.086)	Not applied (continuous cov.)
Depression raw scores	Not applied (continuous covariate)	–0.01 (0.05)	[–0.11, 0.08]	–0.30 (0.766)	Not applied (continuous cov.)

[a]The design-based covariance matrix is singular. The validity of results is uncertain. [b]The column labeled "estimated marginal means" shows each category's marginal means against the reference category's marginal mean. The reference categories and the marginal means of the reference categories are shown in italics. The significant results have been colored.

Table S20. Multiple linear regression model explaining the relationship between CERAD-IR and all combination with abdominal obesity after adjusting by sociodemographic characteristics and medical history, and estimated marginal means for each category of predictors and covariates

**Model coefficient**	**Categories (for categorical predictor and covariates)**	*Beta* (*SE*)	95% CI	*t* (*p*)	Estimated marginal means[a]
Constant	Not applied (model intercept)	35.09 (2.52)	[29.96, 40.23]	13.93 (< 0.001)	Not applied (model intercept)
Education level	Up to 12th grade vs. *college graduate or above*	–1.53 (0.79)	[–3.14, 0.08]	–1.94 (0.062)	18.86 (0.87) vs. *20.39 (0.82)*
	High school graduate vs. *college graduate or above*	–1.00 (0.61)	[–2.25, 0.24]	–1.64 (0.111)	19.38 (0.53) vs. *20.39 (0.82)*
	Some college or AA degree vs. *college graduate or above*	–0.04 (0.61)	[–1.28, 1.20]	–0.06 (0.952)	20.35 (0.79) vs. *20.39 (0.82)*
Gender	Males vs. *females*	–1.20 (0.36)	[–1.94, –0.46]	–3.29 (0.002)	19.15 (0.65) vs. *20.34 (0.72)*
Race	Mexican American vs. *Non-Hispanic White*	–1.78 (0.64)	[–3.09, –0.47]	–2.77 (0.009)	19.05 (0.90) vs. *20.83 (0.73)*
	Other Hispanic vs. *Non-Hispanic White*	–2.38 (0.70)	[–3.81, –0.94]	–3.38 (0.002)	18.45 (0.77) vs. *20.83 (0.73)*
	Non–Hispanic Black vs. *Non-Hispanic White*	–0.95 (0.48)	[–1.93, 0.03]	–1.97 (0.058)	19.88 (0.71) vs. *20.83 (0.73)*
	Non–Hispanic Asian vs. *Non-Hispanic White*	0.67 (0.94)	[–1.25, 2.58]	0.71 (0.482)	21.50 (0.91) vs. *20.83 (0.73)*
	Other Race and Multi-Racial vs. *Non-Hispanic White*	–2.07 (1.09)	[–4.29, 0.15]	–1.90 (0.066)	18.76 (1.16) vs. *20.83 (0.73)*
Marital status	Single or never married vs. *married or living with a partner*	1.61 (0.98)	[–0.39, 3.60]	1.64 (0.110)	21.18 (1.03) vs. *19.57 (0.63)*
	Divorced or separated vs. *married or living with a partner*	–0.32 (0.63)	[–1.60, 0.96]	–0.51 (0.617)	19.26 (1.01) vs. *19.57 (0.63)*
	Widowed vs. *married or living with a partner*	–0.60 (0.58)	[–1.79, 0.58]	–1.04 (0.308)	18.97 (0.64) vs. *19.57 (0.63)*
Self-report general health condition	Poor vs. *excellent or very good*	3.45 (1.01)	[1.39, 5.50]	3.42 (0.002)	21.81 (1.09) vs. *18.37 (0.68)*
	Good or fair vs. *excellent or very good*	0.69 (0.45)	[–0.23, 1.61]	1.53 (0.136)	19.06 (0.60) vs. *18.37 (0.68)*
Difficulties in thinking or remembering	Yes vs. *no*	–1.78 (0.48)	[–2.77, –0.80]	–3.70 (0.001)	18.85 (0.79) vs. *20.64 (0.60)*
Ever told you have a heart disease	Yes vs. *no*	–0.48 (0.62)	[–1.75, 0.78]	–0.78 (0.442)	19.50 (0.84) vs. *19.99 (0.60)*
Ever told you had a stroke	Yes vs. *no*	0.49 (0.86)	[–1.27, 2.25]	0.57 (0.573)	19.99 (0.94) vs. *19.50 (0.59)*
Ever told you have diabetes	Yes vs. *no*	–0.71 (0.72)	[–2.18, 0.76]	–0.98 (0.335)	19.39 (0.77) vs. *20.10 (0.73)*
Have smoked at least 100 cigarettes in life	Yes vs. *no*	–0.39 (0.40)	[–1.22, 0.43]	–0.98 (0.336)	19.55 (0.57) vs. *19.94 (0.79)*
All combinations with abdominal obesity	Combinations with AO vs. *metabolically–healthy people*	–1.24 (0.51)	[–2.27, –0.20]	–2.43 (0.021)	19.13 (0.65) vs. *20.36 (0.76)*
Age at screening	Not applied (continuous covariate)	–0.18 (0.04)	[–0.25, –0.11]	–5.08 (< 0.001)	Not applied (continuous cov.)
Ratio of family income to the poverty level	Not applied (continuous covariate)	–0.04 (0.11)	[–0.28, 0.19]	–0.38 (0.705)	Not applied (continuous cov.)
Depression raw scores	Not applied (continuous covariate)	–0.02 (0.05)	[–0.13, 0.08]	–0.43 (0.667)	Not applied (continuous cov.)

[a]The column labeled "estimated marginal means" shows each category's marginal means against the reference category's marginal mean. The reference categories and the marginal means of the reference categories are shown in italics. The significant results have been colored.

Table S21. Multiple linear regression model explaining the relationship between CERAD-IR and all combinations without abdominal obesity after adjusting by sociodemographic characteristics and medical history, and estimated marginal means for each category of predictors and covariates[a]

**Model coefficient**	**Categories (for categorical predictor and covariates)**	*Beta* (*SE*)	95% CI	*t* (*p*)	Estimated marginal means[b]
Constant	Not applied (model intercept)	43.81 (4.58)	[34.25, 53.38]	9.56 (< 0.001)	Not applied (model intercept)
Education level	Up to 12th grade vs. *college graduate or above*	0.60 (1.18)	[–1.86, 3.06]	0.51 (0.615)	20.50 (1.05) vs. *19.90 (1.40)*
	High school graduate vs. *college graduate or above*	–3.43 (1.06)	[–5.64, –1.23]	–3.24 (0.004)	16.46 (1.51) vs. *19.90 (1.40)*
	Some college or AA degree vs. *college graduate or above*	0.04 (0.85)	[–1.74, 1.81]	0.04 (0.967)	19.93 (1.48) vs. *19.90 (1.40)*
Gender	Males vs. *females*	0.16 (0.62)	[–1.13, 1.45]	0.26 (0.798)	19.28 (1.06) vs. *19.12 (1.41)*
Race	Mexican American vs. *Non-Hispanic White*	–3.86 (1.06)	[–6.07, –1.65]	–3.64 (0.002)	18.10 (1.95) vs. *21.96 (1.16)*
	Other Hispanic vs. *Non-Hispanic White*	–3.15 (1.71)	[–6.72, 0.41]	–1.84 (0.080)	18.80 (1.85) vs. *21.96 (1.16)*
	Non–Hispanic Black vs. *Non-Hispanic White*	–3.41 (1.24)	[–5.99, –0.83]	–2.75 (0.012)	18.55 (1.56) vs. *21.96 (1.16)*
	Non–Hispanic Asian vs. *Non-Hispanic White*	–0.44 (1.30)	[–3.15, 2.26]	–0.34 (0.737)	21.51 (1.50) vs. *21.96 (1.16)*
	Other Race and Multi-Racial vs. *Non-Hispanic White*	–5.69 (0.97)	[–7.72, –3.67]	–5.87 (< 0.001)	16.26 (1.49) vs. *21.96 (1.16)*
Marital status	Single or never married vs. *married or living with a partner*	1.50 (1.79)	[–2.23, 5.23]	0.84 (0.411)	19.61 (2.41) vs. *18.11 (1.12)*
	Divorced or separated vs. *married or living with a partner*	1.85 (0.82)	[0.13, 3.57]	2.24 (0.036)	19.96 (1.07) vs. *18.11 (1.12)*
	Widowed vs. *married or living with a partner*	1.01 (1.28)	[–1.67, 3.68]	0.78 (0.442)	19.11 (1.48) vs. *18.11 (1.12)*
Self-report general health condition	Poor vs. *excellent or very good*	0.14 (1.44)	[–2.86, 3.14]	0.10 (0.925)	19.52 (1.45) vs. *19.38 (1.38)*
	Good or fair vs. *excellent or very good*	–0.69 (0.78)	[–2.32, 0.94]	–0.89 (0.385)	18.69 (1.52) vs. *19.38 (1.38)*
Difficulties in thinking or remembering	Yes vs. *no*	–0.31 (0.94)	[–2.27, 1.65]	–0.33 (0.746)	19.04 (1.53) vs. *19.35 (1.01)*
Ever told you have a heart disease	Yes vs. *no*	–1.96 (1.22)	[–4.50, 0.58]	–1.61 (0.123)	18.22 (1.73) vs. *20.18 (0.81)*
Ever told you had a stroke	Yes vs. *no*	0.77 (1.11)	[–1.54, 3.08]	0.70 (0.494)	19.58 (1.58) vs. *18.81 (1.01)*
Ever told you have diabetes	Yes vs. *no*	0.41 (1.35)	[–2.41, 3.23]	0.30 (0.764)	19.40 (1.40) vs. *18.99 (1.37)*
Have smoked at least 100 cigarettes in life	Yes vs. *no*	1.28 (0.77)	[–0.33, 2.89]	1.66 (0.113)	19.84 (1.21) vs. *18.56 (1.33)*
All combinations without abdominal obesity	Combinations without AO vs. *metabolically–healthy people*	–0.74 (0.86)	[–2.55, 1.06]	–0.86 (0.399)	18.82 (1.46) vs. *19.57 (1.07)*
Age at screening	Not applied (continuous covariate)	–0.30 (0.07)	[–0.44, –0.16]	–4.53 (< 0.001)	Not applied (continuous cov.)
Ratio of family income to the poverty level	Not applied (continuous covariate)	–0.41 (0.19)	[–0.80, –0.02]	–2.22 (0.038)	Not applied (continuous cov.)
Depression raw scores	Not applied (continuous covariate)	–0.13 (0.08)	[–0.30, 0.04]	–1.61 (0.122)	Not applied (continuous cov.)

[a]The design-based covariance matrix is singular. The validity of results is uncertain. [b]The column labeled "estimated marginal means" shows each category's marginal means against the reference category's marginal mean. The reference categories and the marginal means of the reference categories are shown in italics. The significant results have been colored.

Table S22. Multiple linear regression model explaining the relationship between CERAD-IR and all combinations with high glycemia obesity after adjusting by sociodemographic characteristics and medical history, and estimated marginal means for each category of predictors and covariates

**Model coefficient**	**Categories (for categorical predictor and covariates)**	*Beta* (*SE*)	95% CI	*t* (*p*)	Estimated marginal means[a]
Constant	Not applied (model intercept)	34.40 (2.59)	[29.14, 39.67]	13.31 (< 0.001)	Not applied (model intercept)
Education level	Up to 12th grade vs. *college graduate or above*	–1.32 (0.84)	[–3.03, 0.39]	–1.58 (0.125)	19.01 (0.99) vs. *20.33 (0.90)*
	High school graduate vs. *college graduate or above*	–0.83 (0.63)	[–2.12, 0.46]	–1.31 (0.198)	19.50 (0.63) vs. *20.33 (0.90)*
	Some college or AA degree vs. *college graduate or above*	0.37 (0.64)	[–0.93, 1.68]	0.58 (0.567)	20.70 (0.83) vs. *20.33 (0.90)*
Gender	Males vs. *females*	–1.37 (0.42)	[–2.24, –0.51]	–3.23 (0.003)	19.20 (0.77) vs. *20.57 (0.79)*
Race	Mexican American vs. *Non-Hispanic White*	–2.08 (0.70)	[–3.50, –0.66]	–2.99 (0.005)	18.98 (0.95) vs. *21.07 (0.77)*
	Other Hispanic vs. *Non-Hispanic White*	–2.20 (0.71)	[–3.65, –0.75]	–3.10 (0.004)	18.86 (0.84) vs. *21.07 (0.77)*
	Non–Hispanic Black vs. *Non-Hispanic White*	–1.04 (0.52)	[–2.10, 0.03]	–1.99 (0.055)	20.03 (0.83) vs. *21.07 (0.77)*
	Non–Hispanic Asian vs. *Non-Hispanic White*	0.77 (0.84)	[–0.93, 2.47]	0.92 (0.363)	21.84 (0.94) vs. *21.07 (0.77)*
	Other Race and Multi-Racial vs. *Non-Hispanic White*	–2.55 (1.24)	[–5.07, –0.03]	–2.06 (0.047)	18.52 (1.30) vs. *21.07 (0.77)*
Marital status	Single or never married vs. *married or living with a partner*	1.79 (0.89)	[–0.03, 3.61]	2.00 (0.054)	21.37 (1.01) vs. *19.58 (0.72)*
	Divorced or separated vs. *married or living with a partner*	–0.01 (0.55)	[–1.12, 1.10]	–0.01 (0.991)	19.58 (1.05) vs. *19.58 (0.72)*
	Widowed vs. *married or living with a partner*	–0.58 (0.60)	[–1.81, 0.65]	–0.96 (0.344)	19.00 (0.77) vs. *19.58 (0.72)*
Self-report general health condition	Poor vs. *excellent or very good*	3.50 (0.93)	[1.61, 5.40]	3.77 (0.001)	22.03 (1.12) vs. *18.53 (0.77)*
	Good or fair vs. *excellent or very good*	0.56 (0.53)	[–0.53, 1.64]	1.04 (0.305)	19.09 (0.66) vs. *18.53 (0.77)*
Difficulties in thinking or remembering	Yes vs. *no*	–1.26 (0.58)	[–2.44, –0.08]	–2.17 (0.038)	19.25 (0.86) vs. *20.51 (0.74)*
Ever told you have a heart disease	Yes vs. *no*	–0.78 (0.66)	[–2.12, 0.57]	–1.18 (0.249)	19.49 (0.94) vs. *20.27 (0.66)*
Ever told you had a stroke	Yes vs. *no*	0.46 (0.94)	[–1.44, 2.37]	0.49 (0.626)	20.11 (1.09) vs. *19.65 (0.61)*
Ever told you have diabetes	Yes vs. *no*	–0.64 (0.76)	[–2.19, 0.92]	–0.83 (0.411)	19.56 (0.86) vs. *20.20 (0.82)*
Have smoked at least 100 cigarettes in life	Yes vs. *no*	–0.26 (0.40)	[–1.08, 0.55]	–0.66 (0.515)	19.75 (0.65) vs. *20.02 (0.88)*
All combinations with high glycemia	Combinations with GLY vs. *metabolically–healthy people*	–1.12 (0.52)	[–2.18, –0.05]	–2.13 (0.041)	19.32 (0.68) vs. *20.44 (0.89)*
Age at screening	Not applied (continuous covariate)	–0.17 (0.04)	[–0.24, –0.09]	–4.54 (< 0.001)	Not applied (continuous cov.)
Ratio of family income to the poverty level	Not applied (continuous covariate)	–0.15 (0.12)	[–0.39, 0.09]	–1.29 (0.205)	Not applied (continuous cov.)
Depression raw scores	Not applied (continuous covariate)	–0.06 (0.05)	[–0.17, 0.05]	–1.05 (0.300)	Not applied (continuous cov.)

[a]The column labeled "estimated marginal means" shows each category's marginal means against the reference category's marginal mean. The reference categories and the marginal means of the reference categories are shown in italics. The significant results have been colored.

Table S23. Multiple linear regression model explaining the relationship between CERAD-IR and all combinations without high glycemia obesity after adjusting by sociodemographic characteristics and medical history, and estimated marginal means for each category of predictors and covariates[a]

**Model coefficient**	**Categories (for categorical predictor and covariates)**	*Beta* (*SE*)	95% CI	*t* (*p*)	Estimated marginal means[b]
Constant	Not applied (model intercept)	45.16 (4.30)	[36.10, 54.23]	10.51 (< 0.001)	Not applied (model intercept)
Education level	Up to 12th grade vs. *college graduate or above*	–1.86 (0.97)	[–3.91, 0.19]	–1.92 (0.072)	17.78 (1.01) vs. *19.65 (0.96)*
	High school graduate vs. *college graduate or above*	–3.67 (1.09)	[–5.97, –1.38]	–3.37 (0.004)	15.97 (1.09) vs. *19.65 (0.96)*
	Some college or AA degree vs. *college graduate or above*	–1.66 (0.71)	[–3.16, –0.15]	–2.32 (0.033)	17.99 (1.06) vs. *19.65 (0.96)*
Gender	Males vs. *females*	–0.91 (0.62)	[–2.22, 0.41]	–1.46 (0.164)	17.40 (0.94) vs. *18.30 (0.92)*
Race	Mexican American vs. *Non-Hispanic White*	–1.41 (1.28)	[–4.11, 1.29]	–1.10 (0.286)	18.36 (1.66) vs. *19.77 (0.74)*
	Other Hispanic vs. *Non-Hispanic White*	–3.49 (1.26)	[–6.15, –0.82]	–2.76 (0.013)	16.28 (1.09) vs. *19.77 (0.74)*
	Non–Hispanic Black vs. *Non-Hispanic White*	–1.68 (0.73)	[–3.22, –0.14]	–2.30 (0.034)	18.09 (0.80) vs. *19.77 (0.74)*
	Non–Hispanic Asian vs. *Non-Hispanic White*	–0.82 (1.72)	[–4.44, 2.80]	–0.48 (0.637)	18.94 (1.96) vs. *19.77 (0.74)*
	Other Race and Multi-Racial vs. *Non-Hispanic White*	–4.10 (1.52)	[–7.32, –0.88]	–2.69 (0.015)	15.67 (1.81) vs. *19.77 (0.74)*
Marital status	Single or never married vs. *married or living with a partner*	–1.32 (2.14)	[–5.83, 3.19]	–0.62 (0.544)	16.89 (2.39) vs. *18.21 (0.95)*
	Divorced or separated vs. *married or living with a partner*	–0.62 (1.25)	[–3.25, 2.02]	–0.49 (0.628)	17.59 (1.01) vs. *18.21 (0.95)*
	Widowed vs. *married or living with a partner*	0.50 (0.90)	[–1.40, 2.40]	0.56 (0.585)	18.71 (0.88) vs. *18.21 (0.95)*
Self-report general health condition	Poor vs. *excellent or very good*	1.31 (1.50)	[–1.85, 4.47]	0.87 (0.394)	18.47 (1.24) vs. *17.16 (1.08)*
	Good or fair vs. *excellent or very good*	0.75 (0.64)	[–0.60, 2.11]	1.17 (0.259)	17.91 (0.97) vs. *17.16 (1.08)*
Difficulties in thinking or remembering	Yes vs. *no*	–1.51 (0.86)	[–3.33, 0.31]	–1.75 (0.098)	17.09 (1.02) vs. *18.61 (0.92)*
Ever told you have a heart disease	Yes vs. *no*	–0.39 (0.89)	[–2.27, 1.49]	–0.44 (0.664)	17.65 (1.12) vs. *18.05 (0.82)*
Ever told you had a stroke	Yes vs. *no*	0.84 (1.27)	[–1.84, 3.52]	0.66 (0.517)	18.27 (1.37) vs. *17.43 (0.68)*
Ever told you have diabetes	Yes vs. *no*	–0.62 (1.03)	[–2.79, 1.56]	–0.60 (0.557)	17.54 (0.94) vs. *18.16 (1.09)*
Have smoked at least 100 cigarettes in life	Yes vs. *no*	–0.20 (0.76)	[–1.80, 1.39]	–0.27 (0.792)	17.75 (0.85) vs. *17.95 (1.04)*
All combinations without high glycemia	Combinations without GLY vs. *metabolically–healthy people*	–2.39 (0.80)	[–4.08, –0.70]	–2.99 (0.008)	16.65 (0.97) vs. *19.04 (0.95)*
Age at screening	Not applied (continuous covariate)	–0.32 (0.07)	[–0.46, –0.18]	–4.92 (< 0.001)	Not applied (continuous cov.)
Ratio of family income to the poverty level	Not applied (continuous covariate)	0.08 (0.22)	[–0.39, 0.55]	0.38 (0.711)	Not applied (continuous cov.)
Depression raw scores	Not applied (continuous covariate)	0.08 (0.04)	[–0.01, 0.16]	1.84 (0.083)	Not applied (continuous cov.)

[a]The design-based covariance matrix is singular. The validity of results is uncertain. [b]The column labeled "estimated marginal means" shows each category's marginal means against the reference category's marginal mean. The reference categories and the marginal means of the reference categories are shown in italics. The significant results have been colored.

Table S24. Multiple linear regression model explaining the relationship between CERAD-DR and classic MetS diagnosis after adjusting by sociodemographic characteristics and medical history, and estimated marginal means for each category of predictors and covariates

**Model coefficient**	**Categories (for categorical predictor and covariates)**	*Beta* (*SE*)	95% CI	*t* (*p*)	Estimated marginal means[a]
Constant	Not applied (model intercept)	12.09 (1.19)	[9.67, 14.50]	10.19 (< 0.001)	Not applied (model intercept)
Education level	Up to 12th grade vs. *college graduate or above*	–0.48 (0.41)	[–1.32, 0.36]	–1.17 (0.249)	5.87 (0.38) vs. *6.35 (0.44)*
	High school graduate vs. *college graduate or above*	–0.31 (0.36)	[–1.05, 0.43]	–0.86 (0.399)	6.04 (0.42) vs. *6.35 (0.44)*
	Some college or AA degree vs. *college graduate or above*	0.53 (0.29)	[–0.05, 1.11]	1.86 (0.072)	6.88 (0.39) vs. *6.35 (0.44)*
Gender	Males vs. *females*	–0.29 (0.19)	[–0.67, 0.10]	–1.53 (0.135)	6.14 (0.34) vs. *6.43 (0.39)*
Race	Mexican American vs. *Non-Hispanic White*	–0.42 (0.36)	[–1.15, 0.32]	–1.16 (0.255)	6.38 (0.45) vs. *6.80 (0.38)*
	Other Hispanic vs. *Non-Hispanic White*	–1.12 (0.38)	[–1.89, –0.34]	–2.94 (0.006)	5.69 (0.43) vs. *6.80 (0.38)*
	Non–Hispanic Black vs. *Non-Hispanic White*	–0.47 (0.29)	[–1.07, 0.12]	–1.62 (0.116)	6.33 (0.47) vs. *6.80 (0.38)*
	Non–Hispanic Asian vs. *Non-Hispanic White*	0.82 (0.36)	[0.08, 1.56]	2.26 (0.031)	7.63 (0.46) vs. *6.80 (0.38)*
	Other Race and Multi-Racial vs. *Non-Hispanic White*	–1.90 (0.42)	[–2.77, –1.04]	–4.48 (< 0.001)	4.90 (0.47) vs. *6.80 (0.38)*
Marital status	Single or never married vs. *married or living with a partner*	1.08 (0.44)	[0.18, 1.98]	2.44 (0.021)	7.13 (0.48) vs. *6.05 (0.35)*
	Divorced or separated vs. *married or living with a partner*	0.12 (0.23)	[–0.36, 0.60]	0.51 (0.610)	6.17 (0.44) vs. *6.05 (0.35)*
	Widowed vs. *married or living with a partner*	–0.26 (0.30)	[–0.87, 0.35]	–0.86 (0.396)	5.79 (0.45) vs. *6.05 (0.35)*
Self-report general health condition	Poor vs. *excellent or very good*	1.66 (0.71)	[0.22, 3.10]	2.35 (0.025)	7.36 (0.64) vs. *5.69 (0.41)*
	Good or fair vs. *excellent or very good*	0.12 (0.28)	[–0.44, 0.68]	0.44 (0.662)	5.81 (0.34) vs. *5.69 (0.41)*
Difficulties in thinking or remembering	Yes vs. *no*	–1.11 (0.27)	[–1.65, –0.57]	–4.20 (< 0.001)	5.73 (0.43) vs. *6.84 (0.32)*
Ever told you have a heart disease	Yes vs. *no*	–0.13 (0.38)	[–0.91, 0.65]	–0.34 (0.738)	6.22 (0.48) vs. *6.35 (0.32)*
Ever told you had a stroke	Yes vs. *no*	0.18 (0.42)	[–0.67, 1.02]	0.43 (0.673)	6.38 (0.52) vs. *6.20 (0.28)*
Ever told you have diabetes	Yes vs. *no*	–0.46 (0.35)	[–1.18, 0.25]	–1.32 (0.196)	6.06 (0.39) vs. *6.52 (0.41)*
Have smoked at least 100 cigarettes in life	Yes vs. *no*	–0.28 (0.22)	[–0.72, 0.16]	–1.30 (0.203)	6.15 (0.33) vs. *6.43 (0.41)*
Classic MetS diagnosis (all three or more criteria)	MetS vs. *metabolically–healthy people*	–0.57 (0.26)	[–1.10, –0.05]	–2.22 (0.034)	6.00 (0.33) vs. *6.57 (0.43)*
Age at screening	Not applied (continuous covariate)	–0.07 (0.02)	[–0.10, –0.03]	–4.14 (< 0.001)	Not applied (continuous cov.)
Ratio of family income to the poverty level	Not applied (continuous covariate)	0.03 (0.08)	[–0.13, 0.19]	0.41 (0.686)	Not applied (continuous cov.)
Depression raw scores	Not applied (continuous covariate)	–0.01 (0.02)	[–0.05, 0.03]	–0.62 (0.541)	Not applied (continuous cov.)

[a]The column labeled "estimated marginal means" shows each category's marginal means against the reference category's marginal mean. The reference categories and the marginal means of the reference categories are shown in italics. The significant results have been colored.

Table S25. Multiple linear regression model explaining the relationship between CERAD-DR and specific combination AO + TRI + HDL after adjusting by sociodemographic characteristics and medical history, and estimated marginal means for each category of predictors and covariates[a]

**Model coefficient**	**Categories (for categorical predictor and covariates)**	*Beta* (*SE*)	95% CI	*t* (*p*)	Estimated marginal means[b]
Constant	Not applied (model intercept)	19.49 (1.58)	[16.07, 22.91]	12.30 (< 0.001)	Not applied (model intercept)
Education level	Up to 12th grade vs. *college graduate or above*	0.26 (0.34)	[–0.47, 1.00]	0.77 (0.453)	6.03 (0.41) vs. *5.77 (0.23)*
	High school graduate vs. *college graduate or above*	–1.27 (0.53)	[–2.42, –0.12]	–2.39 (0.033)	4.50 (0.54) vs. *5.77 (0.23)*
	Some college or AA degree vs. *college graduate or above*	0.01 (0.33)	[–0.71, 0.73]	0.03 (0.979)	5.78 (0.37) vs. *5.77 (0.23)*
Gender	Males vs. *females*	–0.74 (0.25)	[–1.28, –0.19]	–2.91 (0.012)	5.15 (0.30) vs. *5.89 (0.41)*
Race	Mexican American vs. *Non-Hispanic White*	–1.02 (0.53)	[–2.17, 0.13]	–1.92 (0.077)	5.24 (0.62) vs. *6.26 (0.48)*
	Other Hispanic vs. *Non-Hispanic White*	–1.48 (0.73)	[–3.05, 0.08]	–2.05 (0.061)	4.78 (0.63) vs. *6.26 (0.48)*
	Non–Hispanic Black vs. *Non-Hispanic White*	–1.03 (0.61)	[–2.34, 0.28]	–1.70 (0.113)	5.23 (0.33) vs. *6.26 (0.48)*
	Non–Hispanic Asian vs. *Non-Hispanic White*	–0.20 (0.48)	[–1.23, 0.84]	–0.41 (0.689)	6.07 (0.54) vs. *6.26 (0.48)*
	Other Race and Multi-Racial vs. *Non-Hispanic White*	No cases	No cases	No cases	No cases
Marital status	Single or never married vs. *married or living with a partner*	0.13 (0.74)	[–1.46, 1.72]	0.18 (0.864)	5.85 (0.86) vs. *5.72 (0.30)*
	Divorced or separated vs. *married or living with a partner*	–0.05 (0.44)	[–0.99, 0.90]	–0.10 (0.919)	5.68 (0.58) vs. *5.72 (0.30)*
	Widowed vs. *married or living with a partner*	–0.89 (0.50)	[–1.97, 0.18]	–1.80 (0.096)	4.83 (0.37) vs. *5.72 (0.30)*
Self-report general health condition	Poor vs. *excellent or very good*	1.74 (0.70)	[0.24, 3.25]	2.50 (0.027)	6.78 (0.49) vs. *5.03 (0.51)*
	Good or fair vs. *excellent or very good*	–0.29 (0.27)	[–0.88, 0.30]	–1.06 (0.308)	4.74 (0.39) vs. *5.03 (0.51)*
Difficulties in thinking or remembering	Yes vs. *no*	–0.76 (0.21)	[–1.22, –0.29]	–3.53 (0.004)	5.14 (0.33) vs. *5.90 (0.37)*
Ever told you have a heart disease	Yes vs. *no*	–1.22 (0.55)	[–2.41, –0.03]	–2.22 (0.045)	4.91 (0.53) vs. *6.13 (0.31)*
Ever told you had a stroke	Yes vs. *no*	0.30 (0.55)	[–0.89, 1.48]	0.54 (0.597)	5.67 (0.58) vs. *5.37 (0.20)*
Ever told you have diabetes	Yes vs. *no*	0.29 (0.35)	[–0.46, 1.04]	0.83 (0.420)	5.66 (0.33) vs. *5.37 (0.42)*
Have smoked at least 100 cigarettes in life	Yes vs. *no*	–0.27 (0.33)	[–0.99, 0.45]	–0.80 (0.437)	5.38 (0.26) vs. *5.65 (0.46)*
Three criteria combination: AO + TRI + HDL	AO + TRI + HDL vs. *metabolically–healthy people*	–2.39 (0.46)	[–3.38, –1.40]	–5.21 (< 0.001)	4.32 (0.49) vs. *6.71 (0.30)*
Age at screening	Not applied (continuous covariate)	–0.16 (0.02)	[–0.21, –0.12]	–7.50 (< 0.001)	Not applied (continuous cov.)
Ratio of family income to the poverty level	Not applied (continuous covariate)	–0.06 (0.07)	[–0.21, 0.09]	–0.92 (0.372)	Not applied (continuous cov.)
Depression raw scores	Not applied (continuous covariate)	0.12 (0.02)	[0.07, 0.17]	5.03 (< 0.001)	Not applied (continuous cov.)

[a]The design-based covariance matrix is singular. The validity of results is uncertain. [b]The column labeled "estimated marginal means" shows each category's marginal means against the reference category's marginal mean. The reference categories and the marginal means of the reference categories are shown in italics. The significant results have been colored.

Table S26. Multiple linear regression model explaining the relationship between CERAD-DR and specific combination AO + TRI + HBP after adjusting by sociodemographic characteristics and medical history, and estimated marginal means for each category of predictors and covariates[a]

**Model coefficient**	**Categories (for categorical predictor and covariates)**	*Beta* (*SE*)	95% CI	*t* (*p*)	Estimated marginal means[b]
Constant	Not applied (model intercept)	18.17 (0.92)	[16.20, 20.15]	19.74 (< 0.001)	Not applied (model intercept)
Education level	Up to 12th grade vs. *college graduate or above*	0.78 (0.41)	[−0.10, 1.66]	1.90 (0.079)	7.05 (0.83) vs. *6.27 (0.61)*
	High school graduate vs. *college graduate or above*	−1.05 (0.46)	[−2.04, −0.06]	−2.27 (0.040)	5.22 (0.73) vs. *6.27 (0.61)*
	Some college or AA degree vs. *college graduate or above*	−0.24 (0.27)	[−0.81, 0.33]	−0.90 (0.382)	6.03 (0.74) vs. *6.27 (0.61)*
Gender	Males vs. *females*	−1.17 (0.25)	[−1.70, −0.64]	−4.75 (< 0.001)	5.56 (0.71) vs. *6.73 (0.69)*
Race	Mexican American vs. *Non-Hispanic White*	−0.71 (0.81)	[−2.46, 1.04]	−0.87 (0.398)	5.69 (0.99) vs. *6.41 (0.70)*
	Other Hispanic vs. *Non-Hispanic White*	−0.47 (0.67)	[−1.90, 0.96]	−0.71 (0.491)	5.93 (0.62) vs. *6.41 (0.70)*
	Non–Hispanic Black vs. *Non-Hispanic White*	0.08 (0.43)	[−0.84, 1.00]	0.18 (0.857)	6.48 (0.83) vs. *6.41 (0.70)*
	Non–Hispanic Asian vs. *Non-Hispanic White*	−0.21 (0.43)	[−1.14, 0.72]	−0.49 (0.632)	6.19 (0.93) vs. *6.41 (0.70)*
	Other Race and Multi-Racial vs. *Non-Hispanic White*	No cases	No cases	No cases	No cases
Marital status	Single or never married vs. *married or living with a partner*	−0.41 (0.98)	[−2.51, 1.68]	−0.42 (0.679)	6.22 (1.33) vs. *6.64 (0.75)*
	Divorced or separated vs. *married or living with a partner*	0.01 (0.72)	[−1.54, 1.56]	0.01 (0.989)	6.65 (0.75) vs. *6.64 (0.75)*
	Widowed vs. *married or living with a partner*	−1.58 (0.45)	[−2.53, −0.62]	−3.54 (0.003)	5.06 (0.54) vs. *6.64 (0.75)*
Self-report general health condition	Poor vs. *excellent or very good*	No cases	No cases	No cases	No cases
	Good or fair vs. *excellent or very good*	−0.15 (0.32)	[−0.84, 0.54]	−0.47 (0.648)	6.07 (0.64) vs. *6.22 (0.77)*
Difficulties in thinking or remembering	Yes vs. *no*	−1.08 (0.48)	[−2.11, −0.06]	−2.26 (0.040)	5.60 (0.73) vs. *6.68 (0.73)*
Ever told you have a heart disease	Yes vs. *no*	−0.91 (0.46)	[−1.89, 0.08]	−1.97 (0.069)	5.69 (0.81) vs. *6.60 (0.63)*
Ever told you had a stroke	Yes vs. *no*	2.15 (1.28)	[−0.60, 4.90]	1.68 (0.116)	7.22 (1.28) vs. *5.07 (0.37)*
Ever told you have diabetes	Yes vs. *no*	0.22 (0.49)	[−0.82, 1.26]	0.46 (0.656)	6.25 (0.71) vs. *6.03 (0.75)*
Have smoked at least 100 cigarettes in life	Yes vs. *no*	−0.25 (0.32)	[−0.93, 0.43]	−0.80 (0.439)	6.02 (0.62) vs. *6.27 (0.78)*
Three criteria combination: AO + TRI + HBP	AO + TRI + HBP vs. *metabolically–healthy people*	−1.87 (0.42)	[−2.78, −0.97]	−4.43 (0.001)	5.20 (0.81) vs. *7.08 (0.62)*
Age at screening	Not applied (continuous covariate)	−0.14 (0.01)	[−0.17, −0.12]	−11.86 (< 0.001)	Not applied (continuous cov.)
Ratio of family income to the poverty level	Not applied (continuous covariate)	0.03 (0.09)	[−0.18, 0.23]	0.28 (0.782)	Not applied (continuous cov.)
Depression raw scores	Not applied (continuous covariate)	−0.01 (0.04)	[−0.09, 0.08]	−0.12 (0.905)	Not applied (continuous cov.)

[a]The design-based covariance matrix is singular. The validity of results is uncertain. [b]The column labeled "estimated marginal means" shows each category's marginal means against the reference category's marginal mean. The reference categories and the marginal means of the reference categories are shown in italics. The significant results have been colored.

Table S27. Multiple linear regression model explaining the relationship between CERAD-DR and specific combination AO + TRI + GLY after adjusting by sociodemographic characteristics and medical history, and estimated marginal means for each category of predictors and covariates[a]

**Model coefficient**	**Categories (for categorical predictor and covariates)**	*Beta* (*SE*)	95% CI	*t* (*p*)	Estimated marginal means[b]
Constant	Not applied (model intercept)	15.14 (2.56)	[9.81, 20.47]	5.91 (< 0.001)	Not applied (model intercept)
Education level	Up to 12th grade vs. *college graduate or above*	–0.02 (0.71)	[–1.50, 1.46]	–0.03 (0.980)	5.56 (0.90) vs. *5.57 (1.06)*
	High school graduate vs. *college graduate or above*	–1.38 (0.69)	[–2.81, 0.06]	–1.99 (0.060)	4.20 (0.79) vs. *5.57 (1.06)*
	Some college or AA degree vs. *college graduate or above*	–0.53 (0.57)	[–1.71, 0.65]	–0.93 (0.363)	5.04 (1.01) vs. *5.57 (1.06)*
Gender	Males vs. *females*	–0.76 (0.39)	[–1.57, 0.05]	–1.96 (0.064)	4.71 (0.78) vs. *5.47 (1.00)*
Race	Mexican American vs. *Non-Hispanic White*	–0.92 (0.50)	[–1.97, 0.13]	–1.81 (0.084)	4.36 (1.03) vs. *5.28 (0.74)*
	Other Hispanic vs. *Non-Hispanic White*	–0.70 (0.98)	[–f2.74, 1.34]	–0.71 (0.485)	4.58 (1.25) vs. *5.28 (0.74)*
	Non–Hispanic Black vs. *Non-Hispanic White*	0.15 (0.54)	[–0.97, 1.28]	0.29 (0.778)	5.43 (1.05) vs. *5.28 (0.74)*
	Non–Hispanic Asian vs. *Non-Hispanic White*	0.54 (0.59)	[–0.68, 1.77]	0.92 (0.367)	5.82 (0.91) vs. *5.28 (0.74)*
	Other Race and Multi-Racial vs. *Non-Hispanic White*	No cases	No cases	No cases	No cases
Marital status	Single or never married vs. *married or living with a partner*	–0.04 (0.74)	[–1.58, 1.50]	–0.05 (0.959)	5.27 (1.11) vs. *5.31 (0.81)*
	Divorced or separated vs. *married or living with a partner*	–0.09 (0.52)	[–1.16, 0.99]	–0.17 (0.869)	5.22 (1.00) vs. *5.31 (0.81)*
	Widowed vs. *married or living with a partner*	–0.73 (0.61)	[–2.00, 0.54]	–1.20 (0.245)	4.58 (1.02) vs. *5.31 (0.81)*
Self-report general health condition	Poor vs. *excellent or very good*	–0.15 (1.26)	[–2.77, 2.46]	–0.12 (0.903)	4.88 (1.46) vs. *5.03 (0.79)*
	Good or fair vs. *excellent or very good*	0.34 (0.61)	[–0.94, 1.62]	0.55 (0.585)	5.37 (0.69) vs. *5.03 (0.79)*
Difficulties in thinking or remembering	Yes vs. *no*	–0.71 (0.61)	[–1.98, 0.57]	–1.15 (0.262)	4.74 (1.00) vs. *5.45 (0.84)*
Ever told you have a heart disease	Yes vs. *no*	–1.19 (0.72)	[–2.69, 0.32]	–1.64 (0.116)	4.50 (1.18) vs. *5.69 (0.62)*
Ever told you had a stroke	Yes vs. *no*	–0.42 (1.37)	[–3.26, 2.42]	–0.31 (0.761)	4.88 (1.41) vs. *5.30 (0.69)*
Ever told you have diabetes	Yes vs. *no*	–1.50 (1.72)	[–5.08, 2.07]	–0.87 (0.392)	4.34 (1.11) vs. *5.84 (1.33)*
Have smoked at least 100 cigarettes in life	Yes vs. *no*	0.18 (0.63)	[–1.13, 1.50]	0.29 (0.774)	5.18 (0.94) vs. *5.00 (0.91)*
Three criteria combination: AO + TRI + GLY	AO + TRI + HBP vs. *metabolically–healthy people*	0.13 (0.47)	[–0.85, 1.11]	0.28 (0.785)	5.16 (0.86) vs. *5.03 (0.94)*
Age at screening	Not applied (continuous covariate)	–0.11 (0.03)	[–0.18, –0.03]	–3.09 (0.006)	Not applied (continuous cov.)
Ratio of family income to the poverty level	Not applied (continuous covariate)	0.14 (0.13)	[–0.13, 0.42]	1.07 (0.297)	Not applied (continuous cov.)
Depression raw scores	Not applied (continuous covariate)	–0.07 (0.06)	[–0.19, 0.05]	–1.23 (0.234)	Not applied (continuous cov.)

[a]The design-based covariance matrix is singular. The validity of results is uncertain. [b]The column labeled "estimated marginal means" shows each category's marginal means against the reference category's marginal mean. The reference categories and the marginal means of the reference categories are shown in italics. The significant results have been colored.

Table S28. Multiple linear regression model explaining the relationship between CERAD-DR and specific combination AO + HDL + HBP after adjusting by sociodemographic characteristics and medical history, and estimated marginal means for each category of predictors and covariates[a]

**Model coefficient**	**Categories (for categorical predictor and covariates)**	*Beta* (*SE*)	95% CI	*t* (*p*)	Estimated marginal means[b]
Constant	Not applied (model intercept)	18.88 (1.90)	[14.85, 22.91]	9.92 (< 0.001)	Not applied (model intercept)
Education level	Up to 12th grade vs. *college graduate or above*	0.70 (0.40)	[–0.14, 1.54]	1.77 (0.097)	5.48 (0.35) vs. *4.78 (0.53)*
	High school graduate vs. *college graduate or above*	–1.34 (0.61)	[–2.62, –0.05]	–2.21 (0.042)	3.44 (0.46) vs. *4.78 (0.53)*
	Some college or AA degree vs. *college graduate or above*	0.41 (0.43)	[–0.50, 1.33]	0.95 (0.356)	5.19 (0.45) vs. *4.78 (0.53)*
Gender	Males vs. *females*	–0.72 (0.36)	[–1.49, 0.05]	–1.97 (0.066)	4.37 (0.37) vs. *5.08 (0.44)*
Race	Mexican American vs. *Non-Hispanic White*	–1.24 (0.51)	[–2.32, –0.17]	–2.45 (0.026)	4.45 (0.58) vs. *5.70 (0.38)*
	Other Hispanic vs. *Non-Hispanic White*	–1.29 (0.53)	[–2.42, –0.16]	–2.42 (0.028)	4.41 (0.67) vs. *5.70 (0.38)*
	Non–Hispanic Black vs. *Non-Hispanic White*	–0.61 (0.48)	[–1.63, 0.42]	–1.25 (0.229)	5.09 (0.52) vs. *5.70 (0.38)*
	Non–Hispanic Asian vs. *Non-Hispanic White*	–0.13 (0.49)	[–1.17, 0.91]	–0.27 (0.791)	5.57 (0.57) vs. *5.70 (0.38)*
	Other Race and Multi-Racial vs. *Non-Hispanic White*	–2.57 (0.81)	[–4.27, –0.86]	–3.19 (0.006)	3.13 (0.74) vs. *5.70 (0.38)*
Marital status	Single or never married vs. *married or living with a partner*	0.65 (0.63)	[–0.68, 1.98]	1.04 (0.315)	5.18 (0.60) vs. *4.53 (0.43)*
	Divorced or separated vs. *married or living with a partner*	1.08 (0.37)	[0.30, 1.87]	2.92 (0.010)	5.62 (0.44) vs. *4.53 (0.43)*
	Widowed vs. *married or living with a partner*	–0.96 (0.48)	[–1.98, 0.05]	–2.02 (0.061)	3.57 (0.67) vs. *4.53 (0.43)*
Self-report general health condition	Poor vs. *excellent or very good*	No cases	No cases	No cases	No cases
	Good or fair vs. *excellent or very good*	–0.48 (0.32)	[–1.16, 0.21]	–1.48 (0.159)	4.49 (0.35) vs. *4.96 (0.44)*
Difficulties in thinking or remembering	Yes vs. *no*	–0.67 (0.34)	[–1.38, 0.05]	–1.97 (0.067)	4.39 (0.44) vs. *5.06 (0.36)*
Ever told you have a heart disease	Yes vs. *no*	–1.01 (0.62)	[–2.32, 0.30]	–1.64 (0.122)	4.22 (0.58) vs. *5.23 (0.34)*
Ever told you had a stroke	Yes vs. *no*	–1.61 (0.76)	[–3.21, –0.01]	–2.13 (0.049)	3.92 (0.67) vs. *5.53 (0.31)*
Ever told you have diabetes	Yes vs. *no*	–0.16 (0.37)	[–0.95, 0.62]	–0.44 (0.665)	4.64 (0.31) vs. *4.81 (0.48)*
Have smoked at least 100 cigarettes in life	Yes vs. *no*	0.12 (0.47)	[–0.88, 1.12]	0.26 (0.800)	4.79 (0.41) vs. *4.66 (0.46)*
Three criteria combination: AO + HDL + HBP	AO + HDL + HBP vs. *metabolically–healthy people*	–0.37 (0.63)	[–1.69, 0.96]	–0.58 (0.567)	4.54 (0.53) vs. *4.91 (0.42)*
Age at screening	Not applied (continuous covariate)	–0.16 (0.03)	[–0.22, –0.10]	–5.52 (< 0.001)	Not applied (continuous cov.)
Ratio of family income to the poverty level	Not applied (continuous covariate)	–0.14 (0.09)	[–0.33, 0.06]	–1.51 (0.151)	Not applied (continuous cov.)
Depression raw scores	Not applied (continuous covariate)	0.03 (0.05)	[–0.08, 0.14]	0.50 (0.623)	Not applied (continuous cov.)

[a]The design-based covariance matrix is singular. The validity of results is uncertain. [b]The column labeled "estimated marginal means" shows each category's marginal means against the reference category's marginal mean. The reference categories and the marginal means of the reference categories are shown in italics. The significant results have been colored.

Table S29. Multiple linear regression model explaining the relationship between CERAD-DR and specific combination AO + HDL + GLY after adjusting by sociodemographic characteristics and medical history, and estimated marginal means for each category of predictors and covariates[a]

**Model coefficient**	**Categories (for categorical predictor and covariates)**	*Beta* (*SE*)	95% CI	*t* (*p*)	Estimated marginal means[b]
Constant	Not applied (model intercept)	12.97 (2.26)	[8.30, 17.64]	5.74 (< 0.001)	Not applied (model intercept)
Education level	Up to 12th grade vs. *college graduate or above*	–0.43 (0.72)	[–1.92, 1.06]	–0.59 (0.558)	6.40 (0.78) vs. *6.83 (0.91)*
	High school graduate vs. *college graduate or above*	–0.61 (0.44)	[–1.52, 0.30]	–1.39 (0.177)	6.22 (1.01) vs. *6.83 (0.91)*
	Some college or AA degree vs. *college graduate or above*	0.24 (0.39)	[–0.57, 1.05]	0.61 (0.548)	7.07 (0.95) vs. *6.83 (0.91)*
Gender	Males vs. *females*	–0.48 (0.40)	[–1.31, 0.34]	–1.21 (0.237)	6.39 (0.85) vs. *6.87 (0.84)*
Race	Mexican American vs. *Non-Hispanic White*	–1.24 (0.72)	[–2.73, 0.25]	–1.72 (0.098)	5.61 (0.76) vs. *6.85 (0.61)*
	Other Hispanic vs. *Non-Hispanic White*	0.35 (1.17)	[–2.06, 2.76]	0.30 (0.764)	7.20 (1.40) vs. *6.85 (0.61)*
	Non–Hispanic Black vs. *Non-Hispanic White*	–0.12 (0.62)	[–1.40, 1.17]	–0.19 (0.855)	6.73 (0.84) vs. *6.85 (0.61)*
	Non–Hispanic Asian vs. *Non-Hispanic White*	0.65 (0.70)	[–0.80, 2.10]	0.92 (0.365)	7.50 (0.89) vs. *6.85 (0.61)*
	Other Race and Multi-Racial vs. *Non-Hispanic White*	–0.98 (0.95)	[–2.96, 0.99]	–1.03 (0.312)	5.87 (1.26) vs. *6.85 (0.61)*
Marital status	Single or never married vs. *married or living with a partner*	0.12 (0.81)	[–1.55, 1.79]	0.15 (0.883)	6.82 (1.16) vs. *6.70 (0.56)*
	Divorced or separated vs. *married or living with a partner*	0.63 (0.43)	[–0.26, 1.52]	1.45 (0.159)	7.33 (0.71) vs. *6.70 (0.56)*
	Widowed vs. *married or living with a partner*	–1.05 (0.74)	[–2.59, 0.48]	–1.42 (0.169)	5.65 (1.18) vs. *6.70 (0.56)*
Self-report general health condition	Poor vs. *excellent or very good*	2.08 (0.94)	[0.14, 4.01]	2.21 (0.037)	8.11 (1.33) vs. *6.03 (0.70)*
	Good or fair vs. *excellent or very good*	–0.29 (0.43)	[–1.18, 0.61]	–0.66 (0.514)	5.74 (0.58) vs. *6.03 (0.70)*
Difficulties in thinking or remembering	Yes vs. *no*	–0.72 (0.43)	[–1.62, 0.17]	–1.67 (0.108)	6.27 (0.88) vs. *6.99 (0.81)*
Ever told you have a heart disease	Yes vs. *no*	–2.04 (0.64)	[–3.37, –0.71]	–3.17 (0.004)	5.61 (1.00) vs. *7.65 (0.74)*
Ever told you had a stroke	Yes vs. *no*	1.82 (0.64)	[0.50, 3.13]	2.86 (0.009)	7.54 (1.04) vs. *5.72 (0.68)*
Ever told you have diabetes	Yes vs. *no*	–0.89 (0.56)	[–2.05, 0.27]	–1.59 (0.126)	6.18 (0.86) vs. *7.07 (0.87)*
Have smoked at least 100 cigarettes in life	Yes vs. *no*	–0.03 (0.33)	[–0.70, 0.65]	–0.09 (0.933)	6.61 (0.83) vs. *6.64 (0.84)*
Three criteria combination: AO + HDL + GLY	AO + HDL + GLY vs. *metabolically–healthy people*	–0.58 (0.64)	[–1.90, 0.74]	–0.91 (0.370)	6.34 (0.62) vs. *6.92 (1.08)*
Age at screening	Not applied (continuous covariate)	–0.07 (0.03)	[–0.13, –0.01]	–2.38 (0.026)	Not applied (continuous cov.)
Ratio of family income to the poverty level	Not applied (continuous covariate)	–0.01 (0.11)	[–0.25, 0.23]	–0.10 (0.919)	Not applied (continuous cov.)
Depression raw scores	Not applied (continuous covariate)	–0.05 (0.07)	[–0.19, 0.09]	–0.71 (0.482)	Not applied (continuous cov.)

[a]The design-based covariance matrix is singular. The validity of results is uncertain. [b]The column labeled "estimated marginal means" shows each category's marginal means against the reference category's marginal mean. The reference categories and the marginal means of the reference categories are shown in italics. The significant results have been colored.

Table S30. Multiple linear regression model explaining the relationship between CERAD-DR and specific combination AO + HBP + GLY after adjusting by sociodemographic characteristics and medical history, and estimated marginal means for each category of predictors and covariates

**Model coefficient**	**Categories (for categorical predictor and covariates)**	*Beta* (*SE*)	95% CI	*t* (*p*)	Estimated marginal means[a]
Constant	Not applied (model intercept)	14.41 (1.82)	[10.69, 18.12]	7.93 (< 0.001)	Not applied (model intercept)
Education level	Up to 12th grade vs. *college graduate or above*	0.43 (0.58)	[–0.75, 1.61]	0.75 (0.460)	5.75 (0.79) vs. *5.32 (0.92)*
	High school graduate vs. *college graduate or above*	–0.11 (0.42)	[–0.97, 0.76]	–0.25 (0.801)	5.21 (0.90) vs. *5.32 (0.92)*
	Some college or AA degree vs. *college graduate or above*	0.68 (0.50)	[–0.35, 1.71]	1.36 (0.185)	6.01 (0.84) vs. *5.32 (0.92)*
Gender	Males vs. *females*	–0.35 (0.38)	[–1.12, 0.42]	–0.93 (0.361)	5.40 (0.79) vs. *5.75 (0.87)*
Race	Mexican American vs. *Non–Hispanic White*	–0.09 (0.45)	[–1.01, 0.83]	–0.20 (0.841)	6.17 (0.85) vs. *6.26 (0.62)*
	Other Hispanic vs. *Non–Hispanic White*	–0.73 (0.40)	[–1.55, 0.08]	–1.83 (0.077)	5.53 (0.82) vs. *6.26 (0.62)*
	Non–Hispanic Black vs. *Non–Hispanic White*	–0.97 (0.41)	[–1.81, –0.12]	–2.34 (0.027)	5.29 (0.79) vs. *6.26 (0.62)*
	Non–Hispanic Asian vs. *Non–Hispanic White*	0.45 (0.53)	[–0.64, 1.54]	0.84 (0.406)	6.71 (0.85) vs. *6.26 (0.62)*
	Other Race and Multi–Racial vs. *Non–Hispanic White*	–2.78 (1.92)	[–6.71, 1.15]	–1.45 (0.159)	3.48 (2.08) vs. *6.26 (0.62)*
Marital status	Single or never married vs. *married or living with a partner*	1.44 (0.63)	[0.14, 2.73]	2.27 (0.031)	6.83 (0.95) vs. *5.39 (0.75)*
	Divorced or separated vs. *married or living with a partner*	0.06 (0.53)	[–1.03, 1.15]	0.11 (0.914)	5.45 (0.90) vs. *5.39 (0.75)*
	Widowed vs. *married or living with a partner*	–0.77 (0.49)	[–1.78, 0.24]	–1.56 (0.130)	4.62 (1.00) vs. *5.39 (0.75)*
Self–report general health condition	Poor vs. *excellent or very good*	–0.80 (1.03)	[–2.92, 1.31]	–0.77 (0.445)	5.06 (1.30) vs. *5.86 (0.70)*
	Good or fair vs. *excellent or very good*	–0.04 (0.42)	[–0.90, 0.82]	–0.10 (0.918)	5.81 (0.70) vs. *5.86 (0.70)*
Difficulties in thinking or remembering	Yes vs. *no*	–0.64 (0.50)	[–1.66, 0.38]	–1.28 (0.210)	5.25 (0.95) vs. *5.89 (0.74)*
Ever told you have a heart disease	Yes vs. *no*	–0.57 (0.66)	[–1.93, 0.78]	–0.87 (0.393)	5.29 (1.02) vs. *5.86 (0.71)*
Ever told you had a stroke	Yes vs. *no*	0.23 (0.83)	[–1.46, 1.93]	0.28 (0.780)	5.69 (1.09) vs. *5.46 (0.69)*
Ever told you have diabetes	Yes vs. *no*	–0.34 (0.46)	[–1.27, 0.60]	–0.74 (0.464)	5.41 (0.80) vs. *5.74 (0.89)*
Have smoked at least 100 cigarettes in life	Yes vs. *no*	–0.50 (0.26)	[–1.02, 0.02]	–1.95 (0.060)	5.32 (0.80) vs. *5.82 (0.84)*
Three criteria combination: AO + HBP + GLY	AO + HBP + GLY vs. *metabolically–healthy people*	–0.51 (0.33)	[–1.18, 0.15]	–1.57 (0.126)	5.32 (0.77) vs. *5.83 (0.88)*
Age at screening	Not applied (continuous covariate)	–0.10 (0.03)	[–0.15, –0.05]	–4.00 (< 0.001)	Not applied (continuous cov.)
Ratio of family income to the poverty level	Not applied (continuous covariate)	–0.07 (0.12)	[–0.32, 0.18]	–0.59 (0.560)	Not applied (continuous cov.)
Depression raw scores	Not applied (continuous covariate)	0.06 (0.03)	[0.00, 0.13]	1.95 (0.061)	Not applied (continuous cov.)

[a]The column labeled "estimated marginal means" shows each category's marginal means against the reference category's marginal mean. The reference categories and the marginal means of the reference categories are shown in italics. The significant results have been colored.

Table S31. Multiple linear regression model explaining the relationship between CERAD–DR and specific combination TRI + HDL + HBP after adjusting by sociodemographic characteristics and medical history, and estimated marginal means for each category of predictors and covariates[a]

**Model coefficient**	**Categories (for categorical predictor and covariates)**	*Beta* (*SE*)	95% CI	*t* (*p*)	Estimated marginal means[b]
Constant	Not applied (model intercept)	20.74 (1.32)	[17.86, 23.61]	15.71 (< 0.001)	Not applied (model intercept)
Education level	Up to 12th grade vs. *college graduate or above*	0.59 (0.29)	[–0.05, 1.23]	2.00 (0.069)	6.33 (0.70) vs. *5.74 (0.65)*
	High school graduate vs. *college graduate or above*	–1.50 (0.50)	[–2.59, –0.41]	–3.00 (0.011)	4.24 (0.75) vs. *5.74 (0.65)*
	Some college or AA degree vs. *college graduate or above*	0.04 (0.25)	[–0.51, 0.59]	0.15 (0.883)	5.78 (0.69) vs. *5.74 (0.65)*
Gender	Males vs. *females*	–1.02 (0.25)	[–1.56, –0.48]	–4.14 (0.001)	5.01 (0.64) vs. *6.03 (0.71)*
Race	Mexican American vs. *Non–Hispanic White*	–1.11 (0.62)	[–2.46, 0.23]	–1.80 (0.097)	4.61 (1.00) vs. *5.72 (0.57)*
	Other Hispanic vs. *Non–Hispanic White*	–0.17 (0.73)	[–1.76, 1.42]	–0.23 (0.819)	5.55 (0.81) vs. *5.72 (0.57)*
	Non–Hispanic Black vs. *Non–Hispanic White*	0.15 (0.57)	[–1.10, 1.39]	0.26 (0.801)	5.87 (0.78) vs. *5.72 (0.57)*
	Non–Hispanic Asian vs. *Non–Hispanic White*	0.15 (0.52)	[–0.99, 1.30]	0.29 (0.774)	5.87 (0.76) vs. *5.72 (0.57)*
	Other Race and Multi–Racial vs. *Non–Hispanic White*	No cases	No cases	No cases	No cases
Marital status	Single or never married vs. *married or living with a partner*	0.17 (0.85)	[–1.69, 2.02]	0.20 (0.847)	5.67 (1.01) vs. *5.50 (0.69)*
	Divorced or separated vs. *married or living with a partner*	0.76 (0.29)	[0.14, 1.38]	2.67 (0.020)	6.26 (0.69) vs. *5.50 (0.69)*
	Widowed vs. *married or living with a partner*	–0.85 (0.33)	[–1.56, –0.13]	–2.57 (0.024)	4.66 (0.70) vs. *5.50 (0.69)*
Self–report general health condition	Poor vs. *excellent or very good*	No cases	No cases	No cases	No cases
	Good or fair vs. *excellent or very good*	–0.48 (0.30)	[–1.14, 0.18]	–1.57 (0.142)	5.28 (0.61) vs. *5.76 (0.74)*
Difficulties in thinking or remembering	Yes vs. *no*	–0.51 (0.33)	[–1.22, 0.21]	–1.54 (0.148)	5.27 (0.69) vs. *5.78 (0.68)*
Ever told you have a heart disease	Yes vs. *no*	–0.89 (0.57)	[–2.14, 0.35]	–1.56 (0.144)	5.08 (0.88) vs. *5.97 (0.52)*
Ever told you had a stroke	Yes vs. *no*	–0.27 (0.45)	[–1.24, 0.71]	–0.60 (0.563)	5.39 (0.77) vs. *5.65 (0.62)*
Ever told you have diabetes	Yes vs. *no*	–0.87 (0.99)	[–3.02, 1.28]	–0.88 (0.397)	5.09 (0.94) vs. *5.96 (0.69)*
Have smoked at least 100 cigarettes in life	Yes vs. *no*	0.16 (0.36)	[–0.64, 0.95]	0.43 (0.674)	5.60 (0.62) vs. *5.44 (0.75)*
Three criteria combination: TRI + HDL + HBP	TRI + HDL + HBP vs. *metabolically–healthy people*	–0.80 (0.64)	[–2.19, 0.60]	–1.25 (0.236)	5.12 (0.89) vs. *5.92 (0.55)*
Age at screening	Not applied (continuous covariate)	–0.18 (0.02)	[–0.22, –0.14]	–9.71 (< 0.001)	Not applied (continuous cov.)
Ratio of family income to the poverty level	Not applied (continuous covariate)	–0.10 (0.09)	[–0.29, 0.09]	–1.16 (0.267)	Not applied (continuous cov.)
Depression raw scores	Not applied (continuous covariate)	0.04 (0.04)	[–0.05, 0.12]	0.96 (0.358)	Not applied (continuous cov.)

[a]The design–based covariance matrix is singular. The validity of results is uncertain. [b]The column labeled "estimated marginal means" shows each category's marginal means against the reference category's marginal mean. The reference categories and the marginal means of the reference categories are shown in italics. The significant results have been colored.

Table S32. Multiple linear regression model explaining the relationship between CERAD–DR and specific combination TRI + HDL + GLY after adjusting by sociodemographic characteristics and medical history, and estimated marginal means for each category of predictors and covariates[a]

**Model coefficient**	**Categories (for categorical predictor and covariates)**	*Beta* (*SE*)	95% CI	*t* (*p*)	Estimated marginal means[b]
Constant	Not applied (model intercept)	20.30 (1.22)	[17.68, 22.93]	16.71 (< 0.001)	Not applied (model intercept)
Education level	Up to 12th grade vs. *college graduate or above*	1.23 (0.25)	[0.69, 1.77]	4.94 (< 0.001)	7.01 (0.66) vs. *5.78 (0.68)*
	High school graduate vs. *college graduate or above*	–1.16 (0.50)	[–2.24, –0.09]	–2.34 (0.036)	4.62 (0.53) vs. *5.78 (0.68)*
	Some college or AA degree vs. *college graduate or above*	0.42 (0.24)	[–0.09, 0.93]	1.77 (0.101)	6.20 (0.64) vs. *5.78 (0.68)*
Gender	Males vs. *females*	–1.22 (0.29)	[–1.83, –0.60]	–4.27 (0.001)	5.29 (0.60) vs. *6.51 (0.62)*
Race	Mexican American vs. *Non–Hispanic White*	–1.41 (0.78)	[–3.09, 0.27]	–1.81 (0.093)	5.12 (0.83) vs. *6.53 (0.62)*
	Other Hispanic vs. *Non–Hispanic White*	–1.58 (0.73)	[–3.16, –0.01]	–2.17 (0.049)	4.95 (0.87) vs. *6.53 (0.62)*
	Non–Hispanic Black vs. *Non–Hispanic White*	0.31 (0.58)	[–0.93, 1.56]	0.54 (0.597)	6.84 (0.80) vs. *6.53 (0.62)*
	Non–Hispanic Asian vs. *Non–Hispanic White*	–0.48 (0.52)	[–1.60, 0.65]	–0.92 (0.376)	6.05 (0.62) vs. *6.53 (0.62)*
	Other Race and Multi–Racial vs. *Non–Hispanic White*	No cases	No cases	No cases	No cases
Marital status	Single or never married vs. *married or living with a partner*	0.82 (0.73)	[–0.75, 2.40]	1.13 (0.279)	6.53 (0.97) vs. *5.71 (0.57)*
	Divorced or separated vs. *married or living with a partner*	1.40 (0.26)	[0.84, 1.96]	5.44 (< 0.001)	7.11 (0.62) vs. *5.71 (0.57)*
	Widowed vs. *married or living with a partner*	–1.46 (0.49)	[–2.53, –0.39]	–2.94 (0.011)	4.25 (0.68) vs. *5.71 (0.57)*
Self–report general health condition	Poor vs. *excellent or very good*	No cases	No cases	No cases	No cases
	Good or fair vs. *excellent or very good*	–0.37 (0.32)	[–1.06, 0.33]	–1.14 (0.276)	5.72 (0.59) vs. *6.08 (0.64)*
Difficulties in thinking or remembering	Yes vs. *no*	–0.60 (0.38)	[–1.43, 0.23]	–1.56 (0.142)	5.60 (0.65) vs. *6.20 (0.60)*
Ever told you have a heart disease	Yes vs. *no*	–0.55 (0.60)	[–1.85, 0.75]	–0.91 (0.379)	5.63 (0.82) vs. *6.18 (0.47)*
Ever told you had a stroke	Yes vs. *no*	–0.05 (0.45)	[–1.02, 0.92]	–0.11 (0.912)	5.88 (0.77) vs. *5.93 (0.46)*
Ever told you have diabetes	Yes vs. *no*	0.03 (0.85)	[–1.81, 1.87]	0.04 (0.971)	5.92 (0.86) vs. *5.89 (0.58)*
Have smoked at least 100 cigarettes in life	Yes vs. *no*	–0.05 (0.33)	[–0.78, 0.67]	–0.16 (0.873)	5.87 (0.58) vs. *5.93 (0.66)*
Three criteria combination: TRI + HDL + GLY	TRI + HDL + GLY vs. *metabolically–healthy people*	–0.93 (0.31)	[–1.60, –0.27]	–3.04 (0.010)	5.43 (0.70) vs. *6.37 (0.52)*
Age at screening	Not applied (continuous covariate)	–0.17 (0.02)	[–0.21, –0.14]	–10.39 (< 0.001)	Not applied (continuous cov.)
Ratio of family income to the poverty level	Not applied (continuous covariate)	–0.13 (0.09)	[–0.33, 0.07]	–1.44 (0.173)	Not applied (continuous cov.)
Depression raw scores	Not applied (continuous covariate)	–0.07 (0.05)	[–0.17, 0.04]	–1.37 (0.193)	Not applied (continuous cov.)

[a]The design–based covariance matrix is singular. The validity of results is uncertain. [b]The column labeled "estimated marginal means" shows each category's marginal means against the reference category's marginal mean. The reference categories and the marginal means of the reference categories are shown in italics. The significant results have been colored.

Table S33. Multiple linear regression model explaining the relationship between CERAD–DR and specific combination TRI + HBP + GLY after adjusting by sociodemographic characteristics and medical history, and estimated marginal means for each category of predictors and covariates[a]

**Model coefficient**	**Categories (for categorical predictor and covariates)**	*Beta* (*SE*)	95% CI	*t* (*p*)	Estimated marginal means[b]
Constant	Not applied (model intercept)	18.89 (2.01)	[14.57, 23.21]	9.38 (< 0.001)	Not applied (model intercept)
Education level	Up to 12th grade vs. *college graduate or above*	0.52 (0.40)	[–0.33, 1.38]	1.31 (0.210)	7.09 (0.74) vs. *6.57 (0.67)*
	High school graduate vs. *college graduate or above*	–1.42 (0.61)	[–2.72, –0.12]	–2.34 (0.035)	5.15 (0.76) vs. *6.57 (0.67)*
	Some college or AA degree vs. *college graduate or above*	–0.01 (0.34)	[–0.73, 0.71]	–0.02 (0.981)	6.56 (0.75) vs. *6.57 (0.67)*
Gender	Males vs. *females*	–0.93 (0.31)	[–1.59, –0.26]	–2.97 (0.010)	5.88 (0.64) vs. *6.81 (0.76)*
Race	Mexican American vs. *Non–Hispanic White*	–1.01 (0.61)	[–2.31, 0.29]	–1.67 (0.118)	5.64 (0.91) vs. *6.65 (0.68)*
	Other Hispanic vs. *Non–Hispanic White*	–0.19 (0.75)	[–1.79, 1.42]	–0.25 (0.806)	6.47 (0.77) vs. *6.65 (0.68)*
	Non–Hispanic Black vs. *Non–Hispanic White*	–0.54 (0.79)	[–2.23, 1.15]	–0.68 (0.506)	6.11 (0.90) vs. *6.65 (0.68)*
	Non–Hispanic Asian vs. *Non–Hispanic White*	0.20 (0.48)	[–0.84, 1.24]	0.42 (0.684)	6.85 (0.85) vs. *6.65 (0.68)*
	Other Race and Multi–Racial vs. *Non–Hispanic White*	No cases	No cases	No cases	No cases
Marital status	Single or never married vs. *married or living with a partner*	0.05 (0.79)	[–1.65, 1.74]	0.06 (0.955)	6.51 (1.11) vs. *6.46 (0.71)*
	Divorced or separated vs. *married or living with a partner*	0.52 (0.39)	[–0.32, 1.35]	1.33 (0.205)	6.98 (0.71) vs. *6.46 (0.71)*
	Widowed vs. *married or living with a partner*	–1.02 (0.50)	[–2.10, 0.06]	–2.03 (0.062)	5.44 (0.71) vs. *6.46 (0.71)*
Self–report general health condition	Poor vs. *excellent or very good*	No cases	No cases	No cases	No cases
	Good or fair vs. *excellent or very good*	–0.40 (0.28)	[–1.01, 0.20]	–1.43 (0.174)	6.14 (0.65) vs. *6.55 (0.74)*
Difficulties in thinking or remembering	Yes vs. *no*	–0.54 (0.33)	[–1.23, 0.16]	–1.65 (0.122)	6.08 (0.74) vs. *6.61 (0.67)*
Ever told you have a heart disease	Yes vs. *no*	–0.91 (0.60)	[–2.20, 0.38]	–1.51 (0.152)	5.89 (0.93) vs. *6.80 (0.50)*
Ever told you had a stroke	Yes vs. *no*	0.42 (0.63)	[–0.94, 1.78]	0.66 (0.522)	6.55 (0.89) vs. *6.14 (0.58)*
Ever told you have diabetes	Yes vs. *no*	–0.01 (0.48)	[–1.03, 1.02]	–0.01 (0.991)	6.34 (0.73) vs. *6.35 (0.72)*
Have smoked at least 100 cigarettes in life	Yes vs. *no*	0.10 (0.40)	[–0.76, 0.95]	0.24 (0.814)	6.39 (0.62) vs. *6.30 (0.79)*
Three criteria combination: TRI + HBP + GLY	TRI + HBP + GLY vs. *metabolically–healthy people*	–0.22 (0.76)	[–1.84, 1.40]	–0.29 (0.775)	6.24 (1.02) vs. *6.46 (0.42)*
Age at screening	Not applied (continuous covariate)	–0.16 (0.03)	[–0.22, –0.10]	–5.64 (< 0.001)	Not applied (continuous cov.)
Ratio of family income to the poverty level	Not applied (continuous covariate)	–0.04 (0.10)	[–0.24, 0.17]	–0.40 (0.699)	Not applied (continuous cov.)
Depression raw scores	Not applied (continuous covariate)	0.02 (0.05)	[–0.09, 0.14]	0.40 (0.698)	Not applied (continuous cov.)

[a]The design–based covariance matrix is singular. The validity of results is uncertain. [b]The column labeled "estimated marginal means" shows each category's marginal means against the reference category's marginal mean. The reference categories and the marginal means of the reference categories are shown in italics. The significant results have been colored.

Table S34. Multiple linear regression model explaining the relationship between CERAD–DR and specific combination HDL + HBP + GLY after adjusting by sociodemographic characteristics and medical history, and estimated marginal means for each category of predictors and covariates[a]

**Model coefficient**	**Categories (for categorical predictor and covariates)**	*Beta* (*SE*)	95% CI	*t* (*p*)	Estimated marginal means[b]
Constant	Not applied (model intercept)	18.78 (1.54)	[15.51, 22.04]	12.19 (< 0.001)	Not applied (model intercept)
Education level	Up to 12th grade vs. *college graduate or above*	0.44 (0.61)	[–0.85, 1.73]	0.72 (0.479)	6.12 (0.58) vs. *5.68 (0.49)*
	High school graduate vs. *college graduate or above*	–1.41 (0.60)	[–2.68, –0.13]	–2.34 (0.032)	4.27 (0.76) vs. *5.68 (0.49)*
	Some college or AA degree vs. *college graduate or above*	0.03 (0.30)	[–0.61, 0.67]	0.09 (0.927)	5.70 (0.54) vs. *5.68 (0.49)*
Gender	Males vs. *females*	–0.98 (0.34)	[–1.71, –0.25]	–2.84 (0.012)	4.95 (0.41) vs. *5.93 (0.61)*
Race	Mexican American vs. *Non–Hispanic White*	–0.88 (0.62)	[–2.18, 0.43]	–1.43 (0.173)	4.93 (0.83) vs. *5.81 (0.45)*
	Other Hispanic vs. *Non–Hispanic White*	–0.85 (0.56)	[–2.03, 0.33]	–1.52 (0.148)	4.96 (0.48) vs. *5.81 (0.45)*
	Non–Hispanic Black vs. *Non–Hispanic White*	–0.11 (0.62)	[–1.42, 1.20]	–0.18 (0.859)	5.70 (0.72) vs. *5.81 (0.45)*
	Non–Hispanic Asian vs. *Non–Hispanic White*	–0.02 (0.41)	[–0.88, 0.84]	–0.05 (0.964)	5.79 (0.62) vs. *5.81 (0.45)*
	Other Race and Multi–Racial vs. *Non–Hispanic White*	No cases	No cases	No cases	No cases
Marital status	Single or never married vs. *married or living with a partner*	0.22 (0.74)	[–1.35, 1.79]	0.30 (0.771)	5.78 (0.92) vs. *5.56 (0.52)*
	Divorced or separated vs. *married or living with a partner*	0.75 (0.27)	[0.17, 1.33]	2.73 (0.015)	6.31 (0.54) vs. *5.56 (0.52)*
	Widowed vs. *married or living with a partner*	–1.45 (0.67)	[–2.87, –0.03]	–2.16 (0.046)	4.11 (0.64) vs. *5.56 (0.52)*
Self–report general health condition	Poor vs. *excellent or very good*	–2.19 (0.89)	[–4.08, –0.29]	–2.45 (0.026)	4.13 (0.62) vs. *6.31 (0.68)*
	Good or fair vs. *excellent or very good*	–0.43 (0.36)	[–1.19, 0.33]	–1.21 (0.244)	5.88 (0.66) vs. *6.31 (0.68)*
Difficulties in thinking or remembering	Yes vs. *no*	–0.31 (0.28)	[–0.89, 0.28]	–1.11 (0.283)	5.29 (0.53) vs. *5.59 (0.48)*
Ever told you have a heart disease	Yes vs. *no*	–0.74 (0.60)	[–2.01, 0.54]	–1.22 (0.240)	5.07 (0.77) vs. *5.81 (0.26)*
Ever told you had a stroke	Yes vs. *no*	0.12 (0.51)	[–0.95, 1.19]	0.24 (0.817)	5.50 (0.64) vs. *5.38 (0.44)*
Ever told you have diabetes	Yes vs. *no*	–1.14 (0.82)	[–2.89, 0.60]	–1.39 (0.183)	4.87 (0.68) vs. *6.01 (0.60)*
Have smoked at least 100 cigarettes in life	Yes vs. *no*	–0.03 (0.39)	[–0.85, 0.79]	–0.09 (0.933)	5.42 (0.38) vs. *5.46 (0.64)*
Three criteria combination: HDL + HBP + GLY	HDL + HBP + GLY vs. *metabolically–healthy people*	0.61 (0.55)	[–0.55, 1.77]	1.11 (0.284)	5.74 (0.69) vs. *5.14 (0.39)*
Age at screening	Not applied (continuous covariate)	–0.15 (0.02)	[–0.20, –0.11]	–7.12 (< 0.001)	Not applied (continuous cov.)
Ratio of family income to the poverty level	Not applied (continuous covariate)	–0.07 (0.08)	[–0.24, 0.10]	–0.88 (0.390)	Not applied (continuous cov.)
Depression raw scores	Not applied (continuous covariate)	0.01 (0.05)	[–0.11, 0.12]	0.13 (0.896)	Not applied (continuous cov.)

[a]The design–based covariance matrix is singular. The validity of results is uncertain. [b]The column labeled "estimated marginal means" shows each category's marginal means against the reference category's marginal mean. The reference categories and the marginal means of the reference categories are shown in italics. The significant results have been colored.

Table S35. Multiple linear regression model explaining the relationship between CERAD–DR and all combinations of three criteria after adjusting by sociodemographic characteristics and medical history, and estimated marginal means for each category of predictors and covariates

**Model coefficient**	**Categories (for categorical predictor and covariates)**	*Beta* (*SE*)	95% CI	*t* (*p*)	Estimated marginal means[a]
Constant	Not applied (model intercept)	11.76 (1.44)	[8.83, 14.68]	8.19 (< 0.001)	Not applied (model intercept)
Education level	Up to 12th grade vs. *college graduate or above*	0.00 (0.48)	[–0.99, 0.98]	–0.01 (0.992)	5.53 (0.59) vs. *5.54 (0.68)*
	High school graduate vs. *college graduate or above*	–0.15 (0.42)	[–1.00, 0.70]	–0.36 (0.724)	5.39 (0.60) vs. *5.54 (0.68)*
	Some college or AA degree vs. *college graduate or above*	0.61 (0.38)	[–0.18, 1.39]	1.58 (0.124)	6.14 (0.59) vs. *5.54 (0.68)*
Gender	Males vs. *females*	–0.20 (0.25)	[–0.71, 0.32]	–0.78 (0.441)	5.55 (0.55) vs. *5.75 (0.60)*
Race	Mexican American vs. *Non–Hispanic White*	–0.46 (0.44)	[–1.35, 0.43]	–1.06 (0.298)	5.81 (0.68) vs. *6.27 (0.45)*
	Other Hispanic vs. *Non–Hispanic White*	–0.62 (0.49)	[–1.62, 0.39]	–1.25 (0.221)	5.66 (0.67) vs. *6.27 (0.45)*
	Non–Hispanic Black vs. *Non–Hispanic White*	–0.73 (0.37)	[–1.49, 0.03]	–1.95 (0.060)	5.54 (0.63) vs. *6.27 (0.45)*
	Non–Hispanic Asian vs. *Non–Hispanic White*	0.70 (0.45)	[–0.21, 1.62]	1.56 (0.128)	6.98 (0.64) vs. *6.27 (0.45)*
	Other Race and Multi–Racial vs. *Non–Hispanic White*	–2.64 (1.15)	[–4.98, –0.30]	–2.29 (0.028)	3.63 (1.26) vs. *6.27 (0.45)*
Marital status	Single or never married vs. *married or living with a partner*	1.43 (0.51)	[0.39, 2.48]	2.79 (0.009)	6.82 (0.73) vs. *5.39 (0.45)*
	Divorced or separated vs. *married or living with a partner*	0.23 (0.40)	[–0.58, 1.05]	0.59 (0.562)	5.62 (0.70) vs. *5.39 (0.45)*
	Widowed vs. *married or living with a partner*	–0.63 (0.43)	[–1.51, 0.26]	–1.45 (0.158)	4.76 (0.68) vs. *5.39 (0.45)*
Self–report general health condition	Poor vs. *excellent or very good*	0.73 (0.80)	[–0.91, 2.37]	0.91 (0.369)	6.09 (0.86) vs. *5.35 (0.60)*
	Good or fair vs. *excellent or very good*	0.15 (0.33)	[–0.52, 0.82]	0.45 (0.654)	5.50 (0.51) vs. *5.35 (0.60)*
Difficulties in thinking or remembering	Yes vs. *no*	–0.99 (0.33)	[–1.67, –0.31]	–2.97 (0.006)	5.15 (0.66) vs. *6.14 (0.50)*
Ever told you have a heart disease	Yes vs. *no*	–1.12 (0.60)	[–2.35, 0.11]	–1.85 (0.073)	5.09 (0.78) vs. *6.21 (0.45)*
Ever told you had a stroke	Yes vs. *no*	0.35 (0.51)	[–0.70, 1.39]	0.68 (0.503)	5.82 (0.71) vs. *5.47 (0.51)*
Ever told you have diabetes	Yes vs. *no*	–0.70 (0.52)	[–1.76, 0.36]	–1.34 (0.188)	5.30 (0.61) vs. *6.00 (0.63)*
Have smoked at least 100 cigarettes in life	Yes vs. *no*	–0.33 (0.22)	[–0.77, 0.12]	–1.51 (0.141)	5.48 (0.54) vs. *5.81 (0.61)*
All combinations of three criteria	All combinations of three criteria vs. *metabolically–healthy people*	–0.68 (0.29)	[–1.28, –0.09]	–2.34 (0.026)	5.31 (0.51) vs. *5.99 (0.64)*
Age at screening	Not applied (continuous covariate)	–0.06 (0.02)	[–0.10, –0.02]	–3.02 (0.005)	Not applied (continuous cov.)
Ratio of family income to the poverty level	Not applied (continuous covariate)	–0.02 (0.10)	[–0.22, 0.18]	–0.20 (0.845)	Not applied (continuous cov.)
Depression raw scores	Not applied (continuous covariate)	0.00 (0.02)	[–0.04, 0.05]	0.15 (0.878)	Not applied (continuous cov.)

[a]The column labeled "estimated marginal means" shows each category's marginal means against the reference category's marginal mean. The reference categories and the marginal means of the reference categories are shown in italics. The significant results have been colored.

Table S36. Multiple linear regression model explaining the relationship between CERAD–DR and specific combination AO + TRI + HDL + HBP after adjusting by sociodemographic characteristics and medical history, and estimated marginal means for each category of predictors and covariates[a]

**Model coefficient**	**Categories (for categorical predictor and covariates)**	*Beta* (*SE*)	95% CI	*t* (*p*)	Estimated marginal means[b]
Constant	Not applied (model intercept)	21.00 (0.99)	[18.86, 23.14]	21.22 (< 0.001)	Not applied (model intercept)
Education level	Up to 12th grade vs. *college graduate or above*	0.49 (0.37)	[−0.31, 1.29]	1.33 (0.207)	3.80 (0.48) vs. *3.31 (0.58)*
	High school graduate vs. *college graduate or above*	−1.31 (0.50)	[−2.39, −0.22]	−2.60 (0.022)	2.01 (0.60) vs. *3.31 (0.58)*
	Some college or AA degree vs. *college graduate or above*	0.29 (0.35)	[−0.46, 1.04]	0.84 (0.415)	3.61 (0.69) vs. *3.31 (0.58)*
Gender	Males vs. *females*	−1.00 (0.30)	[−1.65, −0.36]	−3.35 (0.005)	2.68 (0.58) vs. *3.68 (0.55)*
Race	Mexican American vs. *Non–Hispanic White*	−1.38 (0.63)	[−2.73, −0.02]	−2.20 (0.047)	2.05 (0.78) vs. *3.42 (0.55)*
	Other Hispanic vs. *Non–Hispanic White*	−0.02 (0.67)	[−1.46, 1.42]	−0.03 (0.979)	3.41 (0.64) vs. *3.42 (0.55)*
	Non–Hispanic Black vs. *Non–Hispanic White*	0.38 (0.42)	[−0.51, 1.28]	0.92 (0.374)	3.81 (0.53) vs. *3.42 (0.55)*
	Non–Hispanic Asian vs. *Non–Hispanic White*	−0.19 (0.63)	[−1.55, 1.16]	−0.31 (0.763)	3.23 (0.91) vs. *3.42 (0.55)*
	Other Race and Multi–Racial vs. *Non–Hispanic White*	No cases	No cases	No cases	No cases
Marital status	Single or never married vs. *married or living with a partner*	0.22 (1.03)	[−2.01, 2.45]	0.22 (0.832)	3.24 (1.09) vs. *3.02 (0.52)*
	Divorced or separated vs. *married or living with a partner*	0.83 (0.32)	[0.14, 1.51]	2.60 (0.022)	3.84 (0.52) vs. *3.02 (0.52)*
	Widowed vs. *married or living with a partner*	−0.39 (0.41)	[−1.29, 0.50]	−0.95 (0.360)	2.63 (0.64) vs. *3.02 (0.52)*
Self–report general health condition	Poor vs. *excellent or very good*	−4.13 (1.23)	[−6.80, −1.47]	−3.35 (0.005)	0.54 (1.01) vs. *4.67 (0.72)*
	Good or fair vs. *excellent or very good*	−0.33 (0.31)	[−1.00, 0.35]	−1.04 (0.316)	4.34 (0.49) vs. *4.67 (0.72)*
Difficulties in thinking or remembering	Yes vs. *no*	−1.25 (0.42)	[−2.16, −0.34]	−2.96 (0.011)	2.56 (0.61) vs. *3.81 (0.55)*
Ever told you have a heart disease	Yes vs. *no*	−0.80 (0.53)	[−1.95, 0.35]	−1.50 (0.158)	2.78 (0.71) vs. *3.58 (0.48)*
Ever told you had a stroke	Yes vs. *no*	−3.03 (2.07)	[−7.49, 1.44]	−1.47 (0.167)	1.67 (1.34) vs. *4.70 (0.97)*
Ever told you have diabetes	Yes vs. *no*	−0.36 (1.55)	[−3.71, 3.00]	−0.23 (0.821)	3.00 (0.84) vs. *3.36 (1.05)*
Have smoked at least 100 cigarettes in life	Yes vs. *no*	−0.11 (0.34)	[−0.85, 0.63]	−0.33 (0.748)	3.13 (0.53) vs. *3.24 (0.61)*
Four criteria combination: AO + TRI + HDL + HBP	AO + TRI + HDL + HBP vs. *metabolically–healthy people*	−0.11 (0.47)	[−1.13, 0.91]	−0.24 (0.817)	3.13 (0.62) vs. *3.24 (0.57)*
Age at screening	Not applied (continuous covariate)	−0.19 (0.01)	[−0.22, −0.16]	−13.43 (< 0.001)	Not applied (continuous cov.)
Ratio of family income to the poverty level	Not applied (continuous covariate)	−0.09 (0.10)	[−0.29, 0.12]	−0.90 (0.385)	Not applied (continuous cov.)
Depression raw scores	Not applied (continuous covariate)	0.03 (0.05)	[−0.07, 0.13]	0.70 (0.494)	Not applied (continuous cov.)

[a]The design–based covariance matrix is singular. The validity of results is uncertain. [b]The column labeled "estimated marginal means" shows each category's marginal means against the reference category's marginal mean. The reference categories and the marginal means of the reference categories are shown in italics. The significant results have been colored.

Table S37. Multiple linear regression model explaining the relationship between CERAD–DR and specific combination AO + TRI + HDL + GLY after adjusting by sociodemographic characteristics and medical history, and estimated marginal means for each category of predictors and covariates[a]

**Model coefficient**	**Categories (for categorical predictor and covariates)**	*Beta* (*SE*)	95% CI	*t* (*p*)	Estimated marginal means[b]
Constant	Not applied (model intercept)	18.55 (1.22)	[16.00, 21.11]	15.19 (< 0.001)	Not applied (model intercept)
Education level	Up to 12th grade vs. *college graduate or above*	–0.34 (0.74)	[–1.87, 1.20]	–0.46 (0.654)	4.88 (0.62) vs. *5.21 (0.57)*
	High school graduate vs. *college graduate or above*	–1.05 (0.50)	[–2.10, 0.00]	–2.08 (0.051)	4.17 (0.69) vs. *5.21 (0.57)*
	Some college or AA degree vs. *college graduate or above*	–0.13 (0.47)	[–1.11, 0.84]	–0.29 (0.778)	5.08 (0.45) vs. *5.21 (0.57)*
Gender	Males vs. *females*	–1.05 (0.31)	[–1.69, –0.40]	–3.39 (0.003)	4.31 (0.48) vs. *5.36 (0.46)*
Race	Mexican American vs. *Non–Hispanic White*	–1.43 (0.41)	[–2.29, –0.57]	–3.47 (0.003)	3.95 (0.70) vs. *5.37 (0.52)*
	Other Hispanic vs. *Non–Hispanic White*	–0.80 (0.60)	[–2.05, 0.45]	–1.33 (0.198)	4.58 (0.47) vs. *5.37 (0.52)*
	Non–Hispanic Black vs. *Non–Hispanic White*	0.36 (0.47)	[–0.63, 1.34]	0.76 (0.457)	5.73 (0.60) vs. *5.37 (0.52)*
	Non–Hispanic Asian vs. *Non–Hispanic White*	0.43 (0.43)	[–0.47, 1.32]	0.99 (0.334)	5.80 (0.55) vs. *5.37 (0.52)*
	Other Race and Multi–Racial vs. *Non–Hispanic White*	–1.78 (0.58)	[–3.00, –0.57]	–3.06 (0.006)	3.59 (0.63) vs. *5.37 (0.52)*
Marital status	Single or never married vs. *married or living with a partner*	0.24 (0.65)	[–1.12, 1.60]	0.37 (0.717)	5.36 (0.77) vs. *5.12 (0.42)*
	Divorced or separated vs. *married or living with a partner*	–1.05 (0.63)	[–2.36, 0.26]	–1.67 (0.111)	4.07 (0.71) vs. *5.12 (0.42)*
	Widowed vs. *married or living with a partner*	–0.32 (0.49)	[–1.34, 0.69]	–0.66 (0.515)	4.80 (0.46) vs. *5.12 (0.42)*
Self–report general health condition	Poor vs. *excellent or very good*	–2.29 (0.87)	[–4.12, –0.47]	–2.64 (0.016)	3.31 (0.92) vs. *5.60 (0.38)*
	Good or fair vs. *excellent or very good*	–0.01 (0.28)	[–0.60, 0.58]	–0.04 (0.971)	5.59 (0.36) vs. *5.60 (0.38)*
Difficulties in thinking or remembering	Yes vs. *no*	–1.06 (0.42)	[–1.94, –0.18]	–2.53 (0.020)	4.30 (0.56) vs. *5.37 (0.41)*
Ever told you have a heart disease	Yes vs. *no*	–0.06 (0.54)	[–1.19, 1.07]	–0.10 (0.917)	4.81 (0.53) vs. *4.86 (0.51)*
Ever told you had a stroke	Yes vs. *no*	0.18 (0.35)	[–0.55, 0.92]	0.52 (0.607)	4.93 (0.52) vs. *4.74 (0.43)*
Ever told you have diabetes	Yes vs. *no*	–0.30 (0.42)	[–1.19, 0.58]	–0.72 (0.480)	4.68 (0.54) vs. *4.99 (0.44)*
Have smoked at least 100 cigarettes in life	Yes vs. *no*	–0.41 (0.38)	[–1.21, 0.39]	–1.07 (0.297)	4.63 (0.44) vs. *5.04 (0.53)*
Four criteria combination: AO + TRI + HDL + GLY	AO + TRI + HDL + GLY vs. *metabolically–healthy people*	–0.33 (0.41)	[–1.18, 0.52]	–0.82 (0.424)	4.67 (0.47) vs. *5.00 (0.50)*
Age at screening	Not applied (continuous covariate)	–0.16 (0.02)	[–0.19, –0.12]	–8.51 (< 0.001)	Not applied (continuous cov.)
Ratio of family income to the poverty level	Not applied (continuous covariate)	0.21 (0.08)	[0.04, 0.38]	2.63 (0.017)	Not applied (continuous cov.)
Depression raw scores	Not applied (continuous covariate)	–0.06 (0.03)	[–0.13, 0.01]	–1.84 (0.082)	Not applied (continuous cov.)

[a]The design–based covariance matrix is singular. The validity of results is uncertain. [b]The column labeled "estimated marginal means" shows each category's marginal means against the reference category's marginal mean. The reference categories and the marginal means of the reference categories are shown in italics. The significant results have been colored.

Table S38. Multiple linear regression model explaining the relationship between CERAD–DR and specific combination AO + TRI + HBP + GLY after adjusting by sociodemographic characteristics and medical history, and estimated marginal means for each category of predictors and covariates[a]

**Model coefficient**	**Categories (for categorical predictor and covariates)**	*Beta* (*SE*)	95% CI	*t* (*p*)	Estimated marginal means[b]
Constant	Not applied (model intercept)	19.28 (1.92)	[15.32, 23.23]	10.06 (< 0.001)	Not applied (model intercept)
Education level	Up to 12th grade vs. *college graduate or above*	–0.30 (0.54)	[–1.42, 0.81]	–0.57 (0.577)	7.25 (0.71) vs. *7.55 (0.66)*
	High school graduate vs. *college graduate or above*	–0.98 (0.47)	[–1.96, –0.01]	–2.08 (0.049)	6.57 (0.75) vs. *7.55 (0.66)*
	Some college or AA degree vs. *college graduate or above*	0.33 (0.46)	[–0.62, 1.28]	0.72 (0.478)	7.89 (0.79) vs. *7.55 (0.66)*
Gender	Males vs. *females*	–1.18 (0.32)	[–1.83, –0.53]	–3.73 (0.001)	6.73 (0.72) vs. *7.91 (0.64)*
Race	Mexican American vs. *Non–Hispanic White*	–0.92 (0.56)	[–2.07, 0.24]	–1.63 (0.116)	6.54 (0.79) vs. *7.45 (0.68)*
	Other Hispanic vs. *Non–Hispanic White*	–0.74 (0.58)	[–1.94, 0.45]	–1.28 (0.212)	6.71 (0.97) vs. *7.45 (0.68)*
	Non–Hispanic Black vs. *Non–Hispanic White*	0.60 (0.46)	[–0.35, 1.55]	1.31 (0.202)	8.05 (0.69) vs. *7.45 (0.68)*
	Non–Hispanic Asian vs. *Non–Hispanic White*	0.38 (0.39)	[–0.42, 1.18]	0.98 (0.337)	7.83 (0.62) vs. *7.45 (0.68)*
	Other Race and Multi–Racial vs. *Non–Hispanic White*	No cases	No cases	No cases	No cases
Marital status	Single or never married vs. *married or living with a partner*	0.64 (0.74)	[–0.88, 2.16]	0.87 (0.394)	7.47 (0.97) vs. *6.84 (0.70)*
	Divorced or separated vs. *married or living with a partner*	1.39 (0.52)	[0.33, 2.46]	2.70 (0.013)	8.23 (0.81) vs. *6.84 (0.70)*
	Widowed vs. *married or living with a partner*	–0.11 (0.50)	[–1.15, 0.93]	–0.21 (0.832)	6.73 (0.69) vs. *6.84 (0.70)*
Self–report general health condition	Poor vs. *excellent or very good*	0.78 (0.84)	[–0.95, 2.51]	0.93 (0.362)	8.01 (0.99) vs. *7.23 (0.68)*
	Good or fair vs. *excellent or very good*	–0.54 (0.36)	[–1.28, 0.20]	–1.50 (0.148)	6.70 (0.56) vs. *7.23 (0.68)*
Difficulties in thinking or remembering	Yes vs. *no*	–0.45 (0.62)	[–1.72, 0.83]	–0.72 (0.479)	7.09 (0.69) vs. *7.54 (0.77)*
Ever told you have a heart disease	Yes vs. *no*	0.14 (0.54)	[–0.98, 1.27]	0.26 (0.795)	7.39 (0.78) vs. *7.24 (0.65)*
Ever told you had a stroke	Yes vs. *no*	0.46 (0.97)	[–1.53, 2.46]	0.48 (0.638)	7.55 (1.05) vs. *7.09 (0.49)*
Ever told you have diabetes	Yes vs. *no*	–0.28 (0.57)	[–1.45, 0.89]	–0.49 (0.628)	7.18 (0.91) vs. *7.46 (0.46)*
Have smoked at least 100 cigarettes in life	Yes vs. *no*	–0.08 (0.33)	[–0.75, 0.60]	–0.23 (0.817)	7.28 (0.61) vs. *7.35 (0.74)*
Four criteria combination: AO + TRI + HBP + GLY	AO + TRI + HBP + GLY vs. *metabolically–healthy people*	0.11 (0.38)	[–0.68, 0.89]	0.29 (0.778)	7.37 (0.62) vs. *7.26 (0.75)*
Age at screening	Not applied (continuous covariate)	–0.16 (0.03)	[–0.22, –0.11]	–5.87 (< 0.001)	Not applied (continuous cov.)
Ratio of family income to the poverty level	Not applied (continuous covariate)	–0.05 (0.10)	[–0.25, 0.15]	–0.53 (0.601)	Not applied (continuous cov.)
Depression raw scores	Not applied (continuous covariate)	–0.05 (0.02)	[–0.08, –0.01]	–2.68 (0.013)	Not applied (continuous cov.)

[a]The design–based covariance matrix is singular. The validity of results is uncertain. [b]The column labeled "estimated marginal means" shows each category's marginal means against the reference category's marginal mean. The reference categories and the marginal means of the reference categories are shown in italics. The significant results have been colored.

Table S39. Multiple linear regression model explaining the relationship between CERAD–DR and specific combination AO + HDL + HBP + GLY after adjusting by sociodemographic characteristics and medical history, and estimated marginal means for each category of predictors and covariates[a]

**Model coefficient**	**Categories (for categorical predictor and covariates)**	*Beta* (*SE*)	95% CI	*t* (*p*)	Estimated marginal means[b]
Constant	Not applied (model intercept)	17.91 (1.39)	[14.98, 20.84]	12.85 (< 0.001)	Not applied (model intercept)
Education level	Up to 12th grade vs. *college graduate or above*	–0.49 (0.43)	[–1.40, 0.42]	–1.12 (0.276)	6.61 (0.79) vs. *7.10 (0.67)*
	High school graduate vs. *college graduate or above*	–1.51 (0.50)	[–2.57, –0.45]	–2.99 (0.008)	5.59 (0.73) vs. *7.10 (0.67)*
	Some college or AA degree vs. *college graduate or above*	–0.30 (0.33)	[–1.00, 0.39]	–0.91 (0.375)	6.80 (0.71) vs. *7.10 (0.67)*
Gender	Males vs. *females*	–1.11 (0.25)	[–1.64, –0.58]	–4.37 (< 0.001)	5.97 (0.72) vs. *7.08 (0.65)*
Race	Mexican American vs. *Non–Hispanic White*	–0.93 (0.40)	[–1.77, –0.10]	–2.34 (0.031)	6.13 (0.86) vs. *7.06 (0.71)*
	Other Hispanic vs. *Non–Hispanic White*	–1.44 (0.74)	[–2.99, 0.11]	–1.95 (0.067)	5.62 (0.69) vs. *7.06 (0.71)*
	Non–Hispanic Black vs. *Non–Hispanic White*	–0.37 (0.42)	[–1.27, 0.52]	–0.88 (0.390)	6.68 (0.73) vs. *7.06 (0.71)*
	Non–Hispanic Asian vs. *Non–Hispanic White*	0.06 (0.64)	[–1.29, 1.42]	0.10 (0.925)	7.12 (1.03) vs. *7.06 (0.71)*
	Other Race and Multi–Racial vs. *Non–Hispanic White*	–0.51 (0.98)	[–2.57, 1.54]	–0.53 (0.606)	6.54 (0.89) vs. *7.06 (0.71)*
Marital status	Single or never married vs. *married or living with a partner*	–0.79 (0.62)	[–2.09, 0.52]	–1.27 (0.221)	6.06 (1.09) vs. 6.85 (0.57)
	Divorced or separated vs. *married or living with a partner*	0.57 (0.33)	[–0.11, 1.26]	1.75 (0.097)	7.42 (0.68) vs. 6.85 (0.57)
	Widowed vs. *married or living with a partner*	–1.09 (0.64)	[–2.43, 0.25]	–1.70 (0.106)	5.76 (0.66) vs. 6.85 (0.57)
Self–report general health condition	Poor vs. *excellent or very good*	1.10 (1.19)	[–1.40, 3.60]	0.92 (0.368)	7.32 (1.22) vs. *6.22 (0.69)*
	Good or fair vs. *excellent or very good*	–0.19 (0.36)	[–0.94, 0.57]	–0.52 (0.609)	6.03 (0.55) vs. *6.22 (0.69)*
Difficulties in thinking or remembering	Yes vs. *no*	–0.42 (0.29)	[–1.04, 0.20]	–1.43 (0.169)	6.31 (0.72) vs. *6.74 (0.65)*
Ever told you have a heart disease	Yes vs. *no*	–0.58 (0.48)	[–1.60, 0.43]	–1.21 (0.242)	6.23 (0.75) vs. *6.82 (0.67)*
Ever told you had a stroke	Yes vs. *no*	1.33 (0.81)	[–0.36, 3.02]	1.65 (0.116)	7.19 (1.03) vs. *5.86 (0.40)*
Ever told you have diabetes	Yes vs. *no*	–0.35 (0.47)	[–1.34, 0.64]	–0.75 (0.464)	6.35 (0.69) vs. *6.70 (0.73)*
Have smoked at least 100 cigarettes in life	Yes vs. *no*	0.11 (0.32)	[–0.57, 0.78]	0.34 (0.741)	6.58 (0.69) vs. *6.47 (0.69)*
Four criteria combination: AO + HDL + HBP + GLY	AO + HDL + HBP + GLY vs. *metabolically–healthy people*	–0.33 (0.39)	[–1.15, 0.49]	–0.84 (0.412)	6.36 (0.63) vs. *6.69 (0.76)*
Age at screening	Not applied (continuous covariate)	–0.14 (0.02)	[–0.18, –0.10]	–7.45 (< 0.001)	Not applied (continuous cov.)
Ratio of family income to the poverty level	Not applied (continuous covariate)	0.01 (0.08)	[–0.15, 0.18]	0.18 (0.863)	Not applied (continuous cov.)
Depression raw scores	Not applied (continuous covariate)	–0.01 (0.04)	[–0.09, 0.07]	–0.32 (0.756)	Not applied (continuous cov.)

[a]The design–based covariance matrix is singular. The validity of results is uncertain. [b]The column labeled "estimated marginal means" shows each category's marginal means against the reference category's marginal mean. The reference categories and the marginal means of the reference categories are shown in italics. The significant results have been colored.

Table S40. Multiple linear regression model explaining the relationship between CERAD–DR and specific combination TRI + HDL + HBP + GLY after adjusting by sociodemographic characteristics and medical history, and estimated marginal means for each category of predictors and covariates[a]

**Model coefficient**	**Categories (for categorical predictor and covariates)**	*Beta (SE)*	95% CI	*t (p)*	Estimated marginal means[b]
Constant	Not applied (model intercept)	20.61 (1.39)	[17.60, 23.63]	14.78 (< 0.001)	Not applied (model intercept)
Education level	Up to 12th grade vs. *college graduate or above*	0.76 (0.36)	[–0.01, 1.53]	2.13 (0.053)	7.17 (0.75) vs. *6.41 (0.75)*
	High school graduate vs. *college graduate or above*	–1.41 (0.57)	[–2.63, –0.19]	–2.49 (0.027)	5.00 (0.60) vs. *6.41 (0.75)*
	Some college or AA degree vs. *college graduate or above*	0.09 (0.30)	[–0.56, 0.74]	0.28 (0.781)	6.50 (0.70) vs. *6.41 (0.75)*
Gender	Males vs. *females*	–1.10 (0.30)	[–1.75, –0.46]	–3.69 (0.003)	5.72 (0.63) vs. *6.82 (0.72)*
Race	Mexican American vs. *Non–Hispanic White*	–1.09 (0.70)	[–2.60, 0.41]	–1.57 (0.140)	5.60 (0.71) vs. *6.69 (0.63)*
	Other Hispanic vs. *Non–Hispanic White*	–0.18 (0.78)	[–1.87, 1.50]	–0.24 (0.817)	6.51 (0.84) vs. *6.69 (0.63)*
	Non–Hispanic Black vs. *Non–Hispanic White*	0.32 (0.60)	[–0.97, 1.61]	0.54 (0.600)	7.01 (0.88) vs. *6.69 (0.63)*
	Non–Hispanic Asian vs. *Non–Hispanic White*	–0.25 (0.45)	[–1.22, 0.71]	–0.57 (0.580)	6.44 (0.79) vs. *6.69 (0.63)*
	Other Race and Multi–Racial vs. *Non–Hispanic White*	–1.34 (1.07)	[–3.65, 0.98]	–1.25 (0.235)	5.36 (1.24) vs. *6.69 (0.63)*
Marital status	Single or never married vs. *married or living with a partner*	0.21 (0.89)	[–1.71, 2.14]	0.24 (0.815)	6.50 (1.10) vs. *6.29 (0.67)*
	Divorced or separated vs. *married or living with a partner*	0.85 (0.29)	[0.23, 1.48]	2.95 (0.011)	7.14 (0.66) vs. *6.29 (0.67)*
	Widowed vs. *married or living with a partner*	–1.14 (0.51)	[–2.24, –0.04]	–2.24 (0.043)	5.15 (0.77) vs. *6.29 (0.67)*
Self–report general health condition	Poor vs. *excellent or very good*	–1.16 (1.46)	[–4.31, 1.99]	–0.79 (0.441)	5.65 (1.46) vs. *6.81 (0.59)*
	Good or fair vs. *excellent or very good*	–0.47 (0.29)	[–1.09, 0.14]	–1.65 (0.122)	6.34 (0.53) vs. *6.81 (0.59)*
Difficulties in thinking or remembering	Yes vs. *no*	–0.45 (0.35)	[–1.21, 0.30]	–1.30 (0.217)	6.04 (0.72) vs. *6.50 (0.65)*
Ever told you have a heart disease	Yes vs. *no*	–0.63 (0.61)	[–1.94, 0.69]	–1.03 (0.322)	5.96 (0.90) vs. *6.58 (0.50)*
Ever told you had a stroke	Yes vs. *no*	–0.24 (0.44)	[–1.20, 0.71]	–0.55 (0.590)	6.15 (0.81) vs. *6.39 (0.57)*
Ever told you have diabetes	Yes vs. *no*	0.36 (0.51)	[–0.75, 1.46]	0.70 (0.497)	6.45 (0.72) vs. *6.09 (0.70)*
Have smoked at least 100 cigarettes in life	Yes vs. *no*	0.12 (0.38)	[–0.70, 0.94]	0.32 (0.757)	6.33 (0.66) vs. *6.21 (0.71)*
Four criteria combination: TRI + HDL + HBP + GLY	TRI + HDL + HBP + GLY vs. *metabolically–healthy people*	0.38 (0.97)	[–1.72, 2.49]	0.39 (0.700)	6.46 (0.82) vs. *6.08 (0.83)*
Age at screening	Not applied (continuous covariate)	–0.18 (0.02)	[–0.22, –0.14]	–9.38 (< 0.001)	Not applied (continuous cov.)
Ratio of family income to the poverty level	Not applied (continuous covariate)	–0.08 (0.09)	[–0.28, 0.12]	–0.87 (0.400)	Not applied (continuous cov.)
Depression raw scores	Not applied (continuous covariate)	0.00 (0.06)	[–0.12, 0.12]	0.02 (0.985)	Not applied (continuous cov.)

[a]The design–based covariance matrix is singular. The validity of results is uncertain. [b]The column labeled "estimated marginal means" shows each category's marginal means against the reference category's marginal mean. The reference categories and the marginal means of the reference categories are shown in italics. The significant results have been colored.

Table S41. Multiple linear regression model explaining the relationship between CERAD–DR and all combinations of four criteria after adjusting by sociodemographic characteristics and medical history, and estimated marginal means for each category of predictors and covariates

**Model coefficient**	**Categories (for categorical predictor and covariates)**	*Beta* (*SE*)	95% CI	*t* (*p*)	Estimated marginal means[a]
Constant	Not applied (model intercept)	16.05 (1.55)	[12.88, 19.23]	10.38 (< 0.001)	Not applied (model intercept)
Education level	Up to 12th grade vs. *college graduate or above*	–0.91 (0.51)	[–1.96, 0.13]	–1.79 (0.084)	4.95 (0.64) vs. *5.86 (0.56)*
	High school graduate vs. *college graduate or above*	–0.83 (0.38)	[–1.61, –0.06]	–2.20 (0.037)	5.03 (0.67) vs. *5.86 (0.56)*
	Some college or AA degree vs. *college graduate or above*	0.20 (0.44)	[–0.71, 1.10]	0.45 (0.660)	6.06 (0.59) vs. *5.86 (0.56)*
Gender	Males vs. *females*	–0.84 (0.24)	[–1.33, –0.35]	–3.52 (0.002)	5.05 (0.60) vs. *5.89 (0.53)*
Race	Mexican American vs. *Non–Hispanic White*	–0.70 (0.42)	[–1.56, 0.17]	–1.65 (0.111)	5.30 (0.67) vs. *5.99 (0.68)*
	Other Hispanic vs. *Non–Hispanic White*	–1.50 (0.57)	[–2.66, –0.33]	–2.64 (0.014)	4.50 (0.71) vs. *5.99 (0.68)*
	Non–Hispanic Black vs. *Non–Hispanic White*	0.26 (0.32)	[–0.41, 0.92]	0.79 (0.438)	6.25 (0.58) vs. *5.99 (0.68)*
	Non–Hispanic Asian vs. *Non–Hispanic White*	0.61 (0.41)	[–0.23, 1.44]	1.49 (0.147)	6.60 (0.65) vs. *5.99 (0.68)*
	Other Race and Multi–Racial vs. *Non–Hispanic White*	–1.78 (0.38)	[–2.57, –1.00]	–4.67 (< 0.001)	4.21 (0.46) vs. *5.99 (0.68)*
Marital status	Single or never married vs. *married or living with a partner*	–0.24 (0.57)	[–1.41, 0.93]	–0.42 (0.681)	5.35 (0.78) vs. *5.59 (0.47)*
	Divorced or separated vs. *married or living with a partner*	–0.26 (0.43)	[–1.15, 0.63]	–0.60 (0.552)	5.32 (0.75) vs. *5.59 (0.47)*
	Widowed vs. *married or living with a partner*	0.05 (0.32)	[–0.61, 0.71]	0.16 (0.872)	5.64 (0.48) vs. *5.59 (0.47)*
Self–report general health condition	Poor vs. *excellent or very good*	0.91 (0.62)	[–0.37, 2.19]	1.46 (0.155)	6.11 (0.75) vs. *5.20 (0.67)*
	Good or fair vs. *excellent or very good*	–0.08 (0.33)	[–0.77, 0.60]	–0.25 (0.804)	5.11 (0.45) vs. *5.20 (0.67)*
Difficulties in thinking or remembering	Yes vs. *no*	–1.62 (0.43)	[–2.49, –0.74]	–3.79 (0.001)	4.67 (0.71) vs. *6.28 (0.45)*
Ever told you have a heart disease	Yes vs. *no*	–0.12 (0.42)	[–0.98, 0.74]	–0.28 (0.779)	5.41 (0.60) vs. *5.53 (0.59)*
Ever told you had a stroke	Yes vs. *no*	–0.03 (0.66)	[–1.38, 1.33]	–0.04 (0.968)	5.46 (0.83) vs. *5.49 (0.37)*
Ever told you have diabetes	Yes vs. *no*	–0.27 (0.33)	[–0.96, 0.41]	–0.83 (0.416)	5.34 (0.62) vs. *5.61 (0.54)*
Have smoked at least 100 cigarettes in life	Yes vs. *no*	–0.16 (0.28)	[–0.73, 0.41]	–0.57 (0.572)	5.39 (0.58) vs. *5.55 (0.56)*
All combinations of four criteria	All combinations of four criteria vs. *metabolically–healthy people*	–0.38 (0.30)	[–0.99, 0.22]	–1.29 (0.207)	5.28 (0.56) vs. *5.66 (0.59)*
Age at screening	Not applied (continuous covariate)	–0.12 (0.02)	[–0.17, –0.07]	–5.11 (< 0.001)	Not applied (continuous cov.)
Ratio of family income to the poverty level	Not applied (continuous covariate)	0.11 (0.07)	[–0.04, 0.27]	1.49 (0.147)	Not applied (continuous cov.)
Depression raw scores	Not applied (continuous covariate)	–0.03 (0.02)	[–0.07, 0.00]	–2.07 (0.048)	Not applied (continuous cov.)

[a]The column labeled "estimated marginal means" shows each category's marginal means against the reference category's marginal mean. The reference categories and the marginal means of the reference categories are shown in italics. The significant results have been colored.

Table S42. Multiple linear regression model explaining the relationship between CERAD–DR and combination of five criteria after adjusting by sociodemographic characteristics and medical history, and estimated marginal means for each category of predictors and covariates[a]

**Model coefficient**	**Categories (for categorical predictor and covariates)**	*Beta* (*SE*)	95% CI	*t* (*p*)	Estimated marginal means[b]
Constant	Not applied (model intercept)	13.92 (2.20)	[9.32, 18.52]	6.31 (< 0.001)	Not applied (model intercept)
Education level	Up to 12th grade vs. *college graduate or above*	–0.30 (0.87)	[–2.12, 1.51]	–0.35 (0.732)	7.31 (0.78) vs. *7.62 (0.75)*
	High school graduate vs. *college graduate or above*	–2.12 (0.54)	[–3.26, –0.99]	–3.90 (0.001)	5.49 (0.78) vs. *7.62 (0.75)*
	Some college or AA degree vs. *college graduate or above*	–0.54 (0.31)	[–1.18, 0.11]	–1.74 (0.097)	7.08 (0.68) vs. *7.62 (0.75)*
Gender	Males vs. *females*	–0.60 (0.42)	[–1.47, 0.27]	–1.43 (0.168)	6.58 (0.63) vs. *7.17 (0.68)*
Race	Mexican American vs. *Non–Hispanic White*	–0.34 (0.47)	[–1.32, 0.64]	–0.72 (0.481)	7.00 (0.68) vs. *7.33 (0.44)*
	Other Hispanic vs. *Non–Hispanic White*	–2.50 (0.94)	[–4.47, –0.53]	–2.65 (0.015)	4.83 (1.01) vs. *7.33 (0.44)*
	Non–Hispanic Black vs. *Non–Hispanic White*	–0.05 (0.81)	[–1.74, 1.64]	–0.06 (0.954)	7.29 (0.96) vs. *7.33 (0.44)*
	Non–Hispanic Asian vs. *Non–Hispanic White*	0.59 (0.56)	[–0.57, 1.75]	1.06 (0.303)	7.92 (0.81) vs. *7.33 (0.44)*
	Other Race and Multi–Racial vs. *Non–Hispanic White*	No cases	No cases	No cases	No cases
Marital status	Single or never married vs. *married or living with a partner*	0.44 (0.72)	[–1.06, 1.94]	0.61 (0.550)	7.50 (0.60) vs. *7.07 (0.72)*
	Divorced or separated vs. *married or living with a partner*	0.33 (0.43)	[–0.57, 1.23]	0.77 (0.451)	7.40 (0.91) vs. *7.07 (0.72)*
	Widowed vs. *married or living with a partner*	–1.54 (0.60)	[–2.80, –0.28]	–2.55 (0.019)	5.53 (0.83) vs. *7.07 (0.72)*
Self–report general health condition	Poor vs. *excellent or very good*	1.45 (1.21)	[–1.08, 3.98]	1.20 (0.245)	8.17 (1.37) vs. *6.72 (0.40)*
	Good or fair vs. *excellent or very good*	–0.99 (0.41)	[–1.85, –0.13]	–2.40 (0.026)	5.73 (0.45) vs. *6.72 (0.40)*
Difficulties in thinking or remembering	Yes vs. *no*	0.36 (0.58)	[–0.86, 1.57]	0.61 (0.548)	7.05 (0.58) vs. *6.70 (0.78)*
Ever told you have a heart disease	Yes vs. *no*	0.18 (0.47)	[–0.79, 1.15]	0.38 (0.707)	6.96 (0.72) vs. *6.79 (0.60)*
Ever told you had a stroke	Yes vs. *no*	–0.78 (0.61)	[–2.06, 0.50]	–1.27 (0.219)	6.48 (0.86) vs. *7.26 (0.46)*
Ever told you have diabetes	Yes vs. *no*	–0.23 (0.71)	[–1.72, 1.26]	–0.32 (0.750)	6.76 (0.81) vs. *6.99 (0.60)*
Have smoked at least 100 cigarettes in life	Yes vs. *no*	–0.07 (0.47)	[–1.05, 0.92]	–0.14 (0.890)	6.84 (0.72) vs. *6.91 (0.61)*
Combination of five criteria	AO + TRI + HDL + HBP + GLY vs. *metabolically–healthy people*	0.16 (0.28)	[–0.42, 0.73]	0.56 (0.582)	6.95 (0.57) vs. *6.80 (0.69)*
Age at screening	Not applied (continuous covariate)	–0.08 (0.03)	[–0.15, –0.01]	–2.24 (0.036)	Not applied (continuous cov.)
Ratio of family income to the poverty level	Not applied (continuous covariate)	–0.03 (0.11)	[–0.26, 0.20]	–0.28 (0.783)	Not applied (continuous cov.)
Depression raw scores	Not applied (continuous covariate)	0.01 (0.04)	[–0.06, 0.08]	0.31 (0.757)	Not applied (continuous cov.)

[a]The design–based covariance matrix is singular. The validity of results is uncertain. [b]The column labeled "estimated marginal means" shows each category's marginal means against the reference category's marginal mean. The reference categories and the marginal means of the reference categories are shown in italics. The significant results have been colored.

Table S43. Multiple linear regression model explaining the relationship between CERAD–DR and all combination with abdominal obesity after adjusting by sociodemographic characteristics and medical history, and estimated marginal means for each category of predictors and covariates

**Model coefficient**	**Categories (for categorical predictor and covariates)**	*Beta* (*SE*)	95% CI	*t* (*p*)	Estimated marginal means[a]
Constant	Not applied (model intercept)	12.25 (1.18)	[9.85, 14.66]	10.39 (< 0.001)	Not applied (model intercept)
Education level	Up to 12th grade vs. *college graduate or above*	−0.49 (0.41)	[−1.33, 0.35]	−1.20 (0.241)	5.95 (0.38) vs. *6.45 (0.43)*
	High school graduate vs. *college graduate or above*	−0.30 (0.37)	[−1.06, 0.47]	−0.79 (0.434)	6.15 (0.41) vs. *6.45 (0.43)*
	Some college or AA degree vs. *college graduate or above*	0.49 (0.28)	[−0.09, 1.06]	1.72 (0.095)	6.93 (0.39) vs. *6.45 (0.43)*
Gender	Males vs. *females*	−0.29 (0.19)	[−0.69, 0.10]	−1.51 (0.140)	6.22 (0.34) vs. *6.52 (0.38)*
Race	Mexican American vs. *Non–Hispanic White*	−0.43 (0.36)	[−1.17, 0.31]	−1.20 (0.241)	6.44 (0.45) vs. *6.88 (0.39)*
	Other Hispanic vs. *Non–Hispanic White*	−1.07 (0.41)	[−1.90, −0.24]	−2.62 (0.014)	5.81 (0.43) vs. *6.88 (0.39)*
	Non–Hispanic Black vs. *Non–Hispanic White*	−0.47 (0.31)	[−1.10, 0.15]	−1.54 (0.133)	6.40 (0.45) vs. *6.88 (0.39)*
	Non–Hispanic Asian vs. *Non–Hispanic White*	0.89 (0.36)	[0.15, 1.62]	2.46 (0.020)	7.76 (0.44) vs. *6.88 (0.39)*
	Other Race and Multi–Racial vs. *Non–Hispanic White*	−1.95 (0.44)	[−2.85, −1.05]	−4.40 (< 0.001)	4.93 (0.49) vs. *6.88 (0.39)*
Marital status	Single or never married vs. *married or living with a partner*	1.06 (0.45)	[0.15, 1.98]	2.36 (0.025)	7.23 (0.49) vs. *6.16 (0.33)*
	Divorced or separated vs. *married or living with a partner*	0.00 (0.24)	[−0.49, 0.49]	0.01 (0.993)	6.17 (0.45) vs. *6.16 (0.33)*
	Widowed vs. *married or living with a partner*	−0.24 (0.31)	[−0.86, 0.38]	−0.79 (0.436)	5.92 (0.42) vs. *6.16 (0.33)*
Self–report general health condition	Poor vs. *excellent or very good*	1.76 (0.71)	[0.31, 3.20]	2.48 (0.019)	7.51 (0.64) vs. *5.75 (0.39)*
	Good or fair vs. *excellent or very good*	0.10 (0.27)	[−0.45, 0.66]	0.38 (0.703)	5.85 (0.34) vs. *5.75 (0.39)*
Difficulties in thinking or remembering	Yes vs. *no*	−1.13 (0.27)	[−1.68, −0.58]	−4.17 (< 0.001)	5.81 (0.42) vs. *6.94 (0.32)*
Ever told you have a heart disease	Yes vs. *no*	−0.08 (0.37)	[−0.84, 0.68]	−0.21 (0.831)	6.33 (0.47) vs. *6.41 (0.31)*
Ever told you had a stroke	Yes vs. *no*	0.24 (0.40)	[−0.56, 1.05]	0.62 (0.541)	6.49 (0.49) vs. *6.25 (0.29)*
Ever told you have diabetes	Yes vs. *no*	−0.43 (0.36)	[−1.16, 0.30]	−1.21 (0.236)	6.15 (0.39) vs. *6.59 (0.40)*
Have smoked at least 100 cigarettes in life	Yes vs. *no*	−0.26 (0.22)	[−0.71, 0.19]	−1.18 (0.246)	6.24 (0.30) vs. *6.50 (0.42)*
All combinations with abdominal obesity	Combinations with AO vs. *metabolically–healthy people*	−0.58 (0.26)	[−1.11, −0.06]	−2.26 (0.031)	6.08 (0.33) vs. *6.66 (0.41)*
Age at screening	Not applied (continuous covariate)	−0.07 (0.02)	[−0.11, −0.04]	−4.34 (< 0.001)	Not applied (continuous cov.)
Ratio of family income to the poverty level	Not applied (continuous covariate)	0.04 (0.08)	[−0.12, 0.21]	0.56 (0.582)	Not applied (continuous cov.)
Depression raw scores	Not applied (continuous covariate)	−0.01 (0.02)	[−0.05, 0.03]	−0.36 (0.724)	Not applied (continuous cov.)

[a]The column labeled "estimated marginal means" shows each category's marginal means against the reference category's marginal mean. The reference categories and the marginal means of the reference categories are shown in italics. The significant results have been colored.

Table S44. Multiple linear regression model explaining the relationship between CERAD–DR and all combinations without abdominal obesity after adjusting by sociodemographic characteristics and medical history, and estimated marginal means for each category of predictors and covariates[a]

**Model coefficient**	**Categories (for categorical predictor and covariates)**	*Beta* (*SE*)	95% CI	*t* (*p*)	Estimated marginal means[b]
Constant	Not applied (model intercept)	16.83 (1.62)	[13.45, 20.20]	10.41 (< 0.001)	Not applied (model intercept)
Education level	Up to 12th grade vs. *college graduate or above*	0.78 (0.64)	[–0.55, 2.11]	1.22 (0.237)	5.90 (0.63) vs. *5.12 (0.66)*
	High school graduate vs. *college graduate or above*	–1.13 (0.52)	[–2.22, –0.04]	–2.17 (0.042)	3.99 (0.76) vs. *5.12 (0.66)*
	Some college or AA degree vs. *college graduate or above*	0.18 (0.29)	[–0.44, 0.79]	0.60 (0.554)	5.30 (0.68) vs. *5.12 (0.66)*
Gender	Males vs. *females*	–0.88 (0.31)	[–1.52, –0.24]	–2.88 (0.009)	4.64 (0.51) vs. *5.52 (0.72)*
Race	Mexican American vs. *Non–Hispanic White*	–0.93 (0.61)	[–2.19, 0.33]	–1.54 (0.140)	4.87 (0.87) vs. *5.80 (0.46)*
	Other Hispanic vs. *Non–Hispanic White*	–1.78 (0.55)	[–2.92, –0.65]	–3.27 (0.004)	4.02 (0.76) vs. *5.80 (0.46)*
	Non–Hispanic Black vs. *Non–Hispanic White*	–0.05 (0.64)	[–1.38, 1.27]	–0.09 (0.933)	5.74 (0.87) vs. *5.80 (0.46)*
	Non–Hispanic Asian vs. *Non–Hispanic White*	–0.16 (0.45)	[–1.11, 0.78]	–0.36 (0.723)	5.64 (0.67) vs. *5.80 (0.46)*
	Other Race and Multi–Racial vs. *Non–Hispanic White*	–1.40 (0.45)	[–2.34, –0.46]	–3.12 (0.005)	4.40 (0.72) vs. *5.80 (0.46)*
Marital status	Single or never married vs. *married or living with a partner*	0.50 (0.70)	[–0.96, 1.96]	0.71 (0.485)	5.65 (0.90) vs. *5.15 (0.62)*
	Divorced or separated vs. *married or living with a partner*	0.83 (0.32)	[0.17, 1.49]	2.61 (0.017)	5.98 (0.64) vs. *5.15 (0.62)*
	Widowed vs. *married or living with a partner*	–1.62 (0.67)	[–3.02, –0.22]	–2.42 (0.025)	3.53 (0.85) vs. *5.15 (0.62)*
Self–report general health condition	Poor vs. *excellent or very good*	–0.34 (0.92)	[–2.26, 1.59]	–0.37 (0.718)	4.97 (0.89) vs. *5.31 (0.72)*
	Good or fair vs. *excellent or very good*	–0.35 (0.37)	[–1.13, 0.43]	–0.93 (0.365)	4.96 (0.58) vs. *5.31 (0.72)*
Difficulties in thinking or remembering	Yes vs. *no*	–0.55 (0.31)	[–1.20, 0.10]	–1.75 (0.095)	4.80 (0.69) vs. *5.35 (0.55)*
Ever told you have a heart disease	Yes vs. *no*	–0.96 (0.63)	[–2.28, 0.36]	–1.52 (0.144)	4.60 (0.88) vs. *5.56 (0.40)*
Ever told you had a stroke	Yes vs. *no*	–1.11 (0.90)	[–3.00, 0.77]	–1.23 (0.233)	4.52 (0.91) vs. *5.63 (0.57)*
Ever told you have diabetes	Yes vs. *no*	–0.28 (0.51)	[–1.34, 0.79]	–0.55 (0.591)	4.94 (0.63) vs. *5.22 (0.69)*
Have smoked at least 100 cigarettes in life	Yes vs. *no*	–0.13 (0.29)	[–0.74, 0.49]	–0.44 (0.666)	5.01 (0.54) vs. *5.14 (0.70)*
All combinations without abdominal obesity	Combinations without AO vs. *metabolically–healthy people*	–0.03 (0.25)	[–0.56, 0.50]	–0.11 (0.915)	5.06 (0.69) vs. *5.09 (0.54)*
Age at screening	Not applied (continuous covariate)	–0.12 (0.02)	[–0.17, –0.08]	–5.64 (< 0.001)	Not applied (continuous cov.)
Ratio of family income to the poverty level	Not applied (continuous covariate)	–0.08 (0.08)	[–0.25, 0.10]	–0.92 (0.366)	Not applied (continuous cov.)
Depression raw scores	Not applied (continuous covariate)	–0.07 (0.05)	[–0.18, 0.04]	–1.33 (0.199)	Not applied (continuous cov.)

[a]The design–based covariance matrix is singular. The validity of results is uncertain. [b]The column labeled "estimated marginal means" shows each category's marginal means against the reference category's marginal mean. The reference categories and the marginal means of the reference categories are shown in italics. The significant results have been colored.

Table S45. Multiple linear regression model explaining the relationship between CERAD–DR and all combinations with high glycemia obesity after adjusting by sociodemographic characteristics and medical history, and estimated marginal means for each category of predictors and covariates

**Model coefficient**	**Categories (for categorical predictor and covariates)**	*Beta* (*SE*)	95% CI	*t* (*p*)	Estimated marginal means[a]
Constant	Not applied (model intercept)	11.99 (1.29)	[9.36, 14.61]	9.30 (< 0.001)	Not applied (model intercept)
Education level	Up to 12th grade vs. *college graduate or above*	–0.41 (0.44)	[–1.30, 0.48]	–0.94 (0.356)	6.08 (0.41) vs. *6.50 (0.45)*
	High school graduate vs. *college graduate or above*	–0.22 (0.41)	[–1.05, 0.61]	–0.54 (0.594)	6.28 (0.45) vs. *6.50 (0.45)*
	Some college or AA degree vs. *college graduate or above*	0.63 (0.31)	[0.00, 1.26]	2.04 (0.049)	7.13 (0.39) vs. *6.50 (0.45)*
Gender	Males vs. *females*	–0.34 (0.22)	[–0.79, 0.11]	–1.56 (0.129)	6.32 (0.36) vs. *6.67 (0.41)*
Race	Mexican American vs. *Non–Hispanic White*	–0.49 (0.40)	[–1.29, 0.32]	–1.23 (0.228)	6.49 (0.48) vs. *6.98 (0.38)*
	Other Hispanic vs. *Non–Hispanic White*	–1.06 (0.39)	[–1.85, –0.26]	–2.71 (0.011)	5.92 (0.46) vs. *6.98 (0.38)*
	Non–Hispanic Black vs. *Non–Hispanic White*	–0.37 (0.31)	[–0.99, 0.26]	–1.20 (0.240)	6.61 (0.50) vs. *6.98 (0.38)*
	Non–Hispanic Asian vs. *Non–Hispanic White*	0.85 (0.35)	[0.13, 1.57]	2.42 (0.021)	7.83 (0.47) vs. *6.98 (0.38)*
	Other Race and Multi–Racial vs. *Non–Hispanic White*	–1.85 (0.47)	[–2.80, –0.89]	–3.92 (< 0.001)	5.13 (0.48) vs. *6.98 (0.38)*
Marital status	Single or never married vs. *married or living with a partner*	1.13 (0.42)	[0.27, 1.99]	2.67 (0.012)	7.35 (0.46) vs. *6.22 (0.36)*
	Divorced or separated vs. *married or living with a partner*	0.24 (0.23)	[–0.22, 0.70]	1.05 (0.301)	6.46 (0.45) vs. *6.22 (0.36)*
	Widowed vs. *married or living with a partner*	–0.25 (0.32)	[–0.91, 0.40]	–0.80 (0.431)	5.96 (0.48) vs. *6.22 (0.36)*
Self–report general health condition	Poor vs. *excellent or very good*	1.65 (0.77)	[0.08, 3.21]	2.14 (0.040)	7.58 (0.67) vs. *5.93 (0.45)*
	Good or fair vs. *excellent or very good*	0.05 (0.33)	[–0.61, 0.72]	0.16 (0.874)	5.98 (0.35) vs. *5.93 (0.45)*
Difficulties in thinking or remembering	Yes vs. *no*	–0.83 (0.35)	[–1.53, –0.12]	–2.39 (0.023)	6.08 (0.45) vs. *6.91 (0.36)*
Ever told you have a heart disease	Yes vs. *no*	–0.13 (0.39)	[–0.91, 0.66]	–0.33 (0.743)	6.43 (0.50) vs. *6.56 (0.31)*
Ever told you had a stroke	Yes vs. *no*	0.21 (0.43)	[–0.66, 1.08]	0.49 (0.626)	6.60 (0.54) vs. *6.39 (0.27)*
Ever told you have diabetes	Yes vs. *no*	–0.40 (0.39)	[–1.20, 0.39]	–1.03 (0.309)	6.29 (0.39) vs. *6.70 (0.44)*
Have smoked at least 100 cigarettes in life	Yes vs. *no*	–0.27 (0.22)	[–0.73, 0.18]	–1.22 (0.230)	6.36 (0.34) vs. *6.63 (0.42)*
All combinations with high glycemia	Combinations with GLY vs. *metabolically–healthy people*	–0.54 (0.27)	[–1.08, 0.00]	–2.02 (0.052)	6.23 (0.34) vs. *6.77 (0.43)*
Age at screening	Not applied (continuous covariate)	–0.07 (0.02)	[–0.10, –0.03]	–3.77 (0.001)	Not applied (continuous cov.)
Ratio of family income to the poverty level	Not applied (continuous covariate)	0.01 (0.08)	[–0.16, 0.19]	0.16 (0.871)	Not applied (continuous cov.)
Depression raw scores	Not applied (continuous covariate)	–0.03 (0.02)	[–0.07, 0.02]	–1.23 (0.228)	Not applied (continuous cov.)

[a]The column labeled "estimated marginal means" shows each category's marginal means against the reference category's marginal mean. The reference categories and the marginal means of the reference categories are shown in italics. The significant results have been colored.

Table S46. Multiple linear regression model explaining the relationship between CERAD–DR and all combinations without high glycemia obesity after adjusting by sociodemographic characteristics and medical history, and estimated marginal means for each category of predictors and covariates[a]

Model coefficient	Categories (for categorical predictor and covariates)	*Beta* (*SE*)	95% CI	*t* (*p*)	Estimated marginal means[b]
Constant	Not applied (model intercept)	16.52 (1.59)	[13.17, 19.88]	10.39 (< 0.001)	Not applied (model intercept)
Education level	Up to 12th grade vs. *college graduate or above*	–0.29 (0.42)	[–1.17, 0.59]	–0.70 (0.493)	4.82 (0.60) vs. *5.12 (0.62)*
	High school graduate vs. *college graduate or above*	–1.21 (0.45)	[–2.16, –0.25]	–2.67 (0.016)	3.91 (0.66) vs. *5.12 (0.62)*
	Some college or AA degree vs. *college graduate or above*	–0.04 (0.34)	[–0.76, 0.68]	–0.13 (0.900)	5.07 (0.63) vs. *5.12 (0.62)*
Gender	Males vs. *females*	–0.70 (0.30)	[–1.33, –0.07]	–2.34 (0.032)	4.38 (0.60) vs. *5.08 (0.61)*
Race	Mexican American vs. *Non–Hispanic White*	–0.34 (0.63)	[–1.67, 0.99]	–0.54 (0.599)	5.08 (0.81) vs. *5.42 (0.63)*
	Other Hispanic vs. *Non–Hispanic White*	–1.42 (0.60)	[–2.69, –0.15]	–2.35 (0.031)	4.00 (0.63) vs. *5.42 (0.63)*
	Non–Hispanic Black vs. *Non–Hispanic White*	–1.00 (0.33)	[–1.69, –0.31]	–3.06 (0.007)	4.42 (0.51) vs. *5.42 (0.63)*
	Non–Hispanic Asian vs. *Non–Hispanic White*	0.26 (0.57)	[–0.95, 1.47]	0.45 (0.657)	5.68 (0.87) vs. *5.42 (0.63)*
	Other Race and Multi–Racial vs. *Non–Hispanic White*	–1.65 (0.75)	[–3.24, –0.07]	–2.20 (0.042)	3.77 (0.99) vs. *5.42 (0.63)*
Marital status	Single or never married vs. *married or living with a partner*	–0.17 (0.75)	[–1.75, 1.40]	–0.23 (0.821)	4.78 (0.89) vs. *4.95 (0.68)*
	Divorced or separated vs. *married or living with a partner*	–0.01 (0.69)	[–1.47, 1.45]	–0.01 (0.993)	4.95 (0.67) vs. *4.95 (0.68)*
	Widowed vs. *married or living with a partner*	–0.71 (0.42)	[–1.59, 0.18]	–1.67 (0.112)	4.25 (0.72) vs. *4.95 (0.68)*
Self–report general health condition	Poor vs. *excellent or very good*	0.68 (0.98)	[–1.40, 2.75]	0.69 (0.500)	5.19 (1.03) vs. *4.51 (0.55)*
	Good or fair vs. *excellent or very good*	–0.02 (0.31)	[–0.68, 0.63]	–0.07 (0.941)	4.49 (0.54) vs. *4.51 (0.55)*
Difficulties in thinking or remembering	Yes vs. *no*	–1.53 (0.46)	[–2.50, –0.55]	–3.31 (0.004)	3.97 (0.57) vs. *5.49 (0.69)*
Ever told you have a heart disease	Yes vs. *no*	–0.77 (0.58)	[–1.98, 0.45]	–1.33 (0.201)	4.35 (0.68) vs. *5.11 (0.63)*
Ever told you had a stroke	Yes vs. *no*	–0.12 (1.06)	[–2.35, 2.12]	–0.11 (0.914)	4.67 (1.06) vs. *4.79 (0.35)*
Ever told you have diabetes	Yes vs. *no*	–0.74 (0.46)	[–1.72, 0.24]	–1.60 (0.128)	4.36 (0.73) vs. *5.10 (0.51)*
Have smoked at least 100 cigarettes in life	Yes vs. *no*	–0.34 (0.35)	[–1.07, 0.40]	–0.96 (0.350)	4.56 (0.56) vs. *4.90 (0.66)*
All combinations without high glycemia	Combinations without GLY vs. *metabolically–healthy people*	–1.09 (0.35)	[–1.84, –0.35]	–3.11 (0.006)	4.18 (0.57) vs. *5.28 (0.65)*
Age at screening	Not applied (continuous covariate)	–0.12 (0.02)	[–0.17, –0.07]	–5.06 (< 0.001)	Not applied (continuous cov.)
Ratio of family income to the poverty level	Not applied (continuous covariate)	0.05 (0.10)	[–0.17, 0.26]	0.47 (0.641)	Not applied (continuous cov.)
Depression raw scores	Not applied (continuous covariate)	0.05 (0.01)	[0.02, 0.08]	3.69 (0.002)	Not applied (continuous cov.)

[a]The design–based covariance matrix is singular. The validity of results is uncertain. [b]The column labeled "estimated marginal means" shows each category's marginal means against the reference category's marginal mean. The reference categories and the marginal means of the reference categories are shown in italics. The significant results have been colored.

Table S47. Multiple linear regression model explaining the relationship between AFT and classic MetS diagnosis after adjusting by sociodemographic characteristics and medical history, and estimated marginal means for each category of predictors and covariates

**Model coefficient**	**Categories (for categorical predictor and covariates)**	*Beta* (*SE*)	95% CI	*t* (*p*)	Estimated marginal means[a]
Constant	Not applied (model intercept)	33.97 (2.59)	[28.70, 39.24]	13.13 (< 0.001)	Not applied (model intercept)
Education level	Up to 12th grade vs. *college graduate or above*	–3.53 (0.70)	[–4.96, –2.11]	–5.06 (< 0.001)	14.96 (1.28) vs. *18.50 (1.18)*
	High school graduate vs. *college graduate or above*	–2.65 (0.57)	[–3.81, –1.48]	–4.63 (< 0.001)	15.85 (1.18) vs. *18.50 (1.18)*
	Some college or AA degree vs. *college graduate or above*	–1.07 (0.64)	[–2.37, 0.24]	–1.66 (0.106)	17.43 (1.38) vs. *18.50 (1.18)*
Gender	Males vs. *females*	–0.48 (0.59)	[–1.68, 0.72]	–0.82 (0.420)	16.44 (1.20) vs. *16.93 (1.25)*
Race	Mexican American vs. *Non-Hispanic White*	–2.53 (1.04)	[–4.65, –0.41]	–2.43 (0.021)	15.96 (1.35) vs. *18.49 (1.09)*
	Other Hispanic vs. *Non-Hispanic White*	–1.31 (0.90)	[–3.16, 0.53]	–1.45 (0.157)	17.17 (1.30) vs. *18.49 (1.09)*
	Non–Hispanic Black vs. *Non-Hispanic White*	–2.60 (0.45)	[–3.50, –1.69]	–5.83 (< 0.001)	15.89 (1.08) vs. *18.49 (1.09)*
	Non–Hispanic Asian vs. *Non-Hispanic White*	–3.59 (0.77)	[–5.16, –2.03]	–4.68 (< 0.001)	14.89 (1.22) vs. *18.49 (1.09)*
	Other Race and Multi-Racial vs. *Non-Hispanic White*	–0.77 (2.17)	[–5.19, 3.65]	–0.36 (0.724)	17.71 (2.62) vs. *18.49 (1.09)*
Marital status	Single or never married vs. *married or living with a partner*	0.24 (1.58)	[–2.96, 3.45]	0.16 (0.878)	17.17 (1.98) vs. *16.93 (1.21)*
	Divorced or separated vs. *married or living with a partner*	–0.10 (0.57)	[–1.26, 1.06]	–0.18 (0.861)	16.82 (1.28) vs. *16.93 (1.21)*
	Widowed vs. *married or living with a partner*	–1.10 (0.69)	[–2.51, 0.30]	–1.60 (0.119)	15.82 (1.20) vs. *16.93 (1.21)*
Self-report general health condition	Poor vs. *excellent or very good*	1.82 (1.66)	[–1.55, 5.19]	1.10 (0.280)	18.00 (2.05) vs. *16.18 (0.90)*
	Good or fair vs. *excellent or very good*	–0.31 (0.46)	[–1.25, 0.64]	–0.66 (0.511)	15.87 (1.02) vs. *16.18 (0.90)*
Difficulties in thinking or remembering	Yes vs. *no*	–0.21 (0.94)	[–2.12, 1.70]	–0.22 (0.824)	16.58 (1.46) vs. *16.79 (1.08)*
Ever told you have a heart disease	Yes vs. *no*	–0.85 (0.85)	[–2.57, 0.87]	–1.00 (0.324)	16.26 (1.42) vs. *17.11 (1.09)*
Ever told you had a stroke	Yes vs. *no*	0.67 (1.06)	[–1.49, 2.83]	0.63 (0.531)	17.02 (1.45) vs. *16.35 (1.14)*
Ever told you have diabetes	Yes vs. *no*	–1.35 (0.60)	[–2.58, –0.13]	–2.25 (0.032)	16.01 (1.26) vs. *17.36 (1.20)*
Have smoked at least 100 cigarettes in life	Yes vs. *no*	–0.18 (0.53)	[–1.27, 0.90]	–0.34 (0.733)	16.59 (1.19) vs. *16.78 (1.26)*
Classic MetS diagnosis (all three or more criteria)	MetS vs. *metabolically–healthy people*	–0.51 (0.86)	[–2.26, 1.24]	–0.59 (0.556)	16.43 (1.16) vs. *16.94 (1.37)*
Age at screening	Not applied (continuous covariate)	–0.18 (0.04)	[–0.25, –0.11]	–4.96 (< 0.001)	Not applied (continuous cov.)
Ratio of family income to the poverty level	Not applied (continuous covariate)	0.07 (0.18)	[–0.29, 0.43]	0.39 (0.696)	Not applied (continuous cov.)
Depression raw scores	Not applied (continuous covariate)	–0.05 (0.04)	[–0.13, 0.04]	–1.13 (0.267)	Not applied (continuous cov.)

[a]The column labeled "estimated marginal means" shows each category's marginal means against the reference category's marginal mean. The reference categories and the marginal means of the reference categories are shown in italics. The significant results have been colored.

Table S48. Multiple linear regression model explaining the relationship between AFT and specific combination AO + TRI + HDL after adjusting by sociodemographic characteristics and medical history, and estimated marginal means for each category of predictors and covariates[a]

**Model coefficient**	**Categories (for categorical predictor and covariates)**	*Beta* (*SE*)	95% CI	*t* (*p*)	Estimated marginal means[b]
Constant	Not applied (model intercept)	40.40 (9.03)	[20.89, 59.90]	4.47 (0.001)	Not applied (model intercept)
Education level	Up to 12th grade vs. *college graduate or above*	–7.37 (1.65)	[–10.94, –3.81]	–4.47 (0.001)	13.46 (1.49) vs. *20.83 (1.15)*
	High school graduate vs. *college graduate or above*	–3.53 (1.13)	[–5.97, –1.08]	–3.12 (0.008)	17.31 (0.89) vs. *20.83 (1.15)*
	Some college or AA degree vs. *college graduate or above*	–1.12 (1.79)	[–4.97, 2.74]	–0.62 (0.543)	19.72 (1.31) vs. *20.83 (1.15)*
Gender	Males vs. *females*	1.16 (0.89)	[–0.76, 3.09]	1.31 (0.214)	18.41 (1.01) vs. *17.25 (1.03)*
Race	Mexican American vs. *Non–Hispanic White*	–1.77 (1.53)	[–5.07, 1.54]	–1.16 (0.268)	15.40 (1.64) vs. *17.17 (1.02)*
	Other Hispanic vs. *Non–Hispanic White*	8.16 (1.67)	[4.55, 11.77]	4.88 (< 0.001)	25.33 (1.72) vs. *17.17 (1.02)*
	Non–Hispanic Black vs. *Non–Hispanic White*	–0.44 (0.77)	[–2.10, 1.22]	–0.57 (0.576)	16.73 (0.76) vs. *17.17 (1.02)*
	Non–Hispanic Asian vs. *Non–Hispanic White*	–2.63 (1.64)	[–6.17, 0.91]	–1.61 (0.132)	14.53 (1.92) vs. *17.17 (1.02)*
	Other Race and Multi–Racial vs. *Non–Hispanic White*	No cases	No cases	No cases	No cases
Marital status	Single or never married vs. *married or living with a partner*	–10.66 (2.44)	[–15.93, –5.38]	–4.37 (0.001)	10.38 (2.27) vs. *21.04 (0.74)*
	Divorced or separated vs. *married or living with a partner*	–2.08 (1.92)	[–6.22, 2.06]	–1.09 (0.297)	18.96 (1.94) vs. *21.04 (0.74)*
	Widowed vs. *married or living with a partner*	–0.10 (0.98)	[–2.22, 2.02]	–0.10 (0.922)	20.94 (0.81) vs. *21.04 (0.74)*
Self–report general health condition	Poor vs. *excellent or very good*	8.46 (2.98)	[2.02, 14.91]	2.84 (0.014)	23.56 (2.40) vs. *15.10 (1.26)*
	Good or fair vs. *excellent or very good*	–0.26 (0.83)	[–2.06, 1.54]	–0.32 (0.757)	14.83 (0.95) vs. *15.10 (1.26)*
Difficulties in thinking or remembering	Yes vs. *no*	–0.59 (1.58)	[–4.00, 2.82]	–0.37 (0.714)	17.53 (0.93) vs. *18.13 (1.44)*
Ever told you have a heart disease	Yes vs. *no*	–0.94 (0.91)	[–2.91, 1.03]	–1.03 (0.321)	17.36 (0.86) vs. *18.30 (1.17)*
Ever told you had a stroke	Yes vs. *no*	–3.67 (0.79)	[–5.38, –1.95]	–4.62 (< 0.001)	16.00 (1.25) vs. *19.66 (0.66)*
Ever told you have diabetes	Yes vs. *no*	2.74 (1.18)	[0.19, 5.29]	2.32 (0.037)	19.20 (1.08) vs. *16.46 (1.10)*
Have smoked at least 100 cigarettes in life	Yes vs. *no*	–0.80 (1.07)	[–3.11, 1.51]	–0.74 (0.470)	17.43 (1.13) vs. *18.23 (0.99)*
Three criteria combination: AO + TRI + HDL	AO + TRI + HDL vs. *metabolically–healthy people*	–1.22 (0.96)	[–3.29, 0.86]	–1.27 (0.227)	17.22 (1.08) vs. *18.44 (0.99)*
Age at screening	Not applied (continuous covariate)	–0.27 (0.11)	[–0.51, –0.03]	–2.44 (0.030)	Not applied (continuous cov.)
Ratio of family income to the poverty level	Not applied (continuous covariate)	0.12 (0.46)	[–0.87, 1.11]	0.26 (0.795)	Not applied (continuous cov.)
Depression raw scores	Not applied (continuous covariate)	–0.11 (0.07)	[–0.25, 0.04]	–1.62 (0.128)	Not applied (continuous cov.)

[a]The design–based covariance matrix is singular. The validity of results is uncertain. [b]The column labeled "estimated marginal means" shows each category's marginal means against the reference category's marginal mean. The reference categories and the marginal means of the reference categories are shown in italics. The significant results have been colored.

Table S49. Multiple linear regression model explaining the relationship between AFT and specific combination AO + TRI + HBP after adjusting by sociodemographic characteristics and medical history, and estimated marginal means for each category of predictors and covariates[a]

**Model coefficient**	**Categories (for categorical predictor and covariates)**	*Beta* (*SE*)	95% CI	*t* (*p*)	Estimated marginal means[b]
Constant	Not applied (model intercept)	28.05 (7.34)	[12.31, 43.80]	3.82 (0.002)	Not applied (model intercept)
Education level	Up to 12th grade vs. *college graduate or above*	–7.65 (1.61)	[–11.11, –4.19]	–4.75 (< 0.001)	7.46 (2.54) vs. *15.11 (1.77)*
	High school graduate vs. *college graduate or above*	–2.91 (1.04)	[–5.13, –0.68]	–2.80 (0.014)	12.20 (1.40) vs. *15.11 (1.77)*
	Some college or AA degree vs. *college graduate or above*	–1.47 (1.11)	[–3.86, 0.91]	–1.33 (0.206)	13.63 (1.73) vs. *15.11 (1.77)*
Gender	Males vs. *females*	1.35 (0.82)	[–0.41, 3.10]	1.65 (0.121)	12.78 (1.52) vs. *11.43 (1.99)*
Race	Mexican American vs. *Non–Hispanic White*	–0.95 (1.49)	[–4.14, 2.24]	–0.64 (0.534)	10.55 (1.87) vs. *11.50 (1.75)*
	Other Hispanic vs. *Non–Hispanic White*	6.19 (3.65)	[–1.64, 14.01]	1.70 (0.112)	17.68 (3.51) vs. *11.50 (1.75)*
	Non–Hispanic Black vs. *Non–Hispanic White*	–1.03 (0.93)	[–3.03, 0.97]	–1.10 (0.288)	10.47 (1.99) vs. *11.50 (1.75)*
	Non–Hispanic Asian vs. *Non–Hispanic White*	–1.19 (1.78)	[–5.02, 2.63]	–0.67 (0.514)	10.30 (2.41) vs. *11.50 (1.75)*
	Other Race and Multi–Racial vs. *Non–Hispanic White*	No cases	No cases	No cases	No cases
Marital status	Single or never married vs. *married or living with a partner*	–11.21 (2.70)	[–17.00, –5.42]	–4.15 (0.001)	4.32 (3.05) vs. *15.53 (1.44)*
	Divorced or separated vs. *married or living with a partner*	–0.98 (1.53)	[–4.26, 2.31]	–0.64 (0.534)	14.55 (2.01) vs. *15.53 (1.44)*
	Widowed vs. *married or living with a partner*	–1.52 (0.88)	[–3.40, 0.36]	–1.73 (0.105)	14.01 (1.82) vs. *15.53 (1.44)*
Self–report general health condition	Poor vs. *excellent or very good*	No cases	No cases	No cases	No cases
	Good or fair vs. *excellent or very good*	0.41 (0.73)	[–1.16, 1.98]	0.56 (0.586)	12.31 (1.56) vs. *11.90 (1.94)*
Difficulties in thinking or remembering	Yes vs. *no*	–0.05 (1.13)	[–2.46, 2.37]	–0.04 (0.966)	12.08 (1.74) vs. *12.13 (1.89)*
Ever told you have a heart disease	Yes vs. *no*	–2.56 (0.82)	[–4.31, –0.81]	–3.13 (0.007)	10.82 (1.98) vs. *13.38 (1.54)*
Ever told you had a stroke	Yes vs. *no*	–1.21 (2.07)	[–5.66, 3.24]	–0.58 (0.569)	11.50 (2.57) vs. *12.70 (1.23)*
Ever told you have diabetes	Yes vs. *no*	–4.18 (1.00)	[–6.31, –2.04]	–4.19 (0.001)	10.01 (1.98) vs. *14.19 (1.59)*
Have smoked at least 100 cigarettes in life	Yes vs. *no*	–0.95 (1.07)	[–3.25, 1.34]	–0.89 (0.388)	11.62 (1.79) vs. *12.58 (1.82)*
Three criteria combination: AO + TRI + HBP	AO + TRI + HBP vs. *metabolically–healthy people*	–1.54 (0.91)	[–3.50, 0.42]	–1.68 (0.115)	11.33 (1.68) vs. *12.87 (1.89)*
Age at screening	Not applied (continuous covariate)	–0.08 (0.08)	[–0.26, 0.09]	–1.04 (0.314)	Not applied (continuous cov.)
Ratio of family income to the poverty level	Not applied (continuous covariate)	0.11 (0.35)	[–0.64, 0.86]	0.31 (0.761)	Not applied (continuous cov.)
Depression raw scores	Not applied (continuous covariate)	–0.11 (0.10)	[–0.32, 0.10]	–1.10 (0.291)	Not applied (continuous cov.)

[a]The design–based covariance matrix is singular. The validity of results is uncertain. [b]The column labeled "estimated marginal means" shows each category's marginal means against the reference category's marginal mean. The reference categories and the marginal means of the reference categories are shown in italics. The significant results have been colored.

Table S50. Multiple linear regression model explaining the relationship between AFT and specific combination AO + TRI + GLY after adjusting by sociodemographic characteristics and medical history, and estimated marginal means for each category of predictors and covariates[a]

**Model coefficient**	**Categories (for categorical predictor and covariates)**	*Beta* (*SE*)	95% CI	*t* (*p*)	Estimated marginal means[b]
Constant	Not applied (model intercept)	40.71 (5.74)	[28.77, 52.65]	7.09 (< 0.001)	Not applied (model intercept)
Education level	Up to 12th grade vs. *college graduate or above*	–6.04 (1.59)	[–9.34, –2.73]	–3.80 (0.001)	15.42 (2.77) vs. *21.46 (3.10)*
	High school graduate vs. *college graduate or above*	–4.67 (1.20)	[–7.16, –2.19]	–3.91 (0.001)	16.79 (2.61) vs. *21.46 (3.10)*
	Some college or AA degree vs. *college graduate or above*	–2.01 (1.46)	[–5.05, 1.02]	–1.38 (0.182)	19.45 (2.58) vs. *21.46 (3.10)*
Gender	Males vs. *females*	0.52 (1.08)	[–1.72, 2.76]	0.48 (0.636)	18.54 (2.42) vs. *18.02 (2.96)*
Race	Mexican American vs. *Non–Hispanic White*	–1.33 (1.38)	[–4.20, 1.54]	–0.96 (0.347)	17.16 (2.86) vs. *18.49 (2.41)*
	Other Hispanic vs. *Non–Hispanic White*	4.91 (2.29)	[0.15, 9.67]	2.14 (0.044)	23.39 (3.77) vs. 18.49 (2.41)
	Non–Hispanic Black vs. *Non–Hispanic White*	–1.34 (1.42)	[–4.29, 1.60]	–0.95 (0.353)	17.14 (2.96) vs. *18.49 (2.41)*
	Non–Hispanic Asian vs. *Non–Hispanic White*	–3.27 (1.38)	[–6.13, –0.41]	–2.38 (0.027)	15.22 (2.56) vs. *18.49 (2.41)*
	Other Race and Multi–Racial vs. *Non–Hispanic White*	No cases	No cases	No cases	No cases
Marital status	Single or never married vs. *married or living with a partner*	–6.50 (2.75)	[–12.22, –0.78]	–2.36 (0.028)	14.35 (3.95) vs. *20.85 (2.36)*
	Divorced or separated vs. *married or living with a partner*	–2.24 (1.48)	[–5.32, 0.84]	–1.52 (0.145)	18.61 (2.70) vs. *20.85 (2.36)*
	Widowed vs. *married or living with a partner*	–1.54 (0.98)	[–3.58, 0.51]	–1.56 (0.133)	19.31 (2.65) vs. *20.85 (2.36)*
Self–report general health condition	Poor vs. *excellent or very good*	7.91 (2.97)	[1.73, 14.09]	2.66 (0.015)	23.67 (4.39) vs. *15.76 (2.22)*
	Good or fair vs. *excellent or very good*	–0.36 (0.94)	[–2.32, 1.60]	–0.38 (0.706)	15.40 (1.90) vs. *15.76 (2.22)*
Difficulties in thinking or remembering	Yes vs. *no*	–1.11 (1.53)	[–4.29, 2.06]	–0.73 (0.475)	17.72 (2.79) vs. *18.83 (2.73)*
Ever told you have a heart disease	Yes vs. *no*	–1.32 (1.50)	[–4.44, 1.81]	–0.88 (0.390)	17.62 (3.27) vs. *18.94 (2.12)*
Ever told you had a stroke	Yes vs. *no*	–0.63 (2.38)	[–5.59, 4.32]	–0.27 (0.793)	17.96 (3.39) vs. *18.60 (2.33)*
Ever told you have diabetes	Yes vs. *no*	1.22 (1.82)	[–2.56, 5.00]	0.67 (0.510)	18.89 (3.08) vs. *17.67 (2.50)*
Have smoked at least 100 cigarettes in life	Yes vs. *no*	–0.54 (1.17)	[–2.98, 1.90]	–0.46 (0.649)	18.01 (2.77) vs. *18.55 (2.66)*
Three criteria combination: AO + TRI + GLY	AO + TRI + HBP vs. *metabolically–healthy people*	–0.81 (1.03)	[–2.95, 1.32]	–0.79 (0.437)	17.87 (2.62) vs. *18.69 (2.78)*
Age at screening	Not applied (continuous covariate)	–0.25 (0.07)	[–0.40, –0.10]	–3.51 (0.002)	Not applied (continuous cov.)
Ratio of family income to the poverty level	Not applied (continuous covariate)	–0.09 (0.34)	[–0.80, 0.63]	–0.25 (0.805)	Not applied (continuous cov.)
Depression raw scores	Not applied (continuous covariate)	–0.14 (0.15)	[–0.45, 0.17]	–0.92 (0.368)	Not applied (continuous cov.)

[a]The design–based covariance matrix is singular. The validity of results is uncertain. [b]The column labeled "estimated marginal means" shows each category's marginal means against the reference category's marginal mean. The reference categories and the marginal means of the reference categories are shown in italics. The significant results have been colored.

Table S51. Multiple linear regression model explaining the relationship between AFT and specific combination AO + HDL + HBP after adjusting by sociodemographic characteristics and medical history, and estimated marginal means for each category of predictors and covariates[a]

**Model coefficient**	**Categories (for categorical predictor and covariates)**	*Beta* (*SE*)	95% CI	*t* (*p*)	Estimated marginal means[b]
Constant	Not applied (model intercept)	41.02 (10.39)	[18.99, 63.04]	3.95 (0.001)	Not applied (model intercept)
Education level	Up to 12th grade vs. *college graduate or above*	–6.46 (2.31)	[–11.35, –1.57]	–2.80 (0.013)	9.57 (2.37) vs. *16.03 (1.73)*
	High school graduate vs. *college graduate or above*	–3.54 (1.51)	[–6.74, –0.35]	–2.35 (0.032)	12.49 (1.39) vs. *16.03 (1.73)*
	Some college or AA degree vs. *college graduate or above*	–0.16 (2.39)	[–5.22, 4.90]	–0.07 (0.947)	15.87 (2.26) vs. *16.03 (1.73)*
Gender	Males vs. *females*	1.43 (1.33)	[–1.38, 4.24]	1.08 (0.297)	14.21 (1.92) vs. *12.78 (1.64)*
Race	Mexican American vs. *Non–Hispanic White*	–2.65 (1.91)	[–6.71, 1.41]	–1.38 (0.185)	11.22 (1.52) vs. *13.87 (2.05)*
	Other Hispanic vs. *Non–Hispanic White*	7.26 (1.96)	[3.10, 11.41]	3.70 (0.002)	21.12 (1.92) vs. *13.87 (2.05)*
	Non–Hispanic Black vs. *Non–Hispanic White*	–1.01 (1.18)	[–3.52, 1.50]	–0.85 (0.406)	12.86 (2.73) vs. *13.87 (2.05)*
	Non–Hispanic Asian vs. *Non–Hispanic White*	–1.93 (1.57)	[–5.25, 1.40]	–1.23 (0.237)	11.94 (2.62) vs. *13.87 (2.05)*
	Other Race and Multi–Racial vs. *Non–Hispanic White*	–3.93 (2.40)	[–9.01, 1.15]	–1.64 (0.120)	9.94 (2.55) vs. *13.87 (2.05)*
Marital status	Single or never married vs. *married or living with a partner*	–9.82 (3.27)	[–16.75, –2.88]	–3.00 (0.008)	6.51 (3.68) vs. *16.32 (1.32)*
	Divorced or separated vs. *married or living with a partner*	–1.63 (1.92)	[–5.70, 2.43]	–0.85 (0.407)	14.69 (1.55) vs. *16.32 (1.32)*
	Widowed vs. *married or living with a partner*	0.12 (1.07)	[–2.15, 2.39]	0.11 (0.913)	16.44 (1.89) vs. *16.32 (1.32)*
Self–report general health condition	Poor vs. *excellent or very good*	No cases	No cases	No cases	No cases
	Good or fair vs. *excellent or very good*	0.21 (1.03)	[–1.97, 2.39]	0.21 (0.839)	13.60 (1.35) vs. *13.38 (2.06)*
Difficulties in thinking or remembering	Yes vs. *no*	–0.95 (1.34)	[–3.79, 1.90]	–0.70 (0.491)	13.02 (1.80) vs. *13.96 (1.79)*
Ever told you have a heart disease	Yes vs. *no*	–2.13 (1.40)	[–5.09, 0.84]	–1.52 (0.148)	12.43 (2.17) vs. *14.55 (1.33)*
Ever told you had a stroke	Yes vs. *no*	–1.93 (1.53)	[–5.17, 1.31]	–1.26 (0.225)	12.53 (2.19) vs. *14.46 (1.37)*
Ever told you have diabetes	Yes vs. *no*	–1.36 (1.96)	[–5.52, 2.80]	–0.69 (0.497)	12.81 (2.19) vs. *14.17 (1.62)*
Have smoked at least 100 cigarettes in life	Yes vs. *no*	–0.09 (1.30)	[–2.84, 2.66]	–0.07 (0.945)	13.45 (1.37) vs. *13.54 (2.12)*
Three criteria combination: AO + HDL + HBP	AO + HDL + HBP vs. *metabolically–healthy people*	0.54 (1.64)	[–2.95, 4.02]	0.33 (0.748)	13.76 (1.97) vs. *13.22 (1.73)*
Age at screening	Not applied (continuous covariate)	–0.28 (0.14)	[–0.58, 0.01]	–2.05 (0.057)	Not applied (continuous cov.)
Ratio of family income to the poverty level	Not applied (continuous covariate)	–0.05 (0.44)	[–0.99, 0.89]	–0.11 (0.911)	Not applied (continuous cov.)
Depression raw scores	Not applied (continuous covariate)	–0.12 (0.15)	[–0.45, 0.20]	–0.80 (0.433)	Not applied (continuous cov.)

[a]The design–based covariance matrix is singular. The validity of results is uncertain. [b]The column labeled "estimated marginal means" shows each category's marginal means against the reference category's marginal mean. The reference categories and the marginal means of the reference categories are shown in italics. The significant results have been colored.

Table S52. Multiple linear regression model explaining the relationship between AFT and specific combination AO + HDL + GLY after adjusting by sociodemographic characteristics and medical history, and estimated marginal means for each category of predictors and covariates[a]

**Model coefficient**	**Categories (for categorical predictor and covariates)**	*Beta* (*SE*)	95% CI	*t* (*p*)	Estimated marginal means[b]
Constant	Not applied (model intercept)	37.97 (7.82)	[21.79, 54.15]	4.85 (< 0.001)	Not applied (model intercept)
Education level	Up to 12th grade vs. *college graduate or above*	–7.08 (1.89)	[–10.98, –3.17]	–3.75 (0.001)	10.68 (2.17) vs. *17.75 (1.73)*
	High school graduate vs. *college graduate or above*	–3.89 (1.36)	[–6.70, –1.09]	–2.87 (0.009)	13.86 (1.61) vs. *17.75 (1.73)*
	Some college or AA degree vs. *college graduate or above*	–2.59 (1.65)	[–6.01, 0.84]	–1.56 (0.132)	15.17 (1.55) vs. *17.75 (1.73)*
Gender	Males vs. *females*	1.13 (1.03)	[–0.99, 3.25]	1.11 (0.280)	14.93 (1.56) vs. *13.80 (1.69)*
Race	Mexican American vs. *Non–Hispanic White*	–0.91 (1.42)	[–3.84, 2.02]	–0.64 (0.526)	15.28 (1.83) vs. *16.19 (1.62)*
	Other Hispanic vs. *Non–Hispanic White*	2.70 (1.64)	[–0.70, 6.10]	1.64 (0.114)	18.89 (1.91) vs. *16.19 (1.62)*
	Non–Hispanic Black vs. *Non–Hispanic White*	–1.07 (1.46)	[–4.08, 1.94]	–0.74 (0.469)	15.12 (2.07) vs. *16.19 (1.62)*
	Non–Hispanic Asian vs. *Non–Hispanic White*	–4.11 (1.67)	[–7.57, –0.65]	–2.46 (0.022)	12.09 (1.92) vs. *16.19 (1.62)*
	Other Race and Multi–Racial vs. *Non–Hispanic White*	–7.58 (1.97)	[–11.66, –3.49]	–3.84 (0.001)	8.62 (2.75) vs. *16.19 (1.62)*
Marital status	Single or never married vs. *married or living with a partner*	–7.26 (2.17)	[–11.75, –2.77]	–3.34 (0.003)	9.87 (2.39) vs. *17.13 (1.35)*
	Divorced or separated vs. *married or living with a partner*	–2.54 (1.03)	[–4.67, –0.41]	–2.47 (0.021)	14.59 (1.71) vs. *17.13 (1.35)*
	Widowed vs. *married or living with a partner*	–1.26 (1.20)	[–3.74, 1.22]	–1.05 (0.303)	15.87 (2.07) vs. *17.13 (1.35)*
Self–report general health condition	Poor vs. *excellent or very good*	2.42 (2.43)	[–2.62, 7.45]	0.99 (0.331)	15.70 (2.78) vs. *13.29 (1.63)*
	Good or fair vs. *excellent or very good*	0.82 (1.29)	[–1.84, 3.49]	0.64 (0.529)	14.11 (0.93) vs. *13.29 (1.63)*
Difficulties in thinking or remembering	Yes vs. *no*	1.45 (1.17)	[–0.97, 3.86]	1.24 (0.228)	15.09 (1.98) vs. *13.64 (1.22)*
Ever told you have a heart disease	Yes vs. *no*	–0.39 (1.10)	[–2.67, 1.88]	–0.36 (0.723)	14.17 (1.95) vs. *14.56 (1.24)*
Ever told you had a stroke	Yes vs. *no*	–3.67 (1.28)	[–6.31, –1.03]	–2.87 (0.009)	12.53 (1.85) vs. *16.20 (1.46)*
Ever told you have diabetes	Yes vs. *no*	2.27 (1.50)	[–0.84, 5.38]	1.51 (0.145)	15.50 (1.90) vs. *13.23 (1.51)*
Have smoked at least 100 cigarettes in life	Yes vs. *no*	–0.47 (1.18)	[–2.91, 1.96]	–0.40 (0.691)	14.13 (1.56) vs. *14.60 (1.73)*
Three criteria combination: AO + HDL + GLY	AO + HDL + GLY vs. *metabolically–healthy people*	–1.02 (1.30)	[–3.70, 1.67]	–0.78 (0.443)	13.86 (1.49) vs. *14.87 (1.84)*
Age at screening	Not applied (continuous covariate)	–0.24 (0.10)	[–0.45, –0.03]	–2.36 (0.027)	Not applied (continuous cov.)
Ratio of family income to the poverty level	Not applied (continuous covariate)	0.38 (0.42)	[–0.49, 1.25]	0.90 (0.379)	Not applied (continuous cov.)
Depression raw scores	Not applied (continuous covariate)	–0.26 (0.14)	[–0.56, 0.04]	–1.78 (0.088)	Not applied (continuous cov.)

[a]The design–based covariance matrix is singular. The validity of results is uncertain. [b]The column labeled "estimated marginal means" shows each category's marginal means against the reference category's marginal mean. The reference categories and the marginal means of the reference categories are shown in italics. The significant results have been colored.

Table S53. Multiple linear regression model explaining the relationship between AFT and specific combination AO + HBP + GLY after adjusting by sociodemographic characteristics and medical history, and estimated marginal means for each category of predictors and covariates

**Model coefficient**	**Categories (for categorical predictor and covariates)**	*Beta* (*SE*)	95% CI	*t* (*p*)	Estimated marginal means[a]
Constant	Not applied (model intercept)	34.22 (3.91)	[26.21, 42.22]	8.74 (< 0.001)	Not applied (model intercept)
Education level	Up to 12th grade vs. *college graduate or above*	–2.49 (1.21)	[–4.97, –0.01]	–2.06 (0.049)	12.91 (1.81) vs. *15.40 (1.71)*
	High school graduate vs. *college graduate or above*	–1.00 (0.85)	[–2.73, 0.73]	–1.18 (0.246)	14.40 (1.83) vs. *15.40 (1.71)*
	Some college or AA degree vs. *college graduate or above*	–0.89 (0.73)	[–2.39, 0.61]	–1.22 (0.233)	14.51 (1.69) vs. *15.40 (1.71)*
Gender	Males vs. *females*	–0.91 (0.78)	[–2.50, 0.69]	–1.16 (0.255)	13.86 (1.83) vs. *14.76 (1.57)*
Race	Mexican American vs. *Non–Hispanic White*	–1.57 (1.13)	[–3.88, 0.74]	–1.39 (0.175)	14.86 (1.86) vs. *16.43 (1.47)*
	Other Hispanic vs. *Non–Hispanic White*	–1.35 (1.48)	[–4.38, 1.68]	–0.91 (0.370)	15.08 (1.78) vs. *16.43 (1.47)*
	Non–Hispanic Black vs. *Non–Hispanic White*	–2.87 (0.83)	[–4.58, –1.16]	–3.44 (0.002)	13.56 (1.47) vs. *16.43 (1.47)*
	Non–Hispanic Asian vs. *Non–Hispanic White*	–2.41 (1.86)	[–6.22, 1.41]	–1.29 (0.207)	14.02 (2.61) vs. *16.43 (1.47)*
	Other Race and Multi–Racial vs. *Non–Hispanic White*	–4.51 (2.75)	[–10.13, 1.11]	–1.64 (0.111)	11.91 (3.39) vs. *16.43 (1.47)*
Marital status	Single or never married vs. *married or living with a partner*	–1.93 (3.05)	[–8.17, 4.31]	–0.63 (0.533)	12.90 (3.42) vs. *14.82 (1.63)*
	Divorced or separated vs. *married or living with a partner*	1.58 (0.94)	[–0.33, 3.50]	1.69 (0.102)	16.41 (1.74) vs. *14.82 (1.63)*
	Widowed vs. *married or living with a partner*	–1.71 (1.10)	[–3.97, 0.54]	–1.55 (0.131)	13.11 (1.67) vs. *14.82 (1.63)*
Self–report general health condition	Poor vs. *excellent or very good*	–1.98 (1.96)	[–5.99, 2.03]	–1.01 (0.321)	13.48 (2.62) vs. *15.46 (1.26)*
	Good or fair vs. *excellent or very good*	–1.48 (0.64)	[–2.80, –0.16]	–2.30 (0.029)	13.98 (1.48) vs. *15.46 (1.26)*
Difficulties in thinking or remembering	Yes vs. *no*	–1.48 (1.13)	[–3.79, 0.82]	–1.32 (0.198)	13.57 (1.92) vs. *15.05 (1.56)*
Ever told you have a heart disease	Yes vs. *no*	–2.16 (1.44)	[–5.09, 0.78]	–1.50 (0.144)	13.23 (2.13) vs. *15.39 (1.41)*
Ever told you had a stroke	Yes vs. *no*	1.86 (1.68)	[–1.59, 5.30]	1.10 (0.279)	15.24 (2.10) vs. *13.38 (1.58)*
Ever told you have diabetes	Yes vs. *no*	–2.87 (0.85)	[–4.60, –1.14]	–3.39 (0.002)	12.87 (1.78) vs. *15.74 (1.63)*
Have smoked at least 100 cigarettes in life	Yes vs. *no*	–1.12 (0.67)	[–2.48, 0.25]	–1.67 (0.107)	13.75 (1.53) vs. *14.87 (1.84)*
Three criteria combination: AO + HBP + GLY	AO + HBP + GLY vs. *metabolically–healthy people*	0.09 (0.89)	[–1.73, 1.92]	0.11 (0.916)	14.36 (1.68) vs. *14.26 (1.75)*
Age at screening	Not applied (continuous covariate)	–0.17 (0.05)	[–0.28, –0.06]	–3.26 (0.003)	Not applied (continuous cov.)
Ratio of family income to the poverty level	Not applied (continuous covariate)	–0.01 (0.23)	[–0.47, 0.45]	–0.05 (0.961)	Not applied (continuous cov.)
Depression raw scores	Not applied (continuous covariate)	–0.01 (0.07)	[–0.16, 0.14]	–0.09 (0.926)	Not applied (continuous cov.)

[a]The column labeled "estimated marginal means" shows each category's marginal means against the reference category's marginal mean. The reference categories and the marginal means of the reference categories are shown in italics. The significant results have been colored.

Table S54. Multiple linear regression model explaining the relationship between AFT and specific combination TRI + HDL + HBP after adjusting by sociodemographic characteristics and medical history, and estimated marginal means for each category of predictors and covariates[a]

**Model coefficient**	**Categories (for categorical predictor and covariates)**	*Beta* (*SE*)	95% CI	*t* (*p*)	Estimated marginal means[b]
Constant	Not applied (model intercept)	42.98 (7.80)	[25.99, 59.97]	5.51 (< 0.001)	Not applied (model intercept)
Education level	Up to 12th grade vs. *college graduate or above*	–6.22 (2.23)	[–11.08, –1.37]	–2.79 (0.016)	11.67 (2.38) vs. *17.89 (2.69)*
	High school graduate vs. *college graduate or above*	–2.73 (1.12)	[–5.17, –0.28]	–2.42 (0.032)	15.17 (1.86) vs. *17.89 (2.69)*
	Some college or AA degree vs. *college graduate or above*	0.82 (1.92)	[–3.38, 5.01]	0.42 (0.679)	18.71 (2.06) vs. *17.89 (2.69)*
Gender	Males vs. *females*	0.49 (1.32)	[–2.39, 3.37]	0.37 (0.717)	16.10 (1.95) vs. *15.61 (2.32)*
Race	Mexican American vs. *Non–Hispanic White*	–2.77 (1.16)	[–5.29, –0.24]	–2.39 (0.034)	12.69 (2.08) vs. *15.45 (1.57)*
	Other Hispanic vs. *Non–Hispanic White*	9.30 (1.77)	[5.43, 13.16]	5.24 (< 0.001)	24.75 (2.71) vs. *15.45 (1.57)*
	Non–Hispanic Black vs. *Non–Hispanic White*	–1.39 (1.85)	[–5.41, 2.64]	–0.75 (0.467)	14.07 (2.86) vs. *15.45 (1.57)*
	Non–Hispanic Asian vs. *Non–Hispanic White*	–3.12 (1.83)	[–7.11, 0.88]	–1.70 (0.115)	12.34 (2.48) vs. *15.45 (1.57)*
	Other Race and Multi–Racial vs. *Non–Hispanic White*	No cases	No cases	No cases	No cases
Marital status	Single or never married vs. *married or living with a partner*	–10.25 (2.42)	[–15.52, –4.97]	–4.23 (0.001)	8.44 (2.95) vs. *18.69 (2.02)*
	Divorced or separated vs. *married or living with a partner*	–1.22 (2.25)	[–6.12, 3.69]	–0.54 (0.599)	17.47 (2.78) vs. *18.69 (2.02)*
	Widowed vs. *married or living with a partner*	0.15 (0.72)	[–1.43, 1.73]	0.21 (0.838)	18.84 (1.75) vs. *18.69 (2.02)*
Self–report general health condition	Poor vs. *excellent or very good*	No cases	No cases	No cases	No cases
	Good or fair vs. *excellent or very good*	–0.63 (0.66)	[–2.06, 0.80]	–0.97 (0.354)	15.54 (1.84) vs. *16.18 (2.27)*
Difficulties in thinking or remembering	Yes vs. *no*	–1.66 (1.47)	[–4.86, 1.54]	–1.13 (0.281)	15.03 (2.25) vs. *16.69 (2.08)*
Ever told you have a heart disease	Yes vs. *no*	–1.53 (1.38)	[–4.54, 1.48]	–1.11 (0.289)	15.09 (2.59) vs. *16.63 (1.59)*
Ever told you had a stroke	Yes vs. *no*	–3.30 (1.01)	[–5.50, –1.11]	–3.28 (0.007)	14.21 (2.17) vs. *17.51 (2.03)*
Ever told you have diabetes	Yes vs. *no*	1.94 (2.02)	[–2.46, 6.35]	0.96 (0.355)	16.83 (2.31) vs. *14.89 (2.23)*
Have smoked at least 100 cigarettes in life	Yes vs. *no*	–1.07 (1.18)	[–3.64, 1.50]	–0.91 (0.381)	15.32 (2.36) vs. *16.40 (1.85)*
Three criteria combination: TRI + HDL + HBP	TRI + HDL + HBP vs. *metabolically–healthy people*	1.70 (2.29)	[–3.30, 6.70]	0.74 (0.473)	16.71 (2.98) vs. *15.01 (1.44)*
Age at screening	Not applied (continuous covariate)	–0.30 (0.10)	[–0.51, –0.09]	–3.12 (0.009)	Not applied (continuous cov.)
Ratio of family income to the poverty level	Not applied (continuous covariate)	–0.18 (0.44)	[–1.13, 0.77]	–0.42 (0.685)	Not applied (continuous cov.)
Depression raw scores	Not applied (continuous covariate)	0.10 (0.29)	[–0.55, 0.74]	0.32 (0.752)	Not applied (continuous cov.)

[a]The design–based covariance matrix is singular. The validity of results is uncertain. [b]The column labeled "estimated marginal means" shows each category's marginal means against the reference category's marginal mean. The reference categories and the marginal means of the reference categories are shown in italics. The significant results have been colored.

Table S55. Multiple linear regression model explaining the relationship between AFT and specific combination TRI + HDL + GLY after adjusting by sociodemographic characteristics and medical history, and estimated marginal means for each category of predictors and covariates[a]

**Model coefficient**	**Categories (for categorical predictor and covariates)**	*Beta* (*SE*)	95% CI	*t* (*p*)	Estimated marginal means[b]
Constant	Not applied (model intercept)	38.63 (8.52)	[20.22, 57.03]	4.53 (0.001)	Not applied (model intercept)
Education level	Up to 12th grade vs. *college graduate or above*	–5.29 (1.69)	[–8.95, –1.63]	–3.12 (0.008)	10.67 (2.90) vs. *15.96 (1.81)*
	High school graduate vs. *college graduate or above*	–2.65 (1.02)	[–4.85, –0.46]	–2.61 (0.022)	13.31 (1.61) vs. *15.96 (1.81)*
	Some college or AA degree vs. *college graduate or above*	–0.15 (1.39)	[–3.16, 2.86]	–0.11 (0.916)	15.81 (1.85) vs. *15.96 (1.81)*
Gender	Males vs. *females*	0.67 (1.08)	[–1.66, 3.00]	0.62 (0.546)	14.27 (1.65) vs. *13.61 (2.25)*
Race	Mexican American vs. *Non–Hispanic White*	–2.84 (1.39)	[–5.85, 0.16]	–2.05 (0.062)	11.12 (2.10) vs. *13.97 (1.71)*
	Other Hispanic vs. *Non–Hispanic White*	5.95 (2.27)	[1.04, 10.86]	2.62 (0.021)	19.92 (3.12) vs. *13.97 (1.71)*
	Non–Hispanic Black vs. *Non–Hispanic White*	0.65 (1.38)	[–2.34, 3.63]	0.47 (0.646)	14.62 (2.80) vs. *13.97 (1.71)*
	Non–Hispanic Asian vs. *Non–Hispanic White*	–3.89 (2.11)	[–8.44, 0.66]	–1.85 (0.088)	10.08 (2.08) vs. *13.97 (1.71)*
	Other Race and Multi–Racial vs. *Non–Hispanic White*	No cases	No cases	No cases	No cases
Marital status	Single or never married vs. *married or living with a partner*	–8.79 (2.05)	[–13.22, –4.37]	–4.29 (0.001)	7.81 (2.75) vs. *16.60 (1.71)*
	Divorced or separated vs. *married or living with a partner*	–0.06 (1.49)	[–3.29, 3.17]	–0.04 (0.968)	16.54 (2.26) vs. *16.60 (1.71)*
	Widowed vs. *married or living with a partner*	–1.78 (1.24)	[–4.47, 0.90]	–1.44 (0.175)	14.82 (2.05) vs. *16.60 (1.71)*
Self–report general health condition	Poor vs. *excellent or very good*	No cases	No cases	No cases	No cases
	Good or fair vs. *excellent or very good*	0.11 (0.96)	[–1.96, 2.18]	0.11 (0.912)	13.99 (1.58) vs. *13.89 (2.28)*
Difficulties in thinking or remembering	Yes vs. *no*	–0.86 (1.60)	[–4.31, 2.59]	–0.54 (0.600)	13.51 (1.91) vs. *14.37 (2.20)*
Ever told you have a heart disease	Yes vs. *no*	–0.13 (1.18)	[–2.69, 2.43]	–0.11 (0.913)	13.87 (2.30) vs. *14.01 (1.62)*
Ever told you had a stroke	Yes vs. *no*	–3.42 (0.81)	[–5.17, –1.67]	–4.22 (0.001)	12.23 (2.03) vs. *15.65 (1.85)*
Ever told you have diabetes	Yes vs. *no*	2.75 (3.33)	[–4.44, 9.94]	0.83 (0.423)	15.32 (3.23) vs. *12.56 (1.53)*
Have smoked at least 100 cigarettes in life	Yes vs. *no*	–1.26 (1.13)	[–3.69, 1.18]	–1.12 (0.285)	13.31 (2.03) vs. *14.57 (1.93)*
Three criteria combination: TRI + HDL + GLY	TRI + HDL + GLY vs. *metabolically–healthy people*	–3.91 (1.44)	[–7.02, –0.81]	–2.72 (0.017)	11.98 (2.12) vs. *15.90 (1.93)*
Age at screening	Not applied (continuous covariate)	–0.23 (0.10)	[–0.45, –0.01]	–2.30 (0.038)	Not applied (continuous cov.)
Ratio of family income to the poverty level	Not applied (continuous covariate)	0.00 (0.38)	[–0.83, 0.82]	–0.01 (0.990)	Not applied (continuous cov.)
Depression raw scores	Not applied (continuous covariate)	–0.52 (0.16)	[–0.87, –0.17]	–3.19 (0.007)	Not applied (continuous cov.)

[a]The design–based covariance matrix is singular. The validity of results is uncertain. [b]The column labeled "estimated marginal means" shows each category's marginal means against the reference category's marginal mean. The reference categories and the marginal means of the reference categories are shown in italics. The significant results have been colored.

Table S56. Multiple linear regression model explaining the relationship between AFT and specific combination TRI + HBP + GLY after adjusting by sociodemographic characteristics and medical history, and estimated marginal means for each category of predictors and covariates[a]

**Model coefficient**	**Categories (for categorical predictor and covariates)**	*Beta* (*SE*)	95% CI	*t* (*p*)	Estimated marginal means[b]
Constant	Not applied (model intercept)	40.15 (8.18)	[22.60, 57.71]	4.91 (< 0.001)	Not applied (model intercept)
Education level	Up to 12th grade vs. *college graduate or above*	–6.83 (2.07)	[–11.26, –2.39]	–3.30 (0.005)	10.46 (2.65) vs. *17.29 (2.34)*
	High school graduate vs. *college graduate or above*	–3.84 (1.05)	[–6.09, –1.58]	–3.65 (0.003)	13.45 (1.78) vs. *17.29 (2.34)*
	Some college or AA degree vs. *college graduate or above*	–1.23 (1.80)	[–5.10, 2.63]	–0.68 (0.506)	16.06 (1.93) vs. *17.29 (2.34)*
Gender	Males vs. *females*	1.37 (1.11)	[–1.02, 3.76]	1.23 (0.238)	15.00 (1.53) vs. *13.63 (2.47)*
Race	Mexican American vs. *Non–Hispanic White*	–1.93 (1.59)	[–5.33, 1.47]	–1.22 (0.243)	10.96 (2.42) vs. *12.89 (1.95)*
	Other Hispanic vs. *Non–Hispanic White*	10.27 (1.30)	[7.49, 13.05]	7.92 (< 0.001)	23.16 (1.80) vs. *12.89 (1.95)*
	Non–Hispanic Black vs. *Non–Hispanic White*	1.16 (1.21)	[–1.44, 3.75]	0.95 (0.356)	14.05 (2.78) vs. *12.89 (1.95)*
	Non–Hispanic Asian vs. *Non–Hispanic White*	–2.39 (1.48)	[–5.56, 0.77]	–1.62 (0.127)	10.50 (2.25) vs. *12.89 (1.95)*
	Other Race and Multi–Racial vs. *Non–Hispanic White*	No cases	No cases	No cases	No cases
Marital status	Single or never married vs. *married or living with a partner*	–10.79 (2.60)	[–16.37, –5.20]	–4.14 (0.001)	6.91 (3.08) vs. *17.70 (2.05)*
	Divorced or separated vs. *married or living with a partner*	–2.47 (2.07)	[–6.90, 1.96]	–1.20 (0.252)	15.23 (2.47) vs. *17.70 (2.05)*
	Widowed vs. *married or living with a partner*	–0.28 (1.27)	[–3.01, 2.46]	–0.22 (0.832)	17.42 (1.98) vs. *17.70 (2.05)*
Self–report general health condition	Poor vs. *excellent or very good*	No cases	No cases	No cases	No cases
	Good or fair vs. *excellent or very good*	–0.57 (0.97)	[–2.65, 1.51]	–0.59 (0.566)	14.03 (1.79) vs. *14.60 (2.25)*
Difficulties in thinking or remembering	Yes vs. *no*	–0.45 (1.65)	[–3.98, 3.09]	–0.27 (0.791)	14.09 (2.44) vs. *14.54 (1.79)*
Ever told you have a heart disease	Yes vs. *no*	–0.51 (1.17)	[–3.02, 2.01]	–0.43 (0.673)	14.06 (2.51) vs. *14.57 (1.49)*
Ever told you had a stroke	Yes vs. *no*	–4.52 (0.90)	[–6.46, –2.59]	–5.01 (< 0.001)	12.05 (2.21) vs. *16.58 (1.82)*
Ever told you have diabetes	Yes vs. *no*	2.99 (1.62)	[–0.48, 6.46]	1.85 (0.086)	15.81 (2.12) vs. *12.82 (2.15)*
Have smoked at least 100 cigarettes in life	Yes vs. *no*	–0.72 (1.15)	[–3.17, 1.74]	–0.62 (0.542)	13.96 (2.04) vs. *14.67 (2.07)*
Three criteria combination: TRI + HBP + GLY	TRI + HBP + GLY vs. *metabolically–healthy people*	–3.50 (2.28)	[–8.38, 1.39]	–1.53 (0.147)	12.57 (2.88) vs. *16.06 (1.45)*
Age at screening	Not applied (continuous covariate)	–0.26 (0.10)	[–0.47, –0.05]	–2.68 (0.018)	Not applied (continuous cov.)
Ratio of family income to the poverty level	Not applied (continuous covariate)	0.12 (0.45)	[–0.84, 1.09]	0.28 (0.785)	Not applied (continuous cov.)
Depression raw scores	Not applied (continuous covariate)	–0.22 (0.19)	[–0.63, 0.20]	–1.12 (0.283)	Not applied (continuous cov.)

[a]The design–based covariance matrix is singular. The validity of results is uncertain. [b]The column labeled "estimated marginal means" shows each category's marginal means against the reference category's marginal mean. The reference categories and the marginal means of the reference categories are shown in italics. The significant results have been colored.

Table S57. Multiple linear regression model explaining the relationship between AFT and specific combination HDL + HBP + GLY after adjusting by sociodemographic characteristics and medical history, and estimated marginal means for each category of predictors and covariates[a]

**Model coefficient**	**Categories (for categorical predictor and covariates)**	*Beta* (*SE*)	95% CI	*t* (*p*)	Estimated marginal means[b]
Constant	Not applied (model intercept)	39.04 (8.88)	[20.21, 57.86]	4.40 (< 0.001)	Not applied (model intercept)
Education level	Up to 12th grade vs. *college graduate or above*	–6.57 (2.23)	[–11.30, –1.84]	–2.94 (0.010)	11.09 (2.01) vs. *17.65 (2.35)*
	High school graduate vs. *college graduate or above*	–3.46 (1.25)	[–6.11, –0.80]	–2.76 (0.014)	14.20 (1.60) vs. *17.65 (2.35)*
	Some college or AA degree vs. *college graduate or above*	–1.67 (1.79)	[–5.46, 2.12]	–0.93 (0.365)	15.98 (1.46) vs. *17.65 (2.35)*
Gender	Males vs. *females*	1.46 (1.10)	[–0.87, 3.78]	1.33 (0.203)	15.46 (1.32) vs. *14.00 (1.99)*
Race	Mexican American vs. *Non–Hispanic White*	–2.06 (1.60)	[–5.45, 1.33]	–1.29 (0.216)	12.90 (2.01) vs. *14.96 (1.12)*
	Other Hispanic vs. *Non–Hispanic White*	4.82 (2.71)	[–0.93, 10.57]	1.78 (0.095)	19.78 (3.04) vs. *14.96 (1.12)*
	Non–Hispanic Black vs. *Non–Hispanic White*	–0.04 (1.29)	[–2.79, 2.70]	–0.03 (0.974)	14.92 (1.85) vs. *14.96 (1.12)*
	Non–Hispanic Asian vs. *Non–Hispanic White*	–3.86 (2.20)	[–8.53, 0.81]	–1.75 (0.099)	11.10 (2.61) vs. *14.96 (1.12)*
	Other Race and Multi–Racial vs. *Non–Hispanic White*	No cases	No cases	No cases	No cases
Marital status	Single or never married vs. *married or living with a partner*	–10.01 (2.90)	[–16.16, –3.85]	–3.45 (0.003)	8.01 (2.77) vs. *18.02 (1.91)*
	Divorced or separated vs. *married or living with a partner*	–2.70 (2.16)	[–7.28, 1.87]	–1.25 (0.228)	15.31 (2.15) vs. *18.02 (1.91)*
	Widowed vs. *married or living with a partner*	–0.43 (1.49)	[–3.59, 2.73]	–0.29 (0.776)	17.59 (2.12) vs. *18.02 (1.91)*
Self–report general health condition	Poor vs. *excellent or very good*	1.12 (3.20)	[–5.66, 7.90]	0.35 (0.730)	15.61 (2.48) vs. *14.48 (2.27)*
	Good or fair vs. *excellent or very good*	–0.38 (1.22)	[–2.96, 2.20]	–0.31 (0.760)	14.10 (1.74) vs. *14.48 (2.27)*
Difficulties in thinking or remembering	Yes vs. *no*	0.07 (1.69)	[–3.52, 3.65]	0.04 (0.969)	14.76 (2.19) vs. *14.70 (1.31)*
Ever told you have a heart disease	Yes vs. *no*	–1.14 (1.22)	[–3.73, 1.45]	–0.93 (0.364)	14.16 (2.05) vs. *15.30 (1.28)*
Ever told you had a stroke	Yes vs. *no*	–3.97 (0.95)	[–5.98, –1.96]	–4.19 (0.001)	12.75 (1.82) vs. *16.71 (1.49)*
Ever told you have diabetes	Yes vs. *no*	5.90 (2.09)	[1.47, 10.32]	2.83 (0.012)	17.68 (1.64) vs. *11.78 (2.14)*
Have smoked at least 100 cigarettes in life	Yes vs. *no*	–0.60 (1.14)	[–3.02, 1.82]	–0.52 (0.608)	14.43 (1.87) vs. *15.03 (1.49)*
Three criteria combination: HDL + HBP + GLY	HDL + HBP + GLY vs. *metabolically–healthy people*	–4.11 (1.55)	[–7.39, –0.83]	–2.66 (0.017)	12.67 (2.12) vs. *16.79 (1.35)*
Age at screening	Not applied (continuous covariate)	–0.24 (0.11)	[–0.47, –0.02]	–2.27 (0.038)	Not applied (continuous cov.)
Ratio of family income to the poverty level	Not applied (continuous covariate)	0.12 (0.45)	[–0.84, 1.07]	0.26 (0.798)	Not applied (continuous cov.)
Depression raw scores	Not applied (continuous covariate)	–0.25 (0.21)	[–0.69, 0.19]	–1.20 (0.246)	Not applied (continuous cov.)

[a]The design–based covariance matrix is singular. The validity of results is uncertain. [b]The column labeled "estimated marginal means" shows each category's marginal means against the reference category's marginal mean. The reference categories and the marginal means of the reference categories are shown in italics. The significant results have been colored.

Table S58. Multiple linear regression model explaining the relationship between AFT and all combinations of three criteria after adjusting by sociodemographic characteristics and medical history, and estimated marginal means for each category of predictors and covariates

**Model coefficient**	**Categories (for categorical predictor and covariates)**	*Beta* (*SE*)	95% CI	*t* (*p*)	Estimated marginal means[a]
Constant	Not applied (model intercept)	33.13 (3.24)	[26.52, 39.74]	10.21 (< 0.001)	Not applied (model intercept)
Education level	Up to 12th grade vs. *college graduate or above*	–3.80 (0.93)	[–5.69, –1.90]	–4.09 (< 0.001)	13.26 (1.45) vs. *17.06 (1.31)*
	High school graduate vs. *college graduate or above*	–2.89 (0.73)	[–4.38, –1.40]	–3.94 (< 0.001)	14.17 (1.25) vs. *17.06 (1.31)*
	Some college or AA degree vs. *college graduate or above*	–1.64 (0.74)	[–3.14, –0.14]	–2.22 (0.033)	15.42 (1.31) vs. *17.06 (1.31)*
Gender	Males vs. *females*	–0.49 (0.67)	[–1.86, 0.88]	–0.73 (0.471)	14.73 (1.39) vs. *15.22 (1.19)*
Race	Mexican American vs. *Non–Hispanic White*	–1.49 (1.08)	[–3.70, 0.72]	–1.38 (0.178)	15.24 (1.43) vs. *16.73 (1.14)*
	Other Hispanic vs. *Non–Hispanic White*	–0.31 (1.19)	[–2.73, 2.12]	–0.26 (0.797)	16.42 (1.44) vs. *16.73 (1.14)*
	Non–Hispanic Black vs. *Non–Hispanic White*	–2.23 (0.71)	[–3.68, –0.78]	–3.13 (0.004)	14.50 (1.09) vs. *16.73 (1.14)*
	Non–Hispanic Asian vs. *Non–Hispanic White*	–3.34 (1.08)	[–5.54, –1.15]	–3.10 (0.004)	13.39 (1.54) vs. *16.73 (1.14)*
	Other Race and Multi–Racial vs. *Non–Hispanic White*	–3.14 (2.05)	[–7.32, 1.04]	–1.53 (0.135)	13.59 (2.64) vs. *16.73 (1.14)*
Marital status	Single or never married vs. *married or living with a partner*	–2.16 (2.09)	[–6.42, 2.10]	–1.03 (0.309)	13.88 (2.32) vs. *16.05 (1.25)*
	Divorced or separated vs. *married or living with a partner*	–0.47 (0.71)	[–1.90, 0.97]	–0.66 (0.514)	15.58 (1.26) vs. *16.05 (1.25)*
	Widowed vs. *married or living with a partner*	–1.64 (0.99)	[–3.66, 0.38]	–1.66 (0.107)	14.40 (1.53) vs. *16.05 (1.25)*
Self–report general health condition	Poor vs. *excellent or very good*	0.77 (1.87)	[–3.03, 4.57]	0.41 (0.683)	15.70 (2.24) vs. *14.93 (1.00)*
	Good or fair vs. *excellent or very good*	–0.62 (0.58)	[–1.79, 0.56]	–1.07 (0.292)	14.31 (1.07) vs. *14.93 (1.00)*
Difficulties in thinking or remembering	Yes vs. *no*	–1.12 (1.06)	[–3.27, 1.03]	–1.06 (0.297)	14.42 (1.55) vs. *15.54 (1.13)*
Ever told you have a heart disease	Yes vs. *no*	–1.72 (1.13)	[–4.02, 0.58]	–1.52 (0.138)	14.12 (1.59) vs. *15.84 (1.10)*
Ever told you had a stroke	Yes vs. *no*	0.71 (1.20)	[–1.74, 3.16]	0.59 (0.561)	15.33 (1.41) vs. *14.62 (1.36)*
Ever told you have diabetes	Yes vs. *no*	–1.77 (0.70)	[–3.21, –0.34]	–2.52 (0.017)	14.09 (1.31) vs. *15.86 (1.28)*
Have smoked at least 100 cigarettes in life	Yes vs. *no*	–0.29 (0.61)	[–1.54, 0.95]	–0.48 (0.634)	14.83 (1.17) vs. *15.12 (1.39)*
All combinations of three criteria	All combinations of three criteria vs. *metabolically–healthy people*	–0.48 (0.85)	[–2.20, 1.25]	–0.56 (0.577)	14.74 (1.24) vs. *15.22 (1.39)*
Age at screening	Not applied (continuous covariate)	–0.16 (0.05)	[–0.25, –0.06]	–3.44 (0.002)	Not applied (continuous cov.)
Ratio of family income to the poverty level	Not applied (continuous covariate)	0.03 (0.21)	[–0.38, 0.45]	0.17 (0.869)	Not applied (continuous cov.)
Depression raw scores	Not applied (continuous covariate)	–0.02 (0.06)	[–0.13, 0.10]	–0.27 (0.787)	Not applied (continuous cov.)

[a]The column labeled "estimated marginal means" shows each category's marginal means against the reference category's marginal mean. The reference categories and the marginal means of the reference categories are shown in italics. The significant results have been colored.

Table S59. Multiple linear regression model explaining the relationship between AFT and specific combination AO + TRI + HDL + HBP after adjusting by sociodemographic characteristics and medical history, and estimated marginal means for each category of predictors and covariates[a]

**Model coefficient**	**Categories (for categorical predictor and covariates)**	*Beta (SE)*	95% CI	*t (p)*	Estimated marginal means[b]
Constant	Not applied (model intercept)	37.00 (9.43)	[16.64, 57.37]	3.93 (0.002)	Not applied (model intercept)
Education level	Up to 12th grade vs. *college graduate or above*	–6.99 (1.89)	[–11.08, –2.89]	–3.69 (0.003)	9.24 (1.36) vs. *16.23 (1.81)*
	High school graduate vs. *college graduate or above*	–2.73 (1.28)	[–5.49, 0.03]	–2.14 (0.052)	13.50 (0.95) vs. *16.23 (1.81)*
	Some college or AA degree vs. *college graduate or above*	–1.25 (1.59)	[–4.69, 2.19]	–0.78 (0.447)	14.98 (1.17) vs. *16.23 (1.81)*
Gender	Males vs. *females*	1.22 (1.01)	[–0.97, 3.41]	1.21 (0.250)	14.10 (0.85) vs. *12.87 (1.37)*
Race	Mexican American vs. *Non–Hispanic White*	–1.83 (1.42)	[–4.90, 1.23]	–1.29 (0.218)	11.10 (1.35) vs. *12.94 (1.13)*
	Other Hispanic vs. *Non–Hispanic White*	9.35 (1.40)	[6.32, 12.38]	6.67 (< 0.001)	22.29 (1.26) vs. *12.94 (1.13)*
	Non–Hispanic Black vs. *Non–Hispanic White*	–0.91 (1.17)	[–3.43, 1.61]	–0.78 (0.451)	12.03 (1.60) vs. *12.94 (1.13)*
	Non–Hispanic Asian vs. *Non–Hispanic White*	–3.87 (1.30)	[–6.67, –1.06]	–2.98 (0.011)	9.07 (1.84) vs. *12.94 (1.13)*
	Other Race and Multi–Racial vs. *Non–Hispanic White*	No cases	No cases	No cases	No cases
Marital status	Single or never married vs. *married or living with a partner*	–11.03 (2.07)	[–15.51, –6.55]	–5.32 (< 0.001)	5.72 (2.11) vs. *16.75 (1.28)*
	Divorced or separated vs. *married or living with a partner*	–1.89 (2.00)	[–6.21, 2.43]	–0.95 (0.361)	14.86 (1.06) vs. *16.75 (1.28)*
	Widowed vs. *married or living with a partner*	–0.15 (0.84)	[–1.96, 1.66]	–0.18 (0.864)	16.61 (1.77) vs. *16.75 (1.28)*
Self–report general health condition	Poor vs. *excellent or very good*	–7.98 (2.89)	[–14.23, –1.73]	–2.76 (0.016)	8.20 (2.37) vs. *16.18 (1.43)*
	Good or fair vs. *excellent or very good*	–0.11 (0.97)	[–2.20, 1.98]	–0.12 (0.910)	16.07 (0.81) vs. *16.18 (1.43)*
Difficulties in thinking or remembering	Yes vs. *no*	–1.09 (1.43)	[–4.17, 1.99]	–0.76 (0.459)	12.94 (1.42) vs. *14.03 (1.05)*
Ever told you have a heart disease	Yes vs. *no*	–1.71 (0.92)	[–3.69, 0.28]	–1.86 (0.086)	12.63 (1.37) vs. *14.34 (0.80)*
Ever told you had a stroke	Yes vs. *no*	–1.11 (2.54)	[–6.59, 4.37]	–0.44 (0.669)	12.93 (1.74) vs. *14.04 (1.51)*
Ever told you have diabetes	Yes vs. *no*	5.11 (2.76)	[–0.86, 11.07]	1.85 (0.087)	16.04 (1.80) vs. *10.93 (1.63)*
Have smoked at least 100 cigarettes in life	Yes vs. *no*	–1.21 (0.94)	[–3.23, 0.82]	–1.29 (0.220)	12.88 (1.16) vs. *14.09 (1.09)*
Four criteria combination: AO + TRI + HDL + HBP	AO + TRI + HDL + HBP vs. *metabolically–healthy people*	–2.62 (1.30)	[–5.43, 0.19]	–2.02 (0.065)	12.17 (1.03) vs. *14.80 (1.37)*
Age at screening	Not applied (continuous covariate)	–0.22 (0.12)	[–0.46, 0.03]	–1.87 (0.084)	Not applied (continuous cov.)
Ratio of family income to the poverty level	Not applied (continuous covariate)	0.09 (0.44)	[–0.86, 1.04]	0.20 (0.843)	Not applied (continuous cov.)
Depression raw scores	Not applied (continuous covariate)	–0.11 (0.09)	[–0.31, 0.09]	–1.17 (0.264)	Not applied (continuous cov.)

[a]The design–based covariance matrix is singular. The validity of results is uncertain. [b]The column labeled "estimated marginal means" shows each category's marginal means against the reference category's marginal mean. The reference categories and the marginal means of the reference categories are shown in italics. The significant results have been colored.

Table S60. Multiple linear regression model explaining the relationship between AFT and specific combination AO + TRI + HDL + GLY after adjusting by sociodemographic characteristics and medical history, and estimated marginal means for each category of predictors and covariates[a]

**Model coefficient**	**Categories (for categorical predictor and covariates)**	*Beta* (*SE*)	95% CI	*t* (*p*)	Estimated marginal means[b]
Constant	Not applied (model intercept)	43.78 (6.12)	[30.98, 56.58]	7.16 (< 0.001)	Not applied (model intercept)
Education level	Up to 12th grade vs. *college graduate or above*	–3.63 (1.51)	[–6.78, –0.47]	–2.40 (0.027)	14.72 (2.29) vs. *18.35 (2.06)*
	High school graduate vs. *college graduate or above*	–1.03 (1.29)	[–3.73, 1.68]	–0.79 (0.437)	17.32 (1.87) vs. *18.35 (2.06)*
	Some college or AA degree vs. *college graduate or above*	0.40 (1.52)	[–2.79, 3.59]	0.26 (0.796)	18.75 (1.48) vs. *18.35 (2.06)*
Gender	Males vs. *females*	–1.20 (1.14)	[–3.57, 1.18]	–1.05 (0.306)	16.69 (1.68) vs. *17.88 (1.97)*
Race	Mexican American vs. *Non–Hispanic White*	–3.00 (0.90)	[–4.88, –1.13]	–3.35 (0.003)	15.07 (2.01) vs. *18.08 (2.04)*
	Other Hispanic vs. *Non–Hispanic White*	0.39 (2.36)	[–4.56, 5.33]	0.16 (0.871)	18.46 (2.19) vs. *18.08 (2.04)*
	Non–Hispanic Black vs. *Non–Hispanic White*	–0.77 (1.34)	[–3.58, 2.04]	–0.57 (0.573)	17.31 (2.18) vs. *18.08 (2.04)*
	Non–Hispanic Asian vs. *Non–Hispanic White*	–3.06 (1.68)	[–6.57, 0.45]	–1.83 (0.084)	15.01 (2.30) vs. *18.08 (2.04)*
	Other Race and Multi–Racial vs. *Non–Hispanic White*	1.71 (1.10)	[–0.60, 4.02]	1.55 (0.137)	19.79 (2.10) vs. *18.08 (2.04)*
Marital status	Single or never married vs. *married or living with a partner*	–3.77 (2.95)	[–9.95, 2.40]	–1.28 (0.217)	14.46 (3.47) vs. *18.23 (1.34)*
	Divorced or separated vs. *married or living with a partner*	1.26 (1.23)	[–1.32, 3.84]	1.02 (0.321)	19.49 (1.74) vs. *18.23 (1.34)*
	Widowed vs. *married or living with a partner*	–1.27 (1.16)	[–3.69, 1.15]	–1.10 (0.285)	16.96 (2.07) vs. *18.23 (1.34)*
Self–report general health condition	Poor vs. *excellent or very good*	–5.39 (2.67)	[–10.98, 0.20]	–2.02 (0.058)	14.07 (2.97) vs. *19.47 (1.75)*
	Good or fair vs. *excellent or very good*	–1.15 (0.97)	[–3.18, 0.89]	–1.18 (0.253)	18.32 (1.40) vs. *19.47 (1.75)*
Difficulties in thinking or remembering	Yes vs. *no*	2.78 (1.64)	[–0.66, 6.21]	1.69 (0.107)	18.67 (2.30) vs. *15.90 (1.46)*
Ever told you have a heart disease	Yes vs. *no*	0.94 (0.78)	[–0.70, 2.58]	1.19 (0.247)	17.75 (1.81) vs. *16.82 (1.76)*
Ever told you had a stroke	Yes vs. *no*	1.30 (1.27)	[–1.35, 3.95]	1.03 (0.317)	17.94 (2.11) vs. *16.63 (1.56)*
Ever told you have diabetes	Yes vs. *no*	–0.52 (1.05)	[–2.73, 1.68]	–0.50 (0.625)	17.02 (2.13) vs. *17.55 (1.44)*
Have smoked at least 100 cigarettes in life	Yes vs. *no*	–0.61 (0.86)	[–2.41, 1.19]	–0.71 (0.487)	16.98 (1.77) vs. *17.59 (1.82)*
Four criteria combination: AO + TRI + HDL + GLY	AO + TRI + HDL + GLY vs. *metabolically–healthy people*	0.06 (0.94)	[–1.92, 2.03]	0.06 (0.954)	17.31 (1.48) vs. *17.26 (2.08)*
Age at screening	Not applied (continuous covariate)	–0.32 (0.09)	[–0.51, –0.13]	–3.58 (0.002)	Not applied (continuous cov.)
Ratio of family income to the poverty level	Not applied (continuous covariate)	–0.02 (0.17)	[–0.38, 0.34]	–0.11 (0.911)	Not applied (continuous cov.)
Depression raw scores	Not applied (continuous covariate)	–0.30 (0.14)	[–0.59, 0.00]	–2.07 (0.052)	Not applied (continuous cov.)

[a]The design–based covariance matrix is singular. The validity of results is uncertain. [b]The column labeled "estimated marginal means" shows each category's marginal means against the reference category's marginal mean. The reference categories and the marginal means of the reference categories are shown in italics. The significant results have been colored.

Table S61. Multiple linear regression model explaining the relationship between AFT and specific combination AO + TRI + HBP + GLY after adjusting by sociodemographic characteristics and medical history, and estimated marginal means for each category of predictors and covariates[a]

**Model coefficient**	**Categories (for categorical predictor and covariates)**	*Beta* (*SE*)	95% CI	*t* (*p*)	Estimated marginal means[b]
Constant	Not applied (model intercept)	39.28 (6.98)	[24.87, 53.69]	5.63 (< 0.001)	Not applied (model intercept)
Education level	Up to 12th grade vs. *college graduate or above*	–4.10 (1.76)	[–7.73, –0.47]	–2.33 (0.029)	11.48 (1.80) vs. *15.58 (1.80)*
	High school graduate vs. *college graduate or above*	–3.70 (1.68)	[–7.17, –0.24]	–2.21 (0.037)	11.88 (1.12) vs. *15.58 (1.80)*
	Some college or AA degree vs. *college graduate or above*	–0.64 (1.42)	[–3.57, 2.30]	–0.45 (0.657)	14.95 (1.76) vs. *15.58 (1.80)*
Gender	Males vs. *females*	–0.46 (0.90)	[–2.31, 1.39]	–0.52 (0.611)	13.24 (1.34) vs. *13.71 (1.42)*
Race	Mexican American vs. *Non–Hispanic White*	–3.23 (1.17)	[–5.65, –0.81]	–2.75 (0.011)	11.46 (1.34) vs. *14.68 (1.37)*
	Other Hispanic vs. *Non–Hispanic White*	3.95 (1.19)	[1.49, 6.40]	3.32 (0.003)	18.63 (1.42) vs. *14.68 (1.37)*
	Non–Hispanic Black vs. *Non–Hispanic White*	–3.25 (1.13)	[–5.58, –0.92]	–2.87 (0.008)	11.43 (1.79) vs. *14.68 (1.37)*
	Non–Hispanic Asian vs. *Non–Hispanic White*	–3.52 (1.62)	[–6.85, –0.18]	–2.18 (0.039)	11.17 (1.88) vs. *14.68 (1.37)*
	Other Race and Multi–Racial vs. *Non–Hispanic White*	No cases	No cases	No cases	No cases
Marital status	Single or never married vs. *married or living with a partner*	–9.98 (2.67)	[–15.48, –4.47]	–3.74 (0.001)	6.44 (2.96) vs. *16.42 (1.10)*
	Divorced or separated vs. *married or living with a partner*	–1.30 (1.69)	[–4.79, 2.20]	–0.77 (0.451)	15.12 (1.86) vs. *16.42 (1.10)*
	Widowed vs. *married or living with a partner*	–0.51 (0.88)	[–2.33, 1.30]	–0.59 (0.564)	15.91 (1.19) vs. *16.42 (1.10)*
Self–report general health condition	Poor vs. *excellent or very good*	–3.39 (2.60)	[–8.77, 1.99]	–1.30 (0.205)	11.15 (2.38) vs. *14.54 (1.67)*
	Good or fair vs. *excellent or very good*	0.19 (0.92)	[–1.71, 2.09]	0.21 (0.837)	14.73 (1.23) vs. *14.54 (1.67)*
Difficulties in thinking or remembering	Yes vs. *no*	–0.92 (2.07)	[–5.20, 3.35]	–0.45 (0.660)	13.01 (1.73) vs. *13.94 (1.60)*
Ever told you have a heart disease	Yes vs. *no*	–0.88 (1.25)	[–3.46, 1.70]	–0.70 (0.488)	13.03 (1.70) vs. *13.91 (1.13)*
Ever told you had a stroke	Yes vs. *no*	–0.32 (1.71)	[–3.84, 3.20]	–0.19 (0.851)	13.31 (1.55) vs. *13.64 (1.56)*
Ever told you have diabetes	Yes vs. *no*	0.41 (2.00)	[–3.72, 4.54]	0.21 (0.839)	13.68 (1.90) vs. *13.27 (1.33)*
Have smoked at least 100 cigarettes in life	Yes vs. *no*	–0.15 (1.25)	[–2.73, 2.44]	–0.12 (0.908)	13.40 (1.56) vs. *13.55 (1.32)*
Four criteria combination: AO + TRI + HBP + GLY	AO + TRI + HBP + GLY vs. *metabolically–healthy people*	0.17 (0.89)	[–1.68, 2.01]	0.19 (0.852)	13.56 (1.33) vs. *13.39 (1.42)*
Age at screening	Not applied (continuous covariate)	–0.25 (0.09)	[–0.43, –0.07]	–2.87 (0.008)	Not applied (continuous cov.)
Ratio of family income to the poverty level	Not applied (continuous covariate)	–0.04 (0.34)	[–0.75, 0.66]	–0.13 (0.901)	Not applied (continuous cov.)
Depression raw scores	Not applied (continuous covariate)	–0.03 (0.05)	[–0.13, 0.07]	–0.64 (0.527)	Not applied (continuous cov.)

[a]The design–based covariance matrix is singular. The validity of results is uncertain. [b]The column labeled "estimated marginal means" shows each category's marginal means against the reference category's marginal mean. The reference categories and the marginal means of the reference categories are shown in italics. The significant results have been colored.

Table S62. Multiple linear regression model explaining the relationship between AFT and specific combination AO + HDL + HBP + GLY after adjusting by sociodemographic characteristics and medical history, and estimated marginal means for each category of predictors and covariates[a]

**Model coefficient**	**Categories (for categorical predictor and covariates)**	*Beta* (*SE*)	95% CI	*t* (*p*)	Estimated marginal means[b]
Constant	Not applied (model intercept)	43.81 (7.60)	[27.84, 59.78]	5.76 (< 0.001)	Not applied (model intercept)
Education level	Up to 12th grade vs. *college graduate or above*	–5.79 (1.26)	[–8.43, –3.14]	–4.60 (< 0.001)	11.21 (2.27) vs. *17.00 (1.37)*
	High school graduate vs. *college graduate or above*	–3.73 (1.13)	[–6.11, –1.34]	–3.29 (0.004)	13.28 (1.79) vs. *17.00 (1.37)*
	Some college or AA degree vs. *college graduate or above*	–0.34 (1.26)	[–2.99, 2.31]	–0.27 (0.789)	16.66 (1.68) vs. *17.00 (1.37)*
Gender	Males vs. *females*	1.30 (0.81)	[–0.41, 3.01]	1.59 (0.128)	15.19 (1.57) vs. *13.89 (1.78)*
Race	Mexican American vs. *Non–Hispanic White*	–5.71 (1.29)	[–8.42, –3.01]	–4.43 (< 0.001)	11.02 (1.81) vs. *16.73 (2.10)*
	Other Hispanic vs. *Non–Hispanic White*	1.97 (1.55)	[–1.29, 5.23]	1.27 (0.221)	18.70 (1.95) vs. *16.73 (2.10)*
	Non–Hispanic Black vs. *Non–Hispanic White*	–1.28 (1.40)	[–4.22, 1.67]	–0.91 (0.375)	15.46 (2.47) vs. *16.73 (2.10)*
	Non–Hispanic Asian vs. *Non–Hispanic White*	–2.01 (1.45)	[–5.06, 1.05]	–1.38 (0.184)	14.73 (2.34) vs. *16.73 (2.10)*
	Other Race and Multi–Racial vs. *Non–Hispanic White*	–6.15 (2.73)	[–11.88, –0.42]	–2.25 (0.037)	10.59 (1.86) vs. *16.73 (2.10)*
Marital status	Single or never married vs. *married or living with a partner*	–1.64 (2.99)	[–7.94, 4.65]	–0.55 (0.590)	13.28 (2.96) vs. *14.92 (1.46)*
	Divorced or separated vs. *married or living with a partner*	–0.56 (1.30)	[–3.29, 2.17]	–0.43 (0.672)	14.36 (1.96) vs. *14.92 (1.46)*
	Widowed vs. *married or living with a partner*	0.68 (1.38)	[–2.23, 3.58]	0.49 (0.630)	15.60 (2.26) vs. *14.92 (1.46)*
Self–report general health condition	Poor vs. *excellent or very good*	3.59 (3.47)	[–3.70, 10.88]	1.03 (0.314)	16.96 (3.45) vs. *13.37 (1.67)*
	Good or fair vs. *excellent or very good*	–0.09 (1.01)	[–2.21, 2.02]	–0.09 (0.926)	13.28 (1.43) vs. *13.37 (1.67)*
Difficulties in thinking or remembering	Yes vs. *no*	0.22 (1.24)	[–2.39, 2.83]	0.18 (0.863)	14.65 (2.01) vs. *14.43 (1.42)*
Ever told you have a heart disease	Yes vs. *no*	–1.64 (1.12)	[–3.99, 0.70]	–1.47 (0.158)	13.72 (1.80) vs. *15.36 (1.64)*
Ever told you had a stroke	Yes vs. *no*	–1.83 (2.29)	[–6.64, 2.97]	–0.80 (0.433)	13.62 (2.50) vs. *15.46 (1.29)*
Ever told you have diabetes	Yes vs. *no*	–4.02 (1.22)	[–6.59, –1.46]	–3.29 (0.004)	12.53 (1.78) vs. *16.55 (1.70)*
Have smoked at least 100 cigarettes in life	Yes vs. *no*	–1.43 (0.99)	[–3.51, 0.65]	–1.45 (0.165)	13.82 (1.71) vs. *15.25 (1.70)*
Four criteria combination: AO + HDL + HBP + GLY	AO + HDL + HBP + GLY vs. *metabolically–healthy people*	1.12 (1.37)	[–1.76, 4.01]	0.82 (0.425)	15.10 (1.73) vs. *13.98 (1.80)*
Age at screening	Not applied (continuous covariate)	–0.31 (0.10)	[–0.52, –0.10]	–3.11 (0.006)	Not applied (continuous cov.)
Ratio of family income to the poverty level	Not applied (continuous covariate)	–0.24 (0.33)	[–0.94, 0.46]	–0.73 (0.474)	Not applied (continuous cov.)
Depression raw scores	Not applied (continuous covariate)	–0.13 (0.12)	[–0.37, 0.12]	–1.10 (0.287)	Not applied (continuous cov.)

[a]The design–based covariance matrix is singular. The validity of results is uncertain. [b]The column labeled "estimated marginal means" shows each category's marginal means against the reference category's marginal mean. The reference categories and the marginal means of the reference categories are shown in italics. The significant results have been colored.

Table S63. Multiple linear regression model explaining the relationship between AFT and specific combination TRI + HDL + HBP + GLY after adjusting by sociodemographic characteristics and medical history, and estimated marginal means for each category of predictors and covariates[a]

**Model coefficient**	**Categories (for categorical predictor and covariates)**	*Beta (SE)*	95% CI	*t (p)*	Estimated marginal means[b]
Constant	Not applied (model intercept)	39.59 (9.10)	[19.94, 59.24]	4.35 (0.001)	Not applied (model intercept)
Education level	Up to 12th grade vs. *college graduate or above*	−6.74 (2.20)	[−11.50, −1.98]	−3.06 (0.009)	11.00 (2.89) vs. *17.74 (1.78)*
	High school graduate vs. *college graduate or above*	−3.45 (1.18)	[−6.00, −0.90]	−2.93 (0.012)	14.29 (1.56) vs. *17.74 (1.78)*
	Some college or AA degree vs. *college graduate or above*	−1.20 (1.82)	[−5.12, 2.73]	−0.66 (0.521)	16.55 (2.10) vs. *17.74 (1.78)*
Gender	Males vs. *females*	1.07 (1.08)	[−1.25, 3.40]	1.00 (0.336)	15.43 (1.64) vs. *14.36 (2.21)*
Race	Mexican American vs. *Non–Hispanic White*	−1.89 (1.61)	[−5.37, 1.59]	−1.17 (0.262)	11.80 (1.43) vs. *13.69 (1.55)*
	Other Hispanic vs. *Non–Hispanic White*	10.12 (1.29)	[7.33, 12.91]	7.85 (< 0.001)	23.81 (1.44) vs. *13.69 (1.55)*
	Non–Hispanic Black vs. *Non–Hispanic White*	0.74 (1.34)	[−2.17, 3.64]	0.55 (0.592)	14.43 (2.43) vs. *13.69 (1.55)*
	Non–Hispanic Asian vs. *Non–Hispanic White*	−2.92 (2.04)	[−7.34, 1.50]	−1.43 (0.177)	10.77 (2.72) vs. *13.69 (1.55)*
	Other Race and Multi–Racial vs. *Non–Hispanic White*	1.20 (4.53)	[−8.59, 10.98]	0.26 (0.796)	14.88 (5.27) vs. *13.69 (1.55)*
Marital status	Single or never married vs. *married or living with a partner*	−10.61 (2.56)	[−16.14, −5.07]	−4.14 (0.001)	7.59 (3.33) vs. *18.20 (1.34)*
	Divorced or separated vs. *married or living with a partner*	−1.82 (2.24)	[−6.66, 3.02]	−0.81 (0.431)	16.38 (2.54) vs. *18.20 (1.34)*
	Widowed vs. *married or living with a partner*	−0.78 (1.16)	[−3.28, 1.71]	−0.68 (0.510)	17.42 (1.69) vs. *18.20 (1.34)*
Self–report general health condition	Poor vs. *excellent or very good*	8.42 (4.14)	[−0.52, 17.37]	2.03 (0.063)	20.60 (4.43) vs. *12.18 (1.57)*
	Good or fair vs. *excellent or very good*	−0.27 (0.96)	[−2.34, 1.79]	−0.28 (0.781)	11.91 (0.90) vs. *12.18 (1.57)*
Difficulties in thinking or remembering	Yes vs. *no*	−0.36 (1.64)	[−3.90, 3.19]	−0.22 (0.832)	14.72 (1.79) vs. *15.07 (2.26)*
Ever told you have a heart disease	Yes vs. *no*	−0.33 (1.19)	[−2.89, 2.23]	−0.28 (0.784)	14.73 (2.32) vs. *15.06 (1.53)*
Ever told you had a stroke	Yes vs. *no*	−4.01 (0.84)	[−5.81, −2.20]	−4.80 (< 0.001)	12.89 (2.04) vs. *16.90 (1.78)*
Ever told you have diabetes	Yes vs. *no*	3.37 (2.77)	[−2.61, 9.36]	1.22 (0.245)	16.58 (2.55) vs. *13.21 (2.08)*
Have smoked at least 100 cigarettes in life	Yes vs. *no*	−0.72 (1.17)	[−3.26, 1.81]	−0.62 (0.548)	14.53 (1.84) vs. *15.26 (2.07)*
Four criteria combination: TRI + HDL + HBP + GLY	TRI + HDL + HBP + GLY vs. *metabolically–healthy people*	−8.92 (3.73)	[−16.99, −0.85]	−2.39 (0.033)	10.44 (1.99) vs. *19.36 (3.16)*
Age at screening	Not applied (continuous covariate)	−0.25 (0.11)	[−0.49, −0.01]	−2.29 (0.039)	Not applied (continuous cov.)
Ratio of family income to the poverty level	Not applied (continuous covariate)	0.15 (0.47)	[−0.87, 1.18]	0.32 (0.755)	Not applied (continuous cov.)
Depression raw scores	Not applied (continuous covariate)	−0.32 (0.21)	[−0.77, 0.13]	−1.55 (0.144)	Not applied (continuous cov.)

[a]The design–based covariance matrix is singular. The validity of results is uncertain. [b]The column labeled "estimated marginal means" shows each category's marginal means against the reference category's marginal mean. The reference categories and the marginal means of the reference categories are shown in italics. The significant results have been colored.

Table S64. Multiple linear regression model explaining the relationship between AFT and all combinations of four criteria after adjusting by sociodemographic characteristics and medical history, and estimated marginal means for each category of predictors and covariates

**Model coefficient**	**Categories (for categorical predictor and covariates)**	*Beta* (*SE*)	95% CI	*t* (*p*)	Estimated marginal means[a]
Constant	Not applied (model intercept)	39.54 (4.86)	[29.57, 49.52]	8.13 (< 0.001)	Not applied (model intercept)
Education level	Up to 12th grade vs. *college graduate or above*	–2.61 (1.25)	[–5.17, –0.06]	–2.10 (0.046)	14.13 (1.40) vs. *16.74 (1.25)*
	High school graduate vs. *college graduate or above*	–2.09 (1.09)	[–4.33, 0.14]	–1.92 (0.065)	14.65 (1.14) vs. *16.74 (1.25)*
	Some college or AA degree vs. *college graduate or above*	0.90 (1.01)	[–1.16, 2.96]	0.89 (0.379)	17.64 (1.39) vs. *16.74 (1.25)*
Gender	Males vs. *females*	–0.64 (0.95)	[–2.59, 1.31]	–0.68 (0.505)	15.47 (1.16) vs. *16.11 (1.27)*
Race	Mexican American vs. *Non–Hispanic White*	–4.69 (1.30)	[–7.36, –2.02]	–3.61 (0.001)	13.15 (1.29) vs. *17.84 (1.23)*
	Other Hispanic vs. *Non–Hispanic White*	0.33 (1.09)	[–1.91, 2.57]	0.30 (0.765)	18.17 (1.79) vs. *17.84 (1.23)*
	Non–Hispanic Black vs. *Non–Hispanic White*	–3.40 (0.81)	[–5.05, –1.74]	–4.21 (< 0.001)	14.44 (1.41) vs. *17.84 (1.23)*
	Non–Hispanic Asian vs. *Non–Hispanic White*	–3.88 (1.15)	[–6.23, –1.53]	–3.38 (0.002)	13.96 (1.59) vs. *17.84 (1.23)*
	Other Race and Multi–Racial vs. *Non–Hispanic White*	–0.65 (2.07)	[–4.89, 3.59]	–0.31 (0.756)	17.19 (1.84) vs. *17.84 (1.23)*
Marital status	Single or never married vs. *married or living with a partner*	–0.54 (2.25)	[–5.16, 4.08]	–0.24 (0.812)	15.36 (2.51) vs. *15.90 (0.82)*
	Divorced or separated vs. *married or living with a partner*	0.40 (0.61)	[–0.85, 1.65]	0.66 (0.514)	16.30 (1.11) vs. *15.90 (0.82)*
	Widowed vs. *married or living with a partner*	–0.30 (0.83)	[–2.00, 1.40]	–0.36 (0.719)	15.60 (1.18) vs. *15.90 (0.82)*
Self–report general health condition	Poor vs. *excellent or very good*	–4.30 (1.32)	[–7.01, –1.58]	–3.24 (0.003)	13.07 (1.47) vs. *17.36 (1.27)*
	Good or fair vs. *excellent or very good*	–0.42 (0.69)	[–1.84, 1.00]	–0.61 (0.550)	16.95 (1.12) vs. *17.36 (1.27)*
Difficulties in thinking or remembering	Yes vs. *no*	1.14 (1.23)	[–1.39, 3.67]	0.93 (0.363)	16.36 (1.50) vs. *15.22 (1.00)*
Ever told you have a heart disease	Yes vs. *no*	–0.01 (1.00)	[–2.06, 2.03]	–0.01 (0.989)	15.79 (1.28) vs. *15.80 (1.17)*
Ever told you had a stroke	Yes vs. *no*	0.83 (1.00)	[–1.23, 2.89]	0.83 (0.415)	16.21 (1.38) vs. *15.38 (1.05)*
Ever told you have diabetes	Yes vs. *no*	–0.68 (0.85)	[–2.42, 1.06]	–0.80 (0.432)	15.45 (1.30) vs. *16.13 (1.08)*
Have smoked at least 100 cigarettes in life	Yes vs. *no*	–0.54 (1.04)	[–2.68, 1.59]	–0.52 (0.605)	15.52 (1.37) vs. *16.06 (1.08)*
All combinations of four criteria	All combinations of four criteria vs. *metabolically–healthy people*	–0.22 (0.93)	[–2.13, 1.69]	–0.23 (0.818)	15.68 (1.00) vs. *15.90 (1.39)*
Age at screening	Not applied (continuous covariate)	–0.27 (0.07)	[–0.41, –0.13]	–3.89 (0.001)	Not applied (continuous cov.)
Ratio of family income to the poverty level	Not applied (continuous covariate)	–0.10 (0.22)	[–0.56, 0.36]	–0.45 (0.658)	Not applied (continuous cov.)
Depression raw scores	Not applied (continuous covariate)	–0.09 (0.06)	[–0.22, 0.04]	–1.48 (0.150)	Not applied (continuous cov.)

[a]The column labeled "estimated marginal means" shows each category's marginal means against the reference category's marginal mean. The reference categories and the marginal means of the reference categories are shown in italics. The significant results have been colored.

Table S65. Multiple linear regression model explaining the relationship between AFT and combination of five criteria after adjusting by sociodemographic characteristics and medical history, and estimated marginal means for each category of predictors and covariates[a]

**Model coefficient**	**Categories (for categorical predictor and covariates)**	*Beta* (*SE*)	95% CI	*t* (*p*)	Estimated marginal means[b]
Constant	Not applied (model intercept)	35.15 (5.79)	[23.07, 47.22]	6.07 (< 0.001)	Not applied (model intercept)
Education level	Up to 12th grade vs. *college graduate or above*	−7.63 (1.45)	[−10.66, −4.61]	−5.26 (< 0.001)	13.58 (2.25) vs. *21.22 (1.88)*
	High school graduate vs. *college graduate or above*	−4.20 (1.09)	[−6.48, −1.92]	−3.84 (0.001)	17.02 (1.45) vs. *21.22 (1.88)*
	Some college or AA degree vs. *college graduate or above*	−2.45 (1.35)	[−5.26, 0.37]	−1.81 (0.085)	18.77 (1.92) vs. *21.22 (1.88)*
Gender	Males vs. *females*	0.70 (0.90)	[−1.18, 2.58]	0.78 (0.445)	18.00 (1.60) vs. *17.30 (1.96)*
Race	Mexican American vs. *Non–Hispanic White*	0.25 (0.69)	[−1.19, 1.69]	0.36 (0.722)	17.49 (1.40) vs. *17.24 (1.81)*
	Other Hispanic vs. *Non–Hispanic White*	1.57 (1.46)	[−1.47, 4.61]	1.08 (0.295)	18.81 (2.11) vs. *17.24 (1.81)*
	Non–Hispanic Black vs. *Non–Hispanic White*	1.90 (2.00)	[−2.27, 6.07]	0.95 (0.353)	19.14 (2.63) vs. *17.24 (1.81)*
	Non–Hispanic Asian vs. *Non–Hispanic White*	−1.69 (1.99)	[−5.85, 2.47]	−0.85 (0.406)	15.55 (2.68) vs. *17.24 (1.81)*
	Other Race and Multi–Racial vs. *Non–Hispanic White*	0.00 (0.00)	No cases	No cases	No cases
Marital status	Single or never married vs. *married or living with a partner*	−7.84 (4.25)	[−16.72, 1.03]	−1.84 (0.080)	12.47 (4.38) vs. *20.31 (1.41)*
	Divorced or separated vs. *married or living with a partner*	−1.53 (1.51)	[−4.69, 1.62]	−1.01 (0.323)	18.78 (1.91) vs. *20.31 (1.41)*
	Widowed vs. *married or living with a partner*	−1.27 (0.73)	[−2.80, 0.25]	−1.74 (0.097)	19.04 (1.44) vs. *20.31 (1.41)*
Self–report general health condition	Poor vs. *excellent or very good*	8.47 (2.29)	[3.70, 13.24]	3.70 (0.001)	23.19 (2.74) vs. *14.71 (1.61)*
	Good or fair vs. *excellent or very good*	0.33 (0.76)	[−1.25, 1.92]	0.44 (0.667)	15.04 (1.58) vs. *14.71 (1.61)*
Difficulties in thinking or remembering	Yes vs. *no*	1.82 (1.21)	[−0.72, 4.35]	1.50 (0.150)	18.56 (1.90) vs. *16.74 (1.76)*
Ever told you have a heart disease	Yes vs. *no*	−0.61 (0.93)	[−2.55, 1.34]	−0.65 (0.523)	17.34 (1.79) vs. *17.95 (1.80)*
Ever told you had a stroke	Yes vs. *no*	−2.64 (2.14)	[−7.10, 1.82]	−1.24 (0.231)	16.33 (2.48) vs. *18.97 (1.46)*
Ever told you have diabetes	Yes vs. *no*	−0.03 (0.89)	[−1.88, 1.82]	−0.03 (0.973)	17.63 (1.89) vs. *17.66 (1.67)*
Have smoked at least 100 cigarettes in life	Yes vs. *no*	0.39 (0.73)	[−1.14, 1.91]	0.53 (0.603)	17.84 (1.86) vs. *17.45 (1.67)*
Combination of five criteria	AO + TRI + HDL + HBP + GLY vs. *metabolically–healthy people*	−1.61 (0.97)	[−3.64, 0.42]	−1.65 (0.114)	16.84 (1.70) vs. *18.45 (1.89)*
Age at screening	Not applied (continuous covariate)	−0.21 (0.07)	[−0.34, −0.07]	−3.11 (0.006)	Not applied (continuous cov.)
Ratio of family income to the poverty level	Not applied (continuous covariate)	0.36 (0.30)	[−0.28, 0.99]	1.17 (0.256)	Not applied (continuous cov.)
Depression raw scores	Not applied (continuous covariate)	−0.12 (0.11)	[−0.36, 0.11]	−1.11 (0.279)	Not applied (continuous cov.)

[a]The design–based covariance matrix is singular. The validity of results is uncertain. [b]The column labeled "estimated marginal means" shows each category's marginal means against the reference category's marginal mean. The reference categories and the marginal means of the reference categories are shown in italics. The significant results have been colored.

Table S66. Multiple linear regression model explaining the relationship between AFT and all combination with abdominal obesity after adjusting by sociodemographic characteristics and medical history, and estimated marginal means for each category of predictors and covariates

**Model coefficient**	**Categories (for categorical predictor and covariates)**	*Beta* (*SE*)	95% CI	*t* (*p*)	Estimated marginal means[a]
Constant	Not applied (model intercept)	33.98 (2.64)	[28.60, 39.36]	12.89 (< 0.001)	Not applied (model intercept)
Education level	Up to 12th grade vs. *college graduate or above*	–3.56 (0.72)	[–5.03, –2.09]	–4.94 (< 0.001)	15.18 (1.25) vs. *18.75 (1.10)*
	High school graduate vs. *college graduate or above*	–2.42 (0.59)	[–3.63, –1.22]	–4.11 (< 0.001)	16.32 (1.11) vs. *18.75 (1.10)*
	Some college or AA degree vs. *college graduate or above*	–1.13 (0.65)	[–2.46, 0.20]	–1.74 (0.092)	17.61 (1.32) vs. *18.75 (1.10)*
Gender	Males vs. *females*	–0.44 (0.58)	[–1.62, 0.75]	–0.75 (0.460)	16.75 (1.16) vs. *17.18 (1.18)*
Race	Mexican American vs. *Non–Hispanic White*	–2.41 (1.01)	[–4.47, –0.35]	–2.39 (0.023)	16.12 (1.34) vs. *18.53 (1.10)*
	Other Hispanic vs. *Non–Hispanic White*	–1.21 (0.95)	[–3.15, 0.72]	–1.28 (0.211)	17.32 (1.30) vs. *18.53 (1.10)*
	Non–Hispanic Black vs. *Non–Hispanic White*	–2.73 (0.46)	[–3.67, –1.80]	–5.95 (< 0.001)	15.80 (1.07) vs. *18.53 (1.10)*
	Non–Hispanic Asian vs. *Non–Hispanic White*	–3.45 (0.80)	[–5.08, –1.83]	–4.33 (< 0.001)	15.08 (1.24) vs. *18.53 (1.10)*
	Other Race and Multi–Racial vs. *Non–Hispanic White*	0.42 (1.87)	[–3.39, 4.23]	0.23 (0.823)	18.95 (2.21) vs. *18.53 (1.10)*
Marital status	Single or never married vs. *married or living with a partner*	0.23 (1.56)	[–2.95, 3.42]	0.15 (0.882)	17.46 (1.92) vs. *17.22 (1.15)*
	Divorced or separated vs. *married or living with a partner*	–0.05 (0.61)	[–1.30, 1.20]	–0.08 (0.934)	17.17 (1.24) vs. *17.22 (1.15)*
	Widowed vs. *married or living with a partner*	–1.21 (0.72)	[–2.68, 0.25]	–1.69 (0.100)	16.01 (1.17) vs. *17.22 (1.15)*
Self–report general health condition	Poor vs. *excellent or very good*	1.94 (1.76)	[–1.64, 5.53]	1.10 (0.278)	18.38 (2.07) vs. *16.44 (0.82)*
	Good or fair vs. *excellent or very good*	–0.36 (0.46)	[–1.29, 0.56]	–0.80 (0.430)	16.08 (0.96) vs. *16.44 (0.82)*
Difficulties in thinking or remembering	Yes vs. *no*	–0.25 (0.96)	[–2.21, 1.71]	–0.26 (0.795)	16.84 (1.42) vs. *17.09 (1.01)*
Ever told you have a heart disease	Yes vs. *no*	–0.67 (0.83)	[–2.37, 1.04]	–0.80 (0.431)	16.63 (1.35) vs. *17.30 (1.03)*
Ever told you had a stroke	Yes vs. *no*	0.53 (1.07)	[–1.65, 2.72]	0.50 (0.622)	17.23 (1.36) vs. *16.70 (1.13)*
Ever told you have diabetes	Yes vs. *no*	–1.45 (0.62)	[–2.71, –0.20]	–2.36 (0.025)	16.24 (1.19) vs. *17.69 (1.15)*
Have smoked at least 100 cigarettes in life	Yes vs. *no*	–0.29 (0.54)	[–1.40, 0.82]	–0.53 (0.598)	16.82 (1.11) vs. *17.11 (1.21)*
All combinations with abdominal obesity	Combinations with AO vs. *metabolically–healthy people*	–0.44 (0.86)	[–2.18, 1.31]	–0.51 (0.613)	16.75 (1.10) vs. *17.18 (1.31)*
Age at screening	Not applied (continuous covariate)	–0.18 (0.04)	[–0.26, –0.11]	–4.86 (< 0.001)	Not applied (continuous cov.)
Ratio of family income to the poverty level	Not applied (continuous covariate)	0.09 (0.17)	[–0.27, 0.44]	0.51 (0.616)	Not applied (continuous cov.)
Depression raw scores	Not applied (continuous covariate)	–0.03 (0.04)	[–0.11, 0.04]	–0.95 (0.352)	Not applied (continuous cov.)

[a]The column labeled "estimated marginal means" shows each category's marginal means against the reference category's marginal mean. The reference categories and the marginal means of the reference categories are shown in italics. The significant results have been colored.

Table S67. Multiple linear regression model explaining the relationship between AFT and all combinations without abdominal obesity after adjusting by sociodemographic characteristics and medical history, and estimated marginal means for each category of predictors and covariates[a]

**Model coefficient**	**Categories (for categorical predictor and covariates)**	*Beta* (*SE*)	95% CI	*t* (*p*)	Estimated marginal means[b]
Constant	Not applied (model intercept)	37.55 (7.40)	[22.12, 52.98]	5.08 (< 0.001)	Not applied (model intercept)
Education level	Up to 12th grade vs. *college graduate or above*	–4.50 (1.51)	[–7.64, –1.35]	–2.98 (0.007)	10.91 (1.66) vs. *15.41 (1.73)*
	High school graduate vs. *college graduate or above*	–3.43 (1.02)	[–5.55, –1.31]	–3.38 (0.003)	11.97 (1.35) vs. *15.41 (1.73)*
	Some college or AA degree vs. *college graduate or above*	–0.31 (1.37)	[–3.17, 2.55]	–0.23 (0.822)	15.09 (1.35) vs. *15.41 (1.73)*
Gender	Males vs. *females*	1.49 (1.13)	[–0.85, 3.84]	1.33 (0.200)	14.09 (1.09) vs. *12.60 (1.71)*
Race	Mexican American vs. *Non–Hispanic White*	–3.32 (1.17)	[–5.76, –0.87]	–2.83 (0.010)	11.69 (1.65) vs. *15.01 (1.27)*
	Other Hispanic vs. *Non–Hispanic White*	2.16 (3.07)	[–4.23, 8.56]	0.71 (0.488)	17.17 (3.08) vs. *15.01 (1.27)*
	Non–Hispanic Black vs. *Non–Hispanic White*	–0.27 (1.19)	[–2.75, 2.20]	–0.23 (0.821)	14.74 (1.76) vs. *15.01 (1.27)*
	Non–Hispanic Asian vs. *Non–Hispanic White*	–4.03 (1.71)	[–7.61, –0.45]	–2.35 (0.029)	10.98 (2.15) vs. *15.01 (1.27)*
	Other Race and Multi–Racial vs. *Non–Hispanic White*	–4.53 (1.44)	[–7.52, –1.53]	–3.15 (0.005)	10.48 (1.73) vs. *15.01 (1.27)*
Marital status	Single or never married vs. *married or living with a partner*	–8.16 (2.77)	[–13.93, –2.39]	–2.95 (0.008)	7.71 (2.88) vs. *15.87 (1.42)*
	Divorced or separated vs. *married or living with a partner*	–0.87 (1.53)	[–4.05, 2.31]	–0.57 (0.574)	15.00 (1.70) vs. *15.87 (1.42)*
	Widowed vs. *married or living with a partner*	–1.06 (1.49)	[–4.17, 2.06]	–0.71 (0.488)	14.81 (1.41) vs. *15.87 (1.42)*
Self–report general health condition	Poor vs. *excellent or very good*	1.80 (2.19)	[–2.76, 6.36]	0.82 (0.421)	14.68 (1.50) vs. *12.88 (1.86)*
	Good or fair vs. *excellent or very good*	–0.41 (0.97)	[–2.42, 1.61]	–0.42 (0.678)	12.48 (1.55) vs. *12.88 (1.86)*
Difficulties in thinking or remembering	Yes vs. *no*	–0.34 (1.67)	[–3.82, 3.14]	–0.20 (0.840)	13.18 (1.88) vs. *13.52 (1.15)*
Ever told you have a heart disease	Yes vs. *no*	–1.50 (1.06)	[–3.71, 0.71]	–1.41 (0.173)	12.60 (1.70) vs. *14.10 (1.07)*
Ever told you had a stroke	Yes vs. *no*	–3.67 (1.24)	[–6.25, –1.09]	–2.97 (0.008)	11.51 (1.63) vs. *15.18 (1.26)*
Ever told you have diabetes	Yes vs. *no*	3.32 (1.65)	[–0.12, 6.77]	2.01 (0.058)	15.01 (1.53) vs. *11.68 (1.58)*
Have smoked at least 100 cigarettes in life	Yes vs. *no*	–0.72 (1.15)	[–3.13, 1.68]	–0.63 (0.538)	12.99 (1.46) vs. *13.71 (1.42)*
All combinations without abdominal obesity	Combinations without AO vs. *metabolically–healthy people*	–3.13 (1.26)	[–5.75, –0.50]	–2.48 (0.022)	11.78 (1.61) vs. *14.91 (1.30)*
Age at screening	Not applied (continuous covariate)	–0.22 (0.09)	[–0.40, –0.04]	–2.53 (0.020)	Not applied (continuous cov.)
Ratio of family income to the poverty level	Not applied (continuous covariate)	–0.15 (0.33)	[–0.85, 0.55]	–0.46 (0.652)	Not applied (continuous cov.)
Depression raw scores	Not applied (continuous covariate)	–0.37 (0.12)	[–0.63, –0.11]	–2.96 (0.008)	Not applied (continuous cov.)

[a]The design–based covariance matrix is singular. The validity of results is uncertain. [b]The column labeled "estimated marginal means" shows each category's marginal means against the reference category's marginal mean. The reference categories and the marginal means of the reference categories are shown in italics. The significant results have been colored.

Table S68. Multiple linear regression model explaining the relationship between AFT and all combinations with high glycemia obesity after adjusting by sociodemographic characteristics and medical history, and estimated marginal means for each category of predictors and covariates

**Model coefficient**	**Categories (for categorical predictor and covariates)**	*Beta* (*SE*)	95% CI	*t* (*p*)	Estimated marginal means[a]
Constant	Not applied (model intercept)	34.70 (2.78)	[29.05, 40.36]	12.50 (< 0.001)	Not applied (model intercept)
Education level	Up to 12th grade vs. *college graduate or above*	–3.45 (0.74)	[–4.96, –1.93]	–4.63 (< 0.001)	15.12 (1.39) vs. *18.57 (1.23)*
	High school graduate vs. *college graduate or above*	–2.73 (0.61)	[–3.97, –1.49]	–4.50 (< 0.001)	15.84 (1.27) vs. *18.57 (1.23)*
	Some college or AA degree vs. *college graduate or above*	–1.21 (0.70)	[–2.64, 0.23]	–1.72 (0.096)	17.36 (1.52) vs. *18.57 (1.23)*
Gender	Males vs. *females*	–0.66 (0.64)	[–1.96, 0.63]	–1.04 (0.305)	16.39 (1.28) vs. *17.05 (1.37)*
Race	Mexican American vs. *Non–Hispanic White*	–2.50 (1.03)	[–4.60, –0.40]	–2.42 (0.021)	16.03 (1.38) vs. *18.53 (1.17)*
	Other Hispanic vs. *Non–Hispanic White*	–1.45 (0.95)	[–3.38, 0.48]	–1.53 (0.137)	17.08 (1.41) vs. *18.53 (1.17)*
	Non–Hispanic Black vs. *Non–Hispanic White*	–2.78 (0.44)	[–3.68, –1.89]	–6.33 (< 0.001)	15.75 (1.16) vs. *18.53 (1.17)*
	Non–Hispanic Asian vs. *Non–Hispanic White*	–3.52 (0.79)	[–5.13, –1.91]	–4.45 (< 0.001)	15.01 (1.31) vs. *18.53 (1.17)*
	Other Race and Multi–Racial vs. *Non–Hispanic White*	–0.61 (2.30)	[–5.30, 4.08]	–0.27 (0.792)	17.92 (2.79) vs. *18.53 (1.17)*
Marital status	Single or never married vs. *married or living with a partner*	0.18 (1.55)	[–2.98, 3.33]	0.11 (0.910)	17.12 (2.01) vs. *16.95 (1.28)*
	Divorced or separated vs. *married or living with a partner*	0.07 (0.60)	[–1.16, 1.29]	0.11 (0.913)	17.01 (1.42) vs. *16.95 (1.28)*
	Widowed vs. *married or living with a partner*	–1.16 (0.71)	[–2.61, 0.30]	–1.62 (0.115)	15.79 (1.29) vs. *16.95 (1.28)*
Self–report general health condition	Poor vs. *excellent or very good*	1.59 (1.74)	[–1.95, 5.13]	0.92 (0.366)	17.91 (2.18) vs. *16.32 (0.96)*
	Good or fair vs. *excellent or very good*	–0.39 (0.47)	[–1.35, 0.57]	–0.83 (0.412)	15.93 (1.11) vs. *16.32 (0.96)*
Difficulties in thinking or remembering	Yes vs. *no*	0.03 (1.01)	[–2.02, 2.09]	0.03 (0.975)	16.74 (1.58) vs. *16.70 (1.15)*
Ever told you have a heart disease	Yes vs. *no*	–0.78 (0.86)	[–2.53, 0.97]	–0.91 (0.370)	16.33 (1.53) vs. *17.11 (1.15)*
Ever told you had a stroke	Yes vs. *no*	0.62 (1.12)	[–1.66, 2.90]	0.56 (0.581)	17.03 (1.57) vs. *16.41 (1.21)*
Ever told you have diabetes	Yes vs. *no*	–1.39 (0.67)	[–2.75, –0.03]	–2.08 (0.046)	16.02 (1.35) vs. *17.41 (1.31)*
Have smoked at least 100 cigarettes in life	Yes vs. *no*	–0.19 (0.58)	[–1.37, 0.98]	–0.34 (0.738)	16.62 (1.28) vs. *16.82 (1.35)*
All combinations with high glycemia	Combinations with GLY vs. *metabolically–healthy people*	–0.38 (0.87)	[–2.15, 1.38]	–0.44 (0.661)	16.53 (1.25) vs. *16.91 (1.46)*
Age at screening	Not applied (continuous covariate)	–0.19 (0.04)	[–0.27, –0.11]	–4.81 (< 0.001)	Not applied (continuous cov.)
Ratio of family income to the poverty level	Not applied (continuous covariate)	0.10 (0.18)	[–0.28, 0.47]	0.53 (0.599)	Not applied (continuous cov.)
Depression raw scores	Not applied (continuous covariate)	–0.05 (0.05)	[–0.14, 0.04]	–1.13 (0.268)	Not applied (continuous cov.)

[a]The column labeled "estimated marginal means" shows each category's marginal means against the reference category's marginal mean. The reference categories and the marginal means of the reference categories are shown in italics. The significant results have been colored.

Table S69. Multiple linear regression model explaining the relationship between AFT and all combinations without high glycemia obesity after adjusting by sociodemographic characteristics and medical history, and estimated marginal means for each category of predictors and covariates[a]

**Model coefficient**	**Categories (for categorical predictor and covariates)**	*Beta* (*SE*)	95% CI	*t* (*p*)	Estimated marginal means[b]
Constant	Not applied (model intercept)	34.03 (6.10)	[21.16, 46.89]	5.58 (< 0.001)	Not applied (model intercept)
Education level	Up to 12th grade vs. *college graduate or above*	−6.53 (1.23)	[−9.12, −3.95]	−5.32 (< 0.001)	11.36 (1.78) vs. *17.89 (2.28)*
	High school graduate vs. *college graduate or above*	−2.78 (1.27)	[−5.46, −0.09]	−2.18 (0.043)	15.11 (1.70) vs. *17.89 (2.28)*
	Some college or AA degree vs. *college graduate or above*	−0.43 (1.24)	[−3.05, 2.18]	−0.35 (0.730)	17.46 (1.84) vs. *17.89 (2.28)*
Gender	Males vs. *females*	1.02 (0.92)	[−0.92, 2.96]	1.11 (0.282)	15.96 (1.89) vs. *14.94 (1.83)*
Race	Mexican American vs. *Non–Hispanic White*	−2.23 (1.27)	[−4.90, 0.45]	−1.76 (0.097)	13.80 (2.20) vs. *16.03 (1.85)*
	Other Hispanic vs. *Non–Hispanic White*	4.09 (1.88)	[0.11, 8.06]	2.17 (0.045)	20.11 (1.91) vs. *16.03 (1.85)*
	Non–Hispanic Black vs. *Non–Hispanic White*	−0.34 (0.80)	[−2.03, 1.34]	−0.43 (0.673)	15.68 (1.99) vs. *16.03 (1.85)*
	Non–Hispanic Asian vs. *Non–Hispanic White*	−2.67 (1.27)	[−5.35, 0.01]	−2.11 (0.050)	13.35 (2.25) vs. *16.03 (1.85)*
	Other Race and Multi–Racial vs. *Non–Hispanic White*	−2.27 (1.36)	[−5.13, 0.60]	−1.67 (0.114)	13.76 (2.52) vs. *16.03 (1.85)*
Marital status	Single or never married vs. *married or living with a partner*	−9.88 (2.54)	[−15.25, −4.52]	−3.89 (0.001)	8.51 (2.95) vs. *18.39 (1.85)*
	Divorced or separated vs. *married or living with a partner*	−1.37 (1.33)	[−4.19, 1.44]	−1.03 (0.317)	17.02 (1.47) vs. *18.39 (1.85)*
	Widowed vs. *married or living with a partner*	−0.48 (0.79)	[−2.14, 1.17]	−0.62 (0.547)	17.91 (2.16) vs. *18.39 (1.85)*
Self–report general health condition	Poor vs. *excellent or very good*	3.45 (3.92)	[−4.82, 11.72]	0.88 (0.391)	17.74 (4.16) vs. *14.29 (1.20)*
	Good or fair vs. *excellent or very good*	0.03 (0.80)	[−1.66, 1.73]	0.04 (0.968)	14.33 (1.01) vs. *14.29 (1.20)*
Difficulties in thinking or remembering	Yes vs. *no*	−0.65 (0.97)	[−2.70, 1.39]	−0.67 (0.511)	15.13 (1.93) vs. *15.78 (1.80)*
Ever told you have a heart disease	Yes vs. *no*	−2.16 (0.68)	[−3.59, −0.72]	−3.17 (0.006)	14.38 (1.90) vs. *16.53 (1.76)*
Ever told you had a stroke	Yes vs. *no*	2.26 (1.55)	[−1.02, 5.54]	1.46 (0.164)	16.59 (2.36) vs. *14.32 (1.45)*
Ever told you have diabetes	Yes vs. *no*	−1.65 (1.52)	[−4.86, 1.56]	−1.09 (0.293)	14.63 (1.99) vs. *16.28 (1.92)*
Have smoked at least 100 cigarettes in life	Yes vs. *no*	−0.71 (0.82)	[−2.44, 1.01]	−0.87 (0.395)	15.10 (1.79) vs. *15.81 (1.90)*
All combinations without high glycemia	Combinations without GLY vs. *metabolically–healthy people*	−1.42 (0.95)	[−3.42, 0.58]	−1.50 (0.152)	14.75 (1.69) vs. *16.16 (2.02)*
Age at screening	Not applied (continuous covariate)	−0.17 (0.08)	[−0.33, −0.01]	−2.29 (0.035)	Not applied (continuous cov.)
Ratio of family income to the poverty level	Not applied (continuous covariate)	−0.11 (0.30)	[−0.75, 0.53]	−0.36 (0.724)	Not applied (continuous cov.)
Depression raw scores	Not applied (continuous covariate)	−0.09 (0.06)	[−0.22, 0.05]	−1.36 (0.191)	Not applied (continuous cov.)

[a]The design–based covariance matrix is singular. The validity of results is uncertain. [b]The column labeled "estimated marginal means" shows each category's marginal means against the reference category's marginal mean. The reference categories and the marginal means of the reference categories are shown in italics. The significant results have been colored.

Table S70. Multiple linear regression model explaining the relationship between DSST and classic MetS diagnosis after adjusting by sociodemographic characteristics and medical history, and estimated marginal means for each category of predictors and covariates

**Model coefficient**	**Categories (for categorical predictor and covariates)**	*Beta* (*SE*)	95% CI	*t* (*p*)	Estimated marginal means[a]
Constant	Not applied (model intercept)	127.94 (8.38)	[110.87, 145.01]	15.27 (< 0.001)	Not applied (model intercept)
Education level	Up to 12th grade vs. *college graduate or above*	–13.69 (2.06)	[–17.90, –9.49]	–6.63 (< 0.001)	32.11 (2.18) vs. *45.80 (2.71)*
	High school graduate vs. *college graduate or above*	–6.57 (2.24)	[–11.12, –2.01]	–2.94 (0.006)	39.23 (2.14) vs. *45.80 (2.71)*
	Some college or AA degree vs. *college graduate or above*	–4.55 (1.80)	[–8.22, –0.87]	–2.52 (0.017)	41.25 (1.81) vs. *45.80 (2.71)*
Gender	Males vs. *females*	–4.82 (1.39)	[–7.65, –1.99]	–3.46 (0.002)	37.19 (2.10) vs. *42.01 (1.96)*
Race	Mexican American vs. *Non–Hispanic White*	–12.40 (2.18)	[–16.83, –7.96]	–5.70 (< 0.001)	33.60 (2.71) vs. *45.99 (1.62)*
	Other Hispanic vs. *Non–Hispanic White*	–14.52 (2.25)	[–19.10, –9.94]	–6.46 (< 0.001)	31.47 (2.99) vs. *45.99 (1.62)*
	Non–Hispanic Black vs. *Non–Hispanic White*	–12.75 (1.78)	[–16.37, –9.14]	–7.18 (< 0.001)	33.24 (2.47) vs. *45.99 (1.62)*
	Non–Hispanic Asian vs. *Non–Hispanic White*	1.51 (2.05)	[–2.67, 5.69]	0.74 (0.467)	47.50 (2.68) vs. *45.99 (1.62)*
	Other Race and Multi–Racial vs. *Non–Hispanic White*	–0.21 (2.49)	[–5.28, 4.86]	–0.08 (0.933)	45.78 (3.00) vs. *45.99 (1.62)*
Marital status	Single or never married vs. *married or living with a partner*	0.55 (2.51)	[–4.56, 5.66]	0.22 (0.827)	40.48 (3.19) vs. *39.93 (2.15)*
	Divorced or separated vs. *married or living with a partner*	–0.32 (2.22)	[–4.83, 4.19]	–0.14 (0.887)	39.61 (2.64) vs. *39.93 (2.15)*
	Widowed vs. *married or living with a partner*	–1.56 (2.27)	[–6.18, 3.07]	–0.68 (0.498)	38.37 (2.46) vs. *39.93 (2.15)*
Self–report general health condition	Poor vs. *excellent or very good*	–1.38 (3.26)	[–8.01, 5.25]	–0.42 (0.675)	38.94 (3.18) vs. *40.32 (2.15)*
	Good or fair vs. *excellent or very good*	–0.79 (1.61)	[–4.08, 2.49]	–0.49 (0.626)	39.53 (1.79) vs. *40.32 (2.15)*
Difficulties in thinking or remembering	Yes vs. *no*	–7.96 (1.65)	[–11.33, –4.59]	–4.81 (< 0.001)	35.62 (2.35) vs. *43.58 (1.76)*
Ever told you have a heart disease	Yes vs. *no*	–0.81 (2.39)	[–5.68, 4.05]	–0.34 (0.736)	39.19 (2.64) vs. *40.00 (1.77)*
Ever told you had a stroke	Yes vs. *no*	–1.23 (2.14)	[–5.59, 3.13]	–0.57 (0.570)	38.98 (2.30) vs. *40.21 (2.06)*
Ever told you have diabetes	Yes vs. *no*	–3.03 (1.46)	[–6.00, –0.06]	–2.07 (0.046)	38.08 (2.08) vs. *41.11 (2.00)*
Have smoked at least 100 cigarettes in life	Yes vs. *no*	–1.35 (1.41)	[–4.22, 1.53]	–0.95 (0.348)	38.93 (1.96) vs. *40.27 (2.11)*
Classic MetS diagnosis (all three or more criteria)	MetS vs. *metabolically–healthy people*	3.40 (2.45)	[–1.59, 8.38]	1.39 (0.175)	41.30 (1.76) vs. *37.90 (2.68)*
Age at screening	Not applied (continuous covariate)	–0.95 (0.13)	[–1.22, –0.68]	–7.16 (< 0.001)	Not applied (continuous cov.)
Ratio of family income to the poverty level	Not applied (continuous covariate)	–0.10 (0.55)	[–1.23, 1.02]	–0.19 (0.853)	Not applied (continuous cov.)
Depression raw scores	Not applied (continuous covariate)	–0.23 (0.14)	[–0.50, 0.05]	–1.65 (0.109)	Not applied (continuous cov.)

[a]The column labeled "estimated marginal means" shows each category's marginal means against the reference category's marginal mean. The reference categories and the marginal means of the reference categories are shown in italics. The significant results have been colored.

Table S71. Multiple linear regression model explaining the relationship between DSST and specific combination AO + TRI + HDL after adjusting by sociodemographic characteristics and medical history, and estimated marginal means for each category of predictors and covariates[a]

**Model coefficient**	**Categories (for categorical predictor and covariates)**	*Beta* (*SE*)	95% CI	*t* (*p*)	Estimated marginal means[b]
Constant	Not applied (model intercept)	148.45 (14.32)	[117.51, 179.40]	10.36 (< 0.001)	Not applied (model intercept)
Education level	Up to 12th grade vs. *college graduate or above*	–11.55 (3.64)	[–19.42, –3.68]	–3.17 (0.007)	33.36 (3.80) vs. *44.91 (5.58)*
	High school graduate vs. *college graduate or above*	–6.52 (4.66)	[–16.58, 3.54]	–1.40 (0.185)	38.39 (4.12) vs. *44.91 (5.58)*
	Some college or AA degree vs. *college graduate or above*	–1.83 (2.48)	[–7.18, 3.52]	–0.74 (0.473)	43.08 (5.21) vs. *44.91 (5.58)*
Gender	Males vs. *females*	–8.72 (2.51)	[–14.15, –3.30]	–3.47 (0.004)	35.57 (4.39) vs. *44.30 (4.06)*
Race	Mexican American vs. *Non–Hispanic White*	–12.80 (3.15)	[–19.61, –5.99]	–4.06 (0.001)	32.33 (5.40) vs. *45.13 (3.07)*
	Other Hispanic vs. *Non–Hispanic White*	–14.88 (4.59)	[–24.78, –4.97]	–3.24 (0.006)	30.25 (5.12) vs. *45.13 (3.07)*
	Non–Hispanic Black vs. *Non–Hispanic White*	–11.39 (3.38)	[–18.69, –4.10]	–3.38 (0.005)	33.74 (4.22) vs. *45.13 (3.07)*
	Non–Hispanic Asian vs. *Non–Hispanic White*	13.08 (4.94)	[2.42, 23.75]	2.65 (0.020)	58.22 (6.65) vs. *45.13 (3.07)*
	Other Race and Multi–Racial vs. *Non–Hispanic White*	No cases	No cases	No cases	No cases
Marital status	Single or never married vs. *married or living with a partner*	–16.04 (6.75)	[–30.62, –1.46]	–2.38 (0.034)	26.17 (7.87) vs. *42.21 (2.95)*
	Divorced or separated vs. *married or living with a partner*	2.81 (3.15)	[–3.99, 9.61]	0.89 (0.389)	45.02 (4.04) vs. *42.21 (2.95)*
	Widowed vs. *married or living with a partner*	4.13 (3.35)	[–3.11, 11.37]	1.23 (0.239)	46.34 (5.51) vs. *42.21 (2.95)*
Self–report general health condition	Poor vs. *excellent or very good*	–10.55 (9.34)	[–30.74, 9.64]	–1.13 (0.279)	35.36 (4.76) vs. *45.91 (6.79)*
	Good or fair vs. *excellent or very good*	–7.39 (3.70)	[–15.39, 0.60]	–2.00 (0.067)	38.52 (6.40) vs. *45.91 (6.79)*
Difficulties in thinking or remembering	Yes vs. *no*	–3.97 (3.39)	[–11.28, 3.35]	–1.17 (0.263)	37.95 (4.78) vs. *41.92 (3.93)*
Ever told you have a heart disease	Yes vs. *no*	3.23 (6.47)	[–10.76, 17.21]	0.50 (0.626)	41.55 (6.57) vs. *38.32 (3.22)*
Ever told you had a stroke	Yes vs. *no*	3.76 (4.73)	[–6.47, 13.98]	0.79 (0.442)	41.81 (6.25) vs. *38.05 (2.18)*
Ever told you have diabetes	Yes vs. *no*	–8.54 (2.99)	[–14.99, –2.09]	–2.86 (0.013)	35.66 (3.50) vs. *44.20 (4.98)*
Have smoked at least 100 cigarettes in life	Yes vs. *no*	–0.13 (2.09)	[–4.64, 4.38]	–0.06 (0.952)	39.87 (3.66) vs. *40.00 (4.63)*
Three criteria combination: AO + TRI + HDL	AO + TRI + HDL vs. *metabolically–healthy people*	1.95 (5.92)	[–10.83, 14.73]	0.33 (0.747)	40.91 (6.32) vs. *38.96 (3.18)*
Age at screening	Not applied (continuous covariate)	–1.25 (0.18)	[–1.64, –0.86]	–6.98 (< 0.001)	Not applied (continuous cov.)
Ratio of family income to the poverty level	Not applied (continuous covariate)	–0.38 (0.79)	[–2.09, 1.33]	–0.48 (0.642)	Not applied (continuous cov.)
Depression raw scores	Not applied (continuous covariate)	0.31 (0.32)	[–0.39, 1.00]	0.95 (0.357)	Not applied (continuous cov.)

[a]The design–based covariance matrix is singular. The validity of results is uncertain. [b]The column labeled "estimated marginal means" shows each category's marginal means against the reference category's marginal mean. The reference categories and the marginal means of the reference categories are shown in italics. The significant results have been colored.

Table S72. Multiple linear regression model explaining the relationship between DSST and specific combination AO + TRI + HBP after adjusting by sociodemographic characteristics and medical history, and estimated marginal means for each category of predictors and covariates[a]

**Model coefficient**	**Categories (for categorical predictor and covariates)**	*Beta* (*SE*)	95% CI	*t* (*p*)	Estimated marginal means[b]
Constant	Not applied (model intercept)	166.25 (11.65)	[141.26, 191.24]	14.27 (< 0.001)	Not applied (model intercept)
Education level	Up to 12th grade vs. *college graduate or above*	–16.54 (4.85)	[–26.94, –6.14]	–3.41 (0.004)	35.10 (5.79) vs. *51.64 (7.50)*
	High school graduate vs. *college graduate or above*	–11.44 (4.64)	[–21.39, –1.49]	–2.47 (0.027)	40.20 (5.07) vs. *51.64 (7.50)*
	Some college or AA degree vs. *college graduate or above*	–5.66 (3.98)	[–14.20, 2.89]	–1.42 (0.177)	45.98 (5.90) vs. *51.64 (7.50)*
Gender	Males vs. *females*	–8.83 (2.84)	[–14.93, –2.73]	–3.10 (0.008)	38.81 (6.39) vs. *47.64 (5.05)*
Race	Mexican American vs. *Non–Hispanic White*	–12.73 (2.93)	[–19.02, –6.45]	–4.34 (0.001)	37.21 (5.22) vs. *49.95 (4.08)*
	Other Hispanic vs. *Non–Hispanic White*	–16.07 (11.34)	[–40.39, 8.25]	–1.42 (0.178)	33.88 (11.74) vs. *49.95 (4.08)*
	Non–Hispanic Black vs. *Non–Hispanic White*	–17.27 (4.21)	[–26.29, –8.24]	–4.10 (0.001)	32.68 (7.67) vs. *49.95 (4.08)*
	Non–Hispanic Asian vs. *Non–Hispanic White*	12.49 (4.58)	[2.66, 22.31]	2.73 (0.016)	62.43 (7.42) vs. *49.95 (4.08)*
	Other Race and Multi–Racial vs. *Non–Hispanic White*	No cases	No cases	No cases	No cases
Marital status	Single or never married vs. *married or living with a partner*	–18.51 (7.38)	[–34.34, –2.68]	–2.51 (0.025)	29.30 (9.48) vs. *47.81 (6.01)*
	Divorced or separated vs. *married or living with a partner*	–5.40 (5.44)	[–17.08, 6.28]	–0.99 (0.338)	42.41 (3.85) vs. *47.81 (6.01)*
	Widowed vs. *married or living with a partner*	5.60 (2.94)	[–0.70, 11.91]	1.91 (0.077)	53.41 (7.61) vs. *47.81 (6.01)*
Self–report general health condition	Poor vs. *excellent or very good*	No cases	No cases	No cases	No cases
	Good or fair vs. *excellent or very good*	–4.59 (2.90)	[–10.81, 1.64]	–1.58 (0.136)	40.94 (5.18) vs. *45.52 (6.29)*
Difficulties in thinking or remembering	Yes vs. *no*	–6.79 (3.14)	[–13.53, –0.05]	–2.16 (0.049)	39.83 (6.59) vs. *46.63 (4.87)*
Ever told you have a heart disease	Yes vs. *no*	2.40 (6.30)	[–11.10, 15.90]	0.38 (0.709)	44.43 (8.36) vs. *42.03 (3.49)*
Ever told you had a stroke	Yes vs. *no*	10.72 (4.32)	[1.45, 19.99]	2.48 (0.026)	48.59 (6.67) vs. *37.87 (5.20)*
Ever told you have diabetes	Yes vs. *no*	–2.38 (3.75)	[–10.43, 5.67]	–0.64 (0.535)	42.04 (5.18) vs. *44.42 (6.51)*
Have smoked at least 100 cigarettes in life	Yes vs. *no*	2.10 (3.30)	[–4.96, 9.17]	0.64 (0.533)	44.28 (5.07) vs. *42.18 (6.47)*
Three criteria combination: AO + TRI + HBP	AO + TRI + HBP vs. *metabolically–healthy people*	1.16 (4.53)	[–8.56, 10.87]	0.26 (0.802)	43.81 (7.55) vs. *42.65 (3.92)*
Age at screening	Not applied (continuous covariate)	–1.51 (0.15)	[–1.84, –1.18]	–9.87 (< 0.001)	Not applied (continuous cov.)
Ratio of family income to the poverty level	Not applied (continuous covariate)	–0.08 (0.99)	[–2.21, 2.06]	–0.08 (0.940)	Not applied (continuous cov.)
Depression raw scores	Not applied (continuous covariate)	0.70 (0.38)	[–0.11, 1.51]	1.85 (0.085)	Not applied (continuous cov.)

[a]The design–based covariance matrix is singular. The validity of results is uncertain. [b]The column labeled "estimated marginal means" shows each category's marginal means against the reference category's marginal mean. The reference categories and the marginal means of the reference categories are shown in italics. The significant results have been colored.

Table S73. Multiple linear regression model explaining the relationship between DSST and specific combination AO + TRI + GLY after adjusting by sociodemographic characteristics and medical history, and estimated marginal means for each category of predictors and covariates[a]

**Model coefficient**	**Categories (for categorical predictor and covariates)**	*Beta* (*SE*)	95% CI	*t* (*p*)	Estimated marginal means[b]
Constant	Not applied (model intercept)	138.45 (13.06)	[111.30, 165.60]	10.60 (< 0.001)	Not applied (model intercept)
Education level	Up to 12th grade vs. *college graduate or above*	–14.93 (3.73)	[–22.69, –7.17]	–4.00 (0.001)	40.87 (6.79) vs. *55.80 (8.81)*
	High school graduate vs. *college graduate or above*	–7.56 (4.09)	[–16.06, 0.94]	–1.85 (0.079)	48.25 (7.73) vs. *55.80 (8.81)*
	Some college or AA degree vs. *college graduate or above*	–3.60 (2.86)	[–9.54, 2.33]	–1.26 (0.221)	52.20 (8.85) vs. *55.80 (8.81)*
Gender	Males vs. *females*	–6.36 (3.34)	[–13.30, 0.58]	–1.90 (0.071)	46.10 (7.71) vs. *52.46 (8.18)*
Race	Mexican American vs. *Non–Hispanic White*	–10.62 (3.84)	[–18.60, –2.64]	–2.77 (0.012)	43.11 (9.18) vs. *53.73 (7.30)*
	Other Hispanic vs. *Non–Hispanic White*	–3.84 (5.75)	[–15.80, 8.11]	–0.67 (0.511)	49.89 (9.05) vs. *53.73 (7.30)*
	Non–Hispanic Black vs. *Non–Hispanic White*	–14.76 (2.88)	[–20.75, –8.76]	–5.12 (< 0.001)	38.97 (8.85) vs. *53.73 (7.30)*
	Non–Hispanic Asian vs. *Non–Hispanic White*	6.97 (4.65)	[–2.69, 16.64]	1.50 (0.149)	60.70 (8.09) vs. *53.73 (7.30)*
	Other Race and Multi–Racial vs. *Non–Hispanic White*	No cases	No cases	No cases	No cases
Marital status	Single or never married vs. *married or living with a partner*	–8.18 (6.95)	[–22.64, 6.28]	–1.18 (0.252)	43.11 (10.99) vs. *51.29 (7.13)*
	Divorced or separated vs. *married or living with a partner*	–1.42 (3.63)	[–8.97, 6.13]	–0.39 (0.700)	49.87 (7.22) vs. *51.29 (7.13)*
	Widowed vs. *married or living with a partner*	1.56 (2.90)	[–4.47, 7.59]	0.54 (0.596)	52.85 (8.44) vs. *51.29 (7.13)*
Self–report general health condition	Poor vs. *excellent or very good*	15.39 (9.74)	[–4.87, 35.66]	1.58 (0.129)	60.73 (12.90) vs. *45.33 (5.54)*
	Good or fair vs. *excellent or very good*	–3.55 (3.50)	[–10.83, 3.73]	–1.01 (0.322)	41.78 (6.52) vs. *45.33 (5.54)*
Difficulties in thinking or remembering	Yes vs. *no*	–5.78 (3.75)	[–13.59, 2.02]	–1.54 (0.138)	46.39 (8.77) vs. *52.17 (7.13)*
Ever told you have a heart disease	Yes vs. *no*	2.30 (6.54)	[–11.30, 15.89]	0.35 (0.729)	50.43 (10.20) vs. *48.13 (6.16)*
Ever told you had a stroke	Yes vs. *no*	1.82 (5.37)	[–9.34, 12.98]	0.34 (0.738)	50.19 (10.00) vs. *48.37 (5.93)*
Ever told you have diabetes	Yes vs. *no*	–5.39 (4.88)	[–15.53, 4.75]	–1.11 (0.282)	46.59 (8.86) vs. *51.98 (7.36)*
Have smoked at least 100 cigarettes in life	Yes vs. *no*	–2.10 (3.49)	[–9.35, 5.16]	–0.60 (0.555)	48.23 (7.35) vs. *50.33 (8.53)*
Three criteria combination: AO + TRI + GLY	AO + TRI + HBP vs. *metabolically–healthy people*	4.18 (3.32)	[–2.73, 11.09]	1.26 (0.222)	51.37 (8.54) vs. *47.19 (7.30)*
Age at screening	Not applied (continuous covariate)	–1.08 (0.20)	[–1.49, –0.67]	–5.53 (< 0.001)	Not applied (continuous cov.)
Ratio of family income to the poverty level	Not applied (continuous covariate)	–0.15 (0.95)	[–2.13, 1.84]	–0.15 (0.880)	Not applied (continuous cov.)
Depression raw scores	Not applied (continuous covariate)	–0.49 (0.53)	[–1.59, 0.61]	–0.92 (0.368)	Not applied (continuous cov.)

[a]The design–based covariance matrix is singular. The validity of results is uncertain. [b]The column labeled "estimated marginal means" shows each category's marginal means against the reference category's marginal mean. The reference categories and the marginal means of the reference categories are shown in italics. The significant results have been colored.

Table S74. Multiple linear regression model explaining the relationship between DSST and specific combination AO + HDL + HBP after adjusting by sociodemographic characteristics and medical history, and estimated marginal means for each category of predictors and covariates[a]

**Model coefficient**	**Categories (for categorical predictor and covariates)**	*Beta* (*SE*)	95% CI	*t* (*p*)	Estimated marginal means[b]
Constant	Not applied (model intercept)	161.24 (12.68)	[134.36, 188.11]	12.72 (< 0.001)	Not applied (model intercept)
Education level	Up to 12th grade vs. *college graduate or above*	–14.72 (3.31)	[–21.73, –7.71]	–4.45 (< 0.001)	28.69 (5.28) vs. *43.41 (6.20)*
	High school graduate vs. *college graduate or above*	–7.91 (4.69)	[–17.85, 2.04]	–1.68 (0.111)	35.50 (7.03) vs. *43.41 (6.20)*
	Some college or AA degree vs. *college graduate or above*	–1.18 (2.49)	[–6.45, 4.09]	–0.47 (0.641)	42.23 (5.07) vs. *43.41 (6.20)*
Gender	Males vs. *females*	–8.38 (2.43)	[–13.53, –3.23]	–3.45 (0.003)	33.27 (5.49) vs. *41.65 (5.64)*
Race	Mexican American vs. *Non–Hispanic White*	–13.44 (3.72)	[–21.32, –5.56]	–3.62 (0.002)	32.21 (6.47) vs. *45.65 (4.69)*
	Other Hispanic vs. *Non–Hispanic White*	–13.09 (5.85)	[–25.50, –0.69]	–2.24 (0.040)	32.56 (6.53) vs. *45.65 (4.69)*
	Non–Hispanic Black vs. *Non–Hispanic White*	–17.14 (3.17)	[–23.86, –10.42]	–5.41 (< 0.001)	28.51 (4.27) vs. *45.65 (4.69)*
	Non–Hispanic Asian vs. *Non–Hispanic White*	10.84 (4.52)	[1.27, 20.42]	2.40 (0.029)	56.49 (7.57) vs. *45.65 (4.69)*
	Other Race and Multi–Racial vs. *Non–Hispanic White*	–16.34 (7.78)	[–32.84, 0.15]	–2.10 (0.052)	29.31 (10.66) vs. *45.65 (4.69)*
Marital status	Single or never married vs. *married or living with a partner*	–17.66 (6.64)	[–31.73, –3.59]	–2.66 (0.017)	22.73 (9.32) vs. *40.39 (5.50)*
	Divorced or separated vs. *married or living with a partner*	–1.42 (3.78)	[–9.43, 6.59]	–0.38 (0.712)	38.97 (4.80) vs. *40.39 (5.50)*
	Widowed vs. *married or living with a partner*	7.33 (3.20)	[0.56, 14.11]	2.29 (0.036)	47.73 (5.65) vs. *40.39 (5.50)*
Self–report general health condition	Poor vs. *excellent or very good*	No cases	No cases	No cases	No cases
	Good or fair vs. *excellent or very good*	–5.57 (3.73)	[–13.48, 2.35]	–1.49 (0.155)	34.67 (6.15) vs. *40.24 (5.30)*
Difficulties in thinking or remembering	Yes vs. *no*	–8.18 (2.83)	[–14.17, –2.18]	–2.89 (0.011)	33.37 (5.71) vs. *41.54 (5.51)*
Ever told you have a heart disease	Yes vs. *no*	0.64 (6.01)	[–12.10, 13.38]	0.11 (0.917)	37.78 (7.30) vs. *37.14 (4.87)*
Ever told you had a stroke	Yes vs. *no*	–4.86 (7.08)	[–19.87, 10.15]	–0.69 (0.502)	35.02 (8.77) vs. *39.89 (2.68)*
Ever told you have diabetes	Yes vs. *no*	0.31 (5.92)	[–12.24, 12.85]	0.05 (0.959)	37.61 (6.48) vs. *37.30 (5.88)*
Have smoked at least 100 cigarettes in life	Yes vs. *no*	0.26 (3.33)	[–6.81, 7.33]	0.08 (0.938)	37.59 (4.92) vs. *37.32 (6.35)*
Three criteria combination: AO + HDL + HBP	AO + HDL + HBP vs. *metabolically–healthy people*	6.89 (3.02)	[0.49, 13.29]	2.28 (0.037)	40.90 (5.86) vs. *34.01 (5.40)*
Age at screening	Not applied (continuous covariate)	–1.45 (0.19)	[–1.86, –1.05]	–7.59 (< 0.001)	Not applied (continuous cov.)
Ratio of family income to the poverty level	Not applied (continuous covariate)	–0.39 (1.22)	[–2.99, 2.21]	–0.32 (0.755)	Not applied (continuous cov.)
Depression raw scores	Not applied (continuous covariate)	1.04 (0.48)	[0.02, 2.06]	2.17 (0.046)	Not applied (continuous cov.)

[a]The design–based covariance matrix is singular. The validity of results is uncertain. [b]The column labeled "estimated marginal means" shows each category's marginal means against the reference category's marginal mean. The reference categories and the marginal means of the reference categories are shown in italics. The significant results have been colored.

Table S75. Multiple linear regression model explaining the relationship between DSST and specific combination AO + HDL + GLY after adjusting by sociodemographic characteristics and medical history, and estimated marginal means for each category of predictors and covariates[a]

**Model coefficient**	**Categories (for categorical predictor and covariates)**	*Beta* (*SE*)	95% CI	*t* (*p*)	Estimated marginal means[b]
Constant	Not applied (model intercept)	116.18 (13.49)	[88.27, 144.08]	8.61 (< 0.001)	Not applied (model intercept)
Education level	Up to 12th grade vs. *college graduate or above*	–17.85 (3.63)	[–25.35, –10.34]	–4.92 (< 0.001)	20.29 (4.82) vs. *38.14 (4.19)*
	High school graduate vs. *college graduate or above*	–5.96 (3.78)	[–13.79, 1.86]	–1.58 (0.129)	32.17 (4.52) vs. *38.14 (4.19)*
	Some college or AA degree vs. *college graduate or above*	–4.91 (2.75)	[–10.60, 0.78]	–1.79 (0.087)	33.22 (4.16) vs. *38.14 (4.19)*
Gender	Males vs. *females*	–6.18 (2.62)	[–11.60, –0.77]	–2.36 (0.027)	27.86 (4.22) vs. *34.05 (3.50)*
Race	Mexican American vs. *Non–Hispanic White*	–13.60 (4.28)	[–22.45, –4.75]	–3.18 (0.004)	24.30 (4.15) vs. *37.90 (3.16)*
	Other Hispanic vs. *Non–Hispanic White*	–7.67 (4.03)	[–16.02, 0.67]	–1.90 (0.070)	30.23 (4.99) vs. *37.90 (3.16)*
	Non–Hispanic Black vs. *Non–Hispanic White*	–11.10 (3.58)	[–18.51, –3.69]	–3.10 (0.005)	26.80 (3.87) vs. *37.90 (3.16)*
	Non–Hispanic Asian vs. *Non–Hispanic White*	6.25 (4.62)	[–3.30, 15.81]	1.35 (0.189)	44.16 (6.19) vs. *37.90 (3.16)*
	Other Race and Multi–Racial vs. *Non–Hispanic White*	–15.57 (3.83)	[–23.49, –7.64]	–4.06 (< 0.001)	22.34 (5.60) vs. *37.90 (3.16)*
Marital status	Single or never married vs. *married or living with a partner*	–12.66 (6.32)	[–25.74, 0.42]	–2.00 (0.057)	22.17 (7.08) vs. *34.84 (2.91)*
	Divorced or separated vs. *married or living with a partner*	–2.10 (3.08)	[–8.48, 4.27]	–0.68 (0.502)	32.73 (3.89) vs. *34.84 (2.91)*
	Widowed vs. *married or living with a partner*	–0.76 (3.06)	[–7.09, 5.58]	–0.25 (0.807)	34.08 (4.80) vs. *34.84 (2.91)*
Self–report general health condition	Poor vs. *excellent or very good*	–7.71 (4.77)	[–17.58, 2.16]	–1.62 (0.120)	26.87 (5.20) vs. *34.58 (4.90)*
	Good or fair vs. *excellent or very good*	–3.16 (3.97)	[–11.38, 5.05]	–0.80 (0.434)	31.42 (2.64) vs. *34.58 (4.90)*
Difficulties in thinking or remembering	Yes vs. *no*	–4.57 (2.75)	[–10.25, 1.11]	–1.66 (0.110)	28.67 (4.09) vs. *33.24 (3.70)*
Ever told you have a heart disease	Yes vs. *no*	–6.08 (3.44)	[–13.19, 1.04]	–1.77 (0.091)	27.92 (4.85) vs. *33.99 (3.00)*
Ever told you had a stroke	Yes vs. *no*	–7.70 (3.36)	[–14.64, –0.75]	–2.29 (0.031)	27.11 (4.63) vs. *34.80 (3.29)*
Ever told you have diabetes	Yes vs. *no*	–2.70 (4.14)	[–11.28, 5.87]	–0.65 (0.521)	29.60 (3.93) vs. *32.31 (4.45)*
Have smoked at least 100 cigarettes in life	Yes vs. *no*	0.16 (2.39)	[–4.78, 5.11]	0.07 (0.946)	31.04 (3.46) vs. *30.87 (4.18)*
Three criteria combination: AO + HDL + GLY	AO + HDL + GLY vs. *metabolically–healthy people*	4.16 (2.59)	[–1.20, 9.52]	1.61 (0.122)	33.04 (3.78) vs. *28.88 (3.96)*
Age at screening	Not applied (continuous covariate)	–0.76 (0.19)	[–1.16, –0.37]	–4.01 (0.001)	Not applied (continuous cov.)
Ratio of family income to the poverty level	Not applied (continuous covariate)	0.11 (1.08)	[–2.13, 2.35]	0.10 (0.921)	Not applied (continuous cov.)
Depression raw scores	Not applied (continuous covariate)	–0.06 (0.47)	[–1.04, 0.91]	–0.14 (0.892)	Not applied (continuous cov.)

[a]The design–based covariance matrix is singular. The validity of results is uncertain. [b]The column labeled "estimated marginal means" shows each category's marginal means against the reference category's marginal mean. The reference categories and the marginal means of the reference categories are shown in italics. The significant results have been colored.

Table S76. Multiple linear regression model explaining the relationship between DSST and specific combination AO + HBP + GLY after adjusting by sociodemographic characteristics and medical history, and estimated marginal means for each category of predictors and covariates

**Model coefficient**	**Categories (for categorical predictor and covariates)**	*Beta* (*SE*)	95% CI	*t* (*p*)	Estimated marginal means[a]
Constant	Not applied (model intercept)	113.26 (10.26)	[92.28, 134.23]	11.04 (< 0.001)	Not applied (model intercept)
Education level	Up to 12th grade vs. *college graduate or above*	–12.42 (2.77)	[–18.08, –6.76]	–4.49 (< 0.001)	38.54 (3.20) vs. *50.97 (4.24)*
	High school graduate vs. *college graduate or above*	–9.35 (3.03)	[–15.53, –3.16]	–3.09 (0.004)	41.62 (3.96) vs. *50.97 (4.24)*
	Some college or AA degree vs. *college graduate or above*	–5.03 (2.75)	[–10.65, 0.59]	–1.83 (0.078)	45.94 (4.17) vs. *50.97 (4.24)*
Gender	Males vs. *females*	–5.61 (1.87)	[–9.43, –1.79]	–3.00 (0.005)	41.46 (3.68) vs. *47.07 (3.63)*
Race	Mexican American vs. *Non–Hispanic White*	–8.60 (2.95)	[–14.63, –2.58]	–2.92 (0.007)	39.85 (4.92) vs. *48.45 (3.47)*
	Other Hispanic vs. *Non–Hispanic White*	–8.67 (2.10)	[–12.95, –4.38]	–4.13 (< 0.001)	39.79 (4.20) vs. *48.45 (3.47)*
	Non–Hispanic Black vs. *Non–Hispanic White*	–10.26 (2.24)	[–14.84, –5.69]	–4.59 (< 0.001)	38.19 (3.74) vs. *48.45 (3.47)*
	Non–Hispanic Asian vs. *Non–Hispanic White*	8.76 (2.73)	[3.17, 14.35]	3.20 (0.003)	57.21 (3.74) vs. *48.45 (3.47)*
	Other Race and Multi–Racial vs. *Non–Hispanic White*	–6.35 (2.25)	[–10.96, –1.74]	–2.81 (0.009)	42.11 (3.99) vs. *48.45 (3.47)*
Marital status	Single or never married vs. *married or living with a partner*	–3.17 (6.45)	[–16.36, 10.03]	–0.49 (0.627)	43.17 (6.91) vs. *46.34 (3.38)*
	Divorced or separated vs. *married or living with a partner*	1.56 (2.53)	[–3.61, 6.73]	0.62 (0.541)	47.90 (3.51) vs. *46.34 (3.38)*
	Widowed vs. *married or living with a partner*	–6.68 (1.94)	[–10.66, –2.70]	–3.43 (0.002)	39.66 (4.23) vs. *46.34 (3.38)*
Self–report general health condition	Poor vs. *excellent or very good*	6.13 (9.18)	[–12.64, 24.91]	0.67 (0.509)	49.15 (9.14) vs. *43.02 (2.57)*
	Good or fair vs. *excellent or very good*	–2.39 (2.44)	[–7.38, 2.59]	–0.98 (0.335)	40.63 (2.66) vs. *43.02 (2.57)*
Difficulties in thinking or remembering	Yes vs. *no*	–7.82 (1.79)	[–11.49, –4.16]	–4.37 (< 0.001)	40.35 (3.82) vs. *48.18 (3.46)*
Ever told you have a heart disease	Yes vs. *no*	4.85 (3.43)	[–2.16, 11.86]	1.42 (0.168)	46.69 (4.24) vs. *41.84 (3.58)*
Ever told you had a stroke	Yes vs. *no*	0.44 (2.05)	[–3.76, 4.64]	0.21 (0.832)	44.49 (4.00) vs. *44.05 (3.32)*
Ever told you have diabetes	Yes vs. *no*	–6.16 (1.83)	[–9.91, –2.41]	–3.36 (0.002)	41.19 (3.63) vs. *47.35 (3.67)*
Have smoked at least 100 cigarettes in life	Yes vs. *no*	–0.15 (1.85)	[–3.94, 3.64]	–0.08 (0.936)	44.19 (3.69) vs. *44.34 (3.61)*
Three criteria combination: AO + HBP + GLY	AO + HBP + GLY vs. *metabolically–healthy people*	2.60 (1.85)	[–1.18, 6.38]	1.40 (0.171)	45.57 (3.62) vs. *42.97 (3.68)*
Age at screening	Not applied (continuous covariate)	–0.74 (0.14)	[–1.04, –0.45]	–5.19 (< 0.001)	Not applied (continuous cov.)
Ratio of family income to the poverty level	Not applied (continuous covariate)	0.17 (0.58)	[–1.01, 1.35]	0.30 (0.769)	Not applied (continuous cov.)
Depression raw scores	Not applied (continuous covariate)	–0.13 (0.20)	[–0.53, 0.28]	–0.63 (0.535)	Not applied (continuous cov.)

[a]The column labeled "estimated marginal means" shows each category's marginal means against the reference category's marginal mean. The reference categories and the marginal means of the reference categories are shown in italics. The significant results have been colored.

Table S77. Multiple linear regression model explaining the relationship between DSST and specific combination TRI + HDL + HBP after adjusting by sociodemographic characteristics and medical history, and estimated marginal means for each category of predictors and covariates[a]

**Model coefficient**	**Categories (for categorical predictor and covariates)**	*Beta* (*SE*)	95% CI	*t* (*p*)	Estimated marginal means[b]
Constant	Not applied (model intercept)	150.93 (15.26)	[117.69, 184.17]	9.89 (< 0.001)	Not applied (model intercept)
Education level	Up to 12th grade vs. *college graduate or above*	–15.30 (4.20)	[–24.44, –6.15]	–3.64 (0.003)	32.58 (5.61) vs. *47.88 (5.40)*
	High school graduate vs. *college graduate or above*	–8.27 (4.22)	[–17.46, 0.91]	–1.96 (0.073)	39.60 (4.80) vs. *47.88 (5.40)*
	Some college or AA degree vs. *college graduate or above*	–1.59 (2.68)	[–7.42, 4.24]	–0.59 (0.564)	46.29 (5.57) vs. *47.88 (5.40)*
Gender	Males vs. *females*	–7.48 (2.45)	[–12.82, –2.15]	–3.06 (0.010)	37.85 (4.86) vs. *45.33 (5.04)*
Race	Mexican American vs. *Non–Hispanic White*	–11.88 (3.63)	[–19.80, –3.96]	–3.27 (0.007)	34.40 (5.93) vs. *46.28 (4.78)*
	Other Hispanic vs. *Non–Hispanic White*	–7.39 (4.02)	[–16.16, 1.38]	–1.84 (0.091)	38.90 (5.83) vs. *46.28 (4.78)*
	Non–Hispanic Black vs. *Non–Hispanic White*	–18.79 (4.38)	[–28.34, –9.24]	–4.29 (0.001)	27.49 (6.67) vs. *46.28 (4.78)*
	Non–Hispanic Asian vs. *Non–Hispanic White*	14.59 (5.26)	[3.12, 26.06]	2.77 (0.017)	60.87 (6.03) vs. *46.28 (4.78)*
	Other Race and Multi–Racial vs. *Non–Hispanic White*	No cases	No cases	No cases	No cases
Marital status	Single or never married vs. *married or living with a partner*	–18.66 (7.80)	[–35.65, –1.66]	–2.39 (0.034)	25.14 (9.31) vs. *43.80 (4.06)*
	Divorced or separated vs. *married or living with a partner*	1.00 (3.94)	[–7.58, 9.57]	0.25 (0.804)	44.79 (5.56) vs. *43.80 (4.06)*
	Widowed vs. *married or living with a partner*	8.82 (3.31)	[1.61, 16.03]	2.67 (0.021)	52.62 (5.10) vs. *43.80 (4.06)*
Self–report general health condition	Poor vs. *excellent or very good*	No cases	No cases	No cases	No cases
	Good or fair vs. *excellent or very good*	–8.40 (3.29)	[–15.56, –1.24]	–2.56 (0.025)	37.39 (5.46) vs. *45.79 (4.65)*
Difficulties in thinking or remembering	Yes vs. *no*	–5.27 (2.59)	[–10.91, 0.37]	–2.04 (0.064)	38.95 (5.13) vs. *44.22 (4.80)*
Ever told you have a heart disease	Yes vs. *no*	0.39 (6.24)	[–13.19, 13.98]	0.06 (0.951)	41.79 (7.38) vs. *41.39 (3.32)*
Ever told you had a stroke	Yes vs. *no*	7.94 (5.07)	[–3.10, 18.99]	1.57 (0.143)	45.56 (6.57) vs. *37.62 (3.97)*
Ever told you have diabetes	Yes vs. *no*	–7.16 (5.97)	[–20.17, 5.84]	–1.20 (0.253)	38.01 (6.35) vs. *45.17 (4.85)*
Have smoked at least 100 cigarettes in life	Yes vs. *no*	1.25 (2.51)	[–4.22, 6.73]	0.50 (0.627)	42.22 (4.84) vs. *40.96 (5.08)*
Three criteria combination: TRI + HDL + HBP	TRI + HDL + HBP vs. *metabolically–healthy people*	No cases	No cases	No cases	No cases
Age at screening	Not applied (continuous covariate)	–0.43 (0.76)	[–2.07, 1.22]	–0.56 (0.583)	Not applied (continuous cov.)
Ratio of family income to the poverty level	Not applied (continuous covariate)	1.26 (0.76)	[–0.39, 2.91]	1.67 (0.121)	Not applied (continuous cov.)
Depression raw scores	Not applied (continuous covariate)	–0.13 (0.20)	[–0.53, 0.28]	–0.63 (0.535)	Not applied (continuous cov.)

[a]The design–based covariance matrix is singular. The validity of results is uncertain. [b]The column labeled "estimated marginal means" shows each category's marginal means against the reference category's marginal mean. The reference categories and the marginal means of the reference categories are shown in italics. The significant results have been colored.

Table S78. Multiple linear regression model explaining the relationship between DSST and specific combination TRI + HDL + GLY after adjusting by sociodemographic characteristics and medical history, and estimated marginal means for each category of predictors and covariates[a]

**Model coefficient**	**Categories (for categorical predictor and covariates)**	*Beta* (*SE*)	95% CI	*t* (*p*)	Estimated marginal means[b]
Constant	Not applied (model intercept)	142.22 (16.18)	[107.27, 177.17]	8.79 (< 0.001)	Not applied (model intercept)
Education level	Up to 12th grade vs. *college graduate or above*	–8.39 (3.65)	[–16.28, –0.50]	–2.30 (0.039)	34.72 (9.23) vs. *43.11 (10.76)*
	High school graduate vs. *college graduate or above*	–5.66 (4.76)	[–15.95, 4.62]	–1.19 (0.256)	37.45 (9.08) vs. *43.11 (10.76)*
	Some college or AA degree vs. *college graduate or above*	3.73 (2.02)	[–0.63, 8.09]	1.85 (0.088)	46.84 (10.28) vs. *43.11 (10.76)*
Gender	Males vs. *females*	–9.28 (2.67)	[–15.05, –3.52]	–3.48 (0.004)	35.89 (9.50) vs. *45.17 (9.74)*
Race	Mexican American vs. *Non–Hispanic White*	–16.45 (3.47)	[–23.94, –8.97]	–4.75 (< 0.001)	32.42 (9.55) vs. *48.87 (8.32)*
	Other Hispanic vs. *Non–Hispanic White*	–18.06 (5.62)	[–30.19, –5.92]	–3.21 (0.007)	30.81 (12.46) vs. *48.87 (8.32)*
	Non–Hispanic Black vs. *Non–Hispanic White*	–18.37 (4.56)	[–28.21, –8.52]	–4.03 (0.001)	30.50 (10.27) vs. *48.87 (8.32)*
	Non–Hispanic Asian vs. *Non–Hispanic White*	11.18 (5.67)	[–1.07, 23.43]	1.97 (0.070)	60.05 (10.21) vs. *48.87 (8.32)*
	Other Race and Multi–Racial vs. *Non–Hispanic White*	No cases	No cases	No cases	No cases
Marital status	Single or never married vs. *married or living with a partner*	–10.06 (7.75)	[–26.79, 6.68]	–1.30 (0.217)	29.25 (10.76) vs. *39.30 (8.91)*
	Divorced or separated vs. *married or living with a partner*	11.40 (2.88)	[5.19, 17.62]	3.96 (0.002)	50.71 (10.66) vs. *39.30 (8.91)*
	Widowed vs. *married or living with a partner*	3.57 (2.57)	[–1.99, 9.12]	1.39 (0.189)	42.87 (10.47) vs. *39.30 (8.91)*
Self–report general health condition	Poor vs. *excellent or very good*	No cases	No cases	No cases	No cases
	Good or fair vs. *excellent or very good*	–6.56 (4.00)	[–15.20, 2.08]	–1.64 (0.125)	37.25 (9.54) vs. *43.81 (9.93)*
Difficulties in thinking or remembering	Yes vs. *no*	–5.87 (3.60)	[–13.66, 1.91]	–1.63 (0.127)	37.59 (10.22) vs. *43.47 (9.14)*
Ever told you have a heart disease	Yes vs. *no*	0.93 (6.60)	[–13.33, 15.20]	0.14 (0.890)	41.00 (12.04) vs. *40.06 (7.64)*
Ever told you had a stroke	Yes vs. *no*	11.38 (5.34)	[–0.16, 22.91]	2.13 (0.053)	46.22 (11.61) vs. *34.84 (7.81)*
Ever told you have diabetes	Yes vs. *no*	–20.49 (13.68)	[–50.05, 9.06]	–1.50 (0.158)	30.28 (14.87) vs. *50.78 (7.34)*
Have smoked at least 100 cigarettes in life	Yes vs. *no*	–0.12 (3.27)	[–7.18, 6.94]	–0.04 (0.971)	40.47 (9.73) vs. *40.59 (9.60)*
Three criteria combination: TRI + HDL + GLY	TRI + HDL + GLY vs. *metabolically–healthy people*	6.77 (5.03)	[–4.09, 17.63]	1.35 (0.201)	43.92 (10.35) vs. *37.15 (9.33)*
Age at screening	Not applied (continuous covariate)	–1.14 (0.21)	[–1.60, –0.69]	–5.42 (< 0.001)	Not applied (continuous cov.)
Ratio of family income to the poverty level	Not applied (continuous covariate)	–1.39 (0.88)	[–3.29, 0.52]	–1.57 (0.139)	Not applied (continuous cov.)
Depression raw scores	Not applied (continuous covariate)	–0.01 (0.66)	[–1.44, 1.42]	–0.01 (0.991)	Not applied (continuous cov.)

[a]The design–based covariance matrix is singular. The validity of results is uncertain. [b]The column labeled "estimated marginal means" shows each category's marginal means against the reference category's marginal mean. The reference categories and the marginal means of the reference categories are shown in italics. The significant results have been colored.

Table S79. Multiple linear regression model explaining the relationship between DSST and specific combination TRI + HBP + GLY after adjusting by sociodemographic characteristics and medical history, and estimated marginal means for each category of predictors and covariates[a]

**Model coefficient**	**Categories (for categorical predictor and covariates)**	*Beta* (*SE*)	95% CI	*t* (*p*)	Estimated marginal means[b]
Constant	Not applied (model intercept)	152.36 (16.25)	[117.51, 187.21]	9.38 (< 0.001)	Not applied (model intercept)
Education level	Up to 12th grade vs. *college graduate or above*	–14.73 (4.13)	[–23.59, –5.88]	–3.57 (0.003)	33.71 (6.44) vs. *48.44 (7.77)*
	High school graduate vs. *college graduate or above*	–8.19 (4.18)	[–17.16, 0.77]	–1.96 (0.070)	40.25 (5.73) vs. *48.44 (7.77)*
	Some college or AA degree vs. *college graduate or above*	–1.22 (2.80)	[–7.22, 4.79]	–0.43 (0.670)	47.23 (7.62) vs. *48.44 (7.77)*
Gender	Males vs. *females*	–7.59 (2.39)	[–12.73, –2.46]	–3.17 (0.007)	38.61 (6.70) vs. *46.20 (6.57)*
Race	Mexican American vs. *Non–Hispanic White*	–12.83 (3.68)	[–20.72, –4.94]	–3.49 (0.004)	35.00 (7.19) vs. *47.83 (5.79)*
	Other Hispanic vs. *Non–Hispanic White*	–7.93 (4.39)	[–17.34, 1.48]	–1.81 (0.092)	39.90 (7.34) vs. *47.83 (5.79)*
	Non–Hispanic Black vs. *Non–Hispanic White*	–19.37 (3.91)	[–27.76, –10.98]	–4.95 (< 0.001)	28.46 (7.32) vs. *47.83 (5.79)*
	Non–Hispanic Asian vs. *Non–Hispanic White*	13.02 (4.94)	[2.42, 23.62]	2.63 (0.020)	60.85 (8.50) vs. *47.83 (5.79)*
	Other Race and Multi–Racial vs. *Non–Hispanic White*	No cases	No cases	No cases	No cases
Marital status	Single or never married vs. *married or living with a partner*	–17.32 (7.99)	[–34.45, –0.19]	–2.17 (0.048)	26.70 (10.58) vs. *44.02 (5.86)*
	Divorced or separated vs. *married or living with a partner*	1.33 (3.73)	[–6.66, 9.32]	0.36 (0.727)	45.35 (6.36) vs. *44.02 (5.86)*
	Widowed vs. *married or living with a partner*	9.55 (3.67)	[1.66, 17.43]	2.60 (0.021)	53.56 (7.27) vs. *44.02 (5.86)*
Self–report general health condition	Poor vs. *excellent or very good*	No cases	No cases	No cases	No cases
	Good or fair vs. *excellent or very good*	–8.46 (3.45)	[–15.85, –1.06]	–2.45 (0.028)	38.18 (7.02) vs. *46.63 (6.46)*
Difficulties in thinking or remembering	Yes vs. *no*	–5.11 (2.86)	[–11.24, 1.02]	–1.79 (0.096)	39.85 (6.96) vs. *44.96 (6.39)*
Ever told you have a heart disease	Yes vs. *no*	0.43 (6.23)	[–12.92, 13.79]	0.07 (0.946)	42.62 (9.17) vs. *42.19 (4.52)*
Ever told you had a stroke	Yes vs. *no*	8.93 (4.80)	[–1.37, 19.22]	1.86 (0.084)	46.87 (8.45) vs. *37.94 (5.02)*
Ever told you have diabetes	Yes vs. *no*	–8.17 (5.49)	[–19.94, 3.59]	–1.49 (0.158)	38.32 (6.90) vs. *46.49 (7.25)*
Have smoked at least 100 cigarettes in life	Yes vs. *no*	1.17 (2.61)	[–4.43, 6.78]	0.45 (0.660)	42.99 (6.31) vs. *41.82 (6.98)*
Three criteria combination: TRI + HBP + GLY	TRI + HBP + GLY vs. *metabolically–healthy people*	2.02 (7.41)	[–13.87, 17.91]	0.27 (0.789)	43.42 (9.25) vs. *41.39 (5.20)*
Age at screening	Not applied (continuous covariate)	–1.32 (0.22)	[–1.79, –0.86]	–6.10 (< 0.001)	Not applied (continuous cov.)
Ratio of family income to the poverty level	Not applied (continuous covariate)	–0.63 (0.73)	[–2.19, 0.93]	–0.86 (0.402)	Not applied (continuous cov.)
Depression raw scores	Not applied (continuous covariate)	1.12 (0.68)	[–0.34, 2.58]	1.65 (0.122)	Not applied (continuous cov.)

[a]The design–based covariance matrix is singular. The validity of results is uncertain. [b]The column labeled "estimated marginal means" shows each category's marginal means against the reference category's marginal mean. The reference categories and the marginal means of the reference categories are shown in italics. The significant results have been colored.

Table S80. Multiple linear regression model explaining the relationship between DSST and specific combination HDL + HBP + GLY after adjusting by sociodemographic characteristics and medical history, and estimated marginal means for each category of predictors and covariates[a]

**Model coefficient**	**Categories (for categorical predictor and covariates)**	*Beta* (*SE*)	95% CI	*t* (*p*)	Estimated marginal means[b]
Constant	Not applied (model intercept)	152.07 (14.48)	[121.37, 182.77]	10.50 (< 0.001)	Not applied (model intercept)
Education level	Up to 12th grade vs. *college graduate or above*	–14.83 (4.21)	[–23.75, –5.91]	–3.52 (0.003)	24.22 (5.44) vs. *39.05 (6.25)*
	High school graduate vs. *college graduate or above*	–8.52 (4.49)	[–18.05, 1.00]	–1.90 (0.076)	30.53 (4.98) vs. *39.05 (6.25)*
	Some college or AA degree vs. *college graduate or above*	–2.79 (2.64)	[–8.40, 2.81]	–1.06 (0.307)	36.26 (6.00) vs. *39.05 (6.25)*
Gender	Males vs. *females*	–6.86 (2.32)	[–11.79, –1.93]	–2.95 (0.009)	29.09 (5.29) vs. *35.95 (5.23)*
Race	Mexican American vs. *Non–Hispanic White*	–12.13 (3.38)	[–19.30, –4.97]	–3.59 (0.002)	25.75 (5.76) vs. *37.89 (4.86)*
	Other Hispanic vs. *Non–Hispanic White*	–11.85 (6.66)	[–25.95, 2.26]	–1.78 (0.094)	26.04 (7.81) vs. *37.89 (4.86)*
	Non–Hispanic Black vs. *Non–Hispanic White*	–16.30 (3.85)	[–24.46, –8.13]	–4.23 (0.001)	21.59 (6.34) vs. *37.89 (4.86)*
	Non–Hispanic Asian vs. *Non–Hispanic White*	13.43 (4.39)	[4.12, 22.75]	3.06 (0.008)	51.32 (6.55) vs. *37.89 (4.86)*
	Other Race and Multi–Racial vs. *Non–Hispanic White*	No cases	No cases	No cases	No cases
Marital status	Single or never married vs. *married or living with a partner*	–19.20 (7.70)	[–35.53, –2.87]	–2.49 (0.024)	16.11 (9.84) vs. *35.31 (4.66)*
	Divorced or separated vs. *married or living with a partner*	–1.67 (4.06)	[–10.27, 6.93]	–0.41 (0.686)	33.64 (5.18) vs. *35.31 (4.66)*
	Widowed vs. *married or living with a partner*	9.70 (3.60)	[2.07, 17.33]	2.69 (0.016)	45.01 (5.37) vs. *35.31 (4.66)*
Self–report general health condition	Poor vs. *excellent or very good*	–28.00 (5.54)	[–39.75, –16.26]	–5.05 (< 0.001)	16.78 (4.71) vs. *44.78 (6.03)*
	Good or fair vs. *excellent or very good*	–8.79 (3.10)	[–15.37, –2.21]	–2.83 (0.012)	35.99 (6.78) vs. *44.78 (6.03)*
Difficulties in thinking or remembering	Yes vs. *no*	–5.82 (2.61)	[–11.34, –0.29]	–2.23 (0.040)	29.61 (5.81) vs. *35.43 (4.72)*
Ever told you have a heart disease	Yes vs. *no*	0.12 (5.94)	[–12.46, 12.71]	0.02 (0.984)	32.58 (7.72) vs. *32.46 (3.28)*
Ever told you had a stroke	Yes vs. *no*	5.14 (4.90)	[–5.24, 15.52]	1.05 (0.309)	35.09 (7.00) vs. *29.95 (3.96)*
Ever told you have diabetes	Yes vs. *no*	0.73 (5.93)	[–11.84, 13.30]	0.12 (0.903)	32.88 (6.14) vs. *32.15 (5.71)*
Have smoked at least 100 cigarettes in life	Yes vs. *no*	1.55 (2.34)	[–3.41, 6.51]	0.66 (0.518)	33.29 (5.02) vs. *31.75 (5.50)*
Three criteria combination: HDL + HBP + GLY	HDL + HBP + GLY vs. *metabolically–healthy people*	–3.83 (3.47)	[–11.18, 3.51]	–1.11 (0.285)	30.60 (6.47) vs. *34.44 (4.10)*
Age at screening	Not applied (continuous covariate)	–1.33 (0.18)	[–1.72, –0.94]	–7.30 (< 0.001)	Not applied (continuous cov.)
Ratio of family income to the poverty level	Not applied (continuous covariate)	–0.19 (0.79)	[–1.86, 1.49]	–0.24 (0.813)	Not applied (continuous cov.)
Depression raw scores	Not applied (continuous covariate)	1.27 (0.72)	[–0.26, 2.80]	1.76 (0.098)	Not applied (continuous cov.)

[a]The design–based covariance matrix is singular. The validity of results is uncertain. [b]The column labeled "estimated marginal means" shows each category's marginal means against the reference category's marginal mean. The reference categories and the marginal means of the reference categories are shown in italics. The significant results have been colored.

Table S81. Multiple linear regression model explaining the relationship between DSST and all combinations of three criteria after adjusting by sociodemographic characteristics and medical history, and estimated marginal means for each category of predictors and covariates

**Model coefficient**	**Categories (for categorical predictor and covariates)**	*Beta* (*SE*)	95% CI	*t* (*p*)	Estimated marginal means[a]
Constant	Not applied (model intercept)	124.00 (9.36)	[104.93, 143.06]	13.25 (< 0.001)	Not applied (model intercept)
Education level	Up to 12th grade vs. *college graduate or above*	–13.91 (2.60)	[–19.21, –8.61]	–5.34 (< 0.001)	31.89 (2.87) vs. *45.80 (3.61)*
	High school graduate vs. *college graduate or above*	–10.20 (2.86)	[–16.02, –4.39]	–3.57 (0.001)	35.60 (3.05) vs. *45.80 (3.61)*
	Some college or AA degree vs. *college graduate or above*	–6.03 (2.40)	[–10.92, –1.14]	–2.51 (0.017)	39.77 (2.90) vs. *45.80 (3.61)*
Gender	Males vs. *females*	–6.27 (1.77)	[–9.87, –2.67]	–3.55 (0.001)	35.13 (3.05) vs. *41.40 (2.72)*
Race	Mexican American vs. *Non–Hispanic White*	–12.09 (2.56)	[–17.29, –6.88]	–4.73 (< 0.001)	32.38 (3.71) vs. *44.47 (2.15)*
	Other Hispanic vs. *Non–Hispanic White*	–11.07 (2.93)	[–17.04, –5.09]	–3.78 (0.001)	33.40 (4.00) vs. *44.47 (2.15)*
	Non–Hispanic Black vs. *Non–Hispanic White*	–11.50 (1.93)	[–15.44, –7.57]	–5.95 (< 0.001)	32.97 (3.05) vs. *44.47 (2.15)*
	Non–Hispanic Asian vs. *Non–Hispanic White*	4.05 (3.00)	[–2.07, 10.17]	1.35 (0.187)	48.52 (3.62) vs. *44.47 (2.15)*
	Other Race and Multi–Racial vs. *Non–Hispanic White*	–6.60 (2.20)	[–11.07, –2.13]	–3.01 (0.005)	37.87 (3.55) vs. *44.47 (2.15)*
Marital status	Single or never married vs. *married or living with a partner*	–1.64 (3.96)	[–9.70, 6.43]	–0.41 (0.682)	37.83 (4.79) vs. *39.47 (3.02)*
	Divorced or separated vs. *married or living with a partner*	1.00 (2.89)	[–4.89, 6.88]	0.35 (0.732)	40.47 (3.56) vs. *39.47 (3.02)*
	Widowed vs. *married or living with a partner*	–4.17 (3.13)	[–10.54, 2.20]	–1.33 (0.192)	35.30 (3.52) vs. *39.47 (3.02)*
Self–report general health condition	Poor vs. *excellent or very good*	4.37 (5.05)	[–5.92, 14.66]	0.87 (0.393)	41.21 (4.90) vs. *36.84 (3.22)*
	Good or fair vs. *excellent or very good*	–0.08 (2.28)	[–4.73, 4.57]	–0.04 (0.972)	36.75 (2.46) vs. *36.84 (3.22)*
Difficulties in thinking or remembering	Yes vs. *no*	–8.57 (2.07)	[–12.78, –4.36]	–4.14 (< 0.001)	33.98 (3.51) vs. *42.55 (2.22)*
Ever told you have a heart disease	Yes vs. *no*	–1.40 (3.38)	[–8.28, 5.48]	–0.41 (0.682)	37.57 (3.72) vs. *38.96 (2.64)*
Ever told you had a stroke	Yes vs. *no*	–2.94 (1.75)	[–6.51, 0.63]	–1.68 (0.103)	36.80 (3.15) vs. *39.74 (2.60)*
Ever told you have diabetes	Yes vs. *no*	–5.94 (1.43)	[–8.84, –3.03]	–4.16 (< 0.001)	35.30 (2.98) vs. *41.24 (2.70)*
Have smoked at least 100 cigarettes in life	Yes vs. *no*	–0.17 (1.91)	[–4.06, 3.72]	–0.09 (0.931)	38.18 (2.60) vs. *38.35 (3.19)*
All combinations of three criteria	All combinations of three criteria vs. *metabolically–healthy people*	3.31 (2.61)	[–2.01, 8.63]	1.27 (0.214)	39.92 (2.77) vs. *36.61 (3.30)*
Age at screening	Not applied (continuous covariate)	–0.87 (0.14)	[–1.16, –0.57]	–6.02 (< 0.001)	Not applied (continuous cov.)
Ratio of family income to the poverty level	Not applied (continuous covariate)	–0.31 (0.67)	[–1.68, 1.06]	–0.46 (0.647)	Not applied (continuous cov.)
Depression raw scores	Not applied (continuous covariate)	–0.27 (0.19)	[–0.65, 0.11]	–1.43 (0.163)	Not applied (continuous cov.)

[a]The column labeled "estimated marginal means" shows each category's marginal means against the reference category's marginal mean. The reference categories and the marginal means of the reference categories are shown in italics. The significant results have been colored.

Table S82. Multiple linear regression model explaining the relationship between DSST and specific combination AO + TRI + HDL + HBP after adjusting by sociodemographic characteristics and medical history, and estimated marginal means for each category of predictors and covariates[a]

**Model coefficient**	**Categories (for categorical predictor and covariates)**	*Beta* (*SE*)	95% CI	*t* (*p*)	Estimated marginal means[b]
Constant	Not applied (model intercept)	147.50 (14.42)	[116.34, 178.66]	10.23 (< 0.001)	Not applied (model intercept)
Education level	Up to 12th grade vs. *college graduate or above*	–13.41 (4.34)	[–22.78, –4.03]	–3.09 (0.009)	29.34 (4.75) vs. *42.74 (6.43)*
	High school graduate vs. *college graduate or above*	–7.65 (4.42)	[–17.20, 1.89]	–1.73 (0.107)	35.09 (5.60) vs. *42.74 (6.43)*
	Some college or AA degree vs. *college graduate or above*	–0.88 (2.73)	[–6.78, 5.02]	–0.32 (0.753)	41.87 (6.05) vs. *42.74 (6.43)*
Gender	Males vs. *females*	–7.12 (2.44)	[–12.39, –1.85]	–2.92 (0.012)	33.70 (5.83) vs. *40.82 (4.82)*
Race	Mexican American vs. *Non–Hispanic White*	–14.04 (3.58)	[–21.77, –6.31]	–3.92 (0.002)	29.12 (5.83) vs. *43.16 (4.91)*
	Other Hispanic vs. *Non–Hispanic White*	–8.35 (4.37)	[–17.80, 1.09]	–1.91 (0.078)	34.81 (5.58) vs. *43.16 (4.91)*
	Non–Hispanic Black vs. *Non–Hispanic White*	–19.09 (3.64)	[–26.95, –11.24]	–5.25 (< 0.001)	24.06 (5.97) vs. *43.16 (4.91)*
	Non–Hispanic Asian vs. *Non–Hispanic White*	12.00 (4.53)	[2.20, 21.79]	2.65 (0.020)	55.16 (7.54) vs. *43.16 (4.91)*
	Other Race and Multi–Racial vs. *Non–Hispanic White*	No cases	No cases	No cases	No cases
Marital status	Single or never married vs. *married or living with a partner*	–16.04 (7.71)	[–32.70, 0.63]	–2.08 (0.058)	22.57 (9.37) vs. *38.61 (4.86)*
	Divorced or separated vs. *married or living with a partner*	2.00 (3.82)	[–6.25, 10.24]	0.52 (0.610)	40.60 (4.78) vs. *38.61 (4.86)*
	Widowed vs. *married or living with a partner*	8.65 (2.19)	[3.92, 13.38]	3.95 (0.002)	47.26 (5.79) vs. *38.61 (4.86)*
Self–report general health condition	Poor vs. *excellent or very good*	–34.43 (8.17)	[–52.08, –16.77]	–4.21 (0.001)	17.01 (9.01) vs. *51.43 (4.65)*
	Good or fair vs. *excellent or very good*	–8.09 (2.96)	[–14.48, –1.71]	–2.74 (0.017)	43.34 (4.58) vs. *51.43 (4.65)*
Difficulties in thinking or remembering	Yes vs. *no*	–8.16 (3.14)	[–14.94, –1.37]	–2.60 (0.022)	33.18 (5.94) vs. *41.34 (4.88)*
Ever told you have a heart disease	Yes vs. *no*	1.57 (5.49)	[–10.29, 13.44]	0.29 (0.779)	38.05 (7.47) vs. *36.48 (3.67)*
Ever told you had a stroke	Yes vs. *no*	17.78 (7.93)	[0.66, 34.91]	2.24 (0.043)	46.15 (7.15) vs. *28.37 (5.87)*
Ever told you have diabetes	Yes vs. *no*	0.33 (6.59)	[–13.91, 14.58]	0.05 (0.960)	37.43 (5.88) vs. *37.09 (6.44)*
Have smoked at least 100 cigarettes in life	Yes vs. *no*	–1.06 (2.58)	[–6.65, 4.52]	–0.41 (0.687)	36.73 (5.05) vs. *37.79 (5.67)*
Four criteria combination: AO + TRI + HDL + HBP	AO + TRI + HDL + HBP vs. *metabolically–healthy people*	–4.10 (3.42)	[–11.49, 3.29]	–1.20 (0.252)	35.21 (6.30) vs. *39.31 (4.52)*
Age at screening	Not applied (continuous covariate)	–1.23 (0.19)	[–1.64, –0.83]	–6.57 (< 0.001)	Not applied (continuous cov.)
Ratio of family income to the poverty level	Not applied (continuous covariate)	–0.61 (0.84)	[–2.42, 1.21]	–0.72 (0.483)	Not applied (continuous cov.)
Depression raw scores	Not applied (continuous covariate)	0.78 (0.52)	[–0.33, 1.90]	1.52 (0.153)	Not applied (continuous cov.)

[a]The design–based covariance matrix is singular. The validity of results is uncertain. [b]The column labeled "estimated marginal means" shows each category's marginal means against the reference category's marginal mean. The reference categories and the marginal means of the reference categories are shown in italics. The significant results have been colored.

Table S83. Multiple linear regression model explaining the relationship between DSST and specific combination AO + TRI + HDL + GLY after adjusting by sociodemographic characteristics and medical history, and estimated marginal means for each category of predictors and covariates[a]

**Model coefficient**	**Categories (for categorical predictor and covariates)**	*Beta* (*SE*)	95% CI	*t* (*p*)	Estimated marginal means[b]
Constant	Not applied (model intercept)	125.97 (9.31)	[106.49, 145.46]	13.53 (< 0.001)	Not applied (model intercept)
Education level	Up to 12th grade vs. *college graduate or above*	–12.14 (3.36)	[–19.18, –5.11]	–3.61 (0.002)	36.48 (2.66) vs. *48.63 (4.01)*
	High school graduate vs. *college graduate or above*	–3.27 (3.16)	[–9.89, 3.35]	–1.03 (0.315)	45.36 (3.24) vs. *48.63 (4.01)*
	Some college or AA degree vs. *college graduate or above*	–2.15 (2.61)	[–7.62, 3.32]	–0.82 (0.421)	46.48 (3.38) vs. *48.63 (4.01)*
Gender	Males vs. *females*	–8.77 (2.21)	[–13.39, –4.14]	–3.97 (0.001)	39.86 (2.95) vs. *48.62 (2.93)*
Race	Mexican American vs. *Non–Hispanic White*	–10.44 (3.71)	[–18.20, –2.67]	–2.81 (0.011)	39.28 (4.56) vs. *49.71 (2.23)*
	Other Hispanic vs. *Non–Hispanic White*	–14.92 (2.58)	[–20.31, –9.52]	–5.79 (< 0.001)	34.80 (2.97) vs. *49.71 (2.23)*
	Non–Hispanic Black vs. *Non–Hispanic White*	–14.04 (4.42)	[–23.30, –4.78]	–3.17 (0.005)	35.68 (4.74) vs. *49.71 (2.23)*
	Non–Hispanic Asian vs. *Non–Hispanic White*	5.28 (4.19)	[–3.49, 14.04]	1.26 (0.223)	54.99 (4.84) vs. *49.71 (2.23)*
	Other Race and Multi–Racial vs. *Non–Hispanic White*	1.26 (3.92)	[–6.95, 9.47]	0.32 (0.752)	50.97 (3.90) vs. *49.71 (2.23)*
Marital status	Single or never married vs. *married or living with a partner*	–15.28 (4.13)	[–23.92, –6.64]	–3.70 (0.002)	33.10 (4.94) vs. *48.38 (2.35)*
	Divorced or separated vs. *married or living with a partner*	0.44 (2.76)	[–5.34, 6.22]	0.16 (0.875)	48.82 (2.60) vs. *48.38 (2.35)*
	Widowed vs. *married or living with a partner*	–1.73 (3.04)	[–8.10, 4.63]	–0.57 (0.576)	46.65 (4.34) vs. *48.38 (2.35)*
Self–report general health condition	Poor vs. *excellent or very good*	–8.21 (4.22)	[–17.05, 0.62]	–1.95 (0.067)	40.71 (3.10) vs. *48.93 (3.58)*
	Good or fair vs. *excellent or very good*	–5.85 (3.01)	[–12.16, 0.45]	–1.94 (0.067)	43.07 (3.67) vs. *48.93 (3.58)*
Difficulties in thinking or remembering	Yes vs. *no*	–2.67 (2.22)	[–7.31, 1.96]	–1.21 (0.242)	42.90 (3.26) vs. *45.58 (2.58)*
Ever told you have a heart disease	Yes vs. *no*	3.38 (4.00)	[–5.00, 11.76]	0.85 (0.409)	45.93 (4.28) vs. *42.55 (2.13)*
Ever told you had a stroke	Yes vs. *no*	1.90 (4.16)	[–6.80, 10.59]	0.46 (0.653)	45.19 (4.20) vs. *43.29 (2.42)*
Ever told you have diabetes	Yes vs. *no*	–0.68 (2.18)	[–5.23, 3.88]	–0.31 (0.759)	43.90 (2.72) vs. *44.58 (3.14)*
Have smoked at least 100 cigarettes in life	Yes vs. *no*	–3.49 (2.43)	[–8.57, 1.59]	–1.44 (0.166)	42.49 (2.26) vs. *45.98 (3.56)*
Four criteria combination: AO + TRI + HDL + GLY	AO + TRI + HDL + GLY vs. *metabolically–healthy people*	6.38 (1.68)	[2.87, 9.89]	3.81 (0.001)	47.43 (2.86) vs. *41.05 (2.84)*
Age at screening	Not applied (continuous covariate)	–0.92 (0.13)	[–1.20, –0.64]	–6.84 (< 0.001)	Not applied (continuous cov.)
Ratio of family income to the poverty level	Not applied (continuous covariate)	0.70 (0.59)	[–0.54, 1.94]	1.18 (0.252)	Not applied (continuous cov.)
Depression raw scores	Not applied (continuous covariate)	–0.16 (0.31)	[–0.80, 0.48]	–0.53 (0.603)	Not applied (continuous cov.)

[a]The design–based covariance matrix is singular. The validity of results is uncertain. [b]The column labeled "estimated marginal means" shows each category's marginal means against the reference category's marginal mean. The reference categories and the marginal means of the reference categories are shown in italics. The significant results have been colored.

Table S84. Multiple linear regression model explaining the relationship between DSST and specific combination AO + TRI + HBP + GLY after adjusting by sociodemographic characteristics and medical history, and estimated marginal means for each category of predictors and covariates[a]

**Model coefficient**	**Categories (for categorical predictor and covariates)**	*Beta* (*SE*)	95% CI	*t* (*p*)	Estimated marginal means[b]
Constant	Not applied (model intercept)	156.19 (11.94)	[131.54, 180.84]	13.08 (< 0.001)	Not applied (model intercept)
Education level	Up to 12th grade vs. *college graduate or above*	−12.03 (3.38)	[−19.01, −5.05]	−3.56 (0.002)	34.21 (4.16) vs. *46.24 (4.53)*
	High school graduate vs. *college graduate or above*	−1.30 (3.39)	[−8.29, 5.70]	−0.38 (0.705)	44.94 (2.80) vs. *46.24 (4.53)*
	Some college or AA degree vs. *college graduate or above*	−3.55 (2.82)	[−9.37, 2.27]	−1.26 (0.220)	42.69 (4.05) vs. *46.24 (4.53)*
Gender	Males vs. *females*	−11.03 (2.73)	[−16.67, −5.39]	−4.03 (< 0.001)	36.50 (3.58) vs. *47.53 (3.72)*
Race	Mexican American vs. *Non–Hispanic White*	−9.21 (4.73)	[−18.98, 0.56]	−1.95 (0.063)	37.75 (4.08) vs. *46.96 (3.01)*
	Other Hispanic vs. *Non–Hispanic White*	−8.40 (2.57)	[−13.70, −3.10]	−3.27 (0.003)	38.56 (3.95) vs. *46.96 (3.01)*
	Non–Hispanic Black vs. *Non–Hispanic White*	−11.46 (3.84)	[−19.39, −3.53]	−2.98 (0.006)	35.50 (5.92) vs. *46.96 (3.01)*
	Non–Hispanic Asian vs. *Non–Hispanic White*	4.35 (4.59)	[−5.13, 13.83]	0.95 (0.353)	51.31 (5.53) vs. *46.96 (3.01)*
	Other Race and Multi–Racial vs. *Non–Hispanic White*	No cases	No cases	No cases	No cases
Marital status	Single or never married vs. *married or living with a partner*	−20.14 (3.67)	[−27.72, −12.55]	−5.48 (< 0.001)	25.81 (5.12) vs. *45.95 (3.30)*
	Divorced or separated vs. *married or living with a partner*	3.36 (3.01)	[−2.85, 9.57]	1.12 (0.275)	49.31 (4.16) vs. *45.95 (3.30)*
	Widowed vs. *married or living with a partner*	1.05 (2.72)	[−4.56, 6.66]	0.39 (0.703)	47.00 (3.60) vs. *45.95 (3.30)*
Self–report general health condition	Poor vs. *excellent or very good*	2.88 (6.78)	[−11.11, 16.87]	0.43 (0.675)	44.39 (5.53) vs. *41.51 (4.49)*
	Good or fair vs. *excellent or very good*	−1.36 (2.42)	[−6.36, 3.64]	−0.56 (0.580)	40.15 (3.56) vs. *41.51 (4.49)*
Difficulties in thinking or remembering	Yes vs. *no*	−6.43 (3.83)	[−14.33, 1.47]	−1.68 (0.106)	38.80 (4.25) vs. *45.23 (3.49)*
Ever told you have a heart disease	Yes vs. *no*	1.37 (2.94)	[−4.71, 7.45]	0.47 (0.646)	42.70 (4.46) vs. *41.33 (2.71)*
Ever told you had a stroke	Yes vs. *no*	−8.24 (2.85)	[−14.12, −2.37]	−2.90 (0.008)	37.90 (4.54) vs. *46.14 (2.52)*
Ever told you have diabetes	Yes vs. *no*	7.71 (2.56)	[2.42, 13.00]	3.01 (0.006)	45.87 (4.16) vs. *38.16 (2.99)*
Have smoked at least 100 cigarettes in life	Yes vs. *no*	0.11 (2.05)	[−4.12, 4.34]	0.05 (0.958)	42.07 (3.27) vs. *41.96 (3.78)*
Four criteria combination: AO + TRI + HBP + GLY	AO + TRI + HBP + GLY vs. *metabolically–healthy people*	4.37 (2.61)	[−1.01, 9.76]	1.68 (0.107)	44.20 (3.57) vs. *39.83 (3.68)*
Age at screening	Not applied (continuous covariate)	−1.39 (0.17)	[−1.74, −1.05]	−8.35 (< 0.001)	Not applied (continuous cov.)
Ratio of family income to the poverty level	Not applied (continuous covariate)	0.18 (0.62)	[−1.09, 1.45]	0.29 (0.777)	Not applied (continuous cov.)
Depression raw scores	Not applied (continuous covariate)	−0.05 (0.10)	[−0.25, 0.16]	−0.48 (0.637)	Not applied (continuous cov.)

[a]The design–based covariance matrix is singular. The validity of results is uncertain. [b]The column labeled "estimated marginal means" shows each category's marginal means against the reference category's marginal mean. The reference categories and the marginal means of the reference categories are shown in italics. The significant results have been colored.

Table S85. Multiple linear regression model explaining the relationship between DSST and specific combination AO + HDL + HBP + GLY after adjusting by sociodemographic characteristics and medical history, and estimated marginal means for each category of predictors and covariates[a]

Model coefficient	Categories (for categorical predictor and covariates)	*Beta* (*SE*)	95% CI	*t* (*p*)	Estimated marginal means[b]
Constant	Not applied (model intercept)	149.09 (15.50)	[116.52, 181.67]	9.62 (< 0.001)	Not applied (model intercept)
Education level	Up to 12th grade vs. *college graduate or above*	–10.62 (2.31)	[–15.48, –5.76]	–4.59 (< 0.001)	34.89 (2.87) vs. *45.51 (2.64)*
	High school graduate vs. *college graduate or above*	–7.11 (3.95)	[–15.40, 1.18]	–1.80 (0.088)	38.40 (5.11) vs. *45.51 (2.64)*
	Some college or AA degree vs. *college graduate or above*	–0.45 (2.55)	[–5.81, 4.91]	–0.18 (0.863)	45.06 (3.13) vs. *45.51 (2.64)*
Gender	Males vs. *females*	–7.09 (1.93)	[–11.13, –3.04]	–3.68 (0.002)	37.42 (3.23) vs. *44.51 (2.91)*
Race	Mexican American vs. *Non–Hispanic White*	–21.91 (2.96)	[–28.12, –15.70]	–7.41 (< 0.001)	32.28 (5.02) vs. *54.19 (4.02)*
	Other Hispanic vs. *Non–Hispanic White*	–18.93 (4.24)	[–27.84, –10.02]	–4.46 (< 0.001)	35.25 (4.05) vs. *54.19 (4.02)*
	Non–Hispanic Black vs. *Non–Hispanic White*	–17.58 (3.59)	[–25.11, –10.05]	–4.90 (< 0.001)	36.61 (2.26) vs. *54.19 (4.02)*
	Non–Hispanic Asian vs. *Non–Hispanic White*	6.21 (3.59)	[–1.33, 13.74]	1.73 (0.101)	60.39 (4.00) vs. *54.19 (4.02)*
	Other Race and Multi–Racial vs. *Non–Hispanic White*	–27.11 (5.18)	[–37.99, –16.23]	–5.23 (< 0.001)	27.08 (4.08) vs. *54.19 (4.02)*
Marital status	Single or never married vs. *married or living with a partner*	–1.91 (5.96)	[–14.42, 10.61]	–0.32 (0.752)	38.36 (5.72) vs. *40.27 (2.88)*
	Divorced or separated vs. *married or living with a partner*	–0.71 (3.29)	[–7.62, 6.19]	–0.22 (0.831)	39.56 (4.11) vs. *40.27 (2.88)*
	Widowed vs. *married or living with a partner*	5.40 (2.44)	[0.27, 10.53]	2.21 (0.040)	45.67 (4.23) vs. *40.27 (2.88)*
Self–report general health condition	Poor vs. *excellent or very good*	–7.10 (9.35)	[–26.75, 12.54]	–0.76 (0.457)	38.34 (7.65) vs. *45.44 (3.91)*
	Good or fair vs. *excellent or very good*	–6.33 (2.71)	[–12.01, –0.64]	–2.34 (0.031)	39.12 (3.22) vs. *45.44 (3.91)*
Difficulties in thinking or remembering	Yes vs. *no*	–5.20 (1.73)	[–8.84, –1.57]	–3.01 (0.008)	38.36 (3.38) vs. *43.57 (2.66)*
Ever told you have a heart disease	Yes vs. *no*	1.00 (3.96)	[–7.31, 9.31]	0.25 (0.803)	41.47 (3.04) vs. *40.47 (3.95)*
Ever told you had a stroke	Yes vs. *no*	9.14 (5.45)	[–2.31, 20.58]	1.68 (0.111)	45.53 (5.03) vs. *36.40 (2.56)*
Ever told you have diabetes	Yes vs. *no*	–3.72 (2.66)	[–9.30, 1.86]	–1.40 (0.179)	39.11 (3.73) vs. *42.82 (2.58)*
Have smoked at least 100 cigarettes in life	Yes vs. *no*	–1.14 (2.61)	[–6.63, 4.34]	–0.44 (0.667)	40.39 (3.33) vs. *41.54 (3.06)*
Four criteria combination: AO + HDL + HBP + GLY	AO + HDL + HBP + GLY vs. *metabolically–healthy people*	6.60 (3.40)	[–0.55, 13.74]	1.94 (0.068)	44.26 (3.26) vs. *37.67 (3.49)*
Age at screening	Not applied (continuous covariate)	–1.26 (0.22)	[–1.72, –0.81]	–5.81 (< 0.001)	Not applied (continuous cov.)
Ratio of family income to the poverty level	Not applied (continuous covariate)	–0.31 (0.76)	[–1.91, 1.28]	–0.41 (0.683)	Not applied (continuous cov.)
Depression raw scores	Not applied (continuous covariate)	0.29 (0.37)	[–0.49, 1.06]	0.78 (0.447)	Not applied (continuous cov.)

[a]The design–based covariance matrix is singular. The validity of results is uncertain. [b]The column labeled "estimated marginal means" shows each category's marginal means against the reference category's marginal mean. The reference categories and the marginal means of the reference categories are shown in italics. The significant results have been colored.

Table S86. Multiple linear regression model explaining the relationship between DSST and specific combination TRI + HDL + HBP + GLY after adjusting by sociodemographic characteristics and medical history, and estimated marginal means for each category of predictors and covariates[a]

**Model coefficient**	**Categories (for categorical predictor and covariates)**	*Beta* (*SE*)	95% CI	*t* (*p*)	Estimated marginal means[b]
Constant	Not applied (model intercept)	150.93 (15.29)	[117.90, 183.96]	9.87 (< 0.001)	Not applied (model intercept)
Education level	Up to 12th grade vs. *college graduate or above*	–15.30 (4.30)	[–24.59, –6.00]	–3.55 (0.004)	28.07 (9.07) vs. *43.37 (8.75)*
	High school graduate vs. *college graduate or above*	–8.27 (4.23)	[–17.42, 0.87]	–1.96 (0.072)	35.09 (7.61) vs. *43.37 (8.75)*
	Some college or AA degree vs. *college graduate or above*	–1.59 (2.68)	[–7.38, 4.20]	–0.59 (0.563)	41.78 (9.57) vs. *43.37 (8.75)*
Gender	Males vs. *females*	–7.48 (2.47)	[–12.81, –2.15]	–3.03 (0.010)	33.34 (8.24) vs. *40.82 (8.80)*
Race	Mexican American vs. *Non–Hispanic White*	–11.88 (3.64)	[–19.74, –4.02]	–3.27 (0.006)	30.69 (8.00) vs. *42.57 (7.80)*
	Other Hispanic vs. *Non–Hispanic White*	–7.39 (4.03)	[–16.10, 1.33]	–1.83 (0.090)	35.18 (8.46) vs. *42.57 (7.80)*
	Non–Hispanic Black vs. *Non–Hispanic White*	–18.79 (4.40)	[–28.30, –9.28]	–4.27 (0.001)	23.78 (8.76) vs. *42.57 (7.80)*
	Non–Hispanic Asian vs. *Non–Hispanic White*	14.59 (5.26)	[3.21, 25.96]	2.77 (0.016)	57.15 (9.04) vs. *42.57 (7.80)*
	Other Race and Multi–Racial vs. *Non–Hispanic White*	–9.48 (10.49)	[–32.13, 13.18]	–0.90 (0.383)	33.09 (15.30) vs. *42.57 (7.80)*
Marital status	Single or never married vs. *married or living with a partner*	–18.66 (7.80)	[–35.51, –1.80]	–2.39 (0.033)	20.63 (12.58) vs. *39.29 (7.36)*
	Divorced or separated vs. *married or living with a partner*	1.00 (3.98)	[–7.60, 9.60]	0.25 (0.806)	40.28 (9.23) vs. *39.29 (7.36)*
	Widowed vs. *married or living with a partner*	8.82 (3.53)	[1.21, 16.44]	2.50 (0.026)	48.11 (7.51) vs. *39.29 (7.36)*
Self–report general health condition	Poor vs. *excellent or very good*	–47.73 (16.05)	[–82.40, –13.07]	–2.97 (0.011)	8.06 (17.20) vs. *55.79 (5.58)*
	Good or fair vs. *excellent or very good*	–8.40 (3.29)	[–15.51, –1.29]	–2.55 (0.024)	47.39 (6.56) vs. *55.79 (5.58)*
Difficulties in thinking or remembering	Yes vs. *no*	–5.27 (2.59)	[–10.86, 0.32]	–2.04 (0.063)	34.44 (8.66) vs. *39.71 (8.41)*
Ever told you have a heart disease	Yes vs. *no*	0.39 (6.28)	[–13.17, 13.96]	0.06 (0.951)	37.27 (10.90) vs. *36.88 (6.58)*
Ever told you had a stroke	Yes vs. *no*	7.94 (5.07)	[–3.01, 18.90]	1.57 (0.141)	41.05 (10.08) vs. *33.11 (7.32)*
Ever told you have diabetes	Yes vs. *no*	–7.16 (5.97)	[–20.07, 5.74]	–1.20 (0.252)	33.50 (9.45) vs. *40.66 (8.42)*
Have smoked at least 100 cigarettes in life	Yes vs. *no*	1.25 (2.51)	[–4.17, 6.68]	0.50 (0.626)	37.70 (8.62) vs. *36.45 (8.44)*
Four criteria combination: TRI + HDL + HBP + GLY	TRI + HDL + HBP + GLY vs. *metabolically–healthy people*	21.82 (8.94)	[2.50, 41.14]	2.44 (0.030)	47.99 (9.17) vs. *26.17 (9.91)*
Age at screening	Not applied (continuous covariate)	–1.31 (0.19)	[–1.72, –0.89]	–6.81 (< 0.001)	Not applied (continuous cov.)
Ratio of family income to the poverty level	Not applied (continuous covariate)	–0.43 (0.76)	[–2.06, 1.21]	–0.56 (0.582)	Not applied (continuous cov.)
Depression raw scores	Not applied (continuous covariate)	1.26 (0.76)	[–0.37, 2.89]	1.67 (0.120)	Not applied (continuous cov.)

[a]The design–based covariance matrix is singular. The validity of results is uncertain. [b]The column labeled "estimated marginal means" shows each category's marginal means against the reference category's marginal mean. The reference categories and the marginal means of the reference categories are shown in italics. The significant results have been colored.

Table S87. Multiple linear regression model explaining the relationship between DSST and all combinations of four criteria after adjusting by sociodemographic characteristics and medical history, and estimated marginal means for each category of predictors and covariates

**Model coefficient**	**Categories (for categorical predictor and covariates)**	*Beta* (*SE*)	95% CI	*t* (*p*)	Estimated marginal means[a]
Constant	Not applied (model intercept)	137.82 (11.53)	[114.15, 161.48]	11.95 (< 0.001)	Not applied (model intercept)
Education level	Up to 12th grade vs. *college graduate or above*	–10.65 (2.31)	[–15.39, –5.91]	–4.61 (< 0.001)	32.95 (2.81) vs. 43.60 (3.29)
	High school graduate vs. *college graduate or above*	–1.09 (2.98)	[–7.19, 5.02]	–0.37 (0.718)	42.51 (2.52) vs. 43.60 (3.29)
	Some college or AA degree vs. *college graduate or above*	–2.22 (2.22)	[–6.78, 2.33]	–1.00 (0.326)	41.37 (2.98) vs. 43.60 (3.29)
Gender	Males vs. *females*	–6.21 (1.51)	[–9.31, –3.11]	–4.11 (< 0.001)	37.00 (2.72) vs. 43.21 (2.38)
Race	Mexican American vs. *Non–Hispanic White*	–12.88 (3.48)	[–20.02, –5.75]	–3.71 (0.001)	34.86 (3.20) vs. 47.74 (2.22)
	Other Hispanic vs. *Non–Hispanic White*	–17.42 (3.03)	[–23.62, –11.21]	–5.76 (< 0.001)	30.32 (4.06) vs. 47.74 (2.22)
	Non–Hispanic Black vs. *Non–Hispanic White*	–17.29 (3.71)	[–24.90, –9.68]	–4.66 (< 0.001)	30.45 (4.06) vs. 47.74 (2.22)
	Non–Hispanic Asian vs. *Non–Hispanic White*	2.25 (3.87)	[–5.68, 10.19]	0.58 (0.565)	50.00 (4.67) vs. 47.74 (2.22)
	Other Race and Multi–Racial vs. *Non–Hispanic White*	–0.47 (3.30)	[–7.24, 6.29]	–0.14 (0.887)	47.27 (3.30) vs. 47.74 (2.22)
Marital status	Single or never married vs. *married or living with a partner*	–5.81 (3.14)	[–12.26, 0.64]	–1.85 (0.076)	35.57 (4.04) vs. 41.38 (2.24)
	Divorced or separated vs. *married or living with a partner*	–0.43 (2.03)	[–4.61, 3.74]	–0.21 (0.832)	40.95 (2.19) vs. 41.38 (2.24)
	Widowed vs. *married or living with a partner*	1.14 (2.84)	[–4.68, 6.96]	0.40 (0.691)	42.52 (3.73) vs. 41.38 (2.24)
Self–report general health condition	Poor vs. *excellent or very good*	–11.45 (5.43)	[–22.59, –0.30]	–2.11 (0.045)	33.59 (4.70) vs. 45.04 (2.82)
	Good or fair vs. *excellent or very good*	–3.36 (1.67)	[–6.78, 0.06]	–2.01 (0.054)	41.68 (2.79) vs. 45.04 (2.82)
Difficulties in thinking or remembering	Yes vs. *no*	–4.14 (1.97)	[–8.19, –0.09]	–2.10 (0.045)	38.03 (3.08) vs. 42.18 (2.09)
Ever told you have a heart disease	Yes vs. *no*	0.29 (2.86)	[–5.58, 6.17]	0.10 (0.920)	40.25 (3.27) vs. 39.96 (2.31)
Ever told you had a stroke	Yes vs. *no*	0.30 (2.93)	[–5.72, 6.32]	0.10 (0.919)	40.26 (3.20) vs. 39.96 (2.44)
Ever told you have diabetes	Yes vs. *no*	1.43 (1.93)	[–2.53, 5.38]	0.74 (0.465)	40.82 (2.63) vs. 39.39 (2.62)
Have smoked at least 100 cigarettes in life	Yes vs. *no*	–3.31 (1.99)	[–7.38, 0.77]	–1.66 (0.108)	38.45 (2.59) vs. 41.76 (2.68)
All combinations of four criteria	All combinations of four criteria vs. *metabolically–healthy people*	4.58 (2.11)	[0.25, 8.92]	2.17 (0.039)	42.40 (2.69) vs. 37.81 (2.62)
Age at screening	Not applied (continuous covariate)	–1.12 (0.16)	[–1.44, –0.79]	–7.04 (< 0.001)	Not applied (continuous cov.)
Ratio of family income to the poverty level	Not applied (continuous covariate)	0.33 (0.62)	[–0.94, 1.59]	0.53 (0.603)	Not applied (continuous cov.)
Depression raw scores	Not applied (continuous covariate)	–0.20 (0.12)	[–0.45, 0.05]	–1.67 (0.107)	Not applied (continuous cov.)

[a]The column labeled "estimated marginal means" shows each category's marginal means against the reference category's marginal mean. The reference categories and the marginal means of the reference categories are shown in italics. The significant results have been colored.

Table S88. Multiple linear regression model explaining the relationship between DSST and combination of five criteria after adjusting by sociodemographic characteristics and medical history, and estimated marginal means for each category of predictors and covariates[a]

**Model coefficient**	**Categories (for categorical predictor and covariates)**	*Beta* (*SE*)	95% CI	*t* (*p*)	Estimated marginal means[b]
Constant	Not applied (model intercept)	121.37 (11.82)	[96.72, 146.02]	10.27 (< 0.001)	Not applied (model intercept)
Education level	Up to 12th grade vs. *college graduate or above*	–15.53 (3.29)	[–22.39, –8.67]	–4.72 (< 0.001)	22.24 (4.41) vs. *37.77 (4.86)*
	High school graduate vs. *college graduate or above*	–6.19 (3.09)	[–12.63, 0.25]	–2.01 (0.059)	31.58 (5.13) vs. *37.77 (4.86)*
	Some college or AA degree vs. *college graduate or above*	–0.23 (2.33)	[–5.11, 4.64]	–0.10 (0.921)	37.54 (4.90) vs. *37.77 (4.86)*
Gender	Males vs. *females*	–4.32 (1.54)	[–7.53, –1.11]	–2.81 (0.011)	30.12 (4.67) vs. *34.44 (4.38)*
Race	Mexican American vs. *Non–Hispanic White*	–12.18 (4.40)	[–21.36, –2.99]	–2.77 (0.012)	25.86 (5.15) vs. *38.04 (3.94)*
	Other Hispanic vs. *Non–Hispanic White*	–13.87 (6.47)	[–27.36, –0.38]	–2.14 (0.044)	24.17 (7.68) vs. *38.04 (3.94)*
	Non–Hispanic Black vs. *Non–Hispanic White*	–11.03 (3.08)	[–17.45, –4.60]	–3.58 (0.002)	27.01 (4.56) vs. *38.04 (3.94)*
	Non–Hispanic Asian vs. *Non–Hispanic White*	8.31 (4.27)	[–0.61, 17.22]	1.94 (0.066)	46.34 (6.61) vs. *38.04 (3.94)*
	Other Race and Multi–Racial vs. *Non–Hispanic White*	No cases	No cases	No cases	No cases
Marital status	Single or never married vs. *married or living with a partner*	–7.93 (8.45)	[–25.55, 9.69]	–0.94 (0.359)	26.25 (9.78) vs. *34.18 (3.45)*
	Divorced or separated vs. *married or living with a partner*	–0.75 (2.48)	[–5.92, 4.42]	–0.30 (0.765)	33.43 (4.25) vs. *34.18 (3.45)*
	Widowed vs. *married or living with a partner*	1.11 (2.16)	[–3.40, 5.62]	0.51 (0.614)	35.28 (4.23) vs. *34.18 (3.45)*
Self–report general health condition	Poor vs. *excellent or very good*	–10.56 (4.34)	[–19.62, –1.50]	–2.43 (0.025)	27.39 (5.87) vs. *37.95 (4.36)*
	Good or fair vs. *excellent or very good*	–6.43 (2.60)	[–11.84, –1.01]	–2.48 (0.022)	31.52 (4.44) vs. *37.95 (4.36)*
Difficulties in thinking or remembering	Yes vs. *no*	–4.13 (3.14)	[–10.68, 2.42]	–1.32 (0.203)	30.22 (4.07) vs. *34.35 (5.31)*
Ever told you have a heart disease	Yes vs. *no*	0.50 (3.41)	[–6.60, 7.60]	0.15 (0.885)	32.53 (5.40) vs. *32.03 (4.06)*
Ever told you had a stroke	Yes vs. *no*	–12.67 (4.90)	[–22.89, –2.46]	–2.59 (0.018)	25.95 (6.48) vs. *38.62 (3.14)*
Ever told you have diabetes	Yes vs. *no*	–3.93 (3.00)	[–10.20, 2.33]	–1.31 (0.205)	30.32 (5.24) vs. *34.25 (4.12)*
Have smoked at least 100 cigarettes in life	Yes vs. *no*	–2.40 (2.29)	[–7.18, 2.38]	–1.05 (0.308)	31.08 (4.60) vs. *33.48 (4.62)*
Combination of five criteria	AO + TRI + HDL + HBP + GLY vs. *metabolically–healthy people*	2.92 (1.65)	[–0.53, 6.38]	1.77 (0.092)	33.75 (4.75) vs. *30.82 (4.32)*
Age at screening	Not applied (continuous covariate)	–0.84 (0.16)	[–1.18, –0.50]	–5.15 (< 0.001)	Not applied (continuous cov.)
Ratio of family income to the poverty level	Not applied (continuous covariate)	–0.61 (0.69)	[–2.06, 0.84]	–0.88 (0.389)	Not applied (continuous cov.)
Depression raw scores	Not applied (continuous covariate)	0.09 (0.33)	[–0.59, 0.77]	0.27 (0.789)	Not applied (continuous cov.)

[a]The design–based covariance matrix is singular. The validity of results is uncertain. [b]The column labeled "estimated marginal means" shows each category's marginal means against the reference category's marginal mean. The reference categories and the marginal means of the reference categories are shown in italics. The significant results have been colored.

Table S89. Multiple linear regression model explaining the relationship between DSST and all combination with abdominal obesity after adjusting by sociodemographic characteristics and medical history, and estimated marginal means for each category of predictors and covariates

**Model coefficient**	**Categories (for categorical predictor and covariates)**	*Beta* (*SE*)	95% CI	*t* (*p*)	Estimated marginal means[a]
Constant	Not applied (model intercept)	128.34 (8.24)	[111.54, 145.14]	15.58 (< 0.001)	Not applied (model intercept)
Education level	Up to 12th grade vs. *college graduate or above*	−14.29 (2.11)	[−18.58, −9.99]	−6.79 (< 0.001)	32.44 (2.22) vs. *46.73 (2.68)*
	High school graduate vs. *college graduate or above*	−6.68 (2.33)	[−11.44, −1.93]	−2.87 (0.007)	40.04 (2.07) vs. *46.73 (2.68)*
	Some college or AA degree vs. *college graduate or above*	−5.15 (1.77)	[−8.76, −1.53]	−2.90 (0.007)	41.58 (1.77) vs. *46.73 (2.68)*
Gender	Males vs. *females*	−4.70 (1.41)	[−7.57, −1.83]	−3.34 (0.002)	37.85 (2.05) vs. *42.55 (1.96)*
Race	Mexican American vs. *Non–Hispanic White*	−11.86 (2.18)	[−16.30, −7.42]	−5.45 (< 0.001)	34.57 (2.64) vs. *46.44 (1.54)*
	Other Hispanic vs. *Non–Hispanic White*	−13.56 (2.29)	[−18.24, −8.88]	−5.91 (< 0.001)	32.88 (2.84) vs. *46.44 (1.54)*
	Non–Hispanic Black vs. *Non–Hispanic White*	−12.80 (1.84)	[−16.55, −9.05]	−6.97 (< 0.001)	33.64 (2.54) vs. *46.44 (1.54)*
	Non–Hispanic Asian vs. *Non–Hispanic White*	2.36 (2.08)	[−1.87, 6.59]	1.14 (0.265)	48.80 (2.66) vs. *46.44 (1.54)*
	Other Race and Multi–Racial vs. *Non–Hispanic White*	−1.57 (2.98)	[−7.64, 4.50]	−0.53 (0.602)	44.87 (3.59) vs. *46.44 (1.54)*
Marital status	Single or never married vs. *married or living with a partner*	0.33 (2.60)	[−4.97, 5.63]	0.13 (0.899)	41.38 (3.12) vs. *41.05 (2.01)*
	Divorced or separated vs. *married or living with a partner*	−1.95 (1.64)	[−5.30, 1.40]	−1.19 (0.244)	39.10 (2.46) vs. *41.05 (2.01)*
	Widowed vs. *married or living with a partner*	−1.79 (2.28)	[−6.44, 2.86]	−0.79 (0.438)	39.26 (2.40) vs. *41.05 (2.01)*
Self–report general health condition	Poor vs. *excellent or very good*	−1.05 (3.10)	[−7.38, 5.28]	−0.34 (0.738)	39.89 (3.20) vs. *40.93 (1.96)*
	Good or fair vs. *excellent or very good*	−1.16 (1.48)	[−4.18, 1.87]	−0.78 (0.442)	39.78 (1.83) vs. *40.93 (1.96)*
Difficulties in thinking or remembering	Yes vs. *no*	−7.93 (1.72)	[−11.43, −4.43]	−4.61 (< 0.001)	36.23 (2.34) vs. *44.16 (1.74)*
Ever told you have a heart disease	Yes vs. *no*	−0.37 (2.19)	[−4.83, 4.08]	−0.17 (0.865)	40.01 (2.48) vs. *40.39 (1.82)*
Ever told you had a stroke	Yes vs. *no*	−0.66 (2.11)	[−4.97, 3.65]	−0.31 (0.758)	39.87 (2.23) vs. *40.53 (2.07)*
Ever told you have diabetes	Yes vs. *no*	−2.62 (1.42)	[−5.52, 0.28]	−1.84 (0.075)	38.89 (1.97) vs. *41.51 (2.04)*
Have smoked at least 100 cigarettes in life	Yes vs. *no*	−1.20 (1.38)	[−4.02, 1.61]	−0.87 (0.390)	39.60 (1.95) vs. *40.80 (2.05)*
All combinations with abdominal obesity	Combinations with AO vs. *metabolically–healthy people*	3.28 (2.41)	[−1.63, 8.19]	1.36 (0.183)	41.84 (1.72) vs. *38.56 (2.64)*
Age at screening	Not applied (continuous covariate)	−0.96 (0.13)	[−1.23, −0.70]	−7.37 (< 0.001)	Not applied (continuous cov.)
Ratio of family income to the poverty level	Not applied (continuous covariate)	0.05 (0.52)	[−1.01, 1.12]	0.11 (0.917)	Not applied (continuous cov.)
Depression raw scores	Not applied (continuous covariate)	−0.15 (0.11)	[−0.37, 0.07]	−1.36 (0.183)	Not applied (continuous cov.)

[a]The column labeled "estimated marginal means" shows each category's marginal means against the reference category's marginal mean. The reference categories and the marginal means of the reference categories are shown in italics. The significant results have been colored.

Table S90. Multiple linear regression model explaining the relationship between DSST and all combinations without abdominal obesity after adjusting by sociodemographic characteristics and medical history, and estimated marginal means for each category of predictors and covariates[a]

**Model coefficient**	**Categories (for categorical predictor and covariates)**	*Beta* (*SE*)	95% CI	*t* (*p*)	Estimated marginal means[b]
Constant	Not applied (model intercept)	145.97 (15.56)	[113.51, 178.44]	9.38 (< 0.001)	Not applied (model intercept)
Education level	Up to 12th grade vs. *college graduate or above*	–6.27 (4.32)	[–15.28, 2.75]	–1.45 (0.162)	33.37 (6.54) vs. *39.64 (7.62)*
	High school graduate vs. *college graduate or above*	–6.16 (4.75)	[–16.08, 3.76]	–1.30 (0.210)	33.48 (6.14) vs. *39.64 (7.62)*
	Some college or AA degree vs. *college graduate or above*	2.71 (2.19)	[–1.85, 7.27]	1.24 (0.230)	42.35 (7.36) vs. *39.64 (7.62)*
Gender	Males vs. *females*	–8.86 (2.53)	[–14.14, –3.59]	–3.51 (0.002)	32.78 (6.68) vs. *41.64 (6.32)*
Race	Mexican American vs. *Non–Hispanic White*	–16.87 (3.41)	[–23.99, –9.76]	–4.95 (< 0.001)	26.65 (7.11) vs. *43.52 (5.38)*
	Other Hispanic vs. *Non–Hispanic White*	–23.93 (10.75)	[–46.36, –1.50]	–2.23 (0.038)	19.59 (12.09) vs. *43.52 (5.38)*
	Non–Hispanic Black vs. *Non–Hispanic White*	–12.94 (4.26)	[–21.83, –4.05]	–3.04 (0.007)	30.58 (7.37) vs. *43.52 (5.38)*
	Non–Hispanic Asian vs. *Non–Hispanic White*	7.19 (4.69)	[–2.60, 16.98]	1.53 (0.141)	50.70 (7.27) vs. *43.52 (5.38)*
	Other Race and Multi–Racial vs. *Non–Hispanic White*	8.69 (6.10)	[–4.04, 21.42]	1.42 (0.170)	52.21 (8.65) vs. *43.52 (5.38)*
Marital status	Single or never married vs. *married or living with a partner*	–9.29 (7.98)	[–25.94, 7.36]	–1.16 (0.258)	26.64 (10.34) vs. *35.93 (5.47)*
	Divorced or separated vs. *married or living with a partner*	9.39 (3.37)	[2.37, 16.42]	2.79 (0.011)	45.32 (6.71) vs. *35.93 (5.47)*
	Widowed vs. *married or living with a partner*	5.02 (3.34)	[–1.94, 11.98]	1.50 (0.148)	40.95 (6.62) vs. *35.93 (5.47)*
Self–report general health condition	Poor vs. *excellent or very good*	–15.25 (8.56)	[–33.10, 2.60]	–1.78 (0.090)	29.35 (8.58) vs. *44.60 (7.13)*
	Good or fair vs. *excellent or very good*	–6.92 (3.93)	[–15.12, 1.28]	–1.76 (0.094)	37.68 (6.67) vs. *44.60 (7.13)*
Difficulties in thinking or remembering	Yes vs. *no*	–5.36 (3.80)	[–13.29, 2.57]	–1.41 (0.174)	34.53 (7.24) vs. *39.89 (6.01)*
Ever told you have a heart disease	Yes vs. *no*	0.21 (6.35)	[–13.04, 13.45]	0.03 (0.974)	37.31 (9.11) vs. *37.10 (4.29)*
Ever told you had a stroke	Yes vs. *no*	–1.26 (6.95)	[–15.75, 13.23]	–0.18 (0.858)	36.58 (8.15) vs. *37.84 (6.25)*
Ever told you have diabetes	Yes vs. *no*	–10.02 (5.07)	[–20.59, 0.55]	–1.98 (0.062)	32.20 (7.18) vs. *42.22 (6.52)*
Have smoked at least 100 cigarettes in life	Yes vs. *no*	1.14 (2.88)	[–4.87, 7.15]	0.40 (0.697)	37.78 (5.89) vs. *36.64 (7.13)*
All combinations without abdominal obesity	Combinations without AO vs. *metabolically–healthy people*	7.55 (4.36)	[–1.54, 16.64]	1.73 (0.098)	40.98 (7.77) vs. *33.43 (5.52)*
Age at screening	Not applied (continuous covariate)	–1.20 (0.21)	[–1.63, –0.76]	–5.71 (< 0.001)	Not applied (continuous cov.)
Ratio of family income to the poverty level	Not applied (continuous covariate)	–1.41 (0.87)	[–3.22, 0.39]	–1.63 (0.118)	Not applied (continuous cov.)
Depression raw scores	Not applied (continuous covariate)	–0.28 (0.56)	[–1.45, 0.90]	–0.50 (0.625)	Not applied (continuous cov.)

[a]The design–based covariance matrix is singular. The validity of results is uncertain. [b]The column labeled "estimated marginal means" shows each category's marginal means against the reference category's marginal mean. The reference categories and the marginal means of the reference categories are shown in italics. The significant results have been colored.

Table S91. Multiple linear regression model explaining the relationship between DSST and all combinations with high glycemia obesity after adjusting by sociodemographic characteristics and medical history, and estimated marginal means for each category of predictors and covariates

**Model coefficient**	**Categories (for categorical predictor and covariates)**	*Beta* (*SE*)	95% CI	*t* (*p*)	Estimated marginal means[a]
Constant	Not applied (model intercept)	123.65 (8.32)	[106.71, 140.60]	14.86 (< 0.001)	Not applied (model intercept)
Education level	Up to 12th grade vs. *college graduate or above*	–13.51 (2.15)	[–17.90, –9.12]	–6.27 (< 0.001)	32.61 (2.22) vs. *46.12 (2.54)*
	High school graduate vs. *college graduate or above*	–5.53 (2.29)	[–10.20, –0.86]	–2.41 (0.022)	40.59 (2.14) vs. *46.12 (2.54)*
	Some college or AA degree vs. *college graduate or above*	–3.85 (1.82)	[–7.55, –0.14]	–2.12 (0.042)	42.27 (1.76) vs. *46.12 (2.54)*
Gender	Males vs. *females*	–4.41 (1.36)	[–7.18, –1.63]	–3.23 (0.003)	38.20 (1.97) vs. *42.60 (1.90)*
Race	Mexican American vs. *Non–Hispanic White*	–11.69 (2.05)	[–15.85, –7.52]	–5.71 (< 0.001)	34.59 (2.53) vs. *46.28 (1.54)*
	Other Hispanic vs. *Non–Hispanic White*	–13.31 (2.12)	[–17.63, –9.00]	–6.29 (< 0.001)	32.96 (2.85) vs. *46.28 (1.54)*
	Non–Hispanic Black vs. *Non–Hispanic White*	–12.43 (1.76)	[–16.02, –8.84]	–7.06 (< 0.001)	33.85 (2.15) vs. *46.28 (1.54)*
	Non–Hispanic Asian vs. *Non–Hispanic White*	1.39 (2.10)	[–2.88, 5.66]	0.66 (0.512)	47.67 (2.81) vs. *46.28 (1.54)*
	Other Race and Multi–Racial vs. *Non–Hispanic White*	0.78 (2.41)	[–4.12, 5.68]	0.32 (0.748)	47.05 (2.90) vs. *46.28 (1.54)*
Marital status	Single or never married vs. *married or living with a partner*	1.12 (2.70)	[–4.37, 6.61]	0.42 (0.681)	41.45 (3.27) vs. *40.33 (2.04)*
	Divorced or separated vs. *married or living with a partner*	0.74 (2.40)	[–4.14, 5.62]	0.31 (0.759)	41.07 (2.65) vs. *40.33 (2.04)*
	Widowed vs. *married or living with a partner*	–1.58 (2.34)	[–6.34, 3.18]	–0.67 (0.505)	38.75 (2.39) vs. *40.33 (2.04)*
Self–report general health condition	Poor vs. *excellent or very good*	–1.83 (3.07)	[–8.09, 4.42]	–0.60 (0.555)	39.62 (3.01) vs. *41.45 (2.03)*
	Good or fair vs. *excellent or very good*	–1.33 (1.70)	[–4.79, 2.12]	–0.79 (0.437)	40.12 (1.75) vs. *41.45 (2.03)*
Difficulties in thinking or remembering	Yes vs. *no*	–7.01 (1.33)	[–9.72, –4.31]	–5.28 (< 0.001)	36.89 (2.05) vs. *43.90 (1.80)*
Ever told you have a heart disease	Yes vs. *no*	–0.62 (2.45)	[–5.61, 4.36]	–0.25 (0.801)	40.09 (2.57) vs. *40.71 (1.71)*
Ever told you had a stroke	Yes vs. *no*	–1.96 (2.25)	[–6.53, 2.62]	–0.87 (0.390)	39.42 (2.27) vs. *41.38 (1.98)*
Ever told you have diabetes	Yes vs. *no*	–2.78 (1.59)	[–6.03, 0.47]	–1.74 (0.091)	39.01 (1.98) vs. *41.79 (1.98)*
Have smoked at least 100 cigarettes in life	Yes vs. *no*	–1.78 (1.47)	[–4.78, 1.21]	–1.21 (0.234)	39.51 (1.92) vs. *41.29 (1.99)*
All combinations with high glycemia	Combinations with GLY vs. *metabolically–healthy people*	3.26 (2.52)	[–1.88, 8.40]	1.29 (0.205)	42.03 (1.70) vs. *38.77 (2.61)*
Age at screening	Not applied (continuous covariate)	–0.90 (0.14)	[–1.17, –0.62]	–6.60 (< 0.001)	Not applied (continuous cov.)
Ratio of family income to the poverty level	Not applied (continuous covariate)	–0.06 (0.57)	[–1.22, 1.10]	–0.11 (0.914)	Not applied (continuous cov.)
Depression raw scores	Not applied (continuous covariate)	–0.25 (0.16)	[–0.57, 0.07]	–1.59 (0.121)	Not applied (continuous cov.)

[a]The column labeled "estimated marginal means" shows each category's marginal means against the reference category's marginal mean. The reference categories and the marginal means of the reference categories are shown in italics. The significant results have been colored.

Table S92. Multiple linear regression model explaining the relationship between DSST and all combinations without high glycemia obesity after adjusting by sociodemographic characteristics and medical history, and estimated marginal means for each category of predictors and covariates[a]

**Model coefficient**	**Categories (for categorical predictor and covariates)**	*Beta* (*SE*)	95% CI	*t* (*p*)	Estimated marginal means[b]
Constant	Not applied (model intercept)	167.08 (11.37)	[143.10, 191.05]	14.70 (< 0.001)	Not applied (model intercept)
Education level	Up to 12th grade vs. *college graduate or above*	–13.32 (3.11)	[–19.87, –6.76]	–4.28 (0.001)	34.02 (3.91) vs. *47.33 (4.95)*
	High school graduate vs. *college graduate or above*	–10.66 (4.02)	[–19.15, –2.18]	–2.65 (0.017)	36.67 (3.88) vs. *47.33 (4.95)*
	Some college or AA degree vs. *college graduate or above*	–4.89 (3.13)	[–11.49, 1.71]	–1.56 (0.136)	42.44 (4.01) vs. *47.33 (4.95)*
Gender	Males vs. *females*	–9.22 (2.30)	[–14.07, –4.36]	–4.00 (0.001)	35.51 (4.00) vs. *44.72 (3.72)*
Race	Mexican American vs. *Non–Hispanic White*	–15.36 (2.53)	[–20.70, –10.02]	–6.07 (< 0.001)	32.77 (4.03) vs. *48.12 (3.61)*
	Other Hispanic vs. *Non–Hispanic White*	–21.93 (5.28)	[–33.08, –10.79]	–4.15 (0.001)	26.19 (5.26) vs. *48.12 (3.61)*
	Non–Hispanic Black vs. *Non–Hispanic White*	–14.56 (2.64)	[–20.13, –8.98]	–5.51 (< 0.001)	33.57 (4.34) vs. *48.12 (3.61)*
	Non–Hispanic Asian vs. *Non–Hispanic White*	11.26 (3.54)	[3.79, 18.74]	3.18 (0.005)	59.39 (6.10) vs. *48.12 (3.61)*
	Other Race and Multi–Racial vs. *Non–Hispanic White*	–7.47 (4.26)	[–16.46, 1.52]	–1.75 (0.098)	40.65 (5.42) vs. *48.12 (3.61)*
Marital status	Single or never married vs. *married or living with a partner*	–15.05 (6.60)	[–28.98, –1.13]	–2.28 (0.036)	28.81 (7.99) vs. *43.86 (3.59)*
	Divorced or separated vs. *married or living with a partner*	–4.64 (3.78)	[–12.62, 3.34]	–1.23 (0.237)	39.22 (2.28) vs. *43.86 (3.59)*
	Widowed vs. *married or living with a partner*	4.71 (2.41)	[–0.37, 9.79]	1.95 (0.067)	48.57 (4.92) vs. *43.86 (3.59)*
Self–report general health condition	Poor vs. *excellent or very good*	–8.55 (4.53)	[–18.11, 1.01]	–1.89 (0.076)	35.44 (3.89) vs. *43.99 (4.59)*
	Good or fair vs. *excellent or very good*	–3.08 (2.39)	[–8.13, 1.97]	–1.29 (0.216)	40.91 (4.54) vs. *43.99 (4.59)*
Difficulties in thinking or remembering	Yes vs. *no*	–8.07 (2.94)	[–14.28, –1.85]	–2.74 (0.014)	36.08 (4.55) vs. *44.15 (3.28)*
Ever told you have a heart disease	Yes vs. *no*	3.28 (5.20)	[–7.69, 14.25]	0.63 (0.536)	41.76 (5.93) vs. *38.47 (2.35)*
Ever told you had a stroke	Yes vs. *no*	10.45 (4.84)	[0.24, 20.65]	2.16 (0.045)	45.34 (5.38) vs. *34.89 (3.14)*
Ever told you have diabetes	Yes vs. *no*	–3.75 (3.45)	[–11.03, 3.53]	–1.09 (0.292)	38.24 (4.03) vs. *41.99 (4.10)*
Have smoked at least 100 cigarettes in life	Yes vs. *no*	0.68 (2.65)	[–4.92, 6.28]	0.26 (0.800)	40.46 (3.31) vs. *39.77 (4.44)*
All combinations without high glycemia	Combinations without GLY vs. *metabolically–healthy people*	1.92 (2.84)	[–4.09, 7.92]	0.67 (0.510)	41.07 (4.79) vs. *39.16 (2.86)*
Age at screening	Not applied (continuous covariate)	–1.50 (0.19)	[–1.89, –1.11]	–8.06 (< 0.001)	Not applied (continuous cov.)
Ratio of family income to the poverty level	Not applied (continuous covariate)	–0.47 (0.97)	[–2.51, 1.57]	–0.49 (0.631)	Not applied (continuous cov.)
Depression raw scores	Not applied (continuous covariate)	0.20 (0.20)	[–0.23, 0.62]	0.98 (0.341)	Not applied (continuous cov.)

[a]The design–based covariance matrix is singular. The validity of results is uncertain. [b]The column labeled "estimated marginal means" shows each category's marginal means against the reference category's marginal mean. The reference categories and the marginal means of the reference categories are shown in italics. The significant results have been colored.
